# Metabolomics investigation of dietary effects on flesh quality in grass carp (*Ctenopharyngodon idellus*)

**DOI:** 10.1093/gigascience/giy111

**Published:** 2018-09-06

**Authors:** Honghao Zhao, Jasmine Chong, Rong Tang, Li Li, Jianguo Xia, Dapeng Li

**Affiliations:** 1 College of Fisheries, Hubei Provincial Engineering Laboratory for Pond Aquaculture, National Demonstration Center for Experimental Aquaculture Education, Huazhong Agricultural University, Wuhan 430070, China; 2 Institute of Parasitology, McGill University, Saint-Anne-de-Bellevue, QC H9X 3V9, Canada; 3 Department of Animal Science, McGill University, Saint-Anne-de-Bellevue, QC H9X 3V9, Canada

**Keywords:** diets, metabolomics, flesh quality, fat deposition, *Ctenopharyngodon idellus*

## Abstract

**Background:**

The ultrahigh density intensive farming model of grass carp (*Ctenopharyngodon idellus*) may elicit growth inhibition, decrease flesh quality, and increase disease susceptibility of fish. The degradation in quality and excessive fat accumulation in cultured *C. idellus* have long been attributed to possible alterations in the lipid metabolism of fish muscle tissues as a result of overnutrition from artificial diets. To investigate the effects of different diets on fish muscle quality, a large-scale metabolomics study was performed on 250 tails of *C. idellus*.

**Findings:**

The experimental fish were divided into four groups based on sex and diet—female artificial feed (FAF), female grass feed, male artificial feed (MAF), and male grass feed (MGF). After a 113-day rearing period, the artificial feed (AF) group showed a significantly higher total mass of muscle fat (*P* < 0.01), with the FAF group being the highest. Metabolomics profiling based on liquid chromatography-mass spectrometry revealed distinctive patterns of clustering according to the four groups. Overall, artificial feeding was associated with higher concentrations of docosapentaenoic acid, dihomo-gamma-linolenic acid, and arachidonic acid, whereas grass feeding was associated with elevated n-3 unsaturated fatty acids (UFAs) such as eicosapentaenoic acid, alpha-linolenic acid, and gamma-linolenic acid. Artificial feeding also resulted in significant increased docosahexaenoic acid in MAF muscle than in MGF fish, whereas there was no significance in the comparison of female samples. Metabolic pathway analyses using both targeted and untargeted approaches consistently revealed that arachidonic acid metabolism and steroid hormone biosynthesis pathways were significantly different between AF and grass fed groups.

**Conclusions:**

Our results suggest that grass is a better source of dietary fatty acid and protein when compared to artificial feed. Grass feeding could effectively lower triglycerides in serum, reduce fat accumulation, and alter lipid compositions in fish muscle by increasing the concentrations of n-3 UFAs, leading to better nutrition and health.

## Background

Grass carp (*Ctenopharyngodon idellus*) is an important freshwater aquaculture fish species worldwide, accounting for 7.6% (5.8 million tons in 2015) of total global freshwater aquaculture production [1]. Intensive fish farming based on the utilization of artificially formulated feeds has played a critical role in the continuing increase in fish production [2]. However, the flesh quality of farmed *C. idellus* has declined during the course of intensive aquaculture, which has become a growing public concern [3]. It is now generally agreed that production improvement should no longer be the primary goal in aquaculture practice. Obtaining high-quality fish products while maintaining a sustainable aquaculture has become an important objective for the industry [4, 5].

One approach to obtaining sustainable aquaculture is to make full use of new technologies available to the scientific community. Over the past few years, high-throughput omics technologies, such as genomics, transcriptomics, proteomics, and metabolomics, have been used to enable a detailed understanding of molecular changes in different organisms, showing great potential to transform aquaculture research [6, 7]. Metabolomics is the systematic study of all small molecules in a biological system, such as cells, biofluids, and tissues. Global (or untargeted) metabolomics is particularly suitable for comprehensive metabolome characterization and novel biomarker discovery. High-resolution mass spectrometry (MS) systems coupled with liquid chromatography (LC) have become the main workhorse in global metabolomics [8]. It has been widely applied to understand the effects of diets and nutrition strategies for disease prevention and treatment in human populations. These studies have revealed that the high ratio of n-3/n-6 polyunsaturated fatty acids (PUFAs) in diets had protective effects on the risk of obesity, breast cancer, and hypertriglyceridemia [9-11]. These findings have important implications for animal nutrition research that aims to enhance the ratio of n-3/n-6 PUFA in milk and meat products. For instance, Bertol et al. have shown that the concentrations of n-3 PUFAs were significantly higher in the meat of pigs fed canola or canola plus flax oil diets compared to pigs fed a soybean oil diet [12]. A similar study on cows demonstrated that milk from cows fed with grass enriched in n-3 fatty acids (FAs) contained more n-3 fatty acids than milk from cows fed with conserved grass [13]. Metabolomics has also increasingly contributed to our understanding of the effect of different diets or dietary patterns on fish [6, 14]. For instance, feeding plankton to carp was shown to enhance the content of n-3 PUFAs, especially eicosapentaenoic acid (EPA) and docosahexaenoic acid (DHA), whereas feeding carp a diet with rapeseed induced higher oleic acid levels and lower levels of n-3 PUFAs [14]. Metabolomics was also used to explore the possibility of replacing the fishmeal component in artificial diets with zygomycetes, as well as to compare the FA compositions between artificial farmed fish and wild fish [15, 16]. It is noteworthy that high dietary levels of n-3 PUFA not only increased percentages of n-3 PUFAs in liver lipids but also increased the incidence of oxidative stress, characterized by reducing activity of β-oxidation capacity, together with elevated activities of superoxide dismutase and caspase-3 [17]. The lipids β-oxidation in muscles is believed to be responsible for lipid accumulation, lower nutritional quality, and modification of the texture and color of meat [18, 19]. Moreover, dietary EPA supplementation has been reported to reduce FA oxidation, which also facilitates the accumulation of EPA and decreases the total n-3:n-6 ratio [20].

It is widely accepted that long-term feeding of artificial diets likely contributes to the decline of fish flesh taste [21]. However, few studies have investigated the dietary effects on fish muscle metabolism and possible associations between changes in fish flesh quality characteristics and metabolic alterations. In this study, the effects of different diets, artificial feed (AF) and natural grass (GF), were investigated in *C. idellus*. Since sex can easily influence metabolomics data, metabolic profiles were separated by sex (female GF [FGF] vs female AF [FAF]; male GF [MGF] vs male AF [MAF]) in order to better evaluate the footprint of each diet on muscle quality. After a 113-day period of feeding, lipid mass in muscles and muscle fiber characteristics were examined, followed by a comprehensive untargeted metabolomic profiling of fish muscles using LC-MS. Finally, serum levels of total cholesterol (TCHO), high-density cholesterol (HDLC), glucose (GLU), total protein (TP), and triglycerides (TG) were used to determine if physiological and biochemical indicators were consistent with metabolic changes. Significantly different metabolites and mass peaks between the experimental groups were further examined using pathway analysis to gain a better understanding of the effects of diets on metabolic alterations and muscle quality in *C. idellus*.

## Data Description

In this study, we conducted a comprehensive physiological, biochemical, and metabolomic investigation of the effects of artificial and grass feeding on *C. idellus* flesh quality. After 113 days of separate feeding, muscle samples were collected from the two groups. At the same time, because of metabolite sensitivity and sex specificity, metabolomic analyses of muscle samples were divided into four test groups based on the results of the sex determination (n = 10), including FGF, MGF, FAF, and MAF.

**Figure 1:**
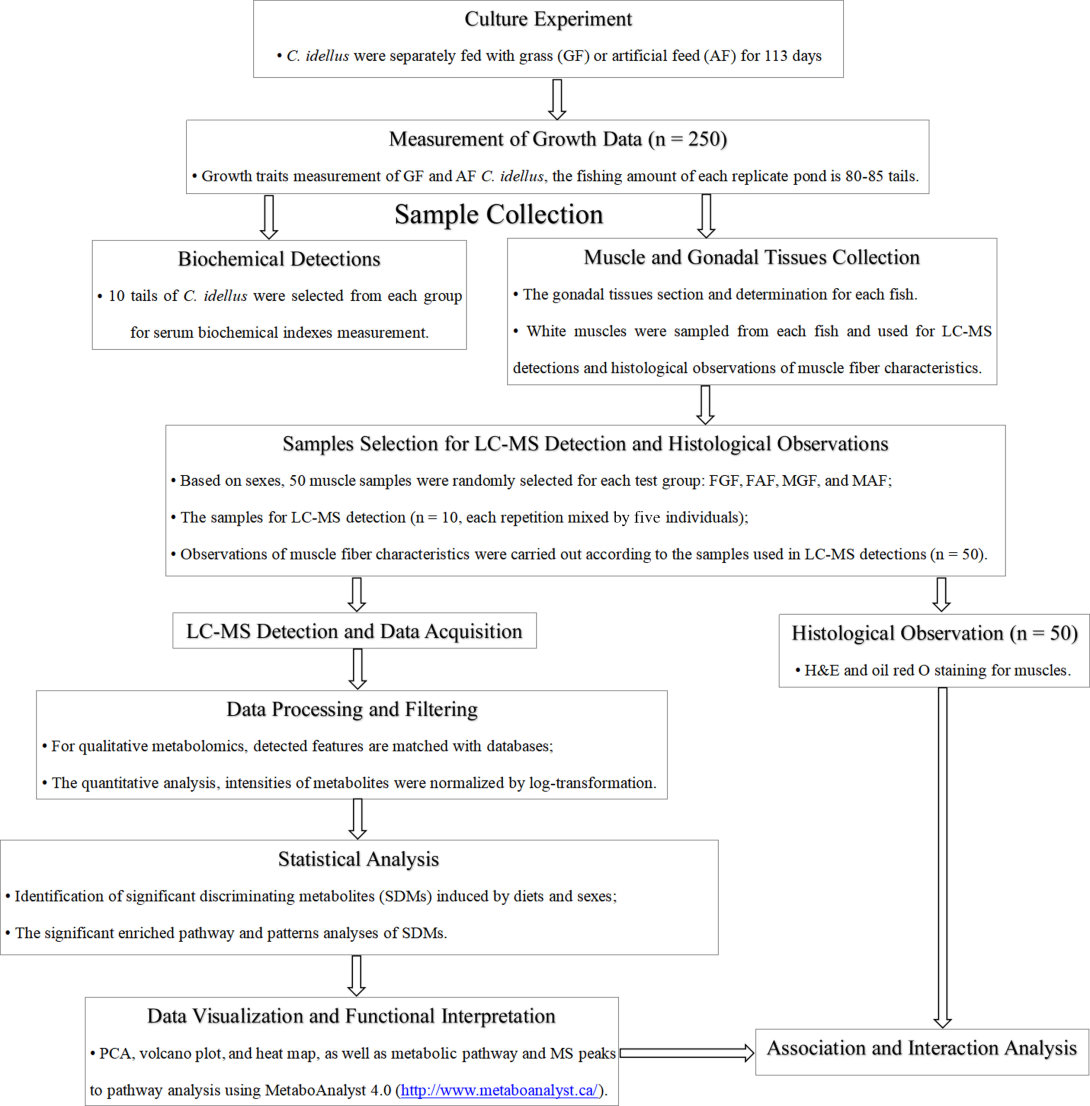
The experimental design and flowchart. H&E: hematoxylin & eosin; PCA: principle component analysis.

All group samples were detected by the BGI (Beijing Genomics Institute, Shenzhen, China) using LC-MS/MS. For qualitative and quantitative metabolomics, raw data were processed using Progenesis QI software (Nonlinear Dynamics, 2017, version: 2.2, Waters, MA, US). To verify and confirm compound identifications, the METLIN batch Metabolite Search , Kyoto Encyclopedia of Genes and Genomes (KEGG), Human Metabolite, and ChemSpider databases were used by comparing molecular weights and .mol files. The molecular and structural formulas of the candidate compounds were retrieved by the comparison and then confirmed by MS/MS scans for the characteristic ions and fragmentation patterns of the metabolites. The statistical analyses of detected features were performed by MetaboAnalyst 4.0 [22], using the Statistical Analysis module [23]. The input data were normalized by a pooled sample (quality control [QC]) from the two experimental groups. The experimental design and analysis flowchart detailing these steps are shown in Fig. 1.

Additionally, we hope these metabolic datasets will contribute to future fish nutrition research, disease and immunization research, optimization of breeding conditions for fish, and even human dietary and nutrition studies. Our raw metabolomics data has been deposited to the European Molecular Biology Laboratory - European Bioinformatics Institute (EMBL-EBI) MetaboLights database with the identifier MTBLS673. The complete dataset can be accessed at [24] and the materials can also be downloaded from GitHub [25]. In addition, the preliminary list of compound identification and information regarding the significant differential metabolites such as potential mapped metabolites, query IDs, *P* values, fold change (FC), false discovery rate (FDR), and corresponding metabolic pathways are provided in the Supplementary Files. The Supplementary files are available via the *GigaScience* repository[26].

## Analyses

### Growth performance

Table 1 shows the growth performances of the four test groups (FAF, FGF, MAF, and MGF). Overall, after 113 days of separate feeding, different diets showed significant effects on different growth traits of the four test groups, regardless of the sex . The body mass, body length, body height, visceral weight, liver weight, and specific growth rate (SGR) of fish in both FAF and MAF were all significantly higher than those in the GF groups (*P* < 0.01). The most pronounced differences due to the artificial diet were increases in weight gain (WG), visceral weight, and liver weight (*P* < 0.05). The final weight of MAF fish was 38.55% higher than that of MGF fish, and the obtained weight of FAF was 11.66% greater than that of FGF. The visceral weights were about 1.5 times higher in AF groups, and the liver weights of AF were about 2-fold greater than that of GF fish. Furthermore, sex is an important factor in fish growth. Compared with MGF *C. idellus*, the WG was 37.44% higher in FGF, whereas in AF groups, the increased weight in female *C. idellus* was 10.73% greater than in male fish. Finally, the condition factor (CF) was the only physical indicator that was significantly higher in GF (*P* < 0.05), despite the sex of the fish:

\begin{equation*}
{{\bf SGR}} = \left( {{\rm{Ln}}\left( {{\rm{W1}}} \right){\rm{ - Ln}}\left( {{\rm{W2}}} \right)} \right)/{\rm{T* }}100
\end{equation*}

\begin{equation*}
{{\bf CF}} = \left( {{\rm{W1}}/{{\rm{L}}^3}} \right)*\,100
\end{equation*}

**Note:** W1- Body Mass (g); W2- Visceral Weight (g); T- Feeding days; L- Body Length (cm)

**Table 1:** Growth data of *C. idellus* fed with different feeds (n = 250)

Gender	Experimental group	Body mass (g)	Body length (cm)	Body height (cm)	Visceral weight (g)	Liver weight (g)	SGR (%)	CF (%)
Female	GF	971.64 ± 5.91 ^a^	31.29 ± 0.56 ^a^	6.46 ± 0.04	38.20 ± 0.31	7.50 ± 0.03 ^a^	2.94 ± 0.01 ^a^	3.19 ± 0.19 *
	AF	1080.80 ± 6.25 ^A^ **	33.81 ± 0.21 **	7.46 ± 0.30 **	60.63 ± 0.78 ^A^ **	14.97 ± 0.07 ^A^ **	3.04 ± 0.00 ^A^ **	2.80 ± 0.04
Male	GF	706.94 ± 10.46	29.02 ± 0.28	6.22 ± 0.07	37.60 ± 0.48	7.01 ± 0.04	2.66 ± 0.02	2.89 ± 0.04 *
	AF	979.50 ± 10.02 **	33.90 ± 0.25 **	7.65 ± 0.11 **	51.71 ± 0.06 **	13.24 ± 0.14 **	2.95 ± 0.01 **	2.51 ± 0.05

**Note:** Measured traits of growth performances are represented as mean ± standard error, compared under the same sex conditions. **, difference between the two experimental groups is significant at the 0.01 level; *, difference is significant at the 0.05 level. The superscripts, lower-case letters mean there is significance between different genders in GF; capital letters indicate significance between female and male in AF.

### Effect of diet on serum biochemical indexes and abdominal fat accumulation

Table 2 shows the serum biochemical data of *C. idellus* in the two experimental groups. The comparisons between GF and AF indicated that different diets resulted in significant differences in concentrations of several serum biochemical indicators (*P* < 0.05). HDLC was the only indicator that showed the least change between the different diets. The majority of the higher concentrations of serum biochemical indicators were found in GF fish, except for albumin (ALB) and TG. The levels of ALB and TG were significant higher in AF (*P* < 0.05).

**Table 2:** Serum biochemical parameters in *C. idellus* farmed under two feeding models

Groups	LD	AST	ALT	ALP	TCHO	HDLC	GLU	ALB	TP	TG
Units	U/L	U/L	U/L	U/L	mmol/L	mmol/L	mmol/L	g/L	g/L	mmol/L
GF	829.17 ± 0.33 **	161.00 ± 0.87 **	237.67 ± 0.44 **	132.83 ± 0.60 **	7.69 ± 0.06 **	2.50 ± 0.20	3.66 ± 0.02 **	3.33 ± 0.17	36.50 ± 0.01 **	6.25 ± 0.00
AF	506.78 ± 0.40	125.78 ± 0.22	163.67 ± 0.51	93.78 ± 0.80	6.27 ± 0.02	2.14 ± 0.05	2.62 ± 0.05	3.89 ± 0.11 *	29.78 ± 0.11	6.71 ± 0.02 **

**Note:** All the serum biochemical parameters were measured and calculated using 10 fish from each experimental group. Measured indexes of serum biochemical are represented as mean ± standard error, compared between the two feeding groups. **, difference between the two experimental groups is significant at the 0.01 level; *, difference is significant at the 0.05 level.

**Figure 2:**
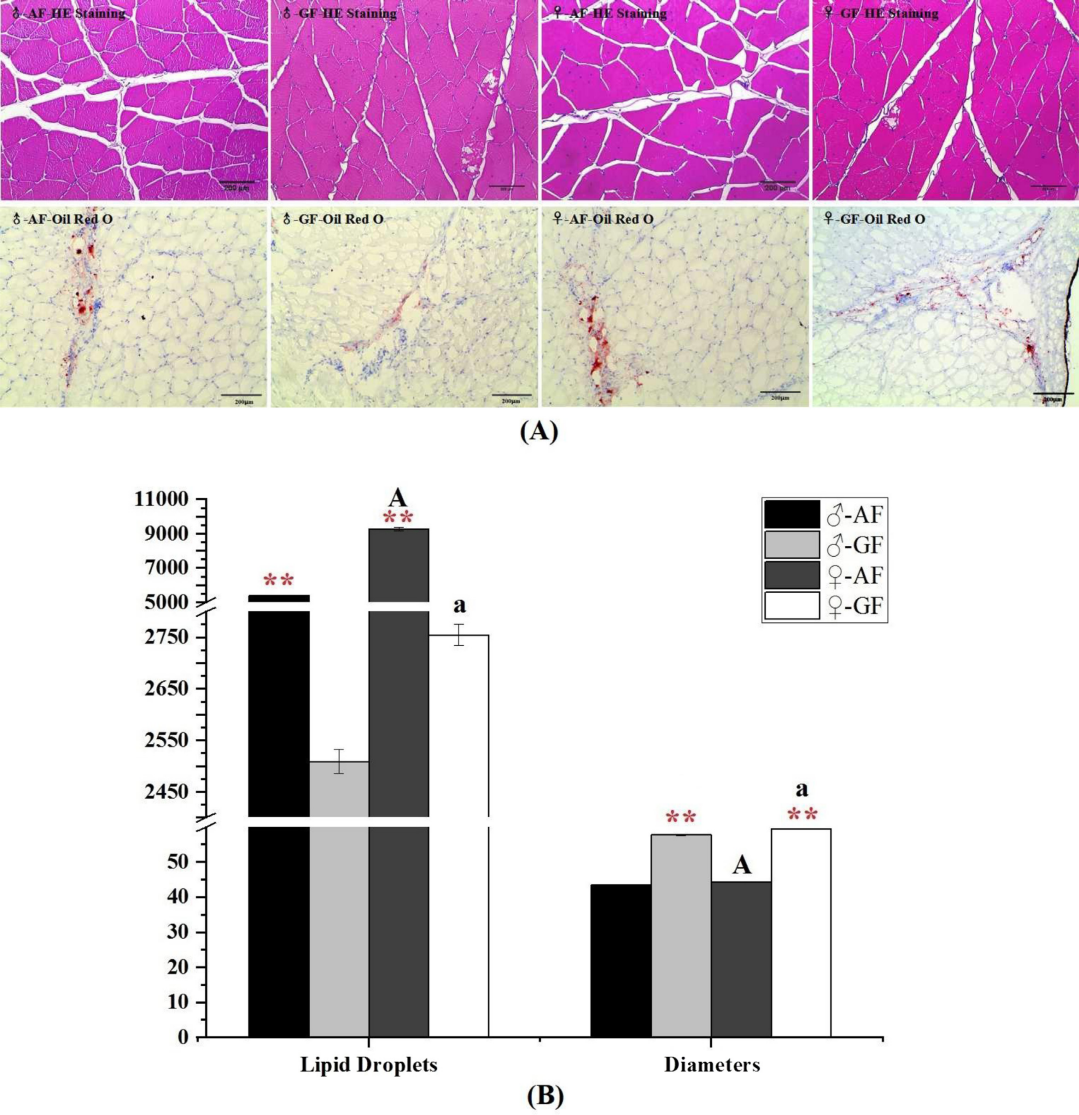
Histological sections of abdominal muscles of *C*. *idellus*. The abdominal muscle samples were collected from AF *C. idellus* and GF *C. idellus*. **(A)** H&E staining (original magnification ×200) shows the characteristics of abdominal muscle fibers, the oil red-O staining sections show the distributions of lipid droplets. **(B)** The statistical observations of muscle tissues sections (n = 50). The four different colors represent the four test groups as follows: black, ♂-AF; gray, ♂-GF; dark gray, ♀-AF; and white, ♀-GF. Vertical bars represent the mean ± standard error. The asterisks (**) indicate the significance between AF and GF under the same sex conditions. The letter “A” was used to represent the significance between two sexes of *C. idellus* fed with artificial feed. In addition, the letter “a” represents the significant difference between ♂-GF and ♀-GF.

The changes in muscle fibers and intramuscular lipid droplet sizes in abdominal muscles were observed using hematoxylin-eosin (HE) and oil red O staining, respectively (Fig. 2A). The corresponding statistics are calculated and visualized in Fig. 2B. Compared with GF groups, the mass of lipid droplets was significantly increased in both MAF and FAF *C. idellus* (*P* < 0.01), which is largely attributed to elevated numbers of adipocytes in these two test groups and not an enlargement of the size of adipocytes. Moreover, the average diameter of muscle fibers was significantly higher in GF groups (*P* < 0.01). Between the two sexes, the size of lipid droplets and the diameter of abdominal muscle fibers were both significantly higher in female fish, specifically FAF and FGF *C. idellus* (*P* < 0.01).

### Effect of diets on metabolomic alterations of muscle samples

**Figure 3:**
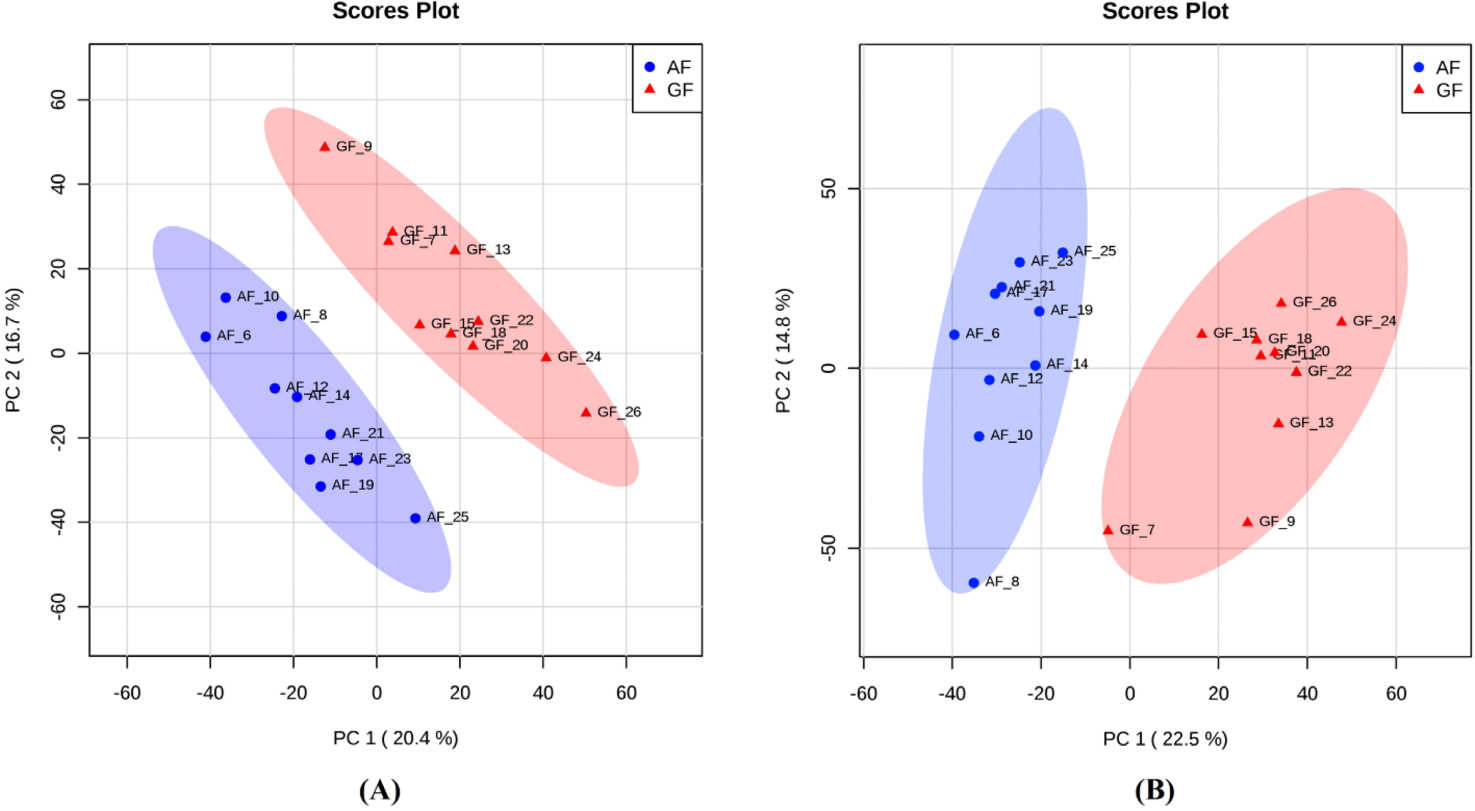
PCA score plots for the metabolomics profiles of *C. idellus* muscle samples (n = 10). PCA score plots for the metabolomics profiles of muscle samples from female **(A)** and male **(B)***C. idellus*. AF group, blue points; GF group, red triangles.

Muscle samples were collected after a 113-day breeding period and subjected to untargeted LC-MS metabolomics analysis. The two score plots of the principle component analysis (PCA) models show a clear separation of samples from different experimental groups and quality controls (Fig. 3), indicating that feeding *C. idellus* with artificial feed or natural grass could induce significant changes in the muscle metabolomic profile in both sexes of *C. idellus*, with male samples showing a slightly more clear separation between the two different diet groups. The corresponding loading plots for PCA models are provided in Supplementary Fig. S1.

### Identification of discriminating features between groups

**Figure 4:**
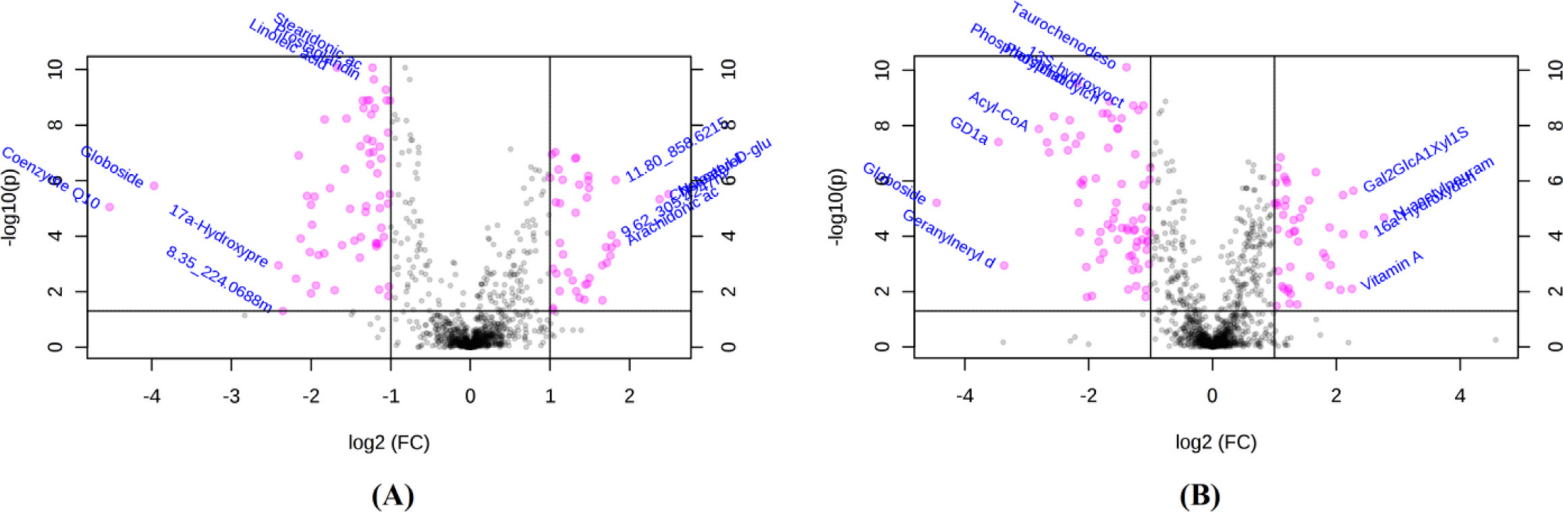
Volcano plots for the potential metabolomic features of muscle samples from female **(A)** and male **(B)***C. idellus* (n = 10). Pink points indicate significant metabolites between the two groups (FC <0.5 or >2.0; *q*value <0.05). The gray points show tentatively matched features with no significance. The potential biomarkers between experimental groups were annotated with their matched metabolite names; those nonannotated peaks were marked with their corresponding mass weights and retention time.

The significant discriminating metabolites (SDMs) were identified based on the following criteria: FC threshold ≥2 (or <0.5) and an FDR-adjusted *P* value (*q*value) <0.05 using the volcano plot analysis (Fig. 4). Based on the criteria, 41 metabolites were significantly upregulated in FAF and 63 metabolites were significantly downregulated in the same group (Fig. 4A). In MAF *C. idellus*, 45 metabolites were upregulated and 75 metabolites were downregulated (Fig. 4B). All SDMs between the two experimental groups are respectively summarized in Supplementary Tables S1 and S2 along with their matched adducts, potential metabolites, query IDs, *P* values, FC, FDR, and corresponding metabolic pathways.

Additionally, the peak intensities of all the SDMs were normalized and log transformed before the Pearson correlation method was used to identify correlations between SDMs that differed between AF groups and GF groups (Supplementary Fig. S2).

### The impacts of different diets on *C. idellus* muscle metabolism

**Figure 5:**
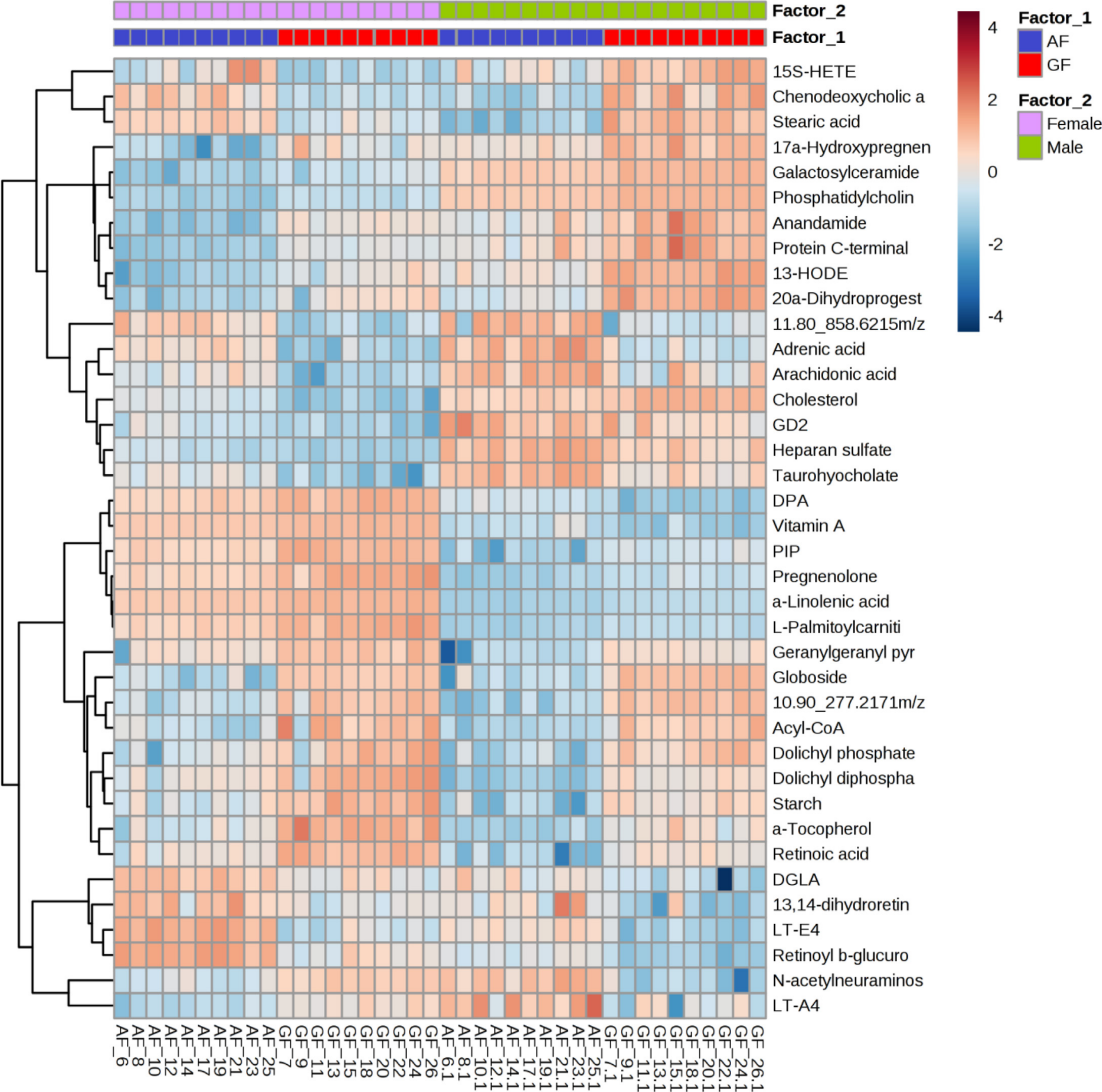
The differential and overlapped metabolites between the four test groups. Heat map visualization of metabolomic data shows the relative intensities of significant features; those are not only annotated by existing metabolites database and also overlapped among the four test groups (FAF, FGF, MAF, and MGF). Each row was labeled with the tentative metabolite names. The colors refer to the relative levels of these compounds from high (red) to low (blue).

The relative peak intensities of 39 SDMs shared between female and male metabolic profiles are visualized as a heat map in Fig. 5. The correlation analysis of the SDMs (including overlapped and sex-specific SDMs) specifically related to lipid and carbohydrate metabolisms are shown in the Supplementary Files (Supplementary Fig. S2).

Compared with FGF *C. idellus*, the relative intensity of stearic acid (a saturated FA) was significantly higher in FAF (FC >2.0, *q*value close to zero). A number of unsaturated FAs (UFAs) discriminated between the FGF and FAF samples. In particular, docosapentaenoic acid (DPA), adrenic acid, dihomo-gamma-linolenic acid (DGLA), arachidonic acid (ARA) and Leukotriene E4 (LTE4) all showed significantly higher concentrations in the FAF *C. idellus* (*q*value <0.05), as did 10 ARA metabolites with a similar structure: 15(S)-hydroxy-eicosatetraenoic acid (15S-HETE), 5-HETE, 8-HETE, 9(S)-HETE, 16(R)-HETE, 19(S)-HETE, 8,9-epoxyeicosatrienoic acid, 11,12-epoxyeicosatrienoic acid, 5,6-epoxy-8,11,14-eicosatrienoic acid, and 14,15-epoxy-5,8,11-eicosatrienoic acid. These metabolites were significantly higher in the FAF and were all positively correlated with each other (Supplementary Fig. S2A). In female grass-fed *C. idellus*, metabolites involved in lipid metabolism exhibited significantly higher levels (FC <0.5, *q*value <0.05), such as diacylglycerol, L-palmitoylcarnitine, LTA4, DHA, palmitic acid, prostaglandin G2 (PGG2), EPA, linoleic acid, 13S-hydroxyoctadecadienoic acid, gamma-linolenic acid (GLA), stearidonic acid, and caprylic acid. These significantly upregulated metabolites in FGF showed positive correlations with each other and negative correlations with those upregulated metabolites in FAF (Supplementary Fig. S2A).

Compared with the results of female *C. idellus*, more SDMs were more highly upregulated in MGF fish. Particularly, among the 75 significantly upregulated metabolites in MGF, 26 were related to lipid metabolism. Further, the differential FAs, such as pelargonic acid, stearic acid, and L-palmitoylcarnitine, displayed significantly higher intensities in MGF (FC <0.5, *q*value <0.05). Additionally, the remaining eight discriminatory metabolites between MAF and MGF were involved in UFAs metabolism, including viz, arachidic acid, EPA, Leukotriene B4 (LTB4), 13(S)-hydroxyoctadecadienoic acid, 15(S)-HETE, 5-HETE, 13(S)-HPOT, alpha-linolenic acid (ALA), and GLA, which all had significantly higher peak intensities in MGF (FC <0.5, *q*value < 0.05). All of the upregulated metabolites in MGF *C. idellus* were negatively correlated with those upregulated metabolites in MAF (namely, adrenic acid, DHA, DPA, ARA, and DGLA, as well as three leukotrienes [LTs]) (FC >2.0, *q*value <0.05) (Supplementary Fig. S2B).

Feeding *C. idellus* with different diets also affects carbohydrate metabolism. FGF significantly increased the intensity of metabolites related to glycometabolism (mannan, globoside, uridine diphosphate galactose [UDP]-glucose, UDP-galactose, starch, Tn-antigen, and protein C-terminal S-farnesyl-L-cysteine methyl ester) (*q*value <0.05). A number of physiologically important metabolites are very different between FGF and MAF groups, including geranyl pyrophosphate, dolichyl diphosphate, dolichyl phosphate, 9-cis-retinoic acid, diacylglycerol, 5-L-glutamyl-L-alanine, alpha-tocopherol, and phosphatidylinositol triphosphate (PIP) 2. These metabolites were all upregulated in FGF muscle samples (FC <0.5). The downregulated metabolites in FGF were heparan sulfate, 3-phosphatidyl-ethanolamine, uridine diphosphate-glucuronic acid (UPD-glucuronic acid), ganglioside G D2 (GD2), 1-acylglycerophosphoinositol, flavin mononucleotide, and sialyl-Tn antigen (FC >2, *q*value <0.05). They showed significant positive correlations with each other and negative correlations with metabolites upregulated in FGF (Supplementary Fig. S2A).

In male *C. idellus* samples, alpha-tocopherol, globoside, starch, protein C-terminal S-farnesyl-L-cysteine methyl ester, 5-L-glutamyl-L-alanine, 9-cis-retinoic acid, dolichyl diphosphate, and dolichyl phosphate showed significantly higher levels in MGF compared to MAF. In addition, GD1a, L-amino acid, PIP3, dihydroxyacetone phosphate, trypanothione disulfide, and UDP-N-acetyl-D-glucosamine also displayed higher intensities in the MGF muscles. Downregulated metabolites in MGF were quite different from the SDMs identified in FGF. In addition to GD2 and heparan sulfate, UDP-D-xylose, GD1b, 1-phosphatidyl-D-myo-inositol, D-glucosaminide, inositol phosphate, and trypanothione also exhibited lower intensities in MGF samples. (Gal)2(GlcA)1(Xyl)1(Ser)1 and naphthyl-2-oxomethyl-succinyl-CoA also showed lower intensities in the MGF samples.

### Pathway enrichment analysis

**Figure 6:**
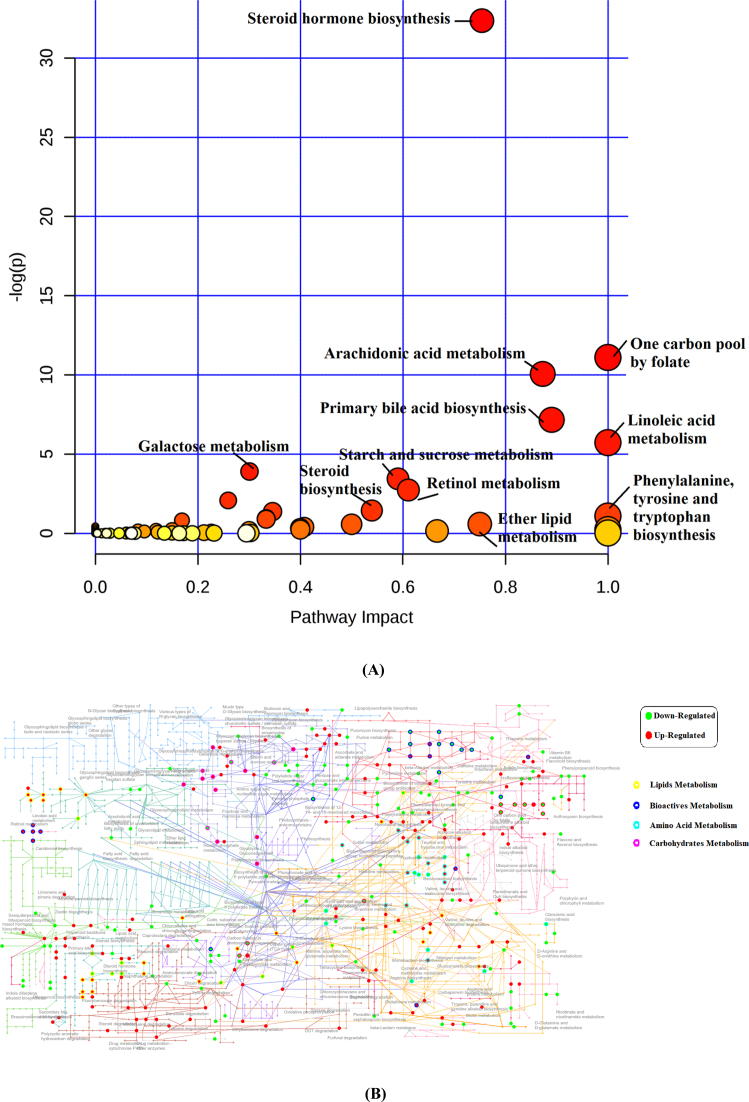
The pathway enrichment and network analyses for the significant metabolites in female *C. idellus*. **(A)** The scatter plot was used to visualize the pathway impact and enrichment results for all matching significant metabolites in female *C. idellus*. **(B)** The KEGG global metabolic network visualization of all significant metabolites (*P* < 0.05) in the female *C. idellus* metabolic profile. The colored points represent different metabolic pathways. The various color levels indicate different levels of significance of metabolic pathways from low (white) to high (red). The different sizes of each point are used to represent the number of metabolites that participated in the metabolic pathway. The greater the rich factor, the greater the degree of pathway enrichment. Moreover, the corresponding pathway's name of each point is labeled. In the metabolic network, all upregulated metabolites (FC AF/GF >2) in AF groups are colored red, whereas the downregulated metabolites (FC <0.5) are colored green. In addition, the different color circles represent the various physiological functions that the discriminating metabolites belong to. Moreover, each enriched pathway is annotated with the corresponding name.

Using *Danio rerio* as the reference library, the pathway impact and enrichment analysis of the significantly different metabolites, as well as the network and the physiological properties of the matched compounds of female and male *C. idellus* samples, are separately shown in Fig. 6 (female *C. idellus* metabolic profile) and Supplementary Fig. S3 (male *C. idellus* metabolic profile). Regardless of the sex of *C. idellus*, most of the discriminating metabolites between AF and GF groups were largely concentrated in FA and UFA metabolism, steroid hormone metabolism, vitamin metabolism, and amino acids metabolism, as well as glycometabolism pathways.

The pathway impact and enrichment analysis of the significantly different metabolites (*P* < 0.05) in both female and male *C. idellus* samples were also conducted using MetaboAnalyst 4.0, Enrichment Analysis and Pathway Analysis modules. Generally, most of the enriched pathways were the same for female and male *C. idellus*. The differential metabolites were significantly enriched in steroid hormone biosynthesis (as above), carbon pool by folate, arachidonic acid, and lenoleic acid metabolisms, as well as primary bile acid biosynthesis pathways, in both female and male metabolic profiles (*P* < 0.05) (Fig. 6A and Supplementary Fig. S3A). These significantly altered pathways also had high impact values (>0.75). However, the differences between the two genders were starch and sucrose metabolism, as well as galactose metabolism, which showed significant enrichment in female *C. idellus* but not in male *C. idellus*. (*P* < 0.05) (Fig. 6A and Supplementary Fig. S3A). The significantly different metabolites in male *C. idellus* were also significantly enriched in retinol metabolism and steroid biosynthesis pathways (*P* < 0.05), which showed no significance in female results.

All significant metabolites appeared in both female and male experimental groups; their corresponding metabolic pathways are listed in Table 3 (female) and Table 4 (male). In addition, they are all highlighted in red and annotated with KEGG IDs in KEGG global metabolic map of the results of the pathway enrichment analysis (Supplementary Fig. S4). In addition to the biomarkers between AF and GF groups, many sex-specific metabolites were found between genders, such as estriol (C05141), 2-hydroxyestradiol (C05301), estradiol (C00951), 4-hydroxyretinoic acid (C16677), 5,6-epoxyretinoic acid (C16680), and 11-cis-retinyl palmitate (C03455). However, all significant metabolites enriched in one carbon pool by folate, primary bile acid metabolism, arachidonic acid, and linoleic acid metabolisms pathways were similar in both female and male *C. idellus* metabolic profiles.

**Table 3:** Pathway impact and overlapped metabolite analysis of female *C. idellus*

Pathway name	Hits	Raw p	FDR	Impact	Overlapping metabolites in pathways
Steroid hormone biosynthesis	44/56	0.0000	0.0000	0.7542	Cholesterol (C00187), Androstenedione (C00280), Progesterone (C00410), Estrone (C00468), Androsterone (C00523), Cortisone (C00762), 17-Hydroxyprogesterone (C01176), DHEA (C01227), Pregnenolone (C01953), Corticosterone (C02140), Deoxycorticosterone (C03205), DHT (C03917), 5-Androstenediol (C04295), Etiocholanolone (C04373), 17α-Hydroxypregnenolone (C05138), Adrenosterone (C05285), 16α-Hydroxy-DHEA (C05139), 11-DHC (C05490), 16α-Hydroxyandrost-4-ene-3,17-dione (C05140), 11β-Hydroxyandrost-4-ene-3,17-dione (C05284), Estriol (C05141), 19-Hydroxyandrost-4-ene-3,17-dione (C05290), 19-Hydroxytestosterone (C05294), 2-Hydroxyestrone (C05298), 2-Methoxyestrone (C05299), 2-Hydroxyestradiol (C05301), Testosterone glucuronide (C11134), 7-Hydroxy-DHEA (C18045), 21-Hydroxypregnenolone (C05485), Tetrahydrocorticosterone (C05476), 20α-Hydroxycholesterol (C05500), 3α,21-Dihydroxy-5b-pregnane-11,20-dione (C05478), 17a,21-Dihydroxypreg-nenolone (C05487), Cortexolone (C05488), 11β,17α,21-Trihydroxypreg-nenolone (C05489), 20α,22β-Dihydroxycholesterol (C05501), 22β-Hydroxycholesterol (C05502), 17β-Estradiol-3-glucuronide (C05503), 2-Methoxy-estradiol-17β 3-glucuronide (C11131), 2-Methoxyestrone 3-glucuronide (C11132), Estrone glucuronide (C11133), Androsterone glucuronide (C11135), Etiocholanolone glucuronide (C11136), 11β,17β-Dihydroxy-4-androsten-3-one (C18075)
One carbon pool by folate	9/9	0.0000	0.0006	1.0000	THF (C00101), 5,10-Methylene-THF (C00143), 10-CHO-THF (C00234), DHF (C00415), 5-MTHF (C00440), 5,10-CH = THF (C00445), Folic acid (C00504), 5-Formimino-THF (C00664), N5-Formyl-THF (C03479)
Arachidonic acid metabolism	20/31	0.0000	0.0012	0.8728	ARA (C00219), PGD2 (C00696), LTA4 (C00909), PGI2 (C01312), LTC4 (C02166), 15(S)-HETE (C04742), 5-HETE (C04805), 5-HPETE (C05356), LTD4 (C05951), PGG2 (C05956), 15(S)-HPETE (C05966), 19(S)-HETE (C14749), 5,6-Epoxy-DGLA (C14768), 8,9-EET (C14769), 11,12-EET (C14770), 11H-14,15-EETA (C14813), 14,15-EET (C14771), 15H-11,12-EETA (C14781), 11,12,15-THETA (C14782), 11,14,15-THETA (C14814)
Primary bile acid biosynthesis	20/36	0.0008	0.0157	0.8903	Cholesterol (C00187), 3α,7α,12α-Trihydroxy-5β-cholestan-26-al (C01301), 7α-Hydroxycholesterol (C03594), 3α,7α-Dihydroxy-5β-cholestanate (C04554), 3α,7α,12α-Trihydroxy-5β-cholestanoic acid (C04722), 3α,7α,26-Trihydroxy-5β-cholestane (C05444), 3α,7α-Dihydroxy-5β-cholestan-26-al (C05445), 27-Deoxy-5β-cyprinol (C05446), 3α,7α-Dihydroxy-5β-cholestane (C05452), 5β-Cholestane-3α,7α,12α-triol (C05454), 12,13-EpOME (C14826), 7α-Hydroxy-cholestene-3-one (C05455), 7α,27-Dihydroxycholesterol (C06341), 24-Hydroxycholesterol (C13550), (24S)-7α,24-Dihydroxycholesterol (C15518), 25-Hydroxycholesterol (C15519), Cholest-5-ene-3b,26-diol (C15610), 3β-Hydroxy-5-cholestenoate (C17333), 7α,26-Dihydroxy-4-cholesten-3-one (C17336), 13(S)-HPODE (C04717), 7α-Hydroxy-3-oxo-4-cholestenoate (C17337), 4-Cholesten-7α,12α-diol-3-one (C17339)
Linoleic acid metabolism	6/7	0.0033	0.0530	1.0000	Linoleic acid (C01595), 13-HODE (C14762), 13-OxoODE (C14765), 9,10-Epoxyoctadecenoic acid (C14825)
Galactose metabolism	13/26	0.0202	0.2732	0.3008	D-Glucose (C00031), UDP-glucose (C00029), UDP-galactose (C00052), Sucrose (C00089), α-Lactose (C00243), α-D-Glucose (C00267), Raffinose (C00492), Sorbitol (C00794), Melibitol (C05399), Epimelibiose (C05400), Galactosylglycerol (C05401), Melibiose (C05402), D-Gal α 1→6D-Gal α 1→6D-Glucose (C05404)
Starch and sucrose metabolism	11/12	0.0320	0.3707	0.5905	Starch (C00369), Sucrose (C00089), α-D-Glucose (C00267), D-Glucose (C00031), UDP-glucose (C00029), Dextrin (C00721), UDP-glucuronic acid (C00167), 1β-D-Glucopyranosyl-4-D-glucopyranose (C00185), D-Maltose (C00208), β-D-Glucose (C00221), 1,4β-D-Glucan (C00760)
Retinol metabolism	8/16	0.0651	0.6589	0.6108	Retinal (C00376), Vitamin A (C00473), 11-cis-Retinol (C00899), All-trans-13,14-dihydroretinol (C15492), Retinoyl β-glucuronide (C11061), 9-cis-Retinoic acid (C15493), 9-cis-Retinal (C16681), 9-cis-Retinol (C16682)

**Table 4:** Pathway impact and overlapped metabolite analysis of male *C. idellus*

Pathway name	Hits	Raw p	FDR	Impact	Overlapping metabolites in pathways
Steroid hormone biosynthesis	43/56	0.0000	0.0000	0.7648	Cholesterol (C00187), Androstenedione (C00280), P4 (C00410), Estrone (C00468), Androsterone (C00523), Cortisone (C00762), 17-OHPG (C01176), Etiocholanolone (C04373), DHEA (C01227), Pregnenolone (C01953), Corticosterone (C02140), DOC (C03205), DHT (C03917), 5-Androstenediol (C04295), 7α-OH-DHEA (C18045), 16α-OH-DHEA (C05139), 17α-Hydroxypregnenolone (C05138), 16α-Hydroxyandrost-4-ene-3,17-dione (C05140), Estradiol (C00951), Cortexolone (C05488), 11β-Hydroxyandrost-4-ene-3,17-dione (C05284), Estrone glucuronide (C11133), Adrenosterone (C05285), 19-Hydroxyandrost-4-ene-3,17-dione (C05290), 11-DHC (C05490), 19-Hydroxytestosterone (C05294), 2-Hydroxyestrone (C05298), 2-Methoxyestrone (C05299), Androsterone glucuronide (C11135), THB (C05476), 3α,21-Dihydroxy-5β-pregnane-11,20-dione (C05478), 20α-Hydroxycholesterol (C05500), 21-Hydroxypregnenolone (C05485), Testosterone glucuronide (C11134), 17α,21-Dihydroxypreg-nenolone (C05487), 11β,17α,21-Trihydroxypreg-nenolone (C05489), 20α,22β-Dihydroxycholesterol (C05501), 22R-Hydroxycholesterol (C05502), 17β-Estradiol-3-glucuronide (C05503), 2-Methoxy-estradiol-17β 3-glucuronide (C11131), 2-Methoxyestrone 3-glucuronide (C11132), Etiocholanolone glucuronide (C11136), 11β,17β-Dihydroxy-4-androsten-3-one (C18075)
Arachidonic acid metabolism	20/31	0.0003	0.0133	0.8728	ARA (C00219), PGD2 (C00696), LTA4 (C00909), PGI2 (C01312), LTC4 (C02166), LTD4 (C05951), PGG2 (C05956), 15(S)-HETE (C04742), 5-HETE (C04805), 5-HPETE (C05356), 15(S)-HPETE (C05966), 19(S)-HETE (C14749), 5,6-Epoxy-DGLA (C14768), 8,9-EET (C14769), 11,12-EET (C14770), 11H-14,15-EETA (C14813), 14,15-EET (C14771), 15H-11,12-EETA (C14781), 11,12,15-THETA (C14782), 11,14,15-THETA (C14814)
One carbon pool by folate	8/9	0.0009	0.0254	1.0000	THF (C00101), 5,10-Methylene-THF (C00143), 10-CHO-THF (C00234), DHF (C00415), 5-Methyl-THF (C00440), 5,10-Methenyl-THF (C00445), 5-Formimino-THF (C00664), N5-Formyl-THF (C03479)
Retinol metabolism	11/16	0.0039	0.0740	0.6108	Retinal (C00376), Vitamin A (C00473), 11-cis-Retinol (C00899), Retinoyl β-glucuronide (C11061), 9-cis-Retinoic acid (C15493), 9-cis-Retinal (C16681), 9-cis-Retinol (C16682), All-trans-13,14-dihydroretinol (C15492), 4-Hydroxyretinoic acid (C16677), all-trans-5,6-Epoxyretinoic acid (C16680), 11-cis-Retinyl palmitate (C03455)
Primary bile acid biosynthesis	20/36	0.0046	0.0740	0.8903	Cholesterol (C00187), 3α,7α,12α-Trihydroxy-5β-cholestan-26-al (C01301), 7α-Hydroxycholesterol (C03594), 3α,7α-Dihydroxy-5β-cholestanate (C04554), 7α,26-Dihydroxy-4-cholesten-3-one (C17336), 3α,7α,12α-Trihydroxy-5β-cholestanoic acid (C04722), 3α,7α,26-Trihydroxy-5β-cholestane (C05444), 3α,7α-Dihydroxy-5β-cholestan-26-al (C05445), 27-Deoxy-5β-cyprinol (C05446), 324-Hydroxycholesterol (C13550), α,7α-Dihydroxy-5β-cholestane (C05452), 5β-Cholestane-3α,7α,12α-triol (C05454), 7α-Hydroxy-cholestene-3-one (C05455), 7α,27-Dihydroxycholesterol (C06341), 25-Hydroxycholesterol (C15519), 3β-Hydroxy-5-cholestenoate (C17333), (24S)-7α,24-Dihydroxycholesterol (C15518), Cholest-5-ene-3β,26-diol (C15610), 7α-Hydroxy-3-oxo-4-cholestenoate (C17337), 4-Cholesten-7α,12a-diol-3-one (C17339)
Linoleic acid metabolism	6/7	0.0068	0.0916	1.0000	Linoleic acid (C01595), 13(S)-HPODE (C04717), 13-HODE (C14762), 13-OxoODE (C14765), 9,10-Epoxyoctadecenoic acid (C14825), 12,13-EpOME (C14826)
Steroid biosynthesis	18/33	0.0091	0.1055	0.6930	Cholesterol (C00187), Squalene (C00751), (S)-2,3-Epoxysqualene (C01054), 7-DHC (C01164), Lathosterol (C01189), Lanosterin (C01724), Desmosterol (C01802), 5α-Cholest-8-en-3β-ol (C03845), 7-Dehydrodesmosterol (C05107), 4,4-Dimethyl-5α-cholesta-8,24-dien-3β-ol (C05108), 24,25-Dihydrolanosterol (C05109), Zymosterol intermediate 2 (C05437), 5α-Cholesta-7,24-dien-3β-ol (C05439), Avenasterol (C08821), 5-Dehydroepisterol (C15780), Delta-7-Avenasterol (C15782), 5-Dehydroavenasterol (C15783), 4,4-Dimethyl-5α-cholesta-8-en-3b-ol (C15915)

**Note:** The significantly enriched pathways were list in the table from lowest FDR *P* value to highest FDR *P* value < 0.05, with their corresponding overlapped metabolites in the pathway. Despite the metabolites' names, the mapping features were also annotated with KEGG component IDs.

To verify the pathway analysis result based on manual annotation, we also used the new MS Peaks to Pathways (Mummichog) module in MetaboAnalyst 4.0 to predict pathway activities (*D. rerio* pathway library) directly from the 1,033 significantly differential peaks detected in the female *C. idellus* samples and 1,495 peaks in the male samples (*q*value <0.05) (data not shown). Five significant enriched pathways were identified in the female *C. idellus* metabolomic profiles, including steroid hormone biosynthesis, arachidonic acid metabolism, retinol metabolism, steroid biosynthesis, and biosynthesis of UFAs (*q*value <0.05). On the other hand, six pathways were significantly enriched by male *C. idellus*'s 1,495 mapped and significant differential features, such as steroid hormone biosynthesis, steroid biosynthesis, arachiconic acid metabolism, and retinol metabolism, as well as glycerophospholipid metabolism and sphingolipid metabolism (*q*value <0.05). All the enriched metabolic pathways in male and female profiles are responsible for lipid and vitamin metabolism.

## Discussion

### Differences in growth performance between groups

In this study, we found significantly different growth performance between the AF and natural GF groups. This observation is consistent with the previous study, whereby feeding *C. idellus* with natural grass resulted in significantly lower WG in both FGF and MGF groups [21, 27]. The negative correlation between enhanced dietary fibers and low-fat diet with reduced WG was also demonstrated in *Barbodes altus* and *Oncorhynchus mykiss* [28, 29]. Although different diets changed the growth traits of *C. idellus*, the dietary effect on fish growth seemed to be sex independent. For instance, significantly lower WG was observed in male fish. This is contrary to the result in a study on mice fed a low-fat diet, in which females showed less WG [30]. Further research is warranted to investigate whether different diets have sex-dependent effects on growth traits in different species.

### Fat deposition induced by AF feeding and improvement of feeding grass

The significant increase in fat mass, as detected in the abdominal muscles of both FAF and MAF groups, was caused by an increased number of adipocyte cells, not an increase in the size of adipocytes. This is in agreement with the conclusion of a previously published *Oreochromis niloticus* study [31]. Proliferation of adipocytes is likely the main strategy of fish responding to the intake of high-fat and high-protein diets. Significantly smaller diameters of muscle fiber were found in AF muscle samples that, together with increased fat contents, would influence the texture and taste of the fish flesh [32]. A common misconception is that high-protein diets inevitably raise cholesterol levels in serum. In contrast, this study found that a significantly higher concentration of cholesterol was detected in the GF group compared to the AF group. The moderate and good cholesterol could give structure to cell walls and produce certain hormones [33]. However, higher levels of TG were found in the *C. idellus* fed an artificial diet. All the findings described above demonstrate that a sustained high-fat and high-protein diet had clear deleterious effects on the fish, characterized by an increased serum TG, fat accumulation in organs (such as viscera and muscle), and decreased stress tolerance [20].

### Changes in lipid metabolism in fish flesh induced by different diets

It is well known that chronic consumption of high dietary fat and protein can disrupt lipid homeostasis, thereby leading to steatosis and fat deposition [34, 35]. Few studies, however, have focused on linking physiological measures to functions at the metabolic level. One study has described the effect of low- and high-fat diets on the metabolism in *O. niloticus* [27], but the influence of different diets on muscle characteristics and lipid metabolism, as well as correlations between them, has only been investigated in mammals [12, 18, 36]. Nevertheless, the assessment of dietary intake in these studies is subjective as there are no definite standards for measuring the nutritional status of a diet nor the experimental subject [37]. On the other hand, ingestion of different diets at various doses can have highly divergent effects [28, 38]; different tissues can have very different metabolic patterns [39, 40]. Although metabolomic studies of the effect of diets in different animal studies may not be directly comparable, they can serve as references for further research. In our study, a large amount of metabolites were detected and differentially enriched in energy metabolism between the two feeding groups, indicating that metabolic alteration is the main mechanism by which fish respond to different feeding patterns. Furthermore, the potential associations between physiological changes (the muscle characteristics, together with nutrients and textures measured in our previously published studies) [21, 27] and metabolic profiles in different feeding conditions are systematically examined below.

In general, flesh quality of cultured animals is primarily attributed to nutritional condition in diets. Higher nutritional levels in diets could result in increased levels of saturated fatty acids (SFA) and decreased levels of PUFAs [41]. In the present study, the proportions of SFA in the GF groups’ muscle samples were markedly higher, while PUFA levels were almost equal to their levels in muscle samples of the AF groups (including both FAF and MAF). The same findings have been proven in our previously published study [27]. Nevertheless, this conclusion is not absolute; different animals can give rise to distinct results. For instance, the opposite result was obtained in a lamb study, with increased PUFA and decreased SFA contents found in the natural diet group rather than the artificial diet group [36]. In addition to species diversity, different sources of plant protein between the two studies may have also contributed to contrasting results.

As reported in other research, SFAs (e.g., C16:0 and C18:0) play important roles in influencing flesh texture, with higher levels resulting in a “crisper” flesh taste [27, 42]. In line with the previous findings, the increased arachidic acid (20:0), stearic acid (18:0), and palmitic acid (16:0) were detected in our GF groups; therefore, the flesh of GF *C. idellus* would be harder than that of AF fish. This conclusion is consistent and proven by our previously published test results of textures, shear force detection, and fatty acid content measurements of the two feeding groups' *C. idellus* flesh [21, 27]. Previous research has also shown that higher arachidic acid can interfere with essential fatty acid metabolism by inhibiting the Δ-6 desaturase enzyme, which reduces the formations of DGLA (20:3n-6) and ARA (20:4n-6) [43]. Thus, significantly lower levels of DGLA and ARA were detected in FGF and MGF. For another SFA, palmitic acid (16:0), its significantly higher levels were also found in GF fish. This is consistent with previous research, where it was higher in the GF *C. idellus*, which in turn were thinner than the other feeding group fish [44]. Similiar to our study, the fat mass of muscle tissues of grass feeding *C. idellus* was significant lower than that of FAF and MAF muscle tissues. Additionally, GF increases the levels of several medium-chain fatty acids (e.g., caprylic acid and pelargonic acid), which are known to contribute to better flesh flavor and odor, help reduce abdominal fat, and improve cholesterol levels [33].

Several n-3 PUFAs, such as EPA (20:5n-3), DHA (22:6n-3), and their important intermediaries, as well as ALA (18:3n-3) and stearidonic acid (18:4n-3), were significantly different between grass-fed and artificial-fed *C. idellus* muscle samples. They are all the most bioactive of n-3 PUFAs and are known to be beneficial for human health [11]. The significantly higher levels of EPA were found in MGF and FGF *C. idellus*, which could be derived from higher levels of ALA in the same groups [45] or could originate from grass rich in n-3 PUFAs. Although the same finding has not been demonstrated in other fish studies, a similar conclusion was obtained in a cow study, where significantly higher levels of n-3 PUFAs were found in grass-fed cows [13]. Because of the important physiological significance of higher EPA to mammals [11, 46], the accumulation of EPA in organisms is a hot research topic and could be facilitated by the reduced mitochondrial FAs β-oxidation [17, 20]. DHA and DPA were markedly upregulated in AF groups, though are known to be rich components in animal artificial feeds [47], so their higher levels may be directly taken from artificial feeds. On the other hand, higher DPA in FAF and MAF muscles could be attributed to aggregate data with different types of DPA (n-3/n-6). Overall, the higher DPA and DHA contents in *C. idellus* fed artificial feed are consistent with the previous statement; they indirectly reflect the corresponding higher contents in the artificial feed used in our study. However, the main fatty acid component in green plants is generally ALA, meanwhile they have a much higher proportion of n-3 PUFAs compared to n-6 PUFAs [38]. Consequently, feeding fish with natural grass caused remarkably higher ALA levels in FGF and MGF than AF *C. idellus*. Another reasonable explanation for the higher ALA in GF groups is that ALA is a substrate for endogenous formation of EPA [46]; therefore, the higher ALA positively correlating with the higher observations of EPA in the same test groups makes sense. In summary, *C. idellus* fed grass is comparable to their wild counterparts, characterized by higher ALA and EPA and lower DHA levels in their muscle tissues [16]. Grass-fed farmed *C. idellus* would be more attractive to consumers for the reason that intake of EPA has been recommended as a promising novel therapy to decrease hepatic triglyceride content [20].

Another noteworthy group of significantly discriminating metabolites between the two feeding groups is the n-6 PUFA family. Contrary to the higher proportions of DGLA and ARA in both FAF and MAF, the levels of GLA (18:3n-6) and LA (18:2n-6) were significantly higher in FGF fish and were only altered in female samples. Their relative intensities in the metabolic profiles of *C. idellus* muscle tissues were sex specific. An earlier study of *Salmo salar* has suggested that the enhanced LA has no effects on fish growth but could result in decreased lipid content in muscle tissues [40]. Accordingly, the strong negative correlation between the content of LA and fat deposition in muscle tissues was also examined in our study. Moreover, this physiological function of LA is not limited in animal models, as LA plays more roles in human health as follows: slightly decreasing abdominal fat accumulation, as well as protecting against death from coronary heart disease and cardiovascular disease [48, 49]. However, another study demonstrated that the nutritious value of ALA for fish products was higher than that of LA. Specifically, prawns fed with supplied ALA diets obtained significantly higher WG than those fed with diets containing an abundance of LA, meanwhile, elevated proportions of n-3 PUFAs were also measured in the ALA-feeding groups [50]. Simply, the higher intensities of both ALA and LA were investigated in GF fish. Following this, the fat deposition in visceral and muscle tissues could be reduced, and the percentages of n-3 series PUFAs could be improved in humans by increasing their consumption of grass-fed *C. idellus*. In summary, GLA, LA, ALA, and EPA were all significantly upregulated in GF groups, illustrating that feeding *C. idellus* grass could improve the nutritional value of the fish flesh. Furthermore, the active ingredients of PUFAs are responsible for lowering triglyceride levels not only in animals but also in humans [11, 20, 40].

Another functionally important set of metabolites that were significantly altered between AF and GF fish are eicosanoids, which are known as the products of enzymatic oxidation of ARA. In humans, n-3 PUFAs together with eicosanoids are engaged in various physiological processes and are essential for normal growth and development [51]. They also play an important role in the prevention of cardiovascular and inflammatory diseases and have a promising impact on the prevention of cognitive decline and dementia in older people [49, 51-53]. Though eicosanoids are ubiquitous in various tissues, their precise physiological roles have not been well defined in animals. In the current study, the different feeding patterns resulted in a significant difference in the concentrations of eicosanoids between GF and AF and were vastly different between female and male metabolic profiles. This meant that metabolic differences between experimental groups were also sex dependent [54]. The details are as follows: LTA4, LTC5, LTE4, and PGG2 were significantly different between FAF and MAF, whereas the differential metabolites between FGF and MGF were LTA4, LTB4, and LTF4. Furthermore, the markedly higher levels of PGG2, LTA4, LTB4, and LTC5 were all measured in GF groups. Accordingly, feeding *C. idellus* a grass diet could not only result in better-quality and more nutritious fish products but also provide higher levels of eicosanoids for consumers' health [49, 51-53]. However, there is other evidence that PGs and LTs are separately generated by the enzymatic action of cyclooxygenases and 5-lipoxygenase, both of which are well-characterized lipid mediators involved in host defense and inflammatory responses [39]. Therefore, the fish fed grass might be in a state of stress. More importantly, although the anti-inflammatory effects of eicosanoids are well known, the side-effects of long-term overuse associated with excessive inflammation, thrombotic tendencies, atherosclerosis, and immune suppression, as well as gastrointestinal complications (e.g., ulceration) and obesity in humans, have been investigated [9, 55, 56]. Due to no definite standard range of eicosanoids content at present, the specific experiments on various doses of eicosanoids and their corresponding physiological functions are urgently needed [37].

### Changes in carbohydrate metabolism in fish muscles induced by different diets

In addition to lipid metabolism, the energy requirement and fat deposition in muscle tissues is also closely related to carbohydrate metabolism, as muscle tissue is a major site of glucose disposal, accounting for approximately 30% of post-prandial glucose disposal [57, 58]. In our study, several metabolites involved in carbohydrate metabolism and activities were greatly increased in FGF and MGF muscle samples, including mannan—a prebiotic in animal husbandry and nutritional supplements, UDP-glucose—an activated form of glucose, UDP-galactose, and amylopectin (the glycogen in animals), as well as Tn-antigen, which were all upregulated in FGF muscle samples [59]. Furthermore, an increased level of diacylglycerol in FGF has been shown to suppress the fat accumulation in fish [60, 61]. Geranylgeranyl pyrophosphate in plants, the precursor to carotenoids and tocopherols, is used to synthetize geranylgeranylated proteins and cholesterol in Perciformes and Salmonidae fish after being consumed [56, 62]. Moreover, α-tocopherol (vitamin E) could be preferentially absorbed and accumulated in humans and has been associated with an enhanced prevention of natural abortions in pregnant women [63]. In summary, feeding *C. idellus* different diets resulted in markedly different metabolic functions, particularly changes in fatty acid metabolism and glucose metabolism [14, 58]. Additionally, we demonstrated that feeding *C. idellus* grass could also improve the contents of physiological active substances in fish muscles, such as those involved in vitamin, amino acid, and steroid hormone metabolism pathways. These beneficial metabolites could then be absorbed and accumulated after consumption by humans and potentially improve physiological functions. Feeding *C. idellus* natural grass can affect the activities of enzymes involved in lipid and carbohydrate metabolism (e.g., acetyl-CoA, glucose-6-phosphate dehydrogenase), modulate the production of metabolites, and decrease fat accumulation, as well as increase fatty acid β-oxidation capacity in muscle tissues, similar to what has been observed in *S. salar* [17]. In addition, feeding with grass could effectively improve the fatty acid compositions and ratio (n-3/n-6) due to the increasing usage of n-6 PUFA-rich ingredients in aquaculture diets [21]. Notably, the higher proportions of n-6 PUFAs in grass-fed *C. idellus* flesh could prevent cardiovascular and inflammatory diseases in humans, and higher n-3 PUFAs cold also play important roles in promoting growth and development, e.g., decreasing hepatic triglyceride content and reducing fat accumulation [20, 64]. Future studies are necessary in order to determine the optimal doses of n-3 PUFA to fish feeds that can improve the concentrations of healthy PUFAs (e.g., ALA, EPA, DPA, and DHA) in fish products, as well as to understand if these beneficial effects can be translated to mammals. These studies will be significant steps toward the goal of meeting consumers’ demand for high-quality, safe, and healthy aquatic products [3].

## Conclusions

In this study, we have conducted a comprehensive physiological, biochemical, and metabolomic investigation of the effects of artificial and grass diet feeding in *C. idellus* and linked these results to specific parameters (e.g., fat accumulation status, muscle fiber thickness, and texture) of flesh quality. It is clear that flesh quality parameters and metabolomic factors are deeply intertwined. The flesh quality-specific differences at the metabolic level were not only related to fat accumulation *invivo* but also affected the final flavor through direct influences on the lipid and carbohydrate metabolism in muscles of *C. idellus*. Moreover, from both environmental and nutritional perspectives, natural grass is a better source of dietary FA and protein when compared to conventional artificial fish feed. This is because grass is more efficiently absorbed and converted into beneficial PUFAs and other nutrients, thereby obtaining higher-quality fish products. In particular, elevated EPA, ALA, stearidonic acid, and some n-3 eicosanoids in muscles of FGF and MGF may improve the ratio of n-3/n-6 PUFA in fish flesh, which is thought to decrease the risk of certain diseases [10, 65, 66]. In addition, the higher levels of mannan, starch, UDP-glucose, UDP-galactose, and dihydroxyacetone phosphate, as well as other metabolites involved in carbohydrate metabolism, are reflective of increased glycometabolism activity in the muscle tissues of grass fed*C. idellus*. It is evident that the *C. idellus* fed *L. perenne*, *E. pectinata*, and *S. sudanense* results in fish with a higher-quality and healthier life than those fed artificial feeds. However, from a commercial point of view, it is still necessary to maintain faster growth and higher yields of *C. idellus* through artificial feeds. Based on the above considerations, we propose that feeding fish with both artificial feed and natural grass in a suitable proportion will produce healthy, fast-growing, and high-yield aquatic products. Further experiments are required to verify and refine the feed in order to achieve optimal growth and health.

## Materials and Methods

### Animals and diets

The fish used in this study were cultured in the basement of the Chonghu Fish Farm, Hubei Province, China. All fish originated from the same batch of *C. idellus* fingerlings, with an initial average weight of 35 g per tail. This study was designed to investigate metabolic alterations in response to different diets. Therefore, fish in one group were fed with natural grass (GF), which included *Lolium perenne*, *Euphrasia pectinata*, and *Sorghum sudanense*. Fish in the other group, the artificial diet group (AF), were fed an artificial diet. The percentages of various nutritional compositions of the two diets are presented in Table 5.

**Table 5:** Percentages of nutrients in the two feeds

**Feed type**	**Crude protein, %**	**Crude fat, %**	**Crude fiber, %**	**Ash, %**
Natural grass	15.3	2.8	25.9	3.5
Artificial feed	28.0	2.6	15.0	15.0

### Experimental procedures

At the beginning of the experiment, about 3,000 grass carp tails were assigned to each pond (roughly 22,666.67 m^2^ per pond), which was co-housed with 550 tails of *Hypophthalmichthys molitrix* (average weight of 14 g per tail) and 350 tails of *Aristichthys nobilis* (average weight of 25 g per tail). Three replicate ponds were used in each experimental group. The feeding experiment spanned from 8 July 2016 to 28 October 2016. During the experimental period, GF fish were fed 100 kg of *L. perenne*, *E. pectinata*, and *S. Sudanense* for each pond per day, whereas 15 kg of artificial diet was supplied to each AG pond two times per day. At the end of the rearing experiment, the fish samples were collected directly at the fish farm. About 85 tails of *C. idellus* were caught from each pond. The total number of fish from each experimental group was 250.

This study complied with the Animal Research: Reporting of In Vivo Experiments (ARRIVE) guidelines and Guidelines for Experimental Animals from the Ministry of Science and Technology (Beijing, China). Further, the Institutional Animal Care and Use Ethics Committee of Huazhong Agricultural University approved the study. All efforts were made to minimize the suffering of sampled fish species.

### Sample collection

Prior to sample collection, *C. idellus* were anesthetized by 100 mg/L MS-222 (Sigma, St. Louis, MO, USA) for 2–4 minutes, then the growth performances were measured for each fish. From 10 tails of fish randomly chosen per experimental group, blood samples (180–200 mL per tail) were taken from the caudal vein without an anti-coagulating substance by injector puncture. The blood samples were placed at room temperature for 30 minutes and then centrifuged at 3,000 *g* for 30 minutes at room temperature for serum preparation. The separated serum was stored at –80°C until the serum biochemical indexes analysis.

White muscle (including those used for both metabolic detections and histological sections) and gonadal tissues were taken from 250 tails per experimental group. The back and abdominal muscle samples were immediately harvested and frozen in liquid nitrogen. Muscle samples were transferred and preserved at –80°C until oil red O staining and subsequent metabolomics analyses. Gonadal tissues and partial abdominal muscle tissues were also collected and kept in Bouin's fixative (saturated solution of picric acid, 75 mL; 40% aqueous formaldehyde, 25 mL; and glacial acetic acid, 5 mL) at room temperature. Serial transverse 10-μm-thick sections of abdominal muscles and gonads were stained routinely with hematoxylin & eosin (H&E). The sex of the grass carp was determined by the contour of the gonad and further results of the gonad tissue slice [67, 68]. To determine the presence of fat in the muscles, frozen muscle tissues were stained with oil red O solution, which would color any fat contained in the muscle.

Based on the results of the sex determination, metabolomic analysis of muscle samples involved division into four test groups (n = 10, each repetition mixed by five individuals randomly selected from each group): FGF, MGF, FAF, and MAF.

### Serum biochemical assay

Serum samples (n = 10) were prepared according to a previously published method [69]. The lactate dehydrogenase (LD), glutamic-oxalacetic transaminase (AST), glutamic-pyruvic transaminase (ALT), alkaline phosphatase (ALP), TCHO, HDLC, GLU, ALB, TP, and TG were measured by automatic biochemistry analyzer (Hitachi 7020, Hitachi High Technologies, Inc., Ibaraki, Japan). Test kits were purchased from the Nanjing Jiancheng Biochemical Corporation (Nanjing Jiancheng Biochemical Corporation, Nanjing, China), and the entire procedure was performed in accordance with the kit instructions.

### Histological observation and analysis

Serial transverse 10-μm-thick sections of muscle tissues were stained with H&E and intracytoplasmic lipids were stained with oil red O (oil O staining) according to previously published procedures [70]. Please note that the samples were selected from the same cohort for metabolome detection; the corresponding sample numbers were the same as samples used for metabolic tests (n = 50). A total of 200–400 fibers of white muscle per fish were studied using a Leica MZ 6 microscope for their cross-sectional area, and the diameter (d = 2r) of each fiber was calculated from the fiber area (A) (A = π·r^2^), thus, d = 2*√ (A·π^−1^)). A size limit for identifying fibers was set at fiber diameters ≥10 μm as the optical resolution below this limit did not allow for sufficient identification and accuracy in the analyses [71]. The circularity of each fiber was also determined. The free software Image J [72] was used for quantitative statistics and analyses.

### Sample preparation for LC-MS

The metabolomic analysis of muscle samples was carried out on four test groups (n = 10). Frozen white muscle samples were thawed slowly, taken from the ultra-cold freezer (–80°C), placed at –20°C for 30 minutes, and then put on ice until the samples were completely melted. Each repetition from each experimental group was taken from five individuals (approximately 25 mg per individual). Samples of five tails were placed in an eppendorf (EP) tube tube and mixed with 800 μL of an ice-cold mixture of methanol and water (1:1 ratio), with two steel balls added to each tube. The tissues were then broken at 60 Hz for 5 minutes by the TissueLyser, then 300 μL of supernatant from each tube was collected after a 10-minute centrifugation at 25,000 *g*at 4°C and then injected into the LC-MS system. Ten microliters of each sample was combined into a new vial and used as a pool sample for quality control and analyte identification; these were acquired after every 10 tested samples.

### Chromatography and mass spectrometry conditions

Chromatographic separations were performed using the ultra-performance liquid chromatography (UPLC) system Ultimate 2777C (Waters, UK). An ACQUITY UPLC BEH C18 column (100 mm* 2.1mm, 1.7 μm, Waters, UK) was used for the reversed phase separation. The column oven was maintained at 50°C. The injection volume for each sample was 10 μL, and the flow rate was 0.4 mL/min. Additionally, the mobile phase consisted of solvent A (water + 0.1% formic acid) and solvent B (acetonitrile + 0.1% formic acid). Gradient elution conditions were set as follows: 0∼2 min, 100% phase A; 2∼11 min, 0% to 100% B; 11∼13 min, 100% B; and 13∼15 min, 0% to 100% A.

The eluents were introduced into a high-resolution MS/MS spectrometer Xevo G2-XS QTOF (Waters, UK) by electrospray ionization with capillary voltages set in the positive and negative modes to 2.0 kV and 1.0 kV, respectively. The cone voltages of both modes were 40 V. The MS data were acquired in Centroid MSE mode. The TOF mass scan range of both simultaneous low- and high-energy mass scan functions was from 50 m/z to 1200 m/z, with a scan time of 0.2 seconds. For the MS/MS detection, all precursors were fragmented using 20–40 eV. During the acquisition, the MS signal was acquired every 3 seconds to calibrate the mass accuracy.

### Data processing and metabolite identification

For qualitative and quantitative metabolomics, raw data were processed using Progenesis QI software (Nonlinear Dynamics, 2017, version: 2.2, Waters, MA, US). First, data were cropped to remove external standards. Masses were detected, and the chromatogram for each mass was built using the Centroid mass detector and Chromatogram builder. Smoothed data were then deconvoluted using a noise amplitude algorithm and deisotoped. The conditions for chromatographic alignment were 0.01 m/z tolerance and 0.1 min RT tolerance. Finally, a sodium and ammonium adducts search was performed prior to exporting the data to Excel for post-processing. The compound identification list, which contained the molecular weight, compound name, statistical scores, and other information to show the result of the identifications, was exported as a .CSV delimited text file .

To verify and confirm compound identifications, the METLIN batch Metabolite Search [73], KEGG [74], Human Metabolite [75], and ChemSpider [76] databases were used by comparing molecular weights and MOL files. The molecular and structural formulas of the candidate compounds were retrieved by the comparison and then confirmed by MS/MS scans for the characteristic ions and fragmentation patterns of the metabolites.

### Statistical analyses

The peak intensity tables of detected features were input into the MetaboAnalyst 4.0 [22 Statistical Analysis module for univariate and multivariate data analyses [23]. The input data were normalized by a pooled sample (QC) from the two experimental groups. Log transformation was also used in data normalization procedures. Univariate data analysis was applied to the metabolomics data using the Student *t*test. Statistical significance was set at *P* < 0.05 and 0.05 < *P* < 0.10 as trends. Multiple testing corrections were performed based on FDR-adjusted *P* values (*q*values) with a significance threshold set at *q*value <0.05 [77]. For multivariate analysis, the data were subject to PCA for pattern discovery. For clustering analysis, a heat map was created based on log-transformed relative intensities of detected features.

Pathway analysis was performed using the Pathway Analysis module, using the list of compound names manually annotated based on the significant peaks. To further validate the result, as well as to adjust for potential bias, we also applied the recent MS Peaks to Pathways module (mummichog) of MetaboAnalyst using the entire list of MS peaks [78]. The *P* value cutoff for the MS Peaks to Pathways module was 0.05, and we used the *D. rerio* as the reference library. The R-command history file generated throughout our analyses on MetaboAnalyst is available in the Supplementary Materials (Female-MetaboAnalyst-Rhistory.R and Male-MetaboAnalyst-Rhistory.R, respectively).

## Availability of source code and requirements

Project name: Metabolic Alterations Induced by Different Diets

Project home page: https://github.com/Zhao253091640/HZAU-Prof.-Dapeng-Li-s-Laboratory

Operating system(s): platform independent

Programming language: R

License: GNU General Public License version 2.0 (GPLv2).

Any restrictions to use by non-academics: none

## Availability of supporting data

Our metabolomics raw data have been deposited to the EMBL-EBI MetaboLights database (DOI: 10.1093/nar/gks1004. PubMed PMID: 23 109 552) with the identifier MTBLS673 [24, 26. In addition, the preliminary list of compound identifications and information on significant differential metabolites (such as potential mapped metabolites, their query IDs, *P* value, FC, FDR, and their corresponding metabolic pathways are proved in Supplementary Materials. The data further supporting this work are available in the GigaScience repository, GigaDB [26].

## Additional files

**Supplementary Figure S1:** The PCA loading plots for the metabolomic data of muscle samples from female (A) and male (B) *C. idellus*.

**Supplementary Table S1:** List of Discriminating Metabolites between Female-Artificial feed feeding group (FAF) and Female-Grass feeding group (FAF) *C. idellus*.

**Supplementary Table S2:** List of Discriminating Muscle Metabolites between MAG and MGG *C. idellus*. The intensity of the most abundant metabolites in females, and the intensity of the metabolites were “normalized”. Putatively identified using KEGG and HMDB.

**Supplementary Figure S2:** The Pearson's correlation analyses for the discriminating metabolites of lipids and carbohydrates metabolisms in muscle tissues of female (A) and male (B) *C. idellus*, respectively. The differential signatures were annotated with their potential metabolite names after mapping with compound databases. The diversity of color referred to the pair-wise correlation coefficient ranging from 1 (red) to -1 (blue).

**Supplementary Figure S3:** The pathway enrichment and network analyses for the significant metabolites in male *C. idellus*. (A) The scatter plot was used to visualize the pathway impact and enrichment results for all matching significant metabolites in male *C. idellus*; (B) The KEGG global metabolic network visualization of all significant metabolites (*P* < 0.05) in the male *C. idellus* metabolic profile. The colored points represent different metabolic pathways. The various color levels indicate different levels of significance of metabolic pathways from low (white) to high (red). The different sizes of each point were used to represent the number of metabolites participated in the metabolic pathway. The greater rich factor, the greater the degree of pathway enrichment. Moreover the corresponding pathway's name of each point is labeled. In the metabolic network, all up-regulated metabolites (FC AF/GF > 2) in AF groups were colored with red, whereas the down regulated metabolites (FC < 0.5) were colored in green. In addition, the different color circles represent the various physiological functions that the discriminating metabolites belong to. Moreover, each enriched pathways is annotated with the corresponding name.

**Supplementary Figure S4:** Visualization of overlapped significant metabolites onto corresponding pathways. (A) The overlapped metabolites in female *C. idellus* highlighted in significantly enriched pathways; (B) The pathway view for the altered metabolites between MAF and MGF. Light blue compounds in the figures mean that these metabolites were undetected in our data, but used as background for pathway enrichment analysis. Red colored compounds mean the metabolites were detected in our metabolomic data and involved in the specific metabolism pathway.

## Abbreviations

AF: artificial feed; ALA: alpha-linolenic acid; ALB: albumin; ARA: arachidonic acid; CF: condition factor; DGLA: dihomo-gamma-linolenic acid; DHA: docosahexaenoic acid; DPA: docosapentaenoic acid; EPA: eicosapentaenoic acid; FA: fatty acid; FAF: female artificial feed; FC: fold change; FDR: false discovery rate; FGF: female grass feed; UPD-glucuronic acid: uridine diphosphate-glucuronic acid; GD2: ganglioside G D2; PGG2: prostaglandin G2; GF: grass feed; GLA: gamma-linolenic acid; GLU: glucose; H&E: hematoxylin & eosin; HDLC: high-density cholesterol; HETE: hydroxy-eicosatetraenoic acid; KEGG: Kyoto Encyclopedia of Genes and Genomes; LC: liquid chromatography; LTs: leukotrienes; LTB4: leukotriene B4; LTE4: leukotriene E4; MAF: male artificial feed; MGF: male grass feed; MOL: the file extension of MDL Molfile chemical file format; MS: mass spectrometry; PCA: principle component analysis; PIP3: phosphatidylinositol triphosphate; PUFA: polyunsaturated fatty acid; QC: quality control; TCHO: total cholesterol; TG: triglycerides; TP: total protein; SDM: significant discriminating metabolite; SFA: saturated fatty acid; SGR: specific growth

## Competing interests

The authors declare that they have no competing interests.

## Funding

This work was supported by the Earmarked Fund for China Agriculture Research System (CARS-45), National Natural Science Foundation of China (project 31 502 140), and the Fundamental Research Funds for the Central Universities (2662015PY119). H.Z. is supported by the China Scholarship Council, which supports her study at McGill University (CSC 201 706 760 039).

## Author contributions

H.Z. had roles in designing the study, culturing fish, collecting samples, collecting data, and performing analysis. The manuscript was written through contributions from H.Z, J.X., and D.L. J.C. did valuable assistance in data analysis. All authors gave approval to the final version of the manuscript and the decision to submit the work for publication.

## Supplementary Material

GIGA-D-18-00146_(Original_Submission).pdf

GIGA-D-18-00146_Revision_1.pdf

Response_to_Reviewer_Comments_Report_(Original_Submission).pdf

Reviewer_1_Report_(Original_Submission) -- Tim Young, Ph.D 06/04/2018 Reviewed

Reviewer_1_Report_Revision_1 -- Tim Young, Ph.D 7/26/2018 Reviewed

Reviewer_2_Report_(Original_Submission) -- Serap Saglik Aslan 6/21/2018 Reviewed

Supplemental Files
