# Supplementary material for: Metabolomics investigation of dietary effects on flesh quality in grass carp (Ctenopharyngodon idellus)
Source: Gigascience. 2018 Sep 6;7(10):giy111. doi: 10.1093/gigascience/giy111 (PMC6176498; doi:10.1093/gigascience/giy111)
Supplement: GIGA-D-18-00146_Revision_1.pdf [file giy111_giga-d-18-00146_revision_1.pdf]

GigaScience

Metabolomics Investigation of Dietary Effects on Flesh Quality in Grass Carp (Ctenopharyngodon idellus)

--Manuscript Draft--

**Manuscript Number:**	GIGA-D-18-00146R1
**Full Title:**	Metabolomics Investigation of Dietary Effects on Flesh Quality in Grass Carp (Ctenopharyngodon idellus)
**Article Type:**	Research
**Funding Information:**	China Agriculture Research System (CARS-45) — Prof. Dapeng Li<br>National Natural Science Foundation of China (31502140) — Prof. Dapeng Li<br>Fundamental Research Funds for the Central Universities (2662015PY119) — Prof. Dapeng Li<br>China Scholarship Council (201706760039) — PhD Honghao Zhao
**Abstract:**	Background: The ultra-high density intensive farming model of grass carp (Ctenopharyngodon idellus) may elicit growth inhibition, decline flesh quality and increase disease susceptibility of fish. The quality degradation and excessive fat accumulation in cultured C. idellus have long been attributed to possible alterations in the lipid metabolism of fish muscle tissues as a result of over-nutrition from artificial diets. To investigate the effects of different diets on fish muscle quality, a large-scale metabolomics study was performed on 250 tails of C. idellus.<br>Findings: The experimental fish were divided into four groups based on sex and diets - female artificial feed (FAF), female grass feed (FGF), male artificial feed (MAF) and male grass feed (MGF). After 113 days rearing period, the AF group showed significantly higher total mass of muscle fat ($P < 0.01$), with the FAF group being the highest. Metabolomics profiling based on liquid chromatography-mass spectrometry (LC-MS) revealed distinctive patterns of clustering according to the four groups. Overall, artificial feeding was associated with higher concentrations of docosapentaenoic acid (DPA), dihomo-gamma-linolenic acid (DGLA) and arachidonic acid (ARA); whereas grass-feeding was associated with elevated n-3 unsaturated fatty acids (n-3 UFAs), such as eicosapentaenoic acid (EPA), alpha-linolenic acid (ALA) and gamma-linolenic acid (GLA). Artificial feeding also resulted in significant increased docosahexaenoic acid (DHA) in MAF muscle than MGF fish, whereas no significance in the comparison of female samples. Metabolic pathway analyses using both targeted and untargeted approaches consistently revealed that the arachidonic acid metabolism and steroid hormone biosynthesis pathways were significantly different between AF and GF groups.<br>Conclusions: Our results suggested that grass is a better source of diet fatty acid and protein when compared to artificial feed. Grass feeding could effectively lower triglycerides in serum, reduce fat accumulation and alter lipid compositions in fish muscle by increasing the concentrations of n-3 UFAs, leading to better nutrition and health.
**Corresponding Author:**	Dapeng Li, PhD<br>College of Fisheries<br>Wuhan, Hubei Province CHINA
**Corresponding Author Secondary Information:**	
**Corresponding Author's Institution:**	College of Fisheries
**Corresponding Author's Secondary Institution:**	
**First Author:**	Honghao Zhao, PhD
**First Author Secondary Information:**	

Powered by Editorial Manager® and ProduXion Manager® from Aries Systems Corporation

**Order of Authors:**	Honghao Zhao, PhD
	Jasmine Chong
	Rong Tang, PhD
	Li Li, PhD
	Dapeng Li, PhD
	Jianguo Xia, PhD
**Order of Authors Secondary Information:**	
**Response to Reviewers:**	Dear Dr. Nogoy and reviewers,<br><br>We gratefully appreciate the helpful comments and suggestions from the editor and reviewers. We have carefully revised our manuscript accordingly, changes in text are highlighted in yellow, and our item-wise replies are provided below.<br><br>Tim Young, Ph.D (Reviewer 1)<br><br>Major questions.:<br>1)Please clarify sample replication, as they are not currently sufficiently detailed to allow study reproduction. Two diets were tested, and three replicate pond systems per treatment group were used. However, it is not clear how the ten replicate fish muscle samples for metabolomics were obtained from this design; precisely how many fish per pond were sampled for metabolomics, and how exactly were they selected? Please provide similar information for the serum samples.<br>Response: Thank you for your valuable advice. The detailed descriptions of sample replications have been modified accordingly in text, which were also highlighted.<br>About 85 tails of C. idellus were caught from each pond. Meanwhile, the total number of fish from each experimental group was 250 (Line 454 to 455).<br>White muscle (including those used for both metabolic detections and histological sections) and gonadal tissues were taken from 250 tails per experimental group. (Line 467 to 468). Blood samples were collected from 5 fish per pond, however, the final presented results were measured and calculated from 10 tails of fish (n = 10) per experimental group (Line 482).<br>In addition, the muscle samples used for LC-MS detections were randomly selected based on sex determinations. (Line 477 to 478). Based on sexes, metabolomic analysis of muscle samples were divided into 4 test groups (n = 10, each repetition mixed by 5 individuals), each repetition was taken from 5 individuals (approximately 25 mg per individual) (Line 5024 to 503).<br>Additionally, please note that the samples were selected based on the same cohort for metabolome detection, the corresponding sample numbers were same with samples used for metabolic tests (n = 50) (Line 491 to 493).<br><br>2)Please provide replication information (n = ?) in legends for all relevant figures and tables (i.e., Figs 2B & 4; Table 1 & 2).<br>Response: We agree that providing replication information to all relevant figures/tables is necessary and have modified the related legends and notes according to the reviewer's suggestions. In detail, we have provided the related replication information for Figures 1, 2, and 4, as well as Tables 1 and 2.<br><br>3)Figure 2B: Please state what data represent e.g., mean +/- SD or SE.<br>Response: We have clarified in the manuscript what the data in Figure 2B represents, providing the corresponding explanation in the figure legend: "Vertical bars represent the mean±S.E." (Line 774).<br><br>4) Were metabolite profile data normalized at all to biomass?<br>Response: Thank you for your question. We have described the related details concerning metabolomics sample collection in the manuscript. Each sample for metabolomic detection of muscle samples (n = 10) consisted of a mixture of 5 individuals (25 mg per individual) (Line 477-478). All replicate samples used for metabolome detection were therefore the same weight (125 mg).<br><br>5) Why were two different normalization approaches used for the multivariate analyses

(i.e., auto-scaled for PCA and log transformed for heatmap and cluster analysis)?
Response: Thank you so much for pointing out this issue. In this study, we consistently used the log-transformation method during the analysis of metabolome profiles. We updated the corresponding text (Line 541 to 546):
" The log transformation and auto-scaling were also used in data normalization procedures. Univariate data analysis was applied to the metabolomics data using the Student's t-test. Statistical significance was set at $P < 0.05$ and $0.05 < P < 0.10$ as trends. Multiple testing corrections were performed based on false discovery rate (FDR)-adjusted p-values (q-values) with a significance threshold set at q-value < 0.05 [28]. For multivariate analysis, the data were subject to principle component analysis (PCA) for pattern discovery. For clustering analysis, a heatmap was created based on log-transformed relative intensities of detected features."

6) Please provide information on the exposure duration of MS-222, and to what level of anaesthesia the fish were dosed to.
Response: Thanks for the advice. We have provided further details of the anesthetic treatment as follows (Line 461-462): "Prior to sample collection, C. idellus were anesthetized by 100 mg·L−1 MS-222 (Sigma, St. Louis, Missouri, USA) for 2-4 minutes, then the growth performances were measured for each fish."

7)Line 271: It seems like a very large dataset of putative metabolite identifications was generated during this study, features of which may be of interest to some readers. This data is mentioned but not shown or provided - perhaps it might be useful to make it accessible. This is not essential for the manuscript, but it would make a nice addition.
Response: Thank you for your valuable suggestion. The metabolomics data have been submitted the data to MetaboLights and we intend to make it available to public. However, we may not be able to disclose this part of data for the time being. This is due to the fact that part of data being carried out comparison and cross-analysis with our other detected multiple-omics data. It may be used and presented in the follow-up research manuscript.

8) The title is misleading, and I would recommend re-wording it. I expected to see an analysis of correlations between the metabolite profiles and flesh quality. This was not the case; apart from fat content and muscle fiber thickness, other measures of fish flesh quality were not evaluated (e.g., appearance, taste, texture, gaping), and the experimental design does not appear to permit such correlations to be tested. I suggest authors make clear the difference between 'potential associations' and true 'correlations' throughout the manuscript. Indeed, a simple suggestion to make the title more reflective of the work being presented would be to just change the word 'correlations' to 'associations'.
Response: We appreciate the reviewer's reasonable proposal and recommendation for a more accurate title. In order to better summarize the results presented in this manuscript, we changed the title to "Metabolomics Investigation of Dietary Effects on Flesh Quality in Grass Carp (Ctenopharyngodon idellus)".
Furthermore, the basic experimental measurements of fish quality (including appearances, textures, shear force detection, nutritional components in muscle tissues and so on) of the two feeding group Ctenopharyngodon idellus have been measured and analyzed in our previous work and have been summarized in two previously published articles. They both are used as references in this manuscript, Ref [21]: Zhao HH, Xia JG, Zhang X, He XG, Li L, Tang R, Chi W, Li DP. Diet Affects Muscle Quality and Growth Traits of Grass Carp (Ctenopharyngodon idellus): A Comparison Between Grass and Artificial Feed. Front. Physiol 2018; 9: 283-295; the other Ref [30] is published in Chinese, and compared the test results of nutritional components (e.g. 17 kinds of amino acids, pH, crude contents and 17 kinds of fatty acids) in muscle tissues of GF and AG fish.
Although these specific test results were not presented in this manuscript, we still had a correlation analysis between the current metabolomics detection results and the previous basic experimental results in the discussion section. We hope to fully analyze the differences in growth, physiology and metabolome between the GF and AF groups, as well as to reveal the potential regulatory mechanisms causing these differences, from epigenetic to molecular, and then to mechanism.
We agree with the reviewer's comments that the "correlations" mentioned and revealed in our manuscript are only "potential links or associations". Accordingly, in view of the major questions the reviewer raised, we further strengthened the descriptions of

previously published research findings in the revised version, so that readers and researchers can easily and clearly understand and support the possible associations or related regulatory mechanisms revealed in our research. Accordingly, we have recited the data of flesh quality of fish under the same conditions we published recently, and further strengthened the discussion on the potential relations between flesh quality and muscle metabolites.

9)Related to the previous comment, components of the discussion are speculative and would benefit from revision. For example, authors state in Lines 317-319 that the higher levels of SFA's (i.e., free [?] C20:0, C18:0 and C16:0) in GF fish would result in a crisper taste and firmer flesh (thus altering taste, texture and quality). Although a citation is provided (albeit from cow meat which may not translate to fish), these indices were not actually tested and is therefore highly speculative in my opinion for a manuscript that centres itself largely on flesh quality. The language therefore should to be toned appropriately to reflect this. The manuscript could be suggestive and build upon the novelty of hypothesis generation, rather than projected to provide conclusive evidence.
Response: We thank the reviewer for their insightful comment. As mentioned in our answer to the previous question, The results of muscle characteristics detections (such as texture, amino acids and fatty acids in muscle tissues, as well as pH) of C. idellus fed with two different diets were summarized and represented in our two previously published articles, Ref [21] and Ref [30]. In these papers, a series of physiological indicators (e.g. growth rate, texture, shear force, pH, crude contents, and profiles of fatty acids and amino acids, as well as the expressions of the growth-related genes) were compared in muscle tissues of GF and AG fish. We therefore used these data as references to discuss in the manuscript. Although these specific measurements were not carried out in this paper, we conducted a correlation analysis between the current metabolomics detection results and the previous basic experimental results in the discussion section. We hope to fully analyze and reveal the potential regulatory mechanisms causing metabolic alterations and muscle characteristic differences, from epigenetic to molecular, then to metabolic pathway analyses.
Accordingly, we further strengthened the detailed descriptions of references and our previously published research findings in the revised version, so that readers and researchers can be easier, clearer, and support the possible associations or related regulatory mechanisms in our research.
Based on the reviewer’s comment, the rewritten sentence is as follows: “As reported in other research, SFAs (e.g. C16:0 and C18:0) play important roles in influencing flesh texture, with higher levels resulting in a “crisper” flesh taste [30,44]. In line with the previous findings, the increased arachidic acid (20:0), stearic acid (18:0) and palmitic acid (16:0) were detected in our GF groups, therefore the flesh of GF C. idellus would be harder than that of AF fish. This conclusion is consistent and proved by our previously published test results of textures, shear force detection and fatty acid contents measurement of the two feeding groups C. idellus flesh [21, 30].” (Line 315 to 320)

10)Lines 308-309: This sentence should be re-phrased as it suggests that correlations between physiological changes and metabolic profiles were systematically studied by the authors. Unless I have missed some core information/dataset, I cannot see such correlation analyses. Also, please define 'physiological changes' if the phrase is to be used.
Response: We thank the reviewer for their recommendations. Accordingly, we have modified the text in manuscript. The corresponding sentence was changed to “Furthermore, the potential associations between physiological changes (the muscle characteristics, together with nutrients and textures measured in our previously published studies) [21,30] and metabolic profiles in different feeding conditions will be systematically further examined below.” (Line 303 to 305).

11) Lines 419-421: Authors state that they correlated physiological, biochemical and metabolic parameters with specific parameters of flesh quality. Please re-state. Perhaps use the term 'associated' or 'linked' rather than correlated.
Response: Thanks for your advice. We modified the text in the manuscript according to your comment (Line 420 to 421), and enumerated the specific test parameters in order to give readers a better understanding of our narration.

Powered by Editorial Manager® and ProduXion Manager® from Aries Systems Corporation

12)Lines 422-423: The results indicate that flavour may have been affected, or perhaps even likely. However, a flavour analysis was not performed in this study. Please re-phrase accordingly.
Response: In view of the major questions you raised, we corrected and unified the word 'correlations' to 'associations' in the whole manuscript.
However, you pointed out the major problem, Although flavor or texture analysis was not performed in this work, a flavors analysis of the same fish in the two feeding group were conducted in our previous publish works (references [21] and [30] in this manuscript). Both studies had demonstrated that the flavors were affected by the two different feeding diets.
Although these specific test results were not reproduced in this manuscript, we still correlated metabolomics alterations with the previously basic physiological and biochemical results in the discussion section. We hope to fully understand the differences in growth, physiology and metabolome of fish caused by feeding with grass and artificial feed, as well as reveal the potential regulatory mechanisms resulting in these differences, from epigenetic to molecular, then to mechanism.

13) Please highlight somewhere that the results of this particular study does not necessarily mean that all AF diets will perform worse than GF diets.
Response: We appreciate your reasonable proposal and agree with your point. Therefore, the related contents were added and the last conclusion section was rewritten as follows: "However, from commercial point of view, we still need to maintain the faster growths and higher yields of C. idellus contributed by artificial feeds. Based on the above considerations, we propose that feeding fish with both artificial feed and natural grass in a suitable proportion will produce healthy, fast growing and high yield aquatic products. Further experiments are required to verify and to refine the feed in order to achieve optimal growth and health." (Line 433 to 437).

14) Lines 438: What ratios by mass were these grasses fed to the fish?
Response: During the whole feeding experiment, we fully fed grass carp to satiation status. We weighed the fresh weight of the forage, not dehydrated. A total of 100 kg of L. perenne, E. pectinata and S. Sudanense were fed to GF group C. idellus each pond per day (Line 452).
On the other hand, the use of the three species of natural grasses is not determined by some specific collocation ratios. It depends on the time/ month course of the whole rearing experiment. Specifically, the best growth period of Lolium perenne is from May to July, that of Sorghum sudanense is from July to September. Compared with the other two grasses, Euphrasia pectinata has the longest suitable growth periods. However, its interlude of circular harvesting is very long, needs 3-4 weeks. Accordingly, our feeding experiment spanned from July 8th, 2016 to October 28th, 2016. During the experimental period, we fed fish with L. perenne at the beginning, it was planted in May, and S. Sudanense was planted at the same time. After July, S. Sudanense could be fed to our GF C. idellus. In addition, natural grasses feeding for all experimental fish should be fully fed. As a result, E. pectinata was repeatedly cut and intermittently fed to the fish during the 113 days.

15) Table S1: Correct the abbreviation for the 'Female-Grass feeding group' (i.e., FAF to FGF)
Response: I'm so sorry for this wrong abbreviation. It has been corrected.

Serap Saglik Aslan (Reviewer 2)

Comments to Author:
1.In the Abstract, the sentence that "DHA was only found in the male samples" is not impossible. Because DHA (Docosahexaenoic acid) is found in the fish tissues certainly. So If you can evidence this matter with a literatüre It may be acceptable. Otherwise There is a big mistake in the experimental of the study.
Response: We appreciate the reviewer's comment. According to the question, we reconfirmed our metabolomics test results. This is caused by our inaccurate expression. The docosahexaenoic acid (DHA) was really detected in all of the four groups muscle samples (including FAF, FGF, MAF and MGF). Hence, we reworded this sentence as "Artificial feeding also resulted in significant increased docosahexaenoic acid (DHA) in MAF muscle than MGF fish, whereas no significance

Powered by Editorial Manager® and ProduXion Manager® from Aries Systems Corporation

	in the comparison of female samples.” (Line 43 to 45).<br>We mean that although DHA has been detected in the four groups samples, the comparisons of DHA between the two feeding patterns (FAF and FGF; MAF and MGF), it only showed significant difference in the comparison between MAF and MGF (q-value < 0.05). In other words, there is no significance in the relative intensities of DHA in FAF and FGF muscle samples (q-value > 0.05).<br><br>2.In the Materials and Methods, the title of "Experimental Design" must be changed as "Experimental part". Because experimental design has a different meaning. You can check in the literatüre.<br>Response: Sorry for this mistake. It has been corrected to “Experimental Procedures” (Line 447)<br><br>3.In Table 5, The total percentages are 125.9 for natural grass and 73.6 for artificial feed. 125.9 is impossible. Because; % moisture+%crude protein+%crude fat+ %crude fiber+%ash+%carbohydrate=100 in the proximate analysis. So it must be corrected.<br>Response: We thank the reviewer for pointing out this problem. In this experiment, the artificial feed is most frequently used in the aquaculture of farmed grass carp with known feed ingredient formula. Meanwhile, the natural grass is the traditional feed in the culture history of grass carp. Because the influence of exact nutrients in both diets on muscle characteristics and metabolites is not the final aim of our current study, we detected the crude nutrients in the two diets. In order to compare the specific and detected components between the two diets, we only listed the percentages of nutrients in artificial feeds corresponding to the detected contents in forage. Therefore, the percentages of many other detailed components in the AF (showed in the feed ingredient formula) were not listed. Consequently, the total percentage of artificial feeds used in our experiment is less than 100%.<br>For the natural grass, we weighed the fresh weight of the grasses under uncleaned and untreated status. As the grass were undehydrated, the soil composition could result in serious errors in the initial weighing, which will directly influence the accuracy of moisture detected data. Because we must present the original, available and real measured results, we removed the percentages of moisture in the two diets, making the overall feed nutrient compositions more reasonable, available, without affecting their authenticities.
**Additional Information:**	
**Question**	**Response**
Are you submitting this manuscript to a special series or article collection?	No
**Experimental design and statistics**<br><br>Full details of the experimental design and statistical methods used should be given in the Methods section, as detailed in our Minimum Standards Reporting Checklist. Information essential to interpreting the data presented should be made available in the figure legends.<br><br>Have you included all the information requested in your manuscript?	Yes
**Resources**	Yes

Powered by Editorial Manager® and ProduXion Manager® from Aries Systems Corporation

A description of all resources used, including antibodies, cell lines, animals and software tools, with enough information to allow them to be uniquely identified, should be included in the Methods section. Authors are strongly encouraged to cite Research Resource Identifiers (RRIDs) for antibodies, model organisms and tools, where possible.<br><br>Have you included the information requested as detailed in our Minimum Standards Reporting Checklist?	
**Availability of data and materials**<br><br>All datasets and code on which the conclusions of the paper rely must be either included in your submission or deposited in publicly available repositories (where available and ethically appropriate), referencing such data using a unique identifier in the references and in the "Availability of Data and Materials" section of your manuscript.<br><br>Have you have met the above requirement as detailed in our Minimum Standards Reporting Checklist?	Yes

Powered by Editorial Manager® and ProduXion Manager® from Aries Systems Corporation

Manuscript with DOI
Click here to view linked References
Click here to access/download;Manuscript;Revised ZHH_Metabo_Manu_GigaSci with DOI.docx

Metabolomics Investigation of Dietary Effects on Flesh Quality in Grass Carp (*Ctenopharyngodon idellus*)

Honghao Zhao[1,2], Jasmine Chong[2], Rong Tang[1], Li Li[1], Jianguo Xia[2,3*] and Dapeng Li[1*]

[1]College of Fisheries, Hubei Provincial Engineering Laboratory for Pond Aquaculture, National Demonstration Center for Experimental Aquaculture Education, Huazhong Agricultural University, Wuhan 430070, China

[2]Institute of Parasitology, and [3]Department of Animal Science, McGill University, Saint-Anne-de-Bellevue, QC H9X 3V9, Canada

Running title: Diet affects fish muscle quality and metabolic alterations of muscle tissues

ms. has 29 pages, 6 figures, 5 tables.

***Corresponding authors:**

1. Prof. Dapeng Li

College of Fisheries, National Demonstration Center for Experimental Aquaculture Education, Hubei Provincial Engineering Laboratory for Pond Aquaculture, Huazhong Agricultural University

No. 1, St. Shizishan, Hongshan District, Wuhan, 430070, China

Tel: +086 15307118600;

Fax: +086 027-87282113

E-mail: ldp@mail.hzau.edu.cn

2. Prof. Jianguo Xia

Institute of Parasitology, and Department of Animal Science, McGill University, Saint-Anne-de-Bellevue, QC H9X 3V9, Canada

Tel: +001 (514) 398-8668;

Fax: +001 (514)-398-7857

E-mail: jeff.xia@mcgill.ca

Abstract

Background: The ultra-high density intensive farming model of grass carp (*Ctenopharyngodon idellus*) may elicit growth inhibition, decline flesh quality and increase disease susceptibility of fish. The quality degradation and excessive fat accumulation in cultured *C. idellus* have long been attributed to possible alterations in the lipid metabolism of fish muscle tissues as a result of over-nutrition from artificial diets. To investigate the effects of different diets on fish muscle quality, a large-scale metabolomics study was performed on 250 tails of *C. idellus*.

Findings: The experimental fish were divided into four groups based on sex and diets - female artificial feed (FAF), female grass feed (FGF), male artificial feed (MAF) and male grass feed (MGF). After 113 days rearing period, the AF group showed significantly higher total mass of muscle fat ($P < 0.01$), with the FAF group being the highest. Metabolomics profiling based on liquid chromatography-mass spectrometry (LC-MS) revealed distinctive patterns of clustering according to the four groups. Overall, artificial feeding was associated with higher concentrations of docosapentaenoic acid (DPA), dihomo-gamma-linolenic acid (DGLA) and arachidonic acid (ARA); whereas grass-feeding was associated with elevated n-3 unsaturated fatty acids (n-3 UFAs), such as eicosapentaenoic acid (EPA), alpha-linolenic acid (ALA) and gamma-linolenic acid (GLA). Artificial feeding also resulted in significant increased docosahexaenoic acid (DHA) in MAF muscle than MGF fish, whereas no significance in the comparison of female samples. Metabolic pathway analyses using both targeted and untargeted approaches consistently revealed that the arachidonic acid metabolism and steroid hormone biosynthesis pathways were significantly different between AF and GF groups.

Conclusions: Our results suggested that grass is a better source of diet fatty acid and protein when compared to artificial feed. Grass feeding could effectively lower triglycerides in serum, reduce fat accumulation and alter lipid compositions in fish muscle by increasing the concentrations of n-3 UFAs, leading to better nutrition and health.

Keywords: Diets, Metabolomics, Flesh quality, Fat deposition, *Ctenopharyngodon idellus*

Background

Grass carp (*Ctenopharyngodon idellus*) is an important freshwater aquaculture fish species worldwide, accounting for 7.6% (5.8 million tons in 2015) of total freshwater aquaculture production in the world [1]. Intensive fish farming based on the utilization of artificially formulated feeds has played a critical role in the continuing increase in fish production [2]. However, the flesh quality of farmed *C. idellus* has declined during the course of intensive aquaculture,

which has become a growing public concern [3]. It is now generally agreed that production improvement should no longer be the primary goal in aquaculture practice. To obtain high quality fish products, whilst maintaining a sustainable aquaculture, has become an important objective in current aquaculture [4,5].

One approach to obtain a sustainable aquaculture is to make full use of new technologies available to the scientific community. Over the last few years, high-throughput omics technologies, such as genomics, transcriptomics, proteomics and metabolomics, have widely been used to enable detailed understanding of molecular changes in different organisms, showing great potential to transform aquaculture research [6,7]. Metabolomics is the systematic study of all small molecules in a biological system such as cells, biofluids or tissues. Global (or untargeted) metabolomics is particularly suitable for comprehensive metabolome characterization and novel biomarker discovery. High-resolution MS systems coupled with liquid chromatography (LC) have become the main workhorse in global metabolomics [8]. It has been widely applied to understand the effects of the diets and nutrition strategies for diseases prevention and treatment in human populations. These studies have revealed that the high ratio of n-3/n-6 polyunsaturated fatty acids (PUFAs) in diets had protective effects on the risk of obesity, breast cancer and hypertriglyceridaemia [9-11]. These findings have important implications for animal nutrition research that aim to enhance the ratio of n-3/n-6 PUFA in milk and meat products. For instance, Bertol et al. have shown that the concentrations of n-3 PUFAs were significant higher in the meat of pigs fed with canola or canola+flax oil diets, compared to pigs fed with a soybean oil diet [12]. A similar study on cows demonstrated that milk from cows fed with grass enriched in n-3 fatty acids contained more n-3 fatty acids than milk from cows fed with conserved grass [13]. Metabolomics has also increasingly contributed to understanding the effect of different diets or dietary patterns on fish [6,14]. For instance, feeding plankton to carp was shown to enhance the content of n-3 PUFAs, especially eicosapentaenoic acid (EPA) and docosahexaenoic acid (DHA), whereas feeding carp a diet with rapeseed induced higher oleic acid levels and lower levels of n-3 PUFAs [14]. Metabolomics was also used to explore the possibility of replacing the fishmeal component in artificial diets with zygomycetes, as well as to compare the fatty acid compositions between artificial farmed fish and the wild fish [15,16]. It is noteworthy that high dietary levels of n-3 PUFA not only increased percentages of n-3 PUFAs in liver lipids, but also increased the incidence of oxidative stress, characterized by reducing activity of β-oxidation capacity, together with elevated activities of superoxide dismutase (SOD) and caspase-3 [17]. The lipids β-oxidation in muscles is believed to be responsible for lipid accumulation, lower nutritional quality and modifying the texture and color of meat [18,19]. Moreover, dietary EPA supplementation has been reported to reduce fatty acid oxidation, which also facilitates the accumulation of EPA and decreases the total n-3/n-6 ratio [20].

It is widely accepted that long-term feeding of artificial diets likely contributes to the decline of fish flesh taste [21]. However, few studies have investigated the dietary effects on fish muscle metabolism and possible associations between changes in fish flesh quality characteristics and metabolic alterations. In this study, the effects of different diets, artificial feed (AF) and natural grass (GF), were investigated in *C. idellus.* Since sex can easily influence metabolomics data, metabolic profiles were separated by sex (FGF vs FAF; MGF vs MAF) in order to better evaluate the footprint of each diet on muscle quality. After a 113-day period of feeding, lipid mass in muscles and muscle fiber characteristics were examined, followed by a comprehensive untargeted metabolomic profiling of fish muscles using LC-MS. Finally, serum levels of total cholesterol (TCHO), high density cholesterol (HDLC), glucose (GLU), total protein (TP) and triglycerides (TG) were used to assess if physiological and biochemical indicators were consistent with metabolic changes. Significantly different metabolites and mass peaks between the experimental groups were further examined using pathway analysis to gain a better understanding of the effects of diets on metabolic alterations and muscle quality

in *C. idellus*.

Data Description

In this study, we conducted a comprehensive physiological, biochemical and metabolomic investigation of the effects of artificial and grass feeding on *C. idellus* flesh quality. After 113 days of separate feeding, muscle samples were collected from the two groups fish. At the same time, because of the metabolite sensitivity and sex specificity metabolomic analysis of muscle samples were divided into four test groups based on the results of the sex determination (n = 10), including female fish of the grass feeding group (FGF), male fish of the grass feeding group (MGF), female fish of the artificial diet group (FAF), as well as male fish of the artificial diet group (MAF).

All groups' samples were detected by Beijing Genomics Institute (BGI, Shenzhen, China) using LC-MS/MS. For qualitative and quantitative metabolomics, raw data were processed using Progenesis QI software (Nonlinear Dynamics, 2017, version: 2.2, Waters, MA, US). To verify and confirm compound identifications, the METLIN batch Metabolite Search Database, Kyoto Encyclopedia of Genes and Genomes, Human Metabolite Database and ChemSpider databases were used by comparing molecular weights and MOL files. The molecular and structural formulas of the candidate compounds were retrieved by the comparison and then confirmed by MS/MS scans for the characteristic ions and fragmentation patterns of the metabolites. The statistical analyses of detected features were performed by MetaboAnalyst 4.0 (http://www.metaboanalyst.ca/), using the "Statistical Analysis" module [27]. The input data were normalized by a pooled sample (quality control, QC) from the two experimental groups. The experimental design and analysis flowchart detailing these steps were shown in Figure 1.

Figure 1. The experimental design and flowchart.

Additionally, our metabolic datasets will contribute to future fish nutrition research, disease and immunization research, or optimization of breeding conditions for fish, even human dietary and nutrition studies. Our raw metabolomics data has been deposited to the EMBL-EBI MetaboLights database (DOI: 10.1093/nar/gks1004. PubMed PMID: 23109552) with the identifier MTBLS673. The complete dataset can be accessed here https://www.ebi.ac.uk/metabolights/MTBLS673. In addition, they can also be downloaded from Github.com (https://github.com/zhao253091640/HZAU-Prof.-Dapeng-Li-s-Laboratory). In addition, the preliminary list of compound identification and information regarding the significant differential metabolites (such as potential mapped metabolites, their query IDs, p.values, FC, FDR, and their corresponding metabolic pathways are provided in the Supplementary files. The Supplementary files are available via the Giga Science.

Analyses

Growth Performance

Table 1 showed the growth performances of the four test groups (FAF, FGF, MAF and MGF), respectively. Overall, after 113 days of separate feeding, different diets showed significant effects on different growth traits of the four test groups, regardless of the sex of *C. idellus*. The body mass, body length, body height, visceral weight, liver weight, and specific growth rate (SGR) of fish in both FAF and MAF were all significantly higher than those in GF groups ($P <$ 0.01). The most pronounced differences due to the artificial diet were increases in body weight (WG), visceral weight,

and liver weight ($P < 0.05$). The final weight of MAF fish was 38.55 % higher than that of MGF fish, and the obtained weight of FAF was 11.66 % greater than that of FGF. The visceral weights were about 1.5 times higher in AF groups, and the liver weights of AF were about 2-fold greater than that of GF fish. Furthermore, sex is an important factor in fish growth. Compared with MGF *C. idellus*, the WG was 37.44% higher in FGF, whereas in AF groups, the increased weight in female *C. idellus* was 10.73% greater than in male fish. Finally, the condition factor (CF) was the only physical indicator that was significant higher in GF ($P < 0.05$), despite the sex of the fish.

$$\textbf{SGR} = (\text{Ln}(W1) - \text{Ln}(W2))/ T * 100$$

$$\textbf{CF} = (W1/ L^3) * 100$$

Note: W1- Body Mass (g); W2- Visceral Weight (g); T- Feeding days; L- Body Length (cm)

Table 1. Growth data of *Ctenopharyngodon idellus* fed with different feeds.

Effect of Diet on Serum Biochemical Indexes and Abdominal Fat Accumulation

Table 2 shows the serum biochemical data of *C. idellus* in the two experimental groups. The comparisons between GF and AF indicated that different feeding diets resulted in significant differences in concentrations of several serum biochemical indicators ($P < 0.05$). HDLC was the only indicator that showed the least change between the different diets. The majority of the higher concentrations of serum biochemical indicators were found in GF fish, except for ALB and TG. The levels of ALB and TG were significant higher in AF ($P < 0.05$).

Table 2. Serum biochemical parameters in *C. idellus* farmed under two feeding models.

The changes of muscle fibers and intramuscular lipid droplet sizes in abdominal muscles were observed by HE and oil red O staining, respectively (Figure 2-A). The corresponding statistics were calculated and visualized in Figure 2-B. Compared with GF groups, the mass of lipid droplets were significantly increased in both MAF and FAF *C. idellus* ($P < 0.01$), which is largely attributed to elevated numbers of adipocytes in these two test groups and not an enlargement of the size of adipocytes. Moreover, the average diameter of muscle fibers was significantly higher in grass feeding groups ($P < 0.01$). Between the two sexes, the size of lipid droplets and the diameter of abdominal muscle fibers were both significantly higher in female fish, specifically FAF and FGF *C. idellus* ($P < 0.01$).

Figure 2. Histological sections of abdominal muscles of *C. idellus*.

Effect of Diets on Metabolomic Alterations of Muscle Samples

Muscle samples were collected after a 113-day breeding period and subjected to untargeted LC-MS metabolomics analysis. The two score plots of the PCA models show a clear separation of samples from different experimental groups and quality controls (Figure 3), indicating that feeding *C. idellus* with artificial feed or natural grass could induce significant changes in the muscle metabolomic profile in both sex of *C. idellus*, with male samples showed a slightly more clear separation between the two different diet groups. The corresponding loadings plots for PCA models were provided in Figure S1.

Figure 3. PCA score plots for the metabolomics profiles of *C. idellus* muscle samples.

Identification of Discriminating Features between Groups

The significant discriminating metabolites (SDMs) were identified based on the following criteria: fold-change (FC) threshold ≥ 2 (or < 0.5) and a FDR-adjusted p-value (q-value) < 0.05 using the volcano plot analysis (Figure 4). Based on the criteria, a total of 41 metabolites were significantly up-regulated in FAF, and 63 metabolites were significantly down-regulated in the same group (Figure 4-A). In MAF *C. idellus*, 45 metabolites were up-regulated and 75 metabolites were down-regulated (Figure 4-B). Furthermore, all the SDMs between the two experimental groups are respectively summarized in Table S1 and Table S2 along with their matched adducts, potential metabolites, their query IDs, p-values, FC, FDR, and their corresponding metabolic pathways.

Figure 4. Volcano plots for the potential metabolomic features of muscle samples from female (A) and male (B) *C. idellus*.

Additionally, the peak intensities of all the SDMs were normalized and log transformed before the Pearson's correlation method was used to identify correlations between SDMs that differed between AF groups and GF groups (Figure S2).

The Impacts of Different Diets on *C. idellus* Muscle Metabolisms

The relative peak intensities of 39 SDMs shared between female and male metabolic profiles are visualized as a heatmap in Figure 5. The correlation analysis of the SDMs (including overlapped and sex-specific SDMs) specifically related to lipids and carbohydrates metabolisms are shown in Supplementary files (Figure S2).

Compared with FGF *C. idellus*, the relative intensity of stearic acid (a saturated fatty acid) was significantly higher in FAF (FC > 2.0, and q-value close to zero). A number of UFAs discriminated between the FGF and FAF samples. In particular, DPA, adrenic acid, DGLA, ARA and LTE4 all showed significantly higher concentrations in the FAF *C. idellus* (q-value < 0.05), as did ten ARA metabolites with a similar structure: 15(S)-HETE, 5-HETE, 8-HETE, 9(S)-HETE, 16(R)-HETE, 19(S)-HETE, 8,9-epoxyeicosatrienoic acid, 11,12-epoxyeicosatrienoic acid, 5,6-epoxy-8,11,14-eicosatrienoic acid and 14,15-epoxy-5,8,11-eicosatrienoic acid. These metabolites were significantly higher in the FAF, and were all positively correlated with each other (Figure S2-A). In female grass-fed *C. idellus*, metabolites involved in lipids metabolism exhibited significantly higher levels (FC < 0.5, and q-value < 0.05), such as diacylglycerol, L-palmitoylcarnitine, LTA4, DHA, palmitic acid, PGG2, EPA, linoleic acid, 13S-hydroxyoctadecadienoic acid, GLA, stearidonic acid, and caprylic acid. These significantly up-regulated metabolites in FGF showed positive correlations with each other, and negative correlations with those up-regulated metabolites in FAF (Figure S2-A).

Compared with the results of female *C. idellus*, more SDMs were up-regulated in MGF fish. Particularly, among the 75 significantly up-regulated metabolites in MGF, a total of 26 were related to lipid metabolism. Further, the differential fatty acids, such as pelargonic acid, stearic acid and L-palmitoylcarnitine, displayed significantly higher intensities in MGF (FC < 0.5, and q-value < 0.05). Additionally, the remaining eight discriminatory metabolites between MAF and MGF were involved in UFAs metabolism, including viz, arachidic acid, EPA, LTB4, 13(S)-hydroxyoctadecadienoic acid, 15(S)-HETE, 5-HETE, 13(S)-HPOT, ALA and GLA, which all had significantly higher peak intensities in MGF (FC < 0.5, and q-value < 0.05). All the up-regulated metabolites in MGF *C. idellus* were all negatively correlated with those up-regulated metabolites in MAF (namely, adrenic acid, DHA, DPA, ARA and DGLA,

as well as three LTs) (FC > 2.0, and q-value < 0.05) (Figure S2-B).

Figure 5. The significant metabolites shared between the four test groups.

Feeding *C. idellus* with different diets also affects carbohydrate metabolism. FGF significantly increased the intensity of metabolites related to glycometabolism (mannan, globoside, UDP-glucose, UDP-galactose, starch, Tn-antigen and protein C-terminal S-farnesyl-L-cysteine methyl ester) (q-value < 0.05). A number of physiologically important metabolites are very different between FGF and MAF groups, including geranylgeranyl pyrophosphate, dolichyl diphosphate, dolichyl phosphate, 9-cis-retinoic acid, diacylglycerol, 5-L-glutamyl-L-alanine, alpha-tocopherol and PIP2. These metabolites were all up-regulated in FGF muscle samples (FC < 0.5). The down-regulated metabolites in FGF were heparan sulfate, 3-Phosphatidyl-ethanolamine, UPD-glucuronic acid, GD2, 1-Acylglycerophosphoinositol, flavin mononucleotide and sialyl-Tn antigen (FC > 2, q-value < 0.05). They showed significant positive correlations with each other, and negative correlations with metabolites up-regulated in FGF (Figure S2-A).

In male *C. idellus* samples, alpha-tocopherol, globoside, starch and protein C-terminal S-farnesyl-L-cysteine methyl ester, 5-L-glutamyl-L-alanine, 9-cis-retinoic acid, dolichyl diphosphate and dolichyl phosphate were showed significantly higher levels in MGF compared to MAF. In addition, GD1a, L-amino acid, PIP3, dihydroxyacetone phosphate, trypanothione disulfide and UDP-N-acetyl-D-glucosamine also displayed higher intensities in the MGF muscles. Down-regulated metabolites in MGF were quite different from the SDMs identified in FGF. Besides GD2 and heparan sulfate, UDP-D-Xylose, GD1b, 1-Phosphatidyl-D-myo-inositol, D-Glucosaminide, inositol phosphate and trypanothione also exhibited lower intensities in MGF samples. (Gal)2(GlcA)1(Xyl)1(Ser)1 and naphthyl-2-oxomethyl-succinyl-CoA also showed lower intensities in the MGF samples.

Pathway Enrichment Analysis

Using *D. rerio* as reference library, the pathway impact and enrichment analysis of the significantly different metabolites, as well as the network and the physiological properties of the matched compounds of female and male *C. idellus* samples are separately shown in Figure 6 (female *C. idellus* metabolic profile) and supplementary material Figure S3 (male *C. idellus* metabolic profile). Regardless of the sex of *C. idellus*, most of the discriminating metabolites between AF and GF groups were largely concentrated in fatty acid and unsaturated fatty acid metabolism (FA and UFA metabolism), steroid hormone metabolism, vitamins metabolism and amino acids metabolism, as well as glycometabolism pathways.

The pathway impact and enrichment analysis of the significantly different metabolites ($P < 0.05$) in both female and male *C. idellus* samples were also conducted using MetaboAnalyst 4.0 - "Enrichment Analysis" and "Pathway Analysis" modules. Generally, most of the enriched pathways were the same for female and male *C. idellus*. The differential metabolites were significantly enriched in steroid hormone biosynthesis (as above), carbon pool by folate, arachidonic acid and lenoleic acid metabolisms, as well as primary bile acid biosynthesis pathways, in both female and male metabolic profiles ($P < 0.05$) (Figure 6-A and supplied Figure S3-A). These significantly altered pathways also had high impact values (Impact-values > 0.75). However, the differences between the two genders were starch and sucrose metabolism, as well as galactose metabolism, which showed significant enrichment in female *C. idellus* but not in male *C. idellus*. ($P < 0.05$) (Figure 6-A and Figure S3-A). The significantly different metabolites in male *C. idellus* were also significantly enriched in retinol metabolism and steroid biosynthesis pathways ($P < 0.05$), which showed no

significance in female results.

Figure 6. The pathway enrichment and network analyses for the significant metabolites in female *C. idellus*. **(A)** The scatter plot showing the pathway impact and enrichment results for all matching significant metabolites in female *C. idellus*; **(B)** The KEGG global metabolic network visualization of all significant metabolites ($P < 0.05$) in the female *C. idellus* metabolic profile.

All significant metabolites appeared in both female and male experimental groups and their corresponding metabolic pathways are listed in Table 3 (female) and Table 4 (male). Meanwhile, they are all highlighted in red and annotated with KEGG IDs in KEGG global metabolic map of the results of the pathway enrichment analysis (Supplementary file Figure S4). In addition to the biomarkers between AF and GF groups, many sex-specific metabolites were found between genders, such as estriol (C05141), 2-hydroxyestradiol (C05301), estradiol (C00951), 4-hydroxyretinoic acid (C16677), 5,6-epoxyretinoic acid (C16680) and 11-cis-retinyl palmitate (C03455). However, all significant metabolites enriched in one carbon pool by folate, primary bile acid metabolism, arachidonic acid and linoleic acid metabolisms pathways were similar in both female and male *C. idellus* metabolic profiles.

Table 3. Pathway impact and overlapped metabolites analysis of female *C. idellus*.

Table 4. Pathway impact and overlapped metabolites analysis of male *C. idellus*.

To verify the pathway analysis result based on manual annotation, we also used the new “MS Peaks to Pathways” (Mummichog) module in MetaboAnalyst 4.0 to predict pathway activities (*Danio rerio* pathway library) directly from the 1033 significantly differential peaks detected in the female *C. idellus* samples, and 1495 peaks in the male samples (q-value < 0.05) (data not shown). In total, 5 significant enriched pathways were identified in the female *C. idellus* metabolomic profiles, including steroid hormone biosynthesis, arachidonic acid metabolism, retinol metabolism, steroid biosynthesis and biosysnthesis of unsaturated fatty acids (q-value < 0.05). On the other hand, 6 pathways were significantly enriched by male *C. idellus* 1495 mapped and significant differential features, sucn as steroid hormone biosynthesis, steroid biosynthesis, arachiconic acid metabolism and retinol metabolism, as well as glycerophospholipid metabolism and sphingolipid metabolism (q-value < 0.05). All the enriched metabolic pathways in male and female profiles, are all responsible for lipids and vitamin metabolisms.

Discussion

Differences in Growth Performances between Groups

In this study, we found significantly different growth performance between the artificial feed and natural grass feed groups. This observation is consistent with the previous study, whereby feeding *C. idellus* with natural grass resulted in significantly lower weight gain (WG) in both FGF and MGF groups [21,30]. The negative correlation between enhanced dietary fibers and low-fat diet with reduced WG was also demonstrated in *Barbodes altus* and *Oncorhynchus mykiss* [31,32]. Although different diets changed the growth traits of *C. idellus*, the dietary effect on fish growth seemed to be sex independent. For instance, significantly lower WG was observed in male fish. This is contrary to the result in a study

on mice fed a low-fat diet, in which female showed less WG [33]. Further research is warranted to investigate whether different diets have sex-dependent effects on growth traits in different species.

Fat Deposition Induced by Artificial Feed Feeding and Improvement of Feeding Grass

The significant increase in fat mass, as detected in the abdominal muscles of both FAF and MAF groups, was caused by an increased number of adipocyte cells, not an increase of the size of adipocytes. This is in agreement with the conclusion of a previously published *Oreochromis niloticus* study [34]. Proliferation of adipocytes is likely the main strategy of fish responding to the intake of high fat and protein diets. Significantly smaller diameters of muscle fiber were found in AFs muscle samples, which together with increased fat contents, would influence the texture and taste of the fish flesh [35]. A common misconception is that high-protein diets inevitably raise cholesterol levels in serum. In contrast, this study found that significantly higher concentration of cholesterol was detected in GF group compared to AF group. The moderate and good cholesterol could give structure to cell walls and produce certain hormones [47]. However, higher levels of triglyceride (TG) were found in the *C. idellus* fed with artificial diet. All the findings above demonstrate that a sustained high-fat and protein diet had clear deleterious effects on the fish, characterized by an increased serum TG, fat accumulation in organs (such as viscera and muscle), and decreased stress tolerance [20].

Changes in Lipid Metabolism in Fish Flesh induced by Different Diets

It is well known that chronic consumption of high dietary fat and protein can disrupt lipid homeostasis, thereby leading to steatosis and fat deposition [36,37]. Few studies however, have focused on linking physiological measures to functions at the metabolic level. One study has described the effect of low- and high-fat diet on the metabolism in *O niloticus* [30], but the influence of different diets on muscle characteristics and lipid metabolism, as well as correlations between them, has only been investigated in mammals [12,18,38]. Nevertheless, the assessment of dietary intake in these studies is subjective as there are no definite standards for measuring the nutritional status of a diet, nor the experimental subject [39]. On the other hand, ingestion of different diets at various doses can have highly divergent effects [31,40]; different tissues can have very different metabolic patterns [41,42]. Although metabolomic studies of the effect of diets in different animal studies may not be directly comparable, they can serve as references for further research. In our study, a large amount of metabolites were detected and differentially enriched in energy metabolisms between the two feeding groups, indicating that metabolic alteration is the main mechanism in which fish respond to different feeding patterns. Furthermore, the potential associations between physiological changes (the muscle characteristics, together with nutrients and textures measured in our previously published studies) [21,30] and metabolic profiles in different feeding conditions will be systematically further examined below.

In general, flesh quality of cultured animals is primarily attributed to nutritional condition in diets. Higher nutritional levels in diets could result in increased levels of saturated fatty acids (SFA) and decreased levels of polyunsaturated fatty acids (PUFA) [43]. In the present study, the proportions of SFA in GF groups' muscle samples were markedly higher, while PUFA levels were almost equal to their intensities in muscle samples of AF groups (including both FAF and MAF). The same findings have been proved in our previously published study [30]. Nevertheless, this conclusion is not absolute, different animals can give rise to distinct results. For instance, the opposite result was obtained in a lamb study, with increased PUFA and decreased SFA contents found in the natural diet group rather than the artificial diet group [38]. In addition to species diversity, different sources of plant protein between the

two studies may have also contributed to contrasting results.

As reported in other research, SFAs (e.g. C16:0 and C18:0) play important roles in influencing flesh texture, with higher levels resulting in a “crisper” flesh taste [30,44]. In line with the previous findings, the increased arachidic acid (20:0), stearic acid (18:0) and palmitic acid (16:0) were detected in our GF groups, therefore the flesh of GF *C. idellus* would be harder than that of AF fish. This conclusion is consistent and proved by our previously published test results of textures, shear force detection and fatty acid contents measurement of the two feeding groups *C. idellus* flesh [21, 30]. Previous research has also shown that higher arachidic acid can interfere with essential fatty acid metabolism by inhibiting Δ-6 desaturase enzyme, which reduces the formations of DGLA (20:3n-6) and ARA (20:4n-6) [45]. Thus significantly lower levels of DGLA and ARA were detected in FGF and MGF. Another SFA, palmitic acid (16:0), its significant higher levels were also found in GF fish. This is consistent with previous research, where it was higher in the GF *C. idellus*, which in turn were thinner than the other feeding group fish [46]. Similiarily in our study, the fat mass of muscle tissues of grass feeding *C. idellus* was significant lower than those of FAF and MAF muscle tissues. Additionally, GF increases the levels of several medium-chain fatty acids (e.g. caprylic acid and pelargonic acid), which are known to contribute to better flesh flavor and odor, help reduce abdominal fat, and improve cholesterol levels [47].

Several n-3 PUFAs, such as EPA (20:5n-3), DHA (22:6n-3) and their important intermediaries, as well as ALA (18:3n-3) and stearidonic acid (18:4n-3), were significantly different between grass-fed and artificial-fed *C. idellus* muscle samples. They are all the most bioactive of n-3 PUFAs and are known to be beneficial for human health [11]. The significantly higher levels of EPA were found in MGF and FGF *C. idellus*, which could be derived from higher levels of ALA in the same groups [48], or originate from grass rich in n-3 PUFAs. Although the same finding has not been demonstrated in other fish studies, a similar conclusion was obtained in a cow study, where significantly higher levels of n-3 PUFAs were found in grass fed cows [13]. Because of the importantly physiological significances of higher EPA to mammals [11,49], the accumulation of EPA in organisms is a hot topic research, and could be facilitated by the reduced mitochondrial FAs β-oxidation [17,20]. The DHA and DPA were markedly up-regulated in AF groups, though are known to be rich components in animal artificial feeds [50], so their higher levels may be directly taken from artificial feeds. On the other hand, higher DPA in FAF and MAF muscles could be attributed to aggregate data with different types of DPA (n-3/ n-6). Overall, the higher DPA and DHA contents in *C. idellus* fed with artificial feed is consistent with the previous statement, they indirectly reflect the corresponding higher contents in the artificial feed used in our study. However, the main fatty acid component in green plants is generally ALA, meanwhile they have a much higher proportion of n-3 PUFAs compared to n-6 PUFAs [40]. Consequently, feeding fish with natural grass caused remarkably higher ALA levels in FGF and MGF than AF *C. idellus*. Another reasonable explanation for the higher ALA in GF groups is that ALA is a substrate for endogenous formation of EPA [49], therefore the higher ALA positively correlating with the higher observations of EPA in the same test groups makes sense. In summary, *C. idellus* fed with grass is comparable to their wild counterparts, characterized by higher ALA, EPA and lower DHA levels in their muscle tissues [16]. Grass-fed farmed *C. idellus* would be more attractive to consumers for the reason that intake of EPA has been recommended as a promising novel therapy to decrease hepatic triglyceride content [20]. Another note-worthy group of significantly discriminating metabolites between the two feeding groups are the n-6 PUFA family. Contrary to the higher proportions of DGLA and ARA in both FAF and MAF, the levels of GLA (18:3n-6) and LA (18:2n-6) were significantly higher in FGF fish and were only altered in female samples. Their relative intensities in the metabolic profiles of *C. idellus* muscle tissues were sex-specific. An earlier study of *Salmo salar* has suggested that the enhanced LA has no effects on fish growth, but could result in decreased lipid content in muscle tissues [42]. Accordingly, the strong negative

correlation between the content of LA and fat deposition in muscle tissues was also examined in our study. Moreover, this physiological function of LA is not limited in animal models, as LA plays more roles in human health as follows: slightly decreasing abdominal fat accumulation, as well as protection against death from coronary heart disease and cardiovascular disease [51,52]. However, another study demonstrated that the nutritious value of ALA for fish products was higher than that of LA. Specifically, prawns fed with supplied ALA diets obtained significantly higher weight gain than those fed with diets containing an abundance of LA, meanwhile, elevated proportions of n-3 PUFAs were also measured in the ALA-feeding groups [53]. Simply, the higher intensities of both ALA and LA were all investigated in GF fish. Following this, the fat deposition in visceral and muscle tissues could be reduced, and the percentages of n-3 series PUFAs could be improved in humans by increasing their consumption of grass-fed *C. idellus*. In summary, the GLA, LA, ALA and EPA were all significantly up-regulated in GF groups, illustrating that feeding *C. idellus* with grass could improve the nutritional value of the fish flesh. Furthermore, these active ingredients of PUFAs are responsible for lowering triglyceride levels not only in animals, also in human [11,20,42].

Another functionally important set of metabolites that were significantly altered between AFs and GFs fish are eicosanoids, which are known as the products of enzymatic oxidation of ARA. In humans, n-3 PUFAs together with eicosanoids, are engaged in various physiological processes and are essential for normal growth and development [54]. They also play an important role in the prevention of cardiovascular and inflammatory diseases, and have a promising impact on the prevention of cognitive decline and dementia in older people [52,54-56]. Though eicosanoids are ubiquitous in various tissues, their precise physiological roles have not been well defined in animals. In the current study, the different feeding patterns resulted in a significant difference in the concentrations of eicosanoids between GFs and AFs, and were vastly different between female and male metabolic profiles. This meant that metabolic differences between experimental groups were also sex-dependent [57]. The details are as follows: LTA4, LTC5, LTE4 and PGG2 were significantly different between FAF and MAF, whereas the differential metabolites between FGF and MGF were LTA4, LTB4 and LTF4. Furthermore, the markedly higher levels of PGG2, LTA4, LTB4 and LTC5 were all measured in GF groups. Accordingly, feeding *C. idellus* a grass diet could not result in better quality and higher nutritious fish products, but also provide higher levels of eicosanoids for consumers's health [52,54-56]. However, there is another evidence showed that PGs and LTs are separately generated by the enzymatic action of cyclooxygenases (COX) and 5-lipoxygenase (5-LO), both of which are well characterized lipid mediators involved in host defense and inflammatory responses [41]. Therefore, the fish fed with grass might be in a state of stress. More importantly, although the anti-inflammatory effects of eicosanoids are well known, their side effects of long-term overuse has been investigated, associated with excessive inflammation, thrombotic tendencies, atherosclerosis, and immune suppression, as well as gastrointestinal complications(e.g. ulceration) and obesity in humans [9,58,59]. Due to no definite standard range of eicosanoids content at present, the specific experiments on various doses of eicosanoids and their corresponding physiological functions are urgently needed [39].

Changes in Carbohydrates Metabolism in Fish Muscles induced by different Diets

In addition to lipids metabolism, the energy requirement and fat deposition in muscle tissues is also closely related to carbohydrate metabolism, as muscle tissue are a major site of glucose disposal, accounting for approximately 30% of postprandial glucose disposal [60,61]. In our study, several metabolites involved in carbohydrate metabolism and activities were greatly increased in FGF and MGF muscle samples including mannan - a prebiotic in animal husbandry and nutritional supplements, UDP-glucose - an activated form of glucose, UDP-galactose and amylopectin (the glycogen

in animal), as well as Tn-antigen, which were all up-regulated in FGF muscle samples [62]. Furthermore, increased level of diacylglycerol in FGF has been shown to suppress the fat accumulation in fish [63,64]. Geranylgeranyl pyrophosphate in plants, the precursor to carotenoids and tocopherols that will be used to synthetize geranylgeranylated proteins and cholesterol in Perciformes and Salmonidae fish after being consumer [59,65]. Moreover, α-Tocopherol (Vitamin E) could be preferentially absorbed and accumulated in humans, and has been associated with an enhanced prevention of natural abortions in pregnant women [66]. In summary, feeding *C. idellus* different diets resulted in markedly different metabolic functions, particularly changes in fatty acid metabolism and glucose metabolism [14,61]. Additionally, we demonstrated that feeding *C. idellus* grass could also improve the contents of physiological active substances in fish muscles, such as those involved in vitamins, amino acids and steroid hormone metabolism pathways. Theses beneficial metabolites could be then absorbed and accumulated after consumption by humans, and potentially improve physiological functions in the human. Feeding *C. idellus* with natural grass can affect the activities of enzymes involved in lipid and carbohydrate metabolism (e.g. Acetyl-CoA, glucose-6-phosphate dehydrogenase), modulate the production of metabolites, decrease fat accumulation, as well as increase fatty acid β-oxidation capacity in muscle tissues, similar to what has been observed in *S. salar* [17]. In addition, feeding with grass could effectively improve the fatty acid compositions and ratio (n-3/n-6) due to the increasing usage of n-6 PUFA-rich ingredients in aquaculture diets [21]. Notably, the higher proportions of n-6 PUFAs in grass-fed *C. idellus* flesh could prevent cardiovascular and inflammatory diseases in humans, as well higher n-3 PUFAs also play important roles in promoting growth and development, decreasing hepatic triglyceride content, reducing fat accumulation and so on [20,67]. Future studies are necessary in order to determine the optimal doses of n-3 PUFA to fish feeds that can improve the concentrations of healthy PUFAs (e.g. ALA, EPA, DPA, and DHA) in fish products, as well as to understand if these beneficial effects can be translated to mammals. These studies will be significant steps towards the goal of meeting consumers' demand for high quality, safe and healthy aquatic products [3].

Conclusion

In this study, we have conducted a comprehensive physiological, biochemical and metabolomic investigation of the effects of artificial and grass diet feeding in *C. idellus*, and linked these results to specific parameters (e.g. fat accumulation status, muscle fibre thickness and texture) of flesh quality. It is clear that flesh quality parameters and metabolomic factors are deeply intertwined. The flesh quality-specific differences at the metabolic level were not only related to fat accumulation in *vivo*, but also affected the final flavor through direct influences on the lipid and carbohydrate metabolism in muscles of *C. idellus*. Moreover, from both environmental and nutritional perspectives, natural grass is a better source of dietary FA and protein when compared to conventional artificial fish feed. This is because grass is more efficiently absorbed and converted into beneficial PUFAs and other nutrients, thereby obtaining higher quality fish products. Particularly, elevated EPA, ALA, stearidonic acid and some n-3 eicosanoids in muscles of FGF and MGF may improve the ratio of n-3/n-6 PUFA in fish flesh, thus decreasing the risk of certain diseases [10,68,69]. In addition, the higher levels of mannan, starch, UDP-glucose, UDP-galactose and dihydroxyacetone phosphate, as well as other metabolites involved in carbohydrate metabolism, are reflective of an increased glycometabolism activity in the muscle tissues of *C. idellus* fed with grass. It is evident that the *C. idellus* fed with *L. perenne*, *E. pectinata* and *S. sudanense* results in fish with a higher quality and healthier life than those fed with artificial feeds. However, from commercial point of view, we still need to maintain the faster growths and higher yields of *C. idellus* contributed by artificial feeds. Based on the above considerations, we propose that feeding fish with both artificial

feed and natural grass in a suitable proportion will produce healthy, fast growing and high yield aquatic products. Further experiments are required to verify and to refine the feed in order to achieve optimal growth and health.

Materials and Methods

Animals and Diets

The fish used in this study were cultured in the basement of the Chonghu Fish Farm, in the Hubei Province of China. All fish originated from the same batch of *C. idellus* fingerling, with an initial average weight of 35 g per tail. This study was designed to investigate metabolic alterations in response to different diets. Therefore, fish in one group were fed with natural grass (GF), which included *Lolium perenne*, *Euphrasia pectinata* and *Sorghum sudanense*. Fish in the other group, the artificial diet group (AF), were fed an artificial diet. The percentages of various nutritional compositions of the two diets are presented in Table 5.

Table 5. Percentages of nutrients in the different two feed.

Experimental Procedures

At the beginning of the experiment, about 3000 tails of grass carp were assigned to each pond (roughly 22666.67 m^2 per pond), which was co-housed with 550 tails of *Hypophthalmichthys molitrix* (average weight of 14 g per tail) and 350 tails of *Aristichthys nobilis* (average weight of 25 g per tail). Three replicate ponds were used in each experimental group. The feeding experiment spanned from July 8, 2016 to October 28, 2016. During the experimental period, GF fish were fed 100 kg of *L. perenne*, *E. pectinata* and *S. Sudanense* for each pond per day, whereas 15 kg of artificial diet was supplied to each AG pond two times per day. At the end of the rearing experiment, the fish samples were collected directly at the fish farm. About 85 tails of *C. idellus* were caught from each pond. Meanwhile, the total number of fish from each experimental group was 250.

This study complied with the Animal Research: Reporting of In Vivo Experiments (ARRIVE) guidelines and “Guidelines for Experimental Animals” from the Ministry of Science and Technology (Beijing, China). Further, the Institutional Animal Care and Use Ethics Committee of Huazhong Agricultural University had approved our study. All efforts were made to minimize the suffering of sampled fish species.

Sample Collection

Prior to sample collection, *C. idellus* were anesthetized by 100 mg·L^{-1} MS-222 (Sigma, St. Louis, Missouri, USA) for 2-4 minutes, then the growth performances were measured for each fish. From 10 tails of fish randomly chosen per experimental group, blood samples (180 - 200 mL per tail) were taken from caudal vein without an anti-coagulating substance by injector puncture. The blood samples were placed at room temperature for 30 minutes and then centrifuged at 3000 g for 30 minutes at room temperature for serum preparation. The separated serum was stored at -80 ℃ until the serum biochemical indexes analysis.

White muscle (including those used for both metabolic detections and histological sections) and gonadal tissues were taken from 250 tails per experimental group. The back and abdominal muscle samples were immediately harvested and frozen in liquid nitrogen. Muscle samples were transferred and preserved at -80 ℃ until Oil red O staining and

subsequent metabolomics analyses. Gonadal tissues and partial abdominal muscle tissues were also collected and kept in Bouin's fixative (saturated solution of picric acid (75 ml), 40% aqueous formaldehyde (25 ml), and glacial acetic acid (5 ml)) at room temperature. Serial transverse 10 μm-thick sections of abdominal muscles and gonads were stained routinely with hematoxylin & eosin (H&E). The sex of the grass carp was determined by the contour of the gonad and further results of the gonad tissue slice [22,23]. To determine the presence of fat in the muscles, frozen muscle tissues were stained with Oil red O solution, which would color any fat contained in the muscle.

Based on the results of the sex determination, metabolomic analysis of muscle samples were divided into four test groups (n = 10, each repetition mixed by 5 individuals randomly selected from each group): female fish of the grass feeding group (FGF), male fish of the grass feeding group (MGF), female fish of the artificial diet group (FAF), as well as male fish of the artificial diet group (MAF).

Serum biochemical assay

Serum samples (n = 10) were prepared according to a previously published method [24]. The lactate dehydrogenase (LD), glutamic-oxalacetic tansaminase (AST), glutamic-pyruvic transaminase (ALT), alkaline phosphatase (ALP), total cholesterol (TCHO), high density cholesterol (HDLC), glucose (GLU), albumin (ALB), total protein (TP) and triglycerides (TG) were measured by automatic biochemistry analyzer (Hitachi 7020, Hitachi High Technologies, Inc., Ibaraki, Japan). Test kits were purchased from the Nanjing Jiancheng Biochemical Corporation (Nanjing Jiancheng Biochemical Corporation, Nanjing, China), and the entire procedure was performed in accordance with the kit instructions.

Histological Observation and Analysis

Serial transverse 10 μm-thick sections of muscle tissues were stained with H&E, and intracytoplasmic lipids with oil red O (Oil O staining) according to previously published procedures, respectively [25]. Please note that the samples were selected from the same cohort for metabolome detection, the corresponding sample numbers were same with samples used for metabolic tests (n = 50). A total of 200 - 400 fibers of white muscle per fish were studied using a Leica MZ 6 microscope for their cross sectional area (CSA), and the diameter (d= 2r) of each fiber was calculated from the fiber area (A) ($A = \pi \cdot r^2$), thus, $d = 2*\sqrt{(A \cdot \pi^{-1})}$). A size limit for identifying fibers was set at fiber diameters ≥ 10 μm as the optical resolution below this limit did not allow for sufficient identification and accuracy in the analyses [26]. The circularity of each fiber was also determined. The free software Image J (http://rsb.info.nih.gov/ij/) was used for quantitative statistics and analyses.

Sample Preparation for LC-MS

The metabolomic analysis of muscle samples were carried out on four test groups (n = 10). Frozen-white muscle samples were thawed slowly, where they were taken from the ultra-cold freezer (-80 ℃) and placed at -20 ℃ for 30 minutes, then put on ice until the samples were completely melted. Each repetition from each experimental group was taken from 5 individuals (approximately 25 mg per individual). Samples of five tails were placed in an EP tube and mixed with 800 μL of an ice-cold mixture of methanol and water (1:1 ratio), with two steel balls added to each tube. The tissues were then broken at 60 Hz for 5 minutes by the TissueLyser, then 300 μL of supernatant from each tube was collected after a 10-minute centrifugation at 25000 g at 4 ℃ and then injected into the LC-MS system. Ten microliters

of each sample was combined into a new vial and used as a pool sample for quality control and analyte identification, and were acquired after every 10 tested samples.

Chromatography and Mass Spectrometry Conditions

Chromatographic separations were performed using ultra performance liquid chromatography (UPLC) system Ultimate 2777C (Waters, UK). An ACQUITY UPLC BEH C18 column (100 mm* 2.1mm, 1.7 µm, Waters, UK) was used for the reversed phase separation. The column oven was maintained at 50□C. The injection volume for each sample was 10 µL, and the flow rate was 0.4 ml per minute. Additionally, the mobile phase consisted of solvent A (water + 0.1% formic acid) and solvent B (acetonitrile + 0.1% formic acid). Gradient elution conditions were set as follows: 0~2 min, 100% phase A; 2~11 min, 0% to 100% B; 11~13 min, 100% B; and 13~15 min, 0% to 100% A.

The eluents were introduced into a high-resolution MS/MS spectrometer Xevo G2-XS QTOF (Waters, UK) by electrospray ionization with capillary voltages set in the positive and negative modes to 2.0 kV and 1.0 kV, respectively. The cone voltages of both modes were 40V. The mass spectrometry data were acquired in Centroid MSE mode. The TOF mass scan range of both simultaneous low- and high-energy mass scan functions was from 50 m/z to 1200 m/z with a scan time 0.2 seconds. For the MS/MS detection, all precursors were fragmented using 20 - 40 eV. During the acquisition, the mass spectrometry signal was acquired every 3 seconds to calibrate the mass accuracy.

Data Processing and Metabolite Identification

For qualitative and quantitative metabolomics, raw data were processed using Progenesis QI software (Nonlinear Dynamics, 2017, version: 2.2, Waters, MA, US). First, data were cropped to remove external standards. Masses were detected, and the chromatogram for each mass was built using the Centroid mass detector and Chromatogram builder, respectively. Smoothed data were then deconvoluted using a noise amplitude algorithm and deisotoped. The conditions for chromatographic alignment were 0.01 m/z tolerance and 0.1 min RT-tolerance. Finally, sodium and ammonium adducts search was performed prior to exporting the data to Excel for post-processing. The compound identification list, which contained the molecular weight, compound name, statistical scores, and other information to show the result of the identifications was exported as an excel file (.csv).

To verify and confirm compound identifications, the METLIN batch Metabolite Search Database (http://metlin.scripps.edu/), Kyoto Encyclopedia of Genes and Genomes (http://www.genome.jp/kegg/), Human Metabolite Database (http://www.hmdb.ca/) and ChemSpider (http://www.chemspider.com/) databases were used by comparing molecular weights and MOL files. The molecular and structural formulas of the candidate compounds were retrieved by the comparison and then confirmed by MS/MS scans for the characteristic ions and fragmentation patterns of the metabolites.

Statistical Analyses

The peak intensity tables of detected features were inputted into the MetaboAnalyst 4.0 (http://www.metaboanalyst.ca/) “Statistical Analysis” module for univariate and multivariate data analysis [27]. The input data were normalized by a pooled sample (quality control, QC) from the two experimental groups. The log transformation ~~and autoscaling~~ were also used in data normalization procedures. Univariate data analysis was applied

to the metabolomics data using the Student's t-test. Statistical significance was set at $P < 0.05$ and $0.05 < P < 0.10$ as trends. Multiple testing corrections were performed based on false discovery rate (FDR)-adjusted p-values (q-values) with a significance threshold set at q-value < 0.05 [28]. For multivariate analysis, the data were subject to principle component analysis (PCA) for pattern discovery. For clustering analysis, a heatmap was created based on log-transformed relative intensities of detected features.

Pathway analysis was performed using the "Pathway Analysis" module, using the list of compound names manually annotated based on the significant peaks. To further validate the result, as well as to adjust for potential bias, we also applied the recent "MS Peaks to Pathways" module (mummichog) of MetaboAnalyst using the entire list of MS peaks [29]. The p-value cut-off for the MS Peaks to Pathways module was 0.05, and we used the *Danio rerio* as the reference library. The R-command history file generated throughout our analyses on MetaboAnalyst is available in the supplementary materials ("Female-MetaboAnalyst-Rhistory.R" and "Male-MetaboAnalyst-Rhistory.R", respectively).

Availability of data materials

Project name: Metabolic Alterations Induced by Different Diets

Project home page: https://github.com/zhao253091640/HZAU-Prof.-Dapeng-Li-s-Laboratory

Operating system(s): platform independent

Programming language: R

License: GNU General Public License version 2.0 (GPLv2).

Any restrictions to use by non-academics: none

Availability of supporting data

Our metabolomics raw data has also been deposited to the EMBL-EBI MetaboLights database (DOI: 10.1093/nar/gks1004. PubMed PMID: 23109552) with the identifier MTBLS673. The complete dataset can be accessed here https://www.ebi.ac.uk/metabolights/MTBLS673. In addition, the preliminary list of compound identification and informations of significant differential metabolites (such as potential mapped metabolites, their query IDs, p.value, FC, FDR, and their corresponding metabolic pathways were proved in Supplementary materials. The data further supporting this work are available in the GigaScience repository, GigaDB [70].

Abbreviations

QC: quality control; FAF: female artificial feed; FGF: female grass feed; MAF: male artificial feed; MGF: male grass feed; LC-MS: liquid chromatography-mass spectrometry; DPA: docosapentaenoic acid; DG-LA: dihomo-gamma-linolenic acid; ARA: arachidonic acid; FA: fatty acid; SFAs: saturated fatty acids; n-3 UFAs: n-3 unsaturated fatty acids; n-6 UFAs: n-6 unsaturated fatty acids; EPA: eicosapentaenoic acid; ALA: alpha-linolenic acid; GLA: gamma-linolenic acid; DHA: docosahexaenoic acid; PUFAs: polyunsaturated fatty acids; SOD: superoxide dismutase; AST: tansaminase; ALT: glutamic-pyruvic transaminase; ALP: alkaline phosphatase; TCHO: total cholesterol; HDLC: high density

cholesterol; GLU: glucose; ALB: albumin; TP: total protein; TG: triglycerides; ARRIVE: Animal Research: Reporting of In Vivo Experiments; H&E: hematoxylin & eosin; CSA: cross sectional area; KEGG: Kyoto Encyclopedia of Genes and Genomes; HMDB: Human Metabolite Database; FDR: false discovery rate; FC: fold-change; PCA: principle component analysis; SGR: specific growth rate; WG: body weight gain; CF: condition factor; SDMs: significant discriminating metabolites; LTs: leukotrienes; PGs: Prostaglandins; UDP: Uridine diphosphate galactose; PIP3: Phosphatidylinositol triphosphate; COX: cyclooxygenases; 5-LO: 5-lipoxygenase.

Competing interests

The authors declare that they have no competing interests.

Funding

This work was supported by the Earmarked Fund for China Agriculture Research System (CARS-45), National Natural Science Foundation of China (project number: 31502140), and the Fundamental Research Funds for the Central Universities (2662015PY119). The author Honghao Zhao is supported by the China Scholarship Council, which supports her study at McGill University (CSC No. 201706760039).

Supplementary data

Supplementary data are available at GIGSCI online, the contents are as follows:

Supplementary Figure S1: The PCA loading plots for the metabolomic data of muscle samples from female (A) and male (B) *C. idellus*.

Supplementary Table S1: List of Discriminating Metabolites between Female-Artificial feed feeding group (FAF) and Female-Grass feeding group (FAF) *C. idellus.*

Supplementary Table S2: List of Discriminating Muscle Metabolites between MAG and MGG *C. idellus*. The intensity of the most abundant metabolites in females, and the intensity of the metabolites were "normalized". Putatively identified using KEGG and HMDB.

Supplementary Figure S2: The Pearson's correlation analyses for the discriminating metabolites of lipids and carbohydrates metabolisms in muscle tissues of female (A) and male (B) *C. idellus*, respectively. The differential signatures were annotated with their potential metabolite names after mapping with compound databases. The diversity of color referred to the pair-wise correlation coefficient ranging from 1 (red) to -1 (blue).

Supplementary Figure S3: The pathway enrichment and network analyses for the significant metabolites in male *C. idellus*. (A) The scatter plot was used to visualize the pathway impact and enrichment results for all matching significant metabolites in male *C. idellus*; (B) The KEGG global metabolic network visualization of all significant metabolites ($P < 0.05$) in the male *C. idellus* metabolic profile. The colored points represent different metabolic pathways. The various color levels indicate different levels of significance of metabolic pathways from low (white) to high (red). The different

sizes of each point were used to represent the number of metabolites participated in the metabolic pathway. The greater rich factor, the greater the degree of pathway enrichment. Moreover the corresponding pathway's name of each point is labeled. In the metabolic network, all up-regulated metabolites (FC AF/GF > 2) in AF groups were colored with red, whereas the down regulated metabolites (FC < 0.5) were colored in green. In addition, the different color circles represent the various physiological functions that the discriminating metabolites belong to. Moreover, each enriched pathways is annotated with the corresponding name.

Supplementary Figure S4: Visualization of overlapped significant metabolites onto corresponding pathways. (A) The overlapped metabolites in female *C. idellus* highlighted in significantly enriched pathways; (B) The pathway view for the altered metabolites between MAF and MGF. Light blue compounds in the figures mean that these metabolites were undetected in our data, but used as background for pathway enrichment analysis. Red colored compounds mean the metabolites were detected in our metabolomic data and involved in the specific metabolism pathway.

Author contributions

Honghao had roles in study design, culturing fish, collecting samples, data collection and analysis. The manuscript was written through contributions of Honghao Zhao, Jianguo Xia and Dapeng Li. Jasmine Chong did valuable assistance in data analysis. All authors have given approval to the final version of the manuscript, decided to submit the work for publication.

Acknowledgement

The metabolic detection was performed at the Beijing Genomics Institute (BGI) in Shenzhen Province of China. The authors also thank Zhimin Zhang and Othman Soufan for their valuable assistance in fish culture, tissues sampling and data analysis.

Reference

1 FAO. FAO yearbook: Fishery and aquaculture statistics. 2012. Rome: FAO.

2 Richardson. FAO Cultured Aquatic Species Information Programme - Hypophthalmichthys nobilis. , 1845.

3 Wang F, Zhang J, Mu W, Fu Z, Zhang X. Consumers' perception toward quality and safety of fishery products, Beijing, China. Food Control 2009; **20**(10): 918-922.

4 Gui J F, Zhu ZY. Molecular basis and genetic improvement of economically important traits in aquaculture animals. Chin Sci Bull 2012; **57**, 1751-1760.

5 Lie éyvind (Ed.). Improving farmed fish quality and safety. Crc Press, 2008.

6 Alfaro AC, Young T. Showcasing metabolomic applications in aquaculture: a review. Reviews in Aquaculture 2016.

7 Martin SAM, Król E. Nutrigenomics and immune function in fish: new insights from omics technologies. Developmental & Comparative Immunology 2017; **75**: 86-98.

8 Patti GJ, Yanes O, Siuzdak G. Metabolomics: the apogee of the omic triology. Nat Rev Mol Cell Biol 2012; **13**(4): 263-269. doi: 10.1038/nrm3314.

9 Simopoulos AP. An Increase in the Omega-6/Omega-3 Fatty Acid Ratio Increases the Risk for Obesity. Nutrients 2016; **8**(3).

10 Yang B, Ren XL, Fu YQ, Gao JL, Li D. Ratio of n-3/n-6 PUFAs and risk of breast cancer: a meta-analysis of 274135 adult females from 11 independent prospective studies. BMC Cancer 2014; **14**(1): 1-14.

11 Pirillo A, Catapano A.L. Omega-3 polyunsaturated fatty acids in the treatment of hypertriglyceridaemia. International Journal of Cardiology 2013; **170**(2 Suppl 1): S16.

12 Bertol TM, Campos RM, Ludke JV, Terra NN, Figueiredo EA, Coldebella A, dos-Santos FJI, Kawski VL, Lehr NM. Effects of genotype and dietary oil supplementation on performance, carcass traits, pork quality and fatty acid composition of backfat and intramuscular fat. Meat Sci 2013; **93**: 507-516.

13 Hebeisen DF, Hoeflin F, Reusch HP, Junker E, Lauterburg BH. Increased concentrations of omega-3 fatty acids in milk and platelet rich plasma of grass-fed cows. International journal for vitamin and nutrition research. Internationale Zeitschrift fur Vitamin- und Ernahrungsforschung. Journal international de vitaminologie et de nutrition 1993; **63**(3): 229-33.

14 Mráz J, Máchová J, Kozák P, Pickova J. Lipid content and composition in common carp-optimization of n-3 fatty acids in different pond production systems. Journal of Applied Ichthyology 2012; **28**(2): 238-244.

15 Abro R, Moazzami AA, Lindberg JE, Lundh T. Metabolic insights in Arctic charr (*Salvelinus alpinus*) fed with zygomycetes and fish meal diets as assessed in liver using nuclear magnetic resonance (NMR) spectroscopy. International Aquatic Research 2014; **6**: 63.

16 Aslan SS, Guven KC, Gezgin T, Alpaslan M, Tekinay A. Comparison of fatty acid contents of wild and cultured rainbow trout *Onchorhynchus mykiss* in Turkey. Fisheries Sci 2007; **73**: 1195-1198.

17 Kjær MA, Todorčević M, Torstensen BE, Vegusdal A, Ruyter B. Dietary n-3 HUFA Affects Mitochondrial Fatty Acid β-Oxidation Capacity and Susceptibility to Oxidative Stress in Atlantic Salmon. Lipids 2008; **43**(9): 813-827.

18 Ladeira MM, Santarosa LC, Chizzotti ML, Ramos EM, Machado-Neto OR, Oliveira DM, Carvalho JRR, Lopes LS, Ribeiro JS. Fatty acid profile, color and lipid oxidation of meat from young bulls fed ground soybean or rumen protected fat with or without monensin. Meat Science 2014; **96**(1): 597-605. https://doi.org/10.1016/j.meatsci.2013.04.062.

19 Wasowicz E, Gramza A, Heś M, Jelen´ HH, Korczak J, Malecka M, Mildner-Szkudlarz S, Rudziñska M, Samotyja U, Zawirka-Wojtasiak R. Oxidation of Lipids in Food. Pol. J. Food Nutr. Sci 2004; **13**(1): 87-100.

20 Du ZY, Ma T, Liaset B, Keenan AH, Araujo P, Lock EJ, Demizieux L, Degrace P, Frøyland L, Kristiansen K, Madsen L. Dietary eicosapentaenoic acid supplementation accentuates hepatic triglyceride accumulation in mice with impaired fatty acid oxidation capacity. Biochim. Biophys. Acta 2013; **1831**, 291-299. doi: 10.1016/j.bbalip.2012.10.002.

21 Zhao HH, Xia JG, Zhang X, He XG, Li L, Tang R, Chi W, Li DP. Diet Affects Muscle Quality and Growth Traits of Grass Carp (*Ctenopharyngodon idellus*): A Comparison Between Grass and Artificial Feed. Front. Physiol 2018; **9**: 283-295.

22 Jensen GL, Shelton WL. Gonadal differentiation in relation to sex control of grass carp, *Ctenopharyngodon idella* (Pisces: Cyprinidae). Copeia 1983; **1983**: 749-755.

23 Ke HW. An excellent freshwater food fish, Megalobrama amblycephala, and its propagating and culturing. Acta Hydrobiol. Sin 1975; **5**, 293-312.

24 Shi X, Li D, Zhuang P, Nie F, Long L. Comparative blood biochemistry of Amur sturgeon, Acipenser schrenckii, and Chinese sturgeon, Acipenser sinensis. Fish Physiology and Biochemistry 2006; **32**: 63-66.

25 Rasmussen RS, Ostenfeld TH. Influence of growth rate on white muscle dynamics in rainbow trout and brook trout. J Fish Biol 2000; **56**: 1548-1552. doi:10.1111/j.1095-8649.2000.tb02164.x.

26 Luther PK, Munro PMG, Squire JM. Muscle ultrastructure in the teleost fish. Micron 1995; **26**: 431-459.

27 Chong J, Soufan O, Li C, Caraus I, Li S, Bourque G, Wishart DS, Xia J. MetaboAnalyst 4.0: towards more transparent and integrative metabolomics analysis . Nucl. Acids Res 2018; doi:10.1093/nar/gky310.

28 Storey JD, Tibshirani R. Statistical significance for genomwide studies. Proc Natl Acad Sci USA 2003; **100**: 9440-5.

29 Li S, Park Y, Duraisingham S, Strobel FH, Khan N, Soltow QA, Jones DP, Pulendran B. Predicting network activity from high throughput metabolomics. PLoS computational biology 2013; **9**(7): e1003123. https://doi.org/10.1371/journal.pcbi.1003123.

30 Cheng HH, Xie CX, Li DP, Xiao YH, Tian X, Chen J, Tang R, Qi CL, Ma LQ. The study of muscular nutritional components and fish quality of grass carp (*Ctenopharyngodon idellus*) in ecological model of cultivating grass carp with grass. Fisheries of China 2016; **40**: 1050-1059. doi:10.11964/jfc.20150709964. (in Chinese)

31 Elangovan A, Shim KF. The influence of replacing fish meal partially in the diet with soybean meal on growth and body composition of juvenile tin foil barb (*Barbodes altus*). Aquaculture 2000; **189**: 133-144.

32 Harlioglu AG. The influence of replacing fish meal partially in diet with soybean meal and full-fat soya on growth and body composition of rainbow trout (*Oncorhynchus mykiss*). Pak. J Zool 2011; **43**: 175-182.

33 Sun Q, Qi W, Xiao X, Yang SH, Kim D, Yoon KS, Clark JM, Park Y. Imidacloprid promotes high fat diet-induced adiposity in female C57BL/6J mice and enhance adipogenesis in 3T3-L1 adipocytes via AMPKα-mediated pathway. Journal of Agricultural & Food Chemistry 2017; **65**(31): 6572-6581. doi: 10.1021/acs.jafc.7b02584.

34 He AY, Ning LJ, Chen LQ, Chen YL, Xing Q, Li JM, Qiao F, Li DL, Zhang ML, Du ZY. Systemic adaptation of lipid metabolism in response to low- and high-fat diet in Nile tilapia (*Oreochromis niloticus*). Physiol Rep 2015; **3**(8): e12485. doi:10.14814/phy2.12485.

35 Listrat A, Lebret B, Louveau I, Astruc T, Bonnet M, Lefaucheur L, Picard B, Bugeon J. How muscle structure and composition influence meat and flesh quality. The Scientific World Journal 2016; **2016**(6): 1-14.

36 Doreau M, Chilliard Y. Digestion and metabolism of dietary fat in farm animals. British Journal of Nutrition 1997; **78** Suppl 1(1): S15.

37 West DB, York B. Dietary fat, genetic predisposition, and obesity: lessons from animal models. American Journal of Clinical Nutrition 1998; **67**(3 Suppl): 505S.

38 Lanza M, Bella M, Priolo A, Barbagallo D, Galofaro V, Landi C, Pennisi P. Lamb meat quality as affected by a natural or artificial milk feeding regime. Meat Science 2006; **73**(2): 313-318.

39 Hedrick VE, Dietrich AM, Estabrooks PA, Savla J, Serrano E, Davy BM. Dietary biomarkers: advances, limitations and future directions. Nutrition Journal 2012; **11**(1): 109-109.

40 Gjerlaugenger E, Haug A, Gaarder M, Ljøkjel K, Stenseth RS, Sigfridson K, Egelandsdal B, Saarem K, Berg P. Pig feeds rich in rapeseed products and organic selenium increased omega-3 fatty acids and selenium in pork meat and backfat. Food Science & Nutrition 2015; **3**(2): 120-128.

41 Markworth JF, Mitchell CJ, D'Souza RF, Aasen KMM, Durainayagam BR, Mitchell SM, Chan AHC, Sinclair AJ, Garg M, Cameron-Smith D. Arachidonic acid supplementation modulates blood and skeletal muscle lipid profile with no effect on basal inflammation in resistance exercise trained men. Prostaglandins Leukotrienes & Essential Fatty Acids 2018; **128**: 74-86.

42 Leaver MJ, Tocher DR, Obach A, Jensen L, Henderson RJ, Porter AR, Krey G. Effect of dietary conjugated linoleic acid (cla) on lipid composition, metabolism and gene expression in atlantic salmon (*Salmo salar*) tissues. Comp Biochem Physiol A Mol Integr Physiol 2006; **145**(2): 258-267.

43 Kondracki S. Anote on fatty acid profile of skeletal muscle fat in Pulawska and Polish Large White pigs as affected by feeding level and sex. Anim. Sci. Pap. Rep 2000; **18**: 137-143.

44 Smith SB, Gill CA, Lunt DK, Brooks MA. Regulation of fat and fatty acid composition in beef cattle. Asian-Australasian Journal of Animal Sciences 2009; **22**(9): 1225-1233.

45 Kaur N, Chugh V, Gupta AK. Essential fatty acids as functional components of foods- a review. Journal of Food Science & Technology 2014; **51**(10): 2289-2303.

46 Aziz NA, Azlan A, Ismail A, Alinafiah SM, Razman MR. Quantitative Determination of Fatty Acids in Marine Fish and Shellfish from Warm

Water of Straits of Malacca for Nutraceutical Purposes. BioMed Research International 2013; Article ID 284329, 12 pages. Doi: org/10.1155/2013/284329.

47 Wilson TA, Kritchevsky D, Kotyla T, Nicolosi RJ. Structured triglycerides containing caprylic (8:0) and oleic (18:1) fatty acids reduce blood cholesterol concentrations and aortic cholesterol accumulation in hamsters. Biochim Biophys Acta 2006; **1761**(3): 345-9. doi: 10.1016/j.bbalip.2006.02.019.

48 Kajikawa M, Yamato KT, Kohzu Y, et al. Isolation and Characterization of Δ 6-Desaturase, an ELO-Like Enzyme and Δ 5-Desaturase from the Liverwort Marchantia Polymorpha, and Production of Arachidonic and Eicosapentaenoic Acids in the Methylotrophic Yeast Pichia Pastoris. Plant Molecular Biology 2004; **54**(3): 335-352.

49 Palombo JD, DeMichele SJ, Boyce PJ, Noursalehi M, Forse RA, Bistrian BR. Metabolism of dietary alpha-linolenic acid vs. eicosapentaenoic acid in rat immune cell phospholipids during endotoxemia. Lipids 1998; **33**(11): 1099-1105.

50 Stark KD, Lim SY, Jr SN. Artificial rearing with docosahexaenoic acid and n-6 docosapentaenoic acid alters rat tissue fatty acid composition. Journal of Lipid Research 2007; **48**(11): 2471-7.

51 Risérus U, Smedman A, Basu S, Vessby B. Metabolic effects of conjugated linoleic acid in humans: the Swedish experience. The American Journal of Clinical Nutrition 2004; **79**(6): 1146S-1148S. https://doi.org/10.1093/ajcn/79.6.1146S.

52 Taylor CG, Zahradka P. Do high dietary intakes of linoleic acid protect against death from coronary heart disease and cardiovascular disease? Clinical Lipidology 2017; **8**(5): 493-495. https://doi.org/10.2217/clp.13.48.

53 Kanazawa A. Essential Fatty Acids in the Diet of Prawn-I. Effects of linoleic and linolenic acids on growth. Nippon Suisan Gakkaishi 1977; **43**(9): 1111-1114. doi: 10.2331/suisan.43.1111.

54 Simopoulos AP. Essential fatty acids in health and chronic disease. Am J Clin Nutr 1999; **70**: 560s-569s.

55 Sinn N, Milte CM, Street SJ, Buckley JD, Coates AM, Petkov J, Howe PRC. Effects of n-3 fatty acids, EPA v. DHA, on depressive symptoms, quality of life, memory and executive function in older adults with mild cognitive impairment: a 6-month randomised controlled trial. Brit J Nutr 2012; **107**: 1682-1693. doi: 10.1017/S0007114511004788.

56 Calder PC. Polyunsaturated fatty acids and inflammation. Prostaglandins, Leukotrienes and Essential Fatty Acids 2006; **75**(3): 197-202.

57 Martineau C, Martin-Falstrault L, Brissette L, Moreau R. Gender- and region-specific alterations in bone metabolism in scarb1-null female mice. Journal of Endocrinology 2014; **222**(2): 277-288.

58 Schacky CV. n-3 Fatty acids and the prevention of coronary atherosclerosis. American Journal of Clinical Nutrition 2000; **71**(71): 224S-7S.

59 Khanapure SP, Garvey DS, Janero DR, Letts LG. Eicosanoids in inflammation: biosynthesis, pharmacology, and therapeutic frontiers. Curr Top Med Chem 2007; **7**(3): 311-40. doi: 10.2174/156802607779941314.

60 Mizock BA. Alterations in carbohydrate metabolism during stress: A review of the literature. The American Journal of Medicine 1995; **98**(1): 75-84.

61 Hocquette JF, Ortigues-Marty I, Pethick D, Herpin P, Fernandez X. Nutritional and hormonal regulation of energy metabolism in skeletal muscles of meat-producing animals. Livestock Production Science 1998; **56**(2): 115-143.

62 Caipang CMA, Lazado C C. 9 - Nutritional impacts on fish mucosa: immunostimulants, pre- and probiotics. Mucosal Health in Aquaculture 2015; **2015**: 211-272.

63 Lo SK, Tan CP, Long K, Yusoff MSA, Lai OM. Diacylglycerol Oil-Properties, Processes and Products: A Review. Food and Bioprocess Technology 2008; **1**(3): 223-233.

64 Phuah ET, Tang TK, Lee YY, Choong TS, Tan CP, Lai OM. Review on the Current State of Diacylglycerol Production Using Enzymatic Approach. Food and Bioprocess Technology 2015; **8**(6): 1169-1186. doi:10.1007/s11947-015-1505-0.

65 Wiemer AJ, Wiemer DF, Hohl RJ. Geranylgeranyl diphosphate synthase: an emerging therapeutic target. Clinical pharmacology and therapeutics 2011; **90**(6): 804-12.

66 Rigotti A. Absorption, transport, and tissue delivery of vitamin E. Mol. Aspects Med 2007; **28**(5-6): 423-36.

67 Strobel C, Jahreis G, Kuhnt K. Survey of n-3 and n-6 polyunsaturated fatty acids in fish and fish products. Lipids Health Dis 2012; **11**: 144. doi: 10.1186/1476-511X-11-144.

68 Williams CD, Whitley BM, Hoyo C, Grant DJ, Iraggi JD, Newman KA, Gerber L, Taylor LA, McKeever MG, Freedland SJ. A high ratio of dietary n-6/n-3 polyunsaturated fatty acids is associated with increased risk of prostate cancer. Nutr Res **2011**; 31(1): 1-8.

69 Xia SH, Wang JD, Kang JX. Decreased n-6/n-3 fatty acid ratio reduces the invasive potential of human lung cancer cells by down regulation of cell adhesion/ invasion-related genes. Carcinogenesis 2005; **26**(4): 779-784.

70 Zhao H, Chong J, Tang R, Li L, Xia J, Li D: Supporting data for "Metabolomics Investigation of Dietary Effects on Flesh Quality in Grass Carp (Ctenopharyngodon idellus)" GigaScience Database. 2018. http://dx.doi.org/10.5524/100494

Legends

Figure 1. The experimental design and flowchart.

Figure 2. Histological sections of abdominal muscles of *C. idellus*. The abdominal muscle samples were collected from artificial fed *C. idellus* (AF) and grass fed *C. idellus* (GF). (A) H&E staining (original magnification × 200) exhibit the characteristics of abdominal muscle fibers, the Oil Red-O staining sections present the distributions of lipid droplets. (B) The statistical observations of muscle tissues sections (n = 50). The four different colors represent the four test groups: Black - ♂-AF, Grey - ♂-GF, Dark Grey - ♀-AF, White - ♀-GF. Vertical bars represent the mean±S.E. The asterisks (**) indicate the significance between AF and GF, under the same sex conditions. The capital “A” was used to represent the significance between two sexes of *C. idellus* fed with artificial feed. In addition, the lowercase “a” represents the significant difference between ♂-GF and ♀-GF.

Figure 3. PCA score plots for the metabolomics profiles of *C. idellus* muscle samples (n = 10). PCA score plots for the metabolomics profiles of muscle samples from female (A) and male (B) *C. idellus*. Artificial feed group (AF), blue points; natural grass group (GF), red triangles.

Figure 4. Volcano plots for the potential metabolomic features of muscle samples from female (A) and male (B) *C. idellus* (n = 10). Pink points indicate significant metabolites between the two groups (FC < 0.5 or > 2.0; q-value < 0.05). The gray points showed tentatively matched features with no significance. The potential biomakers between experimental groups were annotated with their matched metabolite names, those non-annotated peaks were marked with their corresponding mass weights and retention time.

Figure 5. The differential and overlapped metabolites between the four test groups. Heatmap visualization of metabolomic data showed the relative intensities of significant features, those are not only annotated by existed metabolites database and also overlapped among the four test groups (FAF, FGF, MAF and MGF). Each row was labeled

with the tentative metabolite names. The colors refer to the relative levels of these compounds from high (red) to low (blue).

Figure 6. The pathway enrichment and network analyses for the significant metabolites in female *C. idellus.* (A) The scatter plot was used to visualize the pathway impact and enrichment results for all matching significant metabolites in female *C. idellus*; (B) The KEGG global metabolic network visualization of all significant metabolites (P < 0.05) in the female *C. idellus* metabolic profile. The colored points represent different metabolic pathways. The various color levels indicate different levels of significance of metabolic pathways from low (white) to high (red). The different sizes of each point were used to represent the number of metabolites participated in the metabolic pathway. The greater rich factor, the greater the degree of pathway enrichment. Moreover the corresponding pathway's name of each point is labeled. In the metabolic network, All up-regulated metabolites (fold-change AF/GF > 2) in AF groups were colored with red, whereas the down regulated metabolites (fold-change < 0.5) were colored in green. In addition, the different color circles represent the various physiological functions that the discriminating metabolites belong to. Moreover, each enriched pathway is annotated with the corresponding name.

Figure 1

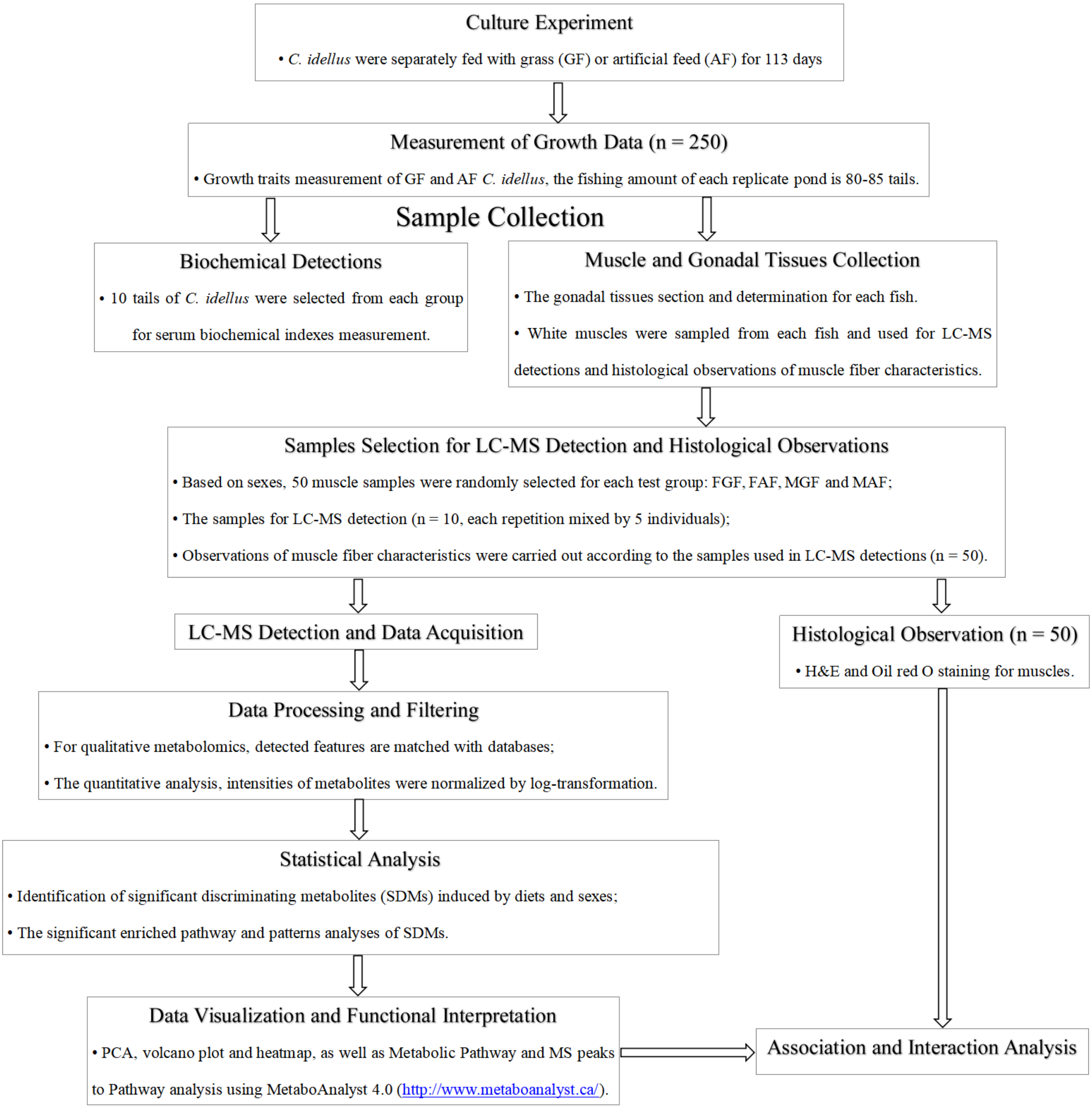
Culture Experiment
• C. idellus were separately fed with grass (GF) or artificial feed (AF) for 113 days
Measurement of Growth Data (n = 250)
• Growth traits measurement of GF and AF C. idellus, the fishing amount of each replicate pond is 80-85 tails.
Sample Collection
Biochemical Detections
• 10 tails of C. idellus were selected from each group for serum biochemical indexes measurement.
Muscle and Gonadal Tissues Collection
• The gonadal tissues section and determination for each fish.
• White muscles were sampled from each fish and used for LC-MS detections and histological observations of muscle fiber characteristics.
Samples Selection for LC-MS Detection and Histological Observations
• Based on sexes, 50 muscle samples were randomly selected for each test group: FGF, FAF, MGF and MAF;
• The samples for LC-MS detection (n = 10, each repetition mixed by 5 individuals);
• Observations of muscle fiber characteristics were carried out according to the samples used in LC-MS detections (n = 50).
LC-MS Detection and Data Acquisition
Histological Observation (n = 50)
• H&E and Oil red O staining for muscles.
Data Processing and Filtering
• For qualitative metabolomics, detected features are matched with databases;
• The quantitative analysis, intensities of metabolites were normalized by log-transformation.
Statistical Analysis
• Identification of significant discriminating metabolites (SDMs) induced by diets and sexes;
• The significant enriched pathway and patterns analyses of SDMs.
Data Visualization and Functional Interpretation
• PCA, volcano plot and heatmap, as well as Metabolic Pathway and MS peaks to Pathway analysis using MetaboAnalyst 4.0 (http://www.metaboanalyst.ca/).
Association and Interaction Analysis

Figure 2

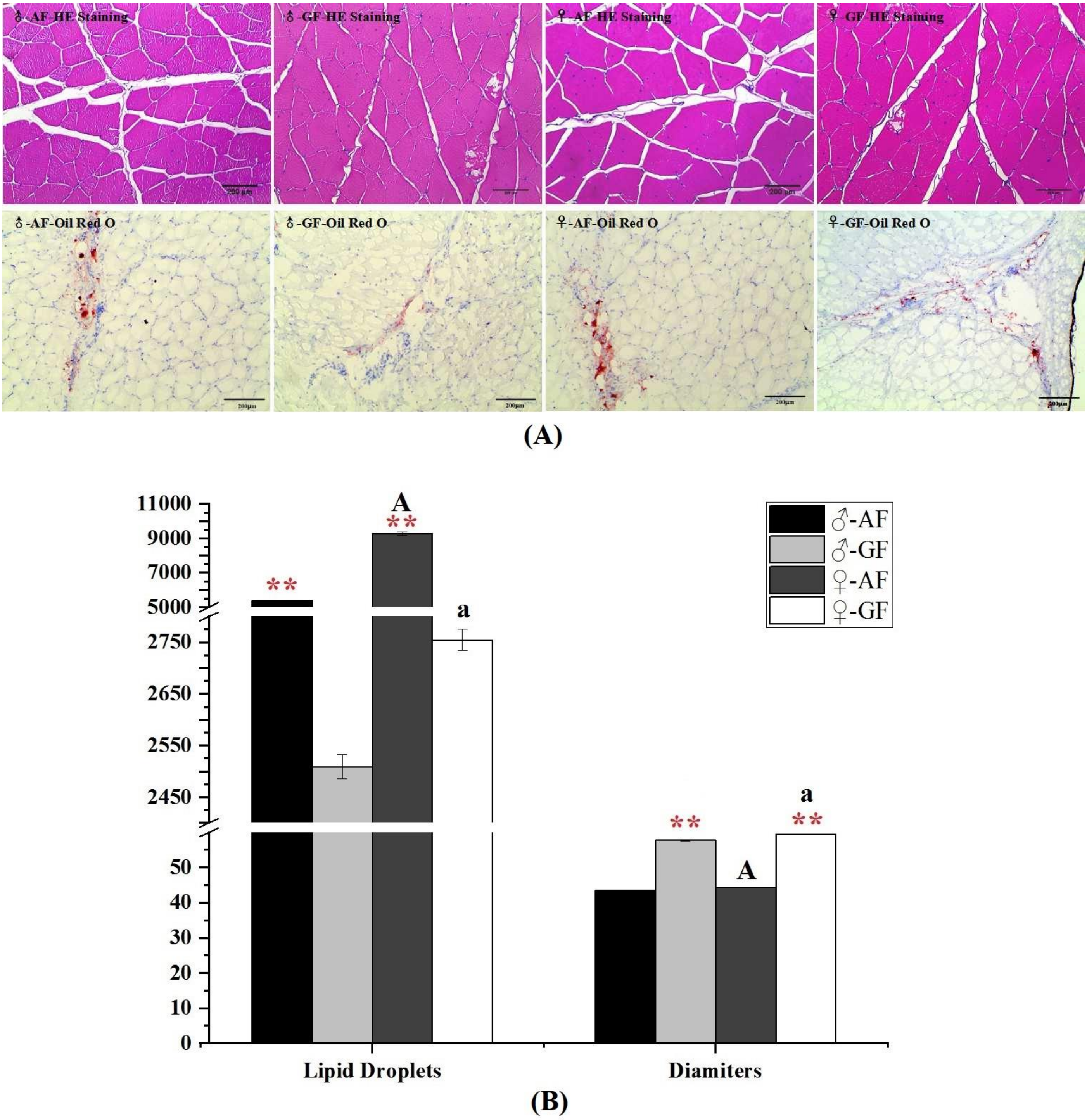
♂-AF-HE Staining
♂-GF-HE Staining
♀-AF-HE Staining
♀-GF-HE Staining
200 μm
♂-AF-Oil Red O
♂-GF-Oil Red O
♀-AF-Oil Red O
♀-GF-Oil Red O
200μm
(A)
11000
9000
7000
5000
2750
2650
2550
2450
50
40
30
20
10
0
A
a
**
♂-AF
♂-GF
♀-AF
♀-GF
Lipid Droplets
Diamiters
(B)

Figure 3

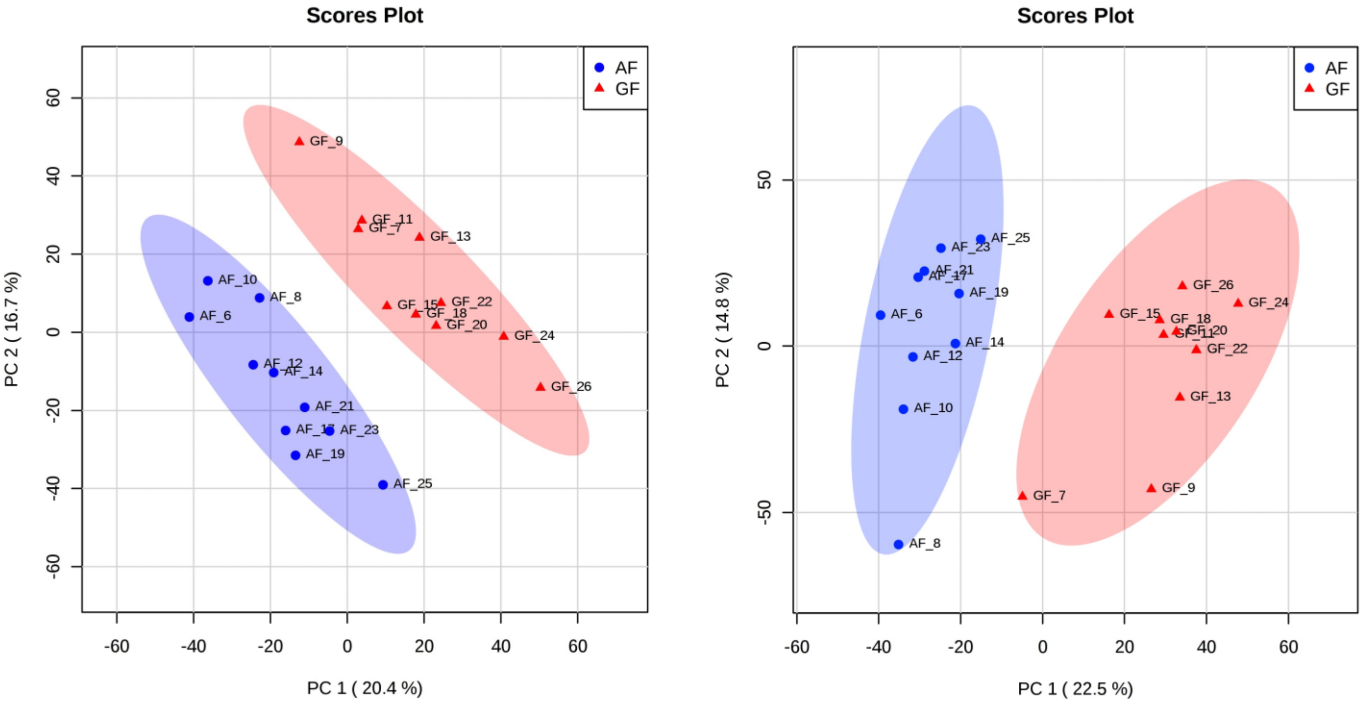

(A) (B)

Figure 4

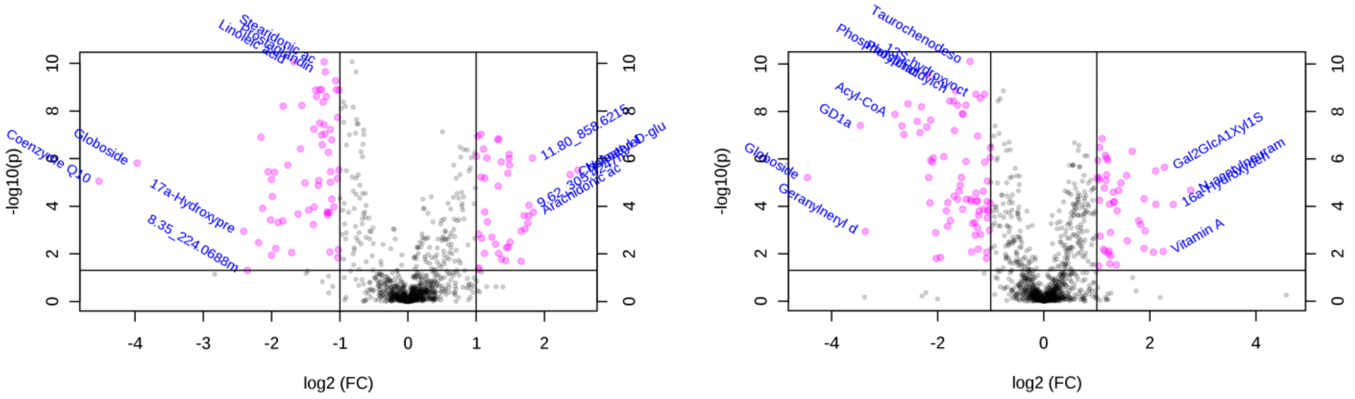

(A) (B)

Figure 5

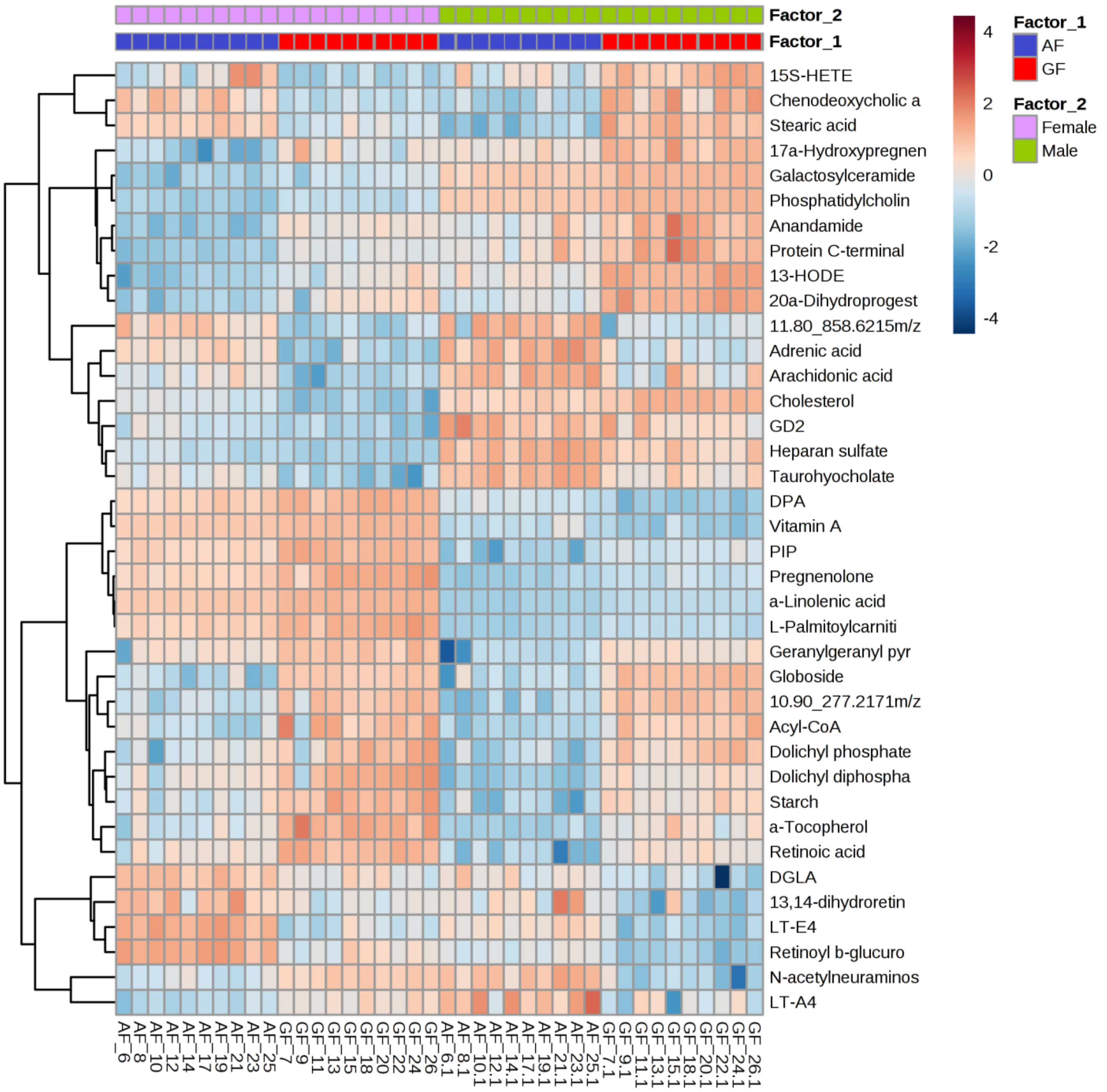
Factor_2
Factor_1
15S-HETE
Chenodeoxycholic a
Stearic acid
17a-Hydroxypregnen
Galactosylceramide
Phosphatidylcholin
Anandamide
Protein C-terminal
13-HODE
20a-Dihydroprogest
11.80_858.6215m/z
Adrenic acid
Arachidonic acid
Cholesterol
GD2
Heparan sulfate
Taurohyocholate
DPA
Vitamin A
PIP
Pregnenolone
a-Linolenic acid
L-Palmitoylcarniti
Geranylgeranyl pyr
Globoside
10.90_277.2171m/z
Acyl-CoA
Dolichyl phosphate
Dolichyl diphospha
Starch
a-Tocopherol
Retinoic acid
DGLA
13,14-dihydroretin
LT-E4
Retinoyl b-glucuro
N-acetylneuraminos
LT-A4
AF_6
AF_8
AF_10
AF_12
AF_14
AF_17
AF_19
AF_21
AF_23
AF_25
GF_7
GF_9
GF_11
GF_13
GF_15
GF_18
GF_20
GF_22
GF_24
GF_26
AF_6.1
AF_8.1
AF_10.1
AF_12.1
AF_14.1
AF_17.1
AF_19.1
AF_21.1
AF_23.1
AF_25.1
GF_7.1
GF_9.1
GF_11.1
GF_13.1
GF_15.1
GF_18.1
GF_20.1
GF_22.1
GF_24.1
GF_26.1
4
2
0
-2
-4
Factor_1
AF
GF
Factor_2
Female
Male

Figure 6

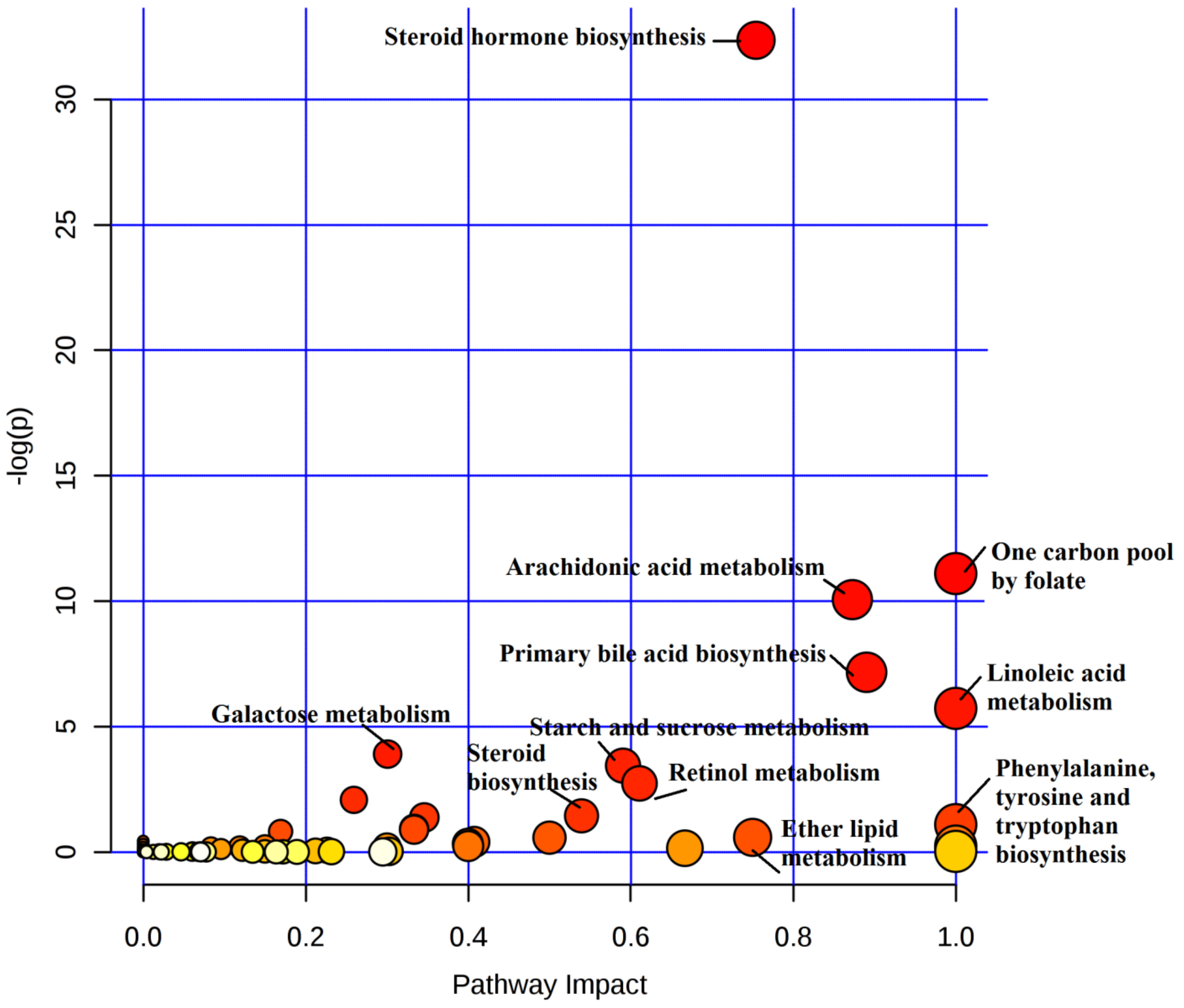
Steroid hormone biosynthesis
Arachidonic acid metabolism
One carbon pool by folate
Primary bile acid biosynthesis
Linoleic acid metabolism
Galactose metabolism
Starch and sucrose metabolism
Steroid biosynthesis
Retinol metabolism
Phenylalanine, tyrosine and tryptophan biosynthesis
Ether lipid metabolism
-log(p)
0
5
10
15
20
25
30
0.0
0.2
0.4
0.6
0.8
1.0
Pathway Impact

(A)

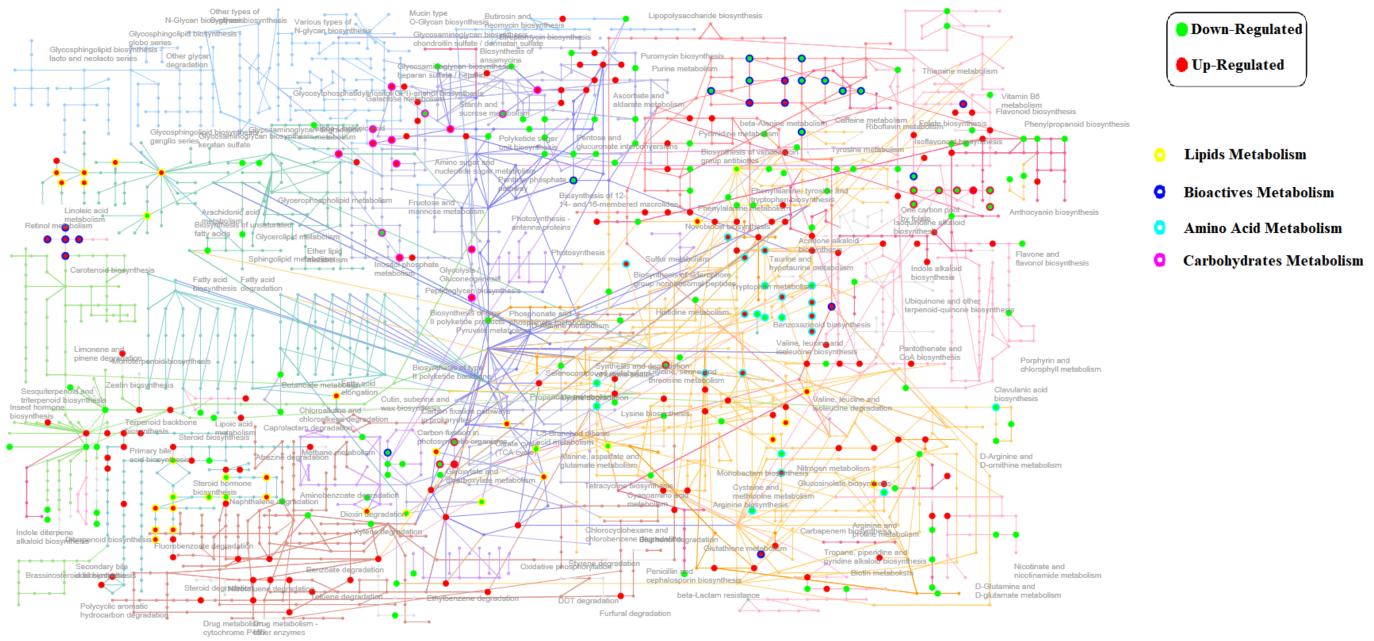
Down-Regulated
Up-Regulated
Lipids Metabolism
Bioactives Metabolism
Amino Acid Metabolism
Carbohydrates Metabolism

(B)

Table 1. Growth data of *Ctenopharyngodon idellus* fed with different feeds (n = 250).

Gender	Experimental Group	Body Mass (g)	Body Length (cm)	Body Height (cm)	Visceral Weight (g)	Liver Weight (g)	SGR (%)	CF (%)
♀	GF	971.64± 5.91 ^{a}	31.29± 0.56 ^{a}	6.46± 0.04	38.20±0.31	7.50±0.03 ^{a}	2.94±0.01 ^{a}	3.19±0.19 *
	AF	1080.80± 6.25 ^{A} **	33.81± 0.21 **	7.46± 0.30 **	60.63±0.78 ^{A} **	14.97±0.07 ^{A} **	3.04±0.00 ^{A} **	2.80±0.04
♂	GF	706.94± 10.46	29.02± 0.28	6.22± 0.07	37.60±0.48	7.01±0.04	2.66±0.02	2.89±0.04 *
	AF	979.50± 10.02 **	33.90± 0.25 **	7.65± 0.11 **	51.71±0.06 **	13.24±0.14 **	2.95±0.01 **	2.51±0.05

Note: Measured traits of growth performances are represented as mean ± S.E.; Compared under the same sex conditions, **, difference between the two experimental groups is significant at the 0.01 level; *, difference is significant at the 0.05 level. The superscripts, lower-case letters mean there is significance between different genders in GF; capital letters indicate significance between female and male in AF.

Table 2. Serum biochemical parameters in *C. idellus* farmed under two feeding models.

Groups	LD	AST	ALT	ALP	TCHO	HDLC	GLU	ALB	TP	TG
**Units**	U/L	U/L	U/L	U/L	mmol/l	mmol/l	mmol/l	g/l	g/l	mmol/l
**GF**	829.17±0.33 **	161.00±0.87 **	237.67±0.44 **	132.83±0.60 **	7.69±0.06 **	2.50±0.20	3.66±0.02 **	3.33±0.17	36.50±0.01 **	6.25±0.00
**AF**	506.78±0.40	125.78±0.22	163.67±0.51	93.78±0.80	6.27±0.02	2.14±0.05	2.62±0.05	3.89±0.11 *	29.78±0.11	6.71±0.02 **

Note: All the serum biochemical parameters were measured and calculated by 10 fish from each experimental group. Measured indexes of serum biochemical are represented as mean ± S.E.; Compared between the two feeding groups, **, difference between the two experimental groups is significant at the 0.01 level; *, difference is significant at the 0.05 level.

Table 3. Pathway impact and overlapped metabolites analysis of female *C. idellus*.

Pathway Name	Hits	Raw p	FDR	Impact	Overlapping Metabolites in Pathways
Steroid hormone biosynthesis	44/ 56	0.0000	0.0000	0.7542	Cholesterol (C00187), Androstenedione (C00280), Progesterone (C00410), Estrone (C00468), Androsterone (C00523), Cortisone (C00762), 17-Hydroxyprogesterone (C01176), DHEA (C01227), Pregnenolone (C01953), Corticosterone (C02140), Deoxycorticosterone (C03205), DHT (C03917), 5-Androstenediol (C04295), Etiocholanolone (C04373), 17α-Hydroxypregnenolone (C05138), Adrenosterone (C05285), 16α-Hydroxy-DHEA (C05139), 11-DHC (C05490), 16α-Hydroxyandrost-4-ene-3,17-dione (C05140), 11β-Hydroxyandrost-4-ene-3,17-dione (C05284), Estriol (C05141), 19-Hydroxyandrost-4-ene-3,17-dione (C05290), 19-Hydroxytestosterone (C05294), 2-Hydroxyestrone (C05298), 2-Methoxyestrone (C05299), 2-Hydroxyestradiol (C05301), Testosterone glucuronide (C11134), 7-Hydroxy-DHEA (C18045), 21-Hydroxypregnenolone (C05485), Tetrahydrocorticosterone (C05476), 20α-Hydroxycholesterol (C05500), 3α,21-Dihydroxy-5b-pregnane-11,20-dione (C05478), 17a,21-Dihydroxypreg-nenolone (C05487), Cortexolone (C05488), 11β,17α,21-Trihydroxypreg-nenolone (C05489), 20α,22β-Dihydroxycholesterol (C05501), 22β-Hydroxycholesterol (C05502), 17β-Estradiol-3-glucuronide (C05503), 2-Methoxy-estradiol-17β 3-glucuronide (C11131), 2-Methoxyestrone 3-glucuronide (C11132), Estrone glucuronide (C11133), Androsterone glucuronide (C11135), Etiocholanolone glucuronide (C11136), 11β,17β-Dihydroxy-4-androsten-3-one (C18075)
One carbon pool by folate	9/ 9	0.0000	0.0006	1.0000	THF (C00101), 5,10-Methylene-THF (C00143), 10-CHO-THF (C00234), DHF (C00415), 5-MTHF (C00440), 5,10-CH=THF (C00445), Folic acid (C00504), 5-Formimino-THF (C00664), N5-Formyl-THF (C03479)
Arachidonic acid metabolism	20/ 31	0.0000	0.0012	0.8728	ARA (C00219), PGD2 (C00696), LTA4 (C00909), PGI2 (C01312), LTC4 (C02166), 15(S)-HETE (C04742), 5-HETE (C04805), 5-HPETE (C05356), LTD4 (C05951), PGG2 (C05956), 15(S)-HPETE (C05966), 19(S)-HETE (C14749), 5,6-Epoxy-DGLA (C14768), 8,9-EET (C14769), 11,12-EET (C14770), 11H-14,15-EETA (C14813), 14,15-EET (C14771), 15H-11,12-EETA (C14781), 11,12,15-THETA (C14782), 11,14,15-THETA (C14814)
Primary bile acid biosynthesis	20/ 36	0.0008	0.0157	0.8903	Cholesterol (C00187), 3α,7α,12α-Trihydroxy-5β-cholestan-26-al (C01301), 7α-Hydroxycholesterol (C03594), 3α,7α-Dihydroxy-5β-cholestanate (C04554), 3α,7α,12α-Trihydroxy-5β-cholestanoic acid (C04722), 3α,7α,26-Trihydroxy-5β-cholestane (C05444), 3α,7α-Dihydroxy-5β-cholestan-26-al (C05445), 27-Deoxy-5β-cyprinol (C05446), 3α,7α-Dihydroxy-5β-cholestane (C05452), 5β-Cholestane-3α,7α,12α-triol (C05454), 12,13-EpOME (C14826), 7α-Hydroxy-cholestene-3-one (C05455), 7α,27-Dihydroxycholesterol (C06341), 24-Hydroxycholesterol (C13550), (24S)-7α,24-Dihydroxycholesterol (C15518), 25-Hydroxycholesterol (C15519), Cholest-5-ene-3b,26-diol (C15610), 3β-Hydroxy-5-cholestenoate (C17333), 7α,26-Dihydroxy-4-cholesten-3-one (C17336), 13(S)-HPODE (C04717), 7α-Hydroxy-3-oxo-4-cholestenoate (C17337), 4-Cholesten-7α,12α-diol-3-one (C17339)

Linoleic acid metabolism	6/ 7	0.0033	0.0530	1.0000	Linoleic acid (C01595), 13-HODE (C14762), 13-OxoODE (C14765), 9,10-Epoxyoctadecenoic acid (C14825)
Galactose metabolism	13/ 26	0.0202	0.2732	0.3008	D-Glucose (C00031), UDP-glucose (C00029), UDP-galactose (C00052), Sucrose (C00089), α-Lactose (C00243), α-D-Glucose (C00267), Raffinose (C00492), Sorbitol (C00794), Melibiitol (C05399), Epimelibiose (C05400), Galactosylglycerol (C05401), Melibiose (C05402), D-Gal α 1->6D-Gal α 1->6D-Glucose (C05404)
Starch and sucrose metabolism	11/ 12	0.0320	0.3707	0.5905	Starch (C00369), Sucrose (C00089), α-D-Glucose (C00267), D-Glucose (C00031), UDP-glucose (C00029), Dextrin (C00721), UDP-glucuronic acid (C00167), 1β-D-Glucopyranosyl-4-D-glucopyranose (C00185), D-Maltose (C00208), β-D-Glucose (C00221), 1,4β-D-Glucan (C00760)
Retinol metabolism	8/ 16	0.0651	0.6589	0.6108	Retinal (C00376), Vitamin A (C00473), 11-cis-Retinol (C00899), All-trans-13,14-dihydroretinol (C15492), Retinoyl β-glucuronide (C11061), 9-cis-Retinoic acid (C15493), 9-cis-Retinal (C16681), 9-cis-Retinol (C16682)

Table 4. Pathway impact and overlapped metabolites analysis of male *C. idellus*.

**Pathway Name**	**Hits**	**Raw p**	**FDR**	**Impact**	**Overlapping Metabolites in Pathways**
Steroid hormone biosynthesis	43/ 56	0.0000	0.0000	0.7648	Cholesterol (C00187), Androstenedione (C00280), P4 (C00410), Estrone (C00468), Androsterone (C00523), Cortisone (C00762), 17-OHPG (C01176), Etiocholanolone (C04373), DHEA (C01227), Pregnenolone (C01953), Corticosterone (C02140), DOC (C03205), DHT (C03917), 5-Androstenediol (C04295), 7α-OH-DHEA (C18045), 16α-OH-DHEA (C05139), 17α-Hydroxypregnenolone (C05138), 16α-Hydroxyandrost-4-ene-3,17-dione (C05140), Estradiol (C00951), Cortexolone (C05488), 11β-Hydroxyandrost-4-ene-3,17-dione (C05284), Estrone glucuronide (C11133), Adrenosterone (C05285), 19-Hydroxyandrost-4-ene-3,17-dione (C05290), 11-DHC (C05490), 19-Hydroxytestosterone (C05294), 2-Hydroxyestrone (C05298), 2-Methoxyestrone (C05299), Androsterone glucuronide (C11135), THB (C05476), 3α,21-Dihydroxy-5β-pregnane-11,20-dione (C05478), 20α-Hydroxycholesterol (C05500), 21-Hydroxypregnenolone (C05485), Testosterone glucuronide (C11134), 17α,21-Dihydroxypreg-nenolone (C05487), 11β,17α,21-Trihydroxypreg-nenolone (C05489), 20α,22β-Dihydroxycholesterol (C05501), 22R-Hydroxycholesterol (C05502), 17β-Estradiol-3-glucuronide (C05503), 2-Methoxy-estradiol-17β 3-glucuronide (C11131), 2-Methoxyestrone 3-glucuronide (C11132), Etiocholanolone glucuronide (C11136), 11β,17β-Dihydroxy-4-androsten-3-one (C18075)
Arachidonic acid metabolism	20/ 31	0.0003	0.0133	0.8728	ARA (C00219), PGD2 (C00696), LTA4 (C00909), PGI2 (C01312), LTC4 (C02166), LTD4 (C05951), PGG2 (C05956), 15(S)-HETE (C04742), 5-HETE (C04805), 5-HPETE (C05356), 15(S)-HPETE (C05966), 19(S)-HETE (C14749), 5,6-Epoxy-DGLA (C14768), 8,9-EET (C14769), 11,12-EET (C14770), 11H-14,15-EETA (C14813), 14,15-EET (C14771), 15H-11,12-EETA (C14781), 11,12,15-THETA (C14782), 11,14,15-THETA (C14814)

One carbon pool by folate	8/ 9	0.0009	0.0254	1.0000	THF (C00101), 5,10-Methylene-THF (C00143), 10-CHO-THF (C00234), DHF (C00415), 5-Methyl-THF (C00440), 5,10-Methenyl-THF (C00445), 5-Formimino-THF (C00664), N5-Formyl-THF (C03479)
Retinol metabolism	11/ 16	0.0039	0.0740	0.6108	Retinal (C00376), Vitamin A (C00473), 11-cis-Retinol (C00899), Retinoyl β-glucuronide (C11061), 9-cis-Retinoic acid (C15493), 9-cis-Retinal (C16681), 9-cis-Retinol (C16682), All-trans-13,14-dihydroretinol (C15492), 4-Hydroxyretinoic acid (C16677), all-trans-5,6-Epoxyretinoic acid (C16680), 11-cis-Retinyl palmitate (C03455)
Primary bile acid biosynthesis	20/ 36	0.0046	0.0740	0.8903	Cholesterol (C00187), 3α,7α,12α-Trihydroxy-5β-cholestan-26-al (C01301), 7α-Hydroxycholesterol (C03594), 3α,7α-Dihydroxy-5β-cholestanate (C04554), 7α,26-Dihydroxy-4-cholesten-3-one (C17336), 3α,7α,12α-Trihydroxy-5β-cholestanoic acid (C04722), 3α,7α,26-Trihydroxy-5β-cholestane (C05444), 3α,7α-Dihydroxy-5β-cholestan-26-al (C05445), 27-Deoxy-5β-cyprinol (C05446), 324-Hydroxycholesterol (C13550), α,7α-Dihydroxy-5β-cholestane (C05452), 5β-Cholestane-3α,7α,12α-triol (C05454), 7α-Hydroxy-cholestene-3-one (C05455), 7α,27-Dihydroxycholesterol (C06341), 25-Hydroxycholesterol (C15519), 3β-Hydroxy-5-cholestenoate (C17333), (24S)-7α,24-Dihydroxycholesterol (C15518), Cholest-5-ene-3β,26-diol (C15610), 7α-Hydroxy-3-oxo-4-cholestenoate (C17337), 4-Cholesten-7α,12a-diol-3-one (C17339)
Linoleic acid metabolism	6/ 7	0.0068	0.0916	1.0000	Linoleic acid (C01595), 13(S)-HPODE (C04717), 13-HODE (C14762), 13-OxoODE (C14765), 9,10-Epoxyoctadecenoic acid (C14825), 12,13-EpOME (C14826)
Steroid biosynthesis	18/ 33	0.0091	0.1055	0.6930	Cholesterol (C00187), Squalene (C00751), (S)-2,3-Epoxysqualene (C01054), 7-DHC (C01164), Lathosterol (C01189), Lanosterin (C01724), Desmosterol (C01802), 5α-Cholest-8-en-3β-ol (C03845), 7-Dehydrodesmosterol (C05107), 4,4-Dimethyl-5α-cholesta-8,24-dien-3β-ol (C05108), 24,25-Dihydrolanosterol (C05109), Zymosterol intermediate 2 (C05437), 5α-Cholesta-7,24-dien-3β-ol (C05439), Avenasterol (C08821), 5-Dehydroepisterol (C15780), Delta-7-Avenasterol (C15782), 5-Dehydroavenasterol (C15783), 4,4-Dimethyl-5α-cholesta-8-en-3b-ol (C15915)

Note: The significantly enriched pathways were list in table from lowest FDR p-value to highest (FDR p-value < 0.05) with their corresponding overlapped metabolites in the pathway. Despite of the metabolites names, the mapping features were also annotated with KEGG component IDs.

Table 5. Percentages of nutrients in the different two feed.

Feed type	Crude Protein	Crude Fat	Crude Fiber	Ash
**Natural Grass**	15.3 %	2.8 %	25.9 %	3.5 %

**Artificial Feed**	28.0 %	2.6 %	15.0 %	15.0 %

Table 5. Percentages of nutrients in the different two feed

Feed type	Crude Protein	Crude Fat	Crude Fiber	Ash
Natural Grass	15.30%	2.80%	25.90%	3.50%
Artificial Feed	28.00%	2.60%	15.00%	15.00%

Figure-1

Click here to access/download;Figure;Figure 1.tif

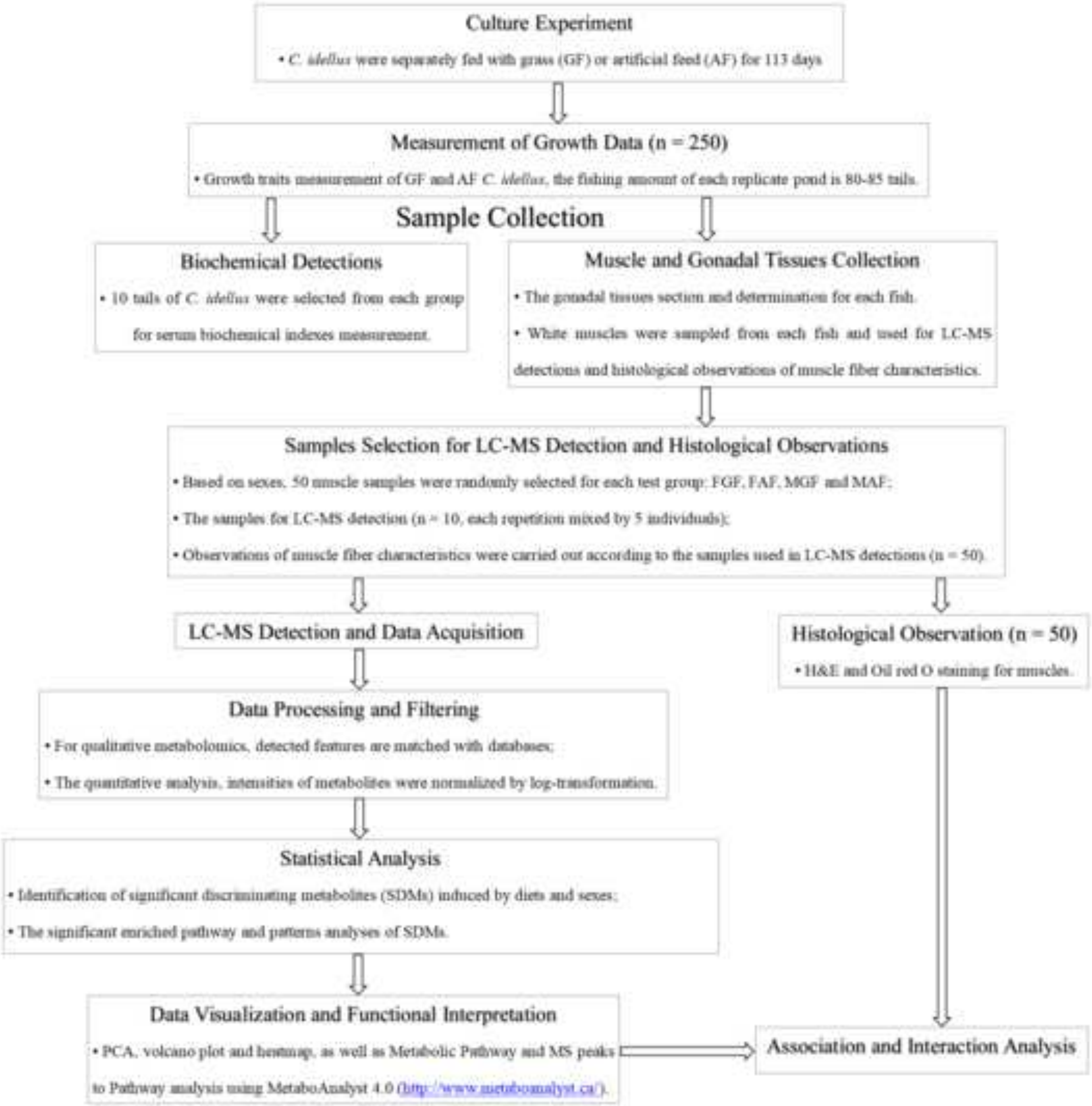

Click here to access/download
Supplementary Material
Revised Supplementary Data.docx

Supplementary Material.pdf

Click here to access/download
Supplementary Material
Revised Supplementary Data.pdf
