# Supplementary material for: Metabolomics investigation of dietary effects on flesh quality in grass carp (Ctenopharyngodon idellus)
Source: Gigascience. 2018 Sep 6;7(10):giy111. doi: 10.1093/gigascience/giy111 (PMC6176498; doi:10.1093/gigascience/giy111)
Supplement: Supplemental Files [file giy111_supplemental_files.zip › Revised Supplementary Data.docx]

**
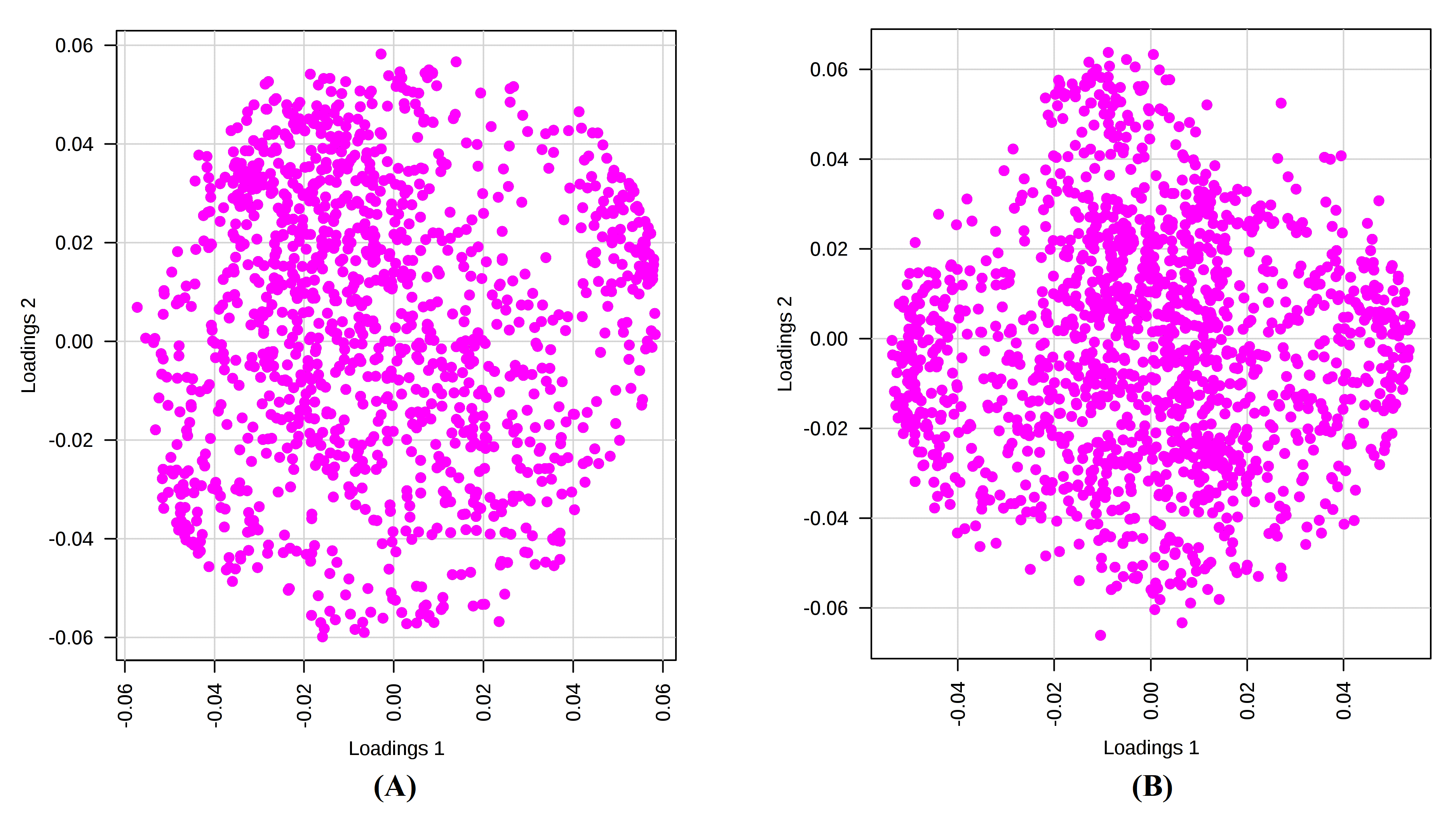
**

**Figure S1.** The PCA loading plots for the metabolomic data of muscle samples from female (A) and male (B) *C. idellus*.

**Table S1.** List of Discriminating Metabolites between Female-Artificial feed feeding group (FAF) and Female-Grass feeding group (FGF) *Ctenopharyngodon idellus*.

| **RT_Mass** | **Adducts** | **ID** | **Description** | **Mass.Diff** | **p.value** | **FDR** | **FC** | **Pathway** |
| --- | --- | --- | --- | --- | --- | --- | --- | --- |
| **9.37_280.2375m/z** | M[1+] | C01595 | Linoleic acid | 0.002700000 | 0.0000 | 0.0000 | 0.3137 | map00591 Linoleic acid metabolism; map01040 Biosynthesis of unsaturated fatty acids |
| **9.37_278.2222n** | M(C13)+H[1+] | C16300 | Stearidonic acid | 0.002623533 | 0.0000 | 0.0000 | 0.4268 | map00592 alpha-Linolenic acid metabolism |
| **8.93_369.2050m/z** | M(S34)-H[-] | C05956 | Prostaglandin G2 | 0.003423533 | 0.0000 | 0.0000 | 0.4319 | map00590 Arachidonic acid metabolism |
|  | M(S34)-H[-] | C05962 | 6-Ketoprostaglandin E1 | 0.003423533 | 0.0000 | 0.0000 | 0.4319 | map00590 Arachidonic acid metabolism |
|  | M(S34)-H[-] | C05964 | 11-Dehydro-thromboxane B2 | 0.003423533 | 0.0000 | 0.0000 | 0.4319 | map00590 Arachidonic acid metabolism |
| **9.37_243.2118m/z** | M-HCOOH+H[1+] | C00535 | Testosterone | 0.001023533 | 0.0000 | 0.0000 | 0.4794 | map00140 Steroid hormone biosynthesis; map00984 Steroid degradation |
|  | M-HCOOH+H[1+] | C01227 | Dehydroepiandrosterone | 0.001023533 | 0.0000 | 0.0000 | 0.4794 | map00140 Steroid hormone biosynthesis; map00984 Steroid degradation |
|  | M-HCOOH+H[1+] | C03772 | Etiocholanedione | 0.001023533 | 0.0000 | 0.0000 | 0.4794 | map00140 Steroid hormone biosynthesis |
| **9.37_204.1522n** | M-H4O2+H[1+] | C04635 | 1-(1-Alkenyl)-sn-glycero-3-phosphoethanolamine | 0.001123533 | 0.0000 | 0.0000 | 0.4157 | map00565 Ether lipid metabolism |
| **8.94_417.1350m/z** | M+Br[-] | C04748 | Protein C-terminal S-farnesyl-L-cysteine methyl ester | 0.001300000 | 0.0000 | 0.0000 | 0.4069 | map00900 Terpenoid backbone biosynthesis |
| **8.93_505.1806m/z** | M+Cl[-] | C04549 | 1-Phosphatidyl-1D-myo-inositol 3-phosphate | 0.002700000 | 0.0000 | 0.0000 | 0.4827 | map00562 Inositol phosphate metabolism; map04070 Phosphatidylinositol signaling system |
| **8.97_345.2049m/z** | M+CH3COO[-] | C00280 | Androstenedione | 0.001695000 | 0.0000 | 0.0000 | 0.3918 | map00140 Steroid hormone biosynthesis; map00984 Steroid degradation |
| **9.37_227.0385m/z** | M+HCOONa[1+] | C02350 | (S)(+)-Allantoin | 0.000176467 | 0.0000 | 0.0000 | 0.4969 | map00230 Purine metabolism |
| **9.37_123.1180m/z** | M-CO+H[1+] | C02576 | Perillyl aldehyde | 0.001223533 | 0.0000 | 0.0000 | 0.3947 | map00903 Limonene and pinene degradation |
| **9.37_333.1511m/z** | M-H4O2+H[1+] | C04555 | Dehydroepiandrosterone sulfate | 0.000676467 | 0.0000 | 0.0000 | 0.4355 | map00140 Steroid hormone biosynthesis |
| **8.93_347.2213m/z** | M+HCOO[-] | C06428 | Eicosapentaenoic acid | 0.000945000 | 0.0000 | 0.0000 | 0.4217 | map01040 Biosynthesis of unsaturated fatty acids |
| **8.97_277.2173m/z** | M-H[-] | C06427 | Alpha-Linolenic acid | 0.000023533 | 0.0000 | 0.0000 | 0.3403 | map00592 alpha-Linolenic acid metabolism; map01040 Biosynthesis of unsaturated fatty acids |
| **10.16_350.2826n** | M(S34)+H[1+] | C11695 | Anandamide | 0.002876467 | 0.0000 | 0.0000 | 0.2813 | map04080 Neuroactive ligand-receptor interaction |
| **9.37_281.0868m/z** | M-HCOOK+H[1+] | C00655 | Xanthylic acid | 0.001176467 | 0.0000 | 0.0000 | 0.4893 | map00230 Purine metabolism; map01065 Biosynthesis of alkaloids derived from histidine and purine |
| **8.94_301.2175m/z** | M-H+O[-] | C00473 | Vitamin A | 0.000166467 | 0.0000 | 0.0000 | 0.4077 | map00830 Retinol metabolism |
| **9.54_349.2740m/z** | M+H2O+H[1+] | C16513 | Docosapentaenoic acid | 0.000223533 | 0.0000 | 0.0000 | 0.4267 | map01040 Biosynthesis of unsaturated fatty acids |
| **9.69_323.2571m/z** | M-CO+H[1+] | C05476 | Tetrahydrocorticosterone | 0.000876467 | 0.0000 | 0.0000 | 0.3846 | map00140 Steroid hormone biosynthesis |
| **9.36_443.2060m/z** | M-H4O2+H[1+] | C11131 | 2-Methoxy-estradiol-17b 3-glucuronide | 0.000376467 | 0.0000 | 0.0000 | 0.4545 | map00140 Steroid hormone biosynthesis |
| **8.93_257.2272m/z** | M(S34)-H[-] | C00249 | Palmitic acid | 0.001523533 | 0.0000 | 0.0000 | 0.4297 | map00062 Fatty acid elongation; map00071 Fatty acid degradation; map01040 Biosynthesis of unsaturated fatty acids; map01212 Fatty acid metabolism |
| **9.97_445.2516m/z** | M+Cl37[-] | C00695 | Cholic acid | 0.001900000 | 0.0000 | 0.0000 | 2.0955 | map00120 Primary bile acid biosynthesis |
| **9.69_449.1999m/z** | M(Cl37)-H[-] | C05503 | 17-beta-Estradiol-3-glucuronide | 0.000276467 | 0.0000 | 0.0000 | 0.4155 | map00140 Steroid hormone biosynthesis |
| **8.85_683.3352m/z** | M+ACN-H[-] | C11304 | S-(PGA1)-glutathione | 0.002031467 | 0.0000 | 0.0000 | 2.0319 |  |
| **9.97_439.2356m/z** | M(C13)-H[-] | C05952 | Leukotriene E4 | 0.000276467 | 0.0000 | 0.0000 | 2.5133 | map00590 Arachidonic acid metabolism |
| **9.55_400.1492m/z** | M+HCOO[-] | C01137 | S-Adenosylmethioninamine | 0.003645000 | 0.0000 | 0.0000 | 2.4926 | map00270 Cysteine and methionine metabolism; map00330 Arginine and proline metabolism |
| **8.99_549.1676m/z** | M(S34)-H[-] | C04637 | Phosphatidylinositol-4,5-bisphosphate | 0.000676467 | 0.0000 | 0.0000 | 0.4606 | map00562 Inositol phosphate metabolism; map04070 Phosphatidylinositol signaling system; map04072 Phospholipase D signaling pathway; map04151 PI3K-Akt signaling pathway; map04725 Cholinergic synapse; map04810 Regulation of actin cytoskeleton |
| **9.39_361.2719m/z** | M-H2O+H[1+] | C13856 | 2-Arachidonylglycerol | 0.001776467 | 0.0000 | 0.0000 | 0.4186 | map04080 Neuroactive ligand-receptor interaction |
| **8.34_714.2561m/z** | M+ACN-H[-] | C04886 | (N-acetylneuraminosyl(a2-6)lactosamine) | 0.001368533 | 0.0000 | 0.0000 | 0.3358 |  |
| **9.55_496.1222m/z** | M+ACN-H[-] | C00061 | Flavin Mononucleotide | 0.001668533 | 0.0000 | 0.0000 | 2.1552 | map00190 Oxidative phosphorylation; map00740 Riboflavin metabolism |
| **9.36_533.4644n** | M+K[1+] | G00035 | Sialyl-Tn antigen | 0.001623533 | 0.0000 | 0.0000 | 0.4453 | map00512 Mucin type O-glycan biosynthesis |
| **9.97_507.2250m/z** | M+CH3COO[-] | C05503 | 17-beta-Estradiol-3-glucuronide | 0.002005000 | 0.0000 | 0.0000 | 2.7953 | map00140 Steroid hormone biosynthesis |
| **9.55_396.1967m/z** | M(C13)-H[-] | C18044 | 3beta-Hydroxypregn-5-en-20-one sulfate | 0.003576467 | 0.0000 | 0.0000 | 2.0027 | map00140 Steroid hormone biosynthesis |
| **9.41_285.2587m/z** | M(S34)-H[-] | C01530 | Stearic acid | 0.001323533 | 0.0000 | 0.0000 | 2.2325 | map01040 Biosynthesis of unsaturated fatty acids |
| **9.96_377.2680m/z** | M+HCOO[-] | C16527 | Adrenic acid | 0.001145000 | 0.0000 | 0.0000 | 2.7913 | map01040 Biosynthesis of unsaturated fatty acids |
| **9.41_311.2379m/z** | M-H2O-H[-] | C16513 | Docosapentaenoic acid | 0.000123533 | 0.0000 | 0.0000 | 2.5819 | map01040 Biosynthesis of unsaturated fatty acids |
| **11.69_688.5872m/z** | M-H[-] | G00094 | Globoside | 0.005523533 | 0.0000 | 0.0000 | 0.0637 | map00603 Glycosphingolipid biosynthesis |
| **9.99_365.3030m/z** | M-CO+H[1+] | C02528 | Chenodeoxycholic acid | 0.001976467 | 0.0000 | 0.0000 | 2.8076 | map00120 Primary bile acid biosynthesis |
| **9.36_147.1180m/z** | M(S34)+H[1+] | C06423 | Caprylic acid | 0.000076467 | 0.0000 | 0.0000 | 0.4958 | map00061 Fatty acid biosynthesis; map01066 Biosynthesis of alkaloids derived from terpenoid and polyketide |
| **8.59_588.5224m/z** | M(S34)-H[-] | C04500 | N-Acetyl-D-glucosaminyldiphosphodolichol | 0.000623533 | 0.0000 | 0.0000 | 5.6192 | Lipids |
| **8.71_299.2015m/z** | M-H2O-H[-] | C00909 | Leukotriene A4 | 0.000123533 | 0.0000 | 0.0000 | 0.4558 | map00590 Arachidonic acid metabolism |
|  | M-H2O-H[-] | C14732 | 5-KETE | 0.000123533 | 0.0000 | 0.0000 | 0.4558 | map00590 Arachidonic acid metabolism |
| **11.72_430.3817m/z** | M[1+] | C02477 | Alpha-Tocopherol | 0.000600000 | 0.0000 | 0.0000 | 0.2414 | map00130 Ubiquinone and other terpenoid-quinone biosynthesis; map04977 Vitamin digestion and absorption |
| **9.96_496.2127m/z** | M+Na-2H[-] | C11061 | Retinoyl b-glucuronide | 0.004252934 | 0.0000 | 0.0000 | 2.7578 | map00830 Retinol metabolism |
| **10.37_467.2677m/z** | M+Br81[-] | C00187 | Cholesterol | 0.003500000 | 0.0000 | 0.0000 | 5.1926 | map00120 Primary bile acid biosynthesis; map00140 Steroid hormone biosynthesis; map01066 Biosynthesis of alkaloids derived from terpenoid and polyketide; map04975 Fat digestion and absorption; map04977 Vitamin digestion and absorption; map04979 Cholesterol metabolism |
| **9.97_247.2428m/z** | M-CO2+H[1+] | C00167 | Uridine diphosphate glucuronic acid | 0.000723533 | 0.0000 | 0.0000 | 2.1033 | map00040 Pentose and glucuronate interconversions; map00520 Amino sugar and nucleotide sugar metabolism |
|  | M-CO2+H[1+] | C00523 | Androsterone | 0.000723533 | 0.0000 | 0.0000 | 2.1033 | map00140 Steroid hormone biosynthesis |
|  | M-CO2+H[1+] | C03917 | Dihydrotestosterone | 0.000723533 | 0.0000 | 0.0000 | 2.1033 | map00140 Steroid hormone biosynthesis |
|  | M-CO2+H[1+] | C04295 | 5-Androstenediol | 0.000723533 | 0.0000 | 0.0000 | 2.1033 | map00140 Steroid hormone biosynthesis |
|  | M-CO2+H[1+] | C04373 | Etiocholanolone | 0.000723533 | 0.0000 | 0.0000 | 2.1033 | map00140 Steroid hormone biosynthesis |
| **9.86_295.2427m/z** | M-H4O2+H[1+] | C16513 | Docosapentaenoic acid | 0.000723533 | 0.0000 | 0.0000 | 2.1815 | map01040 Biosynthesis of unsaturated fatty acids |
| **9.81_375.1817m/z** | M-C3H4O2+H[1+] | C11133 | Estrone glucuronide | 0.001423533 | 0.0000 | 0.0000 | 0.4882 | map00140 Steroid hormone biosynthesis |
| **7.80_422.3272m/z** | M+Na[1+] | C02990 | L-Palmitoylcarnitine | 0.003023533 | 0.0000 | 0.0000 | 0.2499 | map00071 Fatty acid degradation; map01212 Fatty acid metabolism |
| **8.35_692.2700m/z** | M(C13)-H[-] | C00369 | Starch | 0.000123533 | 0.0000 | 0.0000 | 0.4039 | map00500 Starch and sucrose metabolism |
| **8.93_943.6062m/z** | M+Br81[-] | C11378 | Coenzyme Q10 | 0.006000000 | 0.0000 | 0.0000 | 0.0433 | map04216 Ferroptosis |
| **8.94_283.2061m/z** | M-H[-] | C00376 | Retinal | 0.000623533 | 0.0000 | 0.0000 | 0.4549 | map00830 Retinol metabolism |
| **8.99_335.1760m/z** | M+Cl[-] | C00777 | Retinoic acid | 0.001800000 | 0.0000 | 0.0000 | 0.3513 | map00830 Retinol metabolism |
| **9.04_352.2613n** | M-CO+H[1+] | C06124 | Sphingosine 1-phosphate | 0.000223533 | 0.0000 | 0.0000 | 0.4019 | map00600 Sphingolipid metabolism |
| **9.55_440.1357m/z** | M+Br[-] | C03819 | 1-Acylglycerophosphoinositol | 0.000400000 | 0.0000 | 0.0000 | 2.5012 | map00564 Glycerophospholipid metabolism |
| **7.36_277.2171m/z** | M-H2O-H[-] | C14762 | 13S-hydroxyoctadecadienoic acid | 0.000123533 | 0.0000 | 0.0000 | 0.2522 | map00591 Linoleic acid metabolism; map03320 PPAR signaling pathway |
| **8.48_240.1001m/z** | M-HCOOK+H[1+] | C00055 | Cytidine monophosphate | 0.002223533 | 0.0000 | 0.0001 | 0.4610 | map00240 Pyrimidine metabolism |
|  | M-HCOOK+H[1+] | C00165 | Diacylglycerol | 0.002223533 | 0.0000 | 0.0001 | 0.4610 | map04066 HIF-1 signaling pathway; map04918 Thyroid hormone synthesis; map04923 Regulation of lipolysis in adipocytes; map04975 Fat digestion and absorption |
|  | M-HCOOK+H[1+] | C01290 | Lactosylceramide | 0.002223533 | 0.0000 | 0.0001 | 0.4610 | map00600 Sphingolipid metabolism |
|  | M-HCOOK+H[1+] | C04776 | T antigen | 0.002223533 | 0.0000 | 0.0001 | 0.4610 | map00512 Mucin type O-glycan biosynthesis |
| **8.62_549.2955m/z** | M-H2O+H[1+] | C00052 | Uridine diphosphategalactose | 0.002976467 | 0.0000 | 0.0001 | 0.4709 | map00052 Galactose metabolism; map00520 Amino sugar and nucleotide sugar metabolism |
| **8.34_414.3582m/z** | M-NH3+H[1+] | C02477 | Alpha-Tocopherol | 0.003676467 | 0.0000 | 0.0001 | 0.3857 | map00130 Ubiquinone and other terpenoid-quinone biosynthesis; map04977 Vitamin digestion and absorption |
| **9.04_313.0948m/z** | M+H[1+] | C00157 | Phosphatidylcholine | 0.002523533 | 0.0000 | 0.0001 | 0.2284 | map00564 Glycerophospholipid metabolism; map00590 Arachidonic acid metabolism; map00591 Linoleic acid metabolism; map00592 alpha-Linolenic acid metabolism |
| **8.50_335.2587m/z** | M+H2O+H[1+] | C01953 | Pregnenolone | 0.000623533 | 0.0000 | 0.0001 | 0.3647 | map00140 Steroid hormone biosynthesis; map04925 Aldosterone synthesis and secretion |
| **8.69_496.1751m/z** | M+Na-2H[-] | C11132 | 2-Methoxyestrone 3-glucuronide | 0.003052934 | 0.0000 | 0.0002 | 0.4404 | map00140 Steroid hormone biosynthesis |
| **9.92_271.2429m/z** | M-H4O2+H[1+] | C03242 | 8,11,14-Eicosatrienoic acid | 0.000923533 | 0.0000 | 0.0002 | 2.1821 | map00591 Linoleic acid metabolism; map01040 Biosynthesis of unsaturated fatty acids |
| **8.22_319.2279m/z** | M-H+O[-] | C00219 | Arachidonic acid | 0.000066467 | 0.0000 | 0.0002 | 3.5723 | map00590 Arachidonic acid metabolism; map00591 Linoleic acid metabolism; map01040 Biosynthesis of unsaturated fatty acids |
| **8.35_647.2144m/z** | M+Br81[-] | C00029 | Uridine diphosphate glucose | 0.003700000 | 0.0000 | 0.0002 | 0.4508 | map00040 Pentose and glucuronate interconversions; map00052 Galactose metabolism; map00500 Starch and sucrose metabolism; map00520 Amino sugar and nucleotide sugar metabolism; map00561 Glycerolipid metabolism |
| **8.35_712.2450m/z** | M+Na-2H[-] | C00369 | Starch | 0.003552934 | 0.0000 | 0.0002 | 0.4416 | map00500 Starch and sucrose metabolism |
| **8.63_477.2161n** | M+H[1+] | C11132 | 2-Methoxyestrone 3-glucuronide | 0.004223533 | 0.0000 | 0.0002 | 0.3273 | map00140 Steroid hormone biosynthesis |
| **11.06_810.5628m/z** | M(Cl37)-H[-] | C00040 | Acyl-CoA | 0.001876467 | 0.0000 | 0.0002 | 0.4411 | map00062 Fatty acid elongation; map00561 Glycerolipid metabolism; map00564 Glycerophospholipid metabolism; map04922 Glucagon signaling pathway; map04975 Fat digestion and absorption |
| **8.44_345.2439m/z** | M+CH3COO[-] | C00899 | 11-cis-Retinol | 0.000905000 | 0.0000 | 0.0002 | 3.2567 | map00830 Retinol metabolism |
|  | M+CH3COO[-] | C16682 | 9-cis-Retinol | 0.000905000 | 0.0000 | 0.0002 | 3.2567 | map00830 Retinol metabolism |
| **9.37_269.6435m/z** | M+H+Na[2+] | C15516 | Taurohyocholate | 0.000626467 | 0.0000 | 0.0003 | 3.4145 |  |
| **8.50_532.1983m/z** | M(C13)+2H[2+] | C07297 | 3-Oxopristanoyl-CoA | 0.003773533 | 0.0000 | 0.0004 | 0.2475 |  |
| **8.50_475.2707n** | M-HCOOH+H[1+] | C00621 | Dolichyl diphosphate | 0.003176467 | 0.0000 | 0.0004 | 0.2804 | map00510 N-Glycan biosynthesis; map00900 Terpenoid backbone biosynthesis |
| **8.25_426.3586m/z** | M+H+Na[2+] | G00343 | Maltopentaose | 0.001173533 | 0.0001 | 0.0005 | 2.2398 |  |
| **8.62_556.7798m/z** | M-HCOONa+H[1+] | HMDB12993 | Leukotriene C5 | 0.001923533 | 0.0001 | 0.0005 | 0.2669 |  |
| **9.26_393.2645m/z** | M-H+O[-] | C13856 | 2-Arachidonylglycerol | 0.000133533 | 0.0001 | 0.0005 | 3.3809 | map04080 Neuroactive ligand-receptor interaction |
| **8.63_335.2584m/z** | M+H2O+H[1+] | C01953 | Pregnenolone | 0.000323533 | 0.0001 | 0.0006 | 0.3822 | map00140 Steroid hormone biosynthesis |
| **10.42_434.3409m/z** | M[1+] | C01301 | 3a,7a,12a-Trihydroxy-5b-cholestan-26-al | 0.001300000 | 0.0001 | 0.0009 | 3.2825 | map00120 Primary bile acid biosynthesis |
| **8.56_347.2596m/z** | M+CH3COO[-] | C15492 | All-trans-13,14-dihydroretinol | 0.001005000 | 0.0001 | 0.0011 | 3.1542 | map00830 Retinol metabolism |
| **9.99_261.2216m/z** | M-C3H4O2+H[1+] | C05138 | 17a-Hydroxypregnenolone | 0.000323533 | 0.0002 | 0.0011 | 0.1884 | map00140 Steroid hormone biosynthesis |
| **9.20_403.2098m/z** | M+CH3COO[-] | C05490 | 11-Dehydrocorticosterone | 0.002295000 | 0.0002 | 0.0015 | 2.0519 | map00140 Steroid hormone biosynthesis |
| **9.53_461.2211m/z** | M-H2O+H[1+] | C11131 | 2-Methoxy-estradiol-17b 3-glucuronide | 0.004123533 | 0.0003 | 0.0020 | 2.3475 | map00140 Steroid hormone biosynthesis |
| **9.64_660.5101m/z** | M+Na[1+] | C00925 | Heparan sulfate | 0.001176467 | 0.0003 | 0.0022 | 2.1154 | map00531 Glycosaminoglycan degradation |
| **10.90_780.4798m/z** | M+Br81[-] | C02686 | Galactosylceramide | 0.001500000 | 0.0005 | 0.0034 | 0.2193 | map00600 Sphingolipid metabolism |
| **11.06_303.2326m/z** | M-H[-] | C00219 | Arachidonic acid | 0.000123533 | 0.0006 | 0.0039 | 2.4364 | map00590 Arachidonic acid metabolism; map00591 Linoleic acid metabolism; map01040 Biosynthesis of unsaturated fatty acids |
| **11.37_637.5644m/z** | M(C13)+2H[2+] | C06140 | GT1b | 0.005423533 | 0.0009 | 0.0052 | 2.7712 | map00604 Glycosphingolipid biosynthesis |
| **9.57_635.5003m/z** | M(Cl37)-H[-] | C00195 | N-Acylsphingosine | 0.005376467 | 0.0009 | 0.0055 | 2.7227 | map00600 Sphingolipid metabolism |
|  | M(Cl37)-H[-] | C02960 | Ceramide 1-phosphate | 0.005376467 | 0.0009 | 0.0055 | 2.7227 | map00600 Sphingolipid metabolism |
| **8.97_323.2226m/z** | M+HCOO[-] | C06426 | Gamma-Linolenic acid | 0.000355000 | 0.0010 | 0.0060 | 0.2608 | map00591 Linoleic acid metabolism; map01040 Biosynthesis of unsaturated fatty acids |
| **9.04_208.1113m/z** | M+NaCl[1+] | C05578 | 5,6-Dihydroxyindole | 0.001276467 | 0.0012 | 0.0066 | 0.4928 | map00350 Tyrosine metabolism |
| **8.63_499.2714n** | M+NaCl[1+] | C00110 | dolichyl phosphate | 0.000476467 | 0.0015 | 0.0085 | 0.4522 | map00510 N-Glycan biosynthesis |
| **8.63_475.2712n** | M-HCOOH+H[1+] | C00621 | Dolichyl diphosphate | 0.002676467 | 0.0016 | 0.0089 | 0.3068 | map00510 N-Glycan biosynthesis; map00900 Terpenoid backbone biosynthesis |
| **8.86_329.2486m/z** | M-H[-] | C16513 | Docosapentaenoic acid | 0.000023533 | 0.0017 | 0.0095 | 2.1767 | map01040 Biosynthesis of unsaturated fatty acids |
| **9.54_427.2832m/z** | M+HCOONa[1+] | HMDB06322 | Tetracosapentaenoic acid | 0.001523533 | 0.0018 | 0.0096 | 2.5131 |  |
| **8.69_451.1790m/z** | M(S34)-H[-] | C11356 | Geranylneryl diphosphate | 0.003123533 | 0.0022 | 0.0115 | 0.2499 | map01062 Biosynthesis of terpenoids and steroids |
| **6.37_334.2955m/z** | M+2H[2+] | C00464 | Mannan | 0.001276467 | 0.0028 | 0.0142 | 0.4904 | map00051 Fructose and mannose metabolism |
| **8.37_319.2284m/z** | M-H[-] | C04742 | 15(S)-HETE | 0.000576467 | 0.0034 | 0.0167 | 2.5865 | map00590 Arachidonic acid metabolism |
|  | M-H[-] | C04805 | 5-HETE | 0.000576467 | 0.0034 | 0.0167 | 2.5865 | map00590 Arachidonic acid metabolism |
|  | M-H[-] | C14749 | 19(S)-HETE | 0.000576467 | 0.0034 | 0.0167 | 2.5865 | map00590 Arachidonic acid metabolism |
|  | M-H[-] | C14768 | 5,6-Epoxy-8,11,14-eicosatrienoic acid | 0.000576467 | 0.0034 | 0.0167 | 2.5865 | map00590 Arachidonic acid metabolism |
|  | M-H[-] | C14769 | 8,9-Epoxyeicosatrienoic acid | 0.000576467 | 0.0034 | 0.0167 | 2.5865 | map00590 Arachidonic acid metabolism |
|  | M-H[-] | C14770 | 11,12-Epoxyeicosatrienoic acid | 0.000576467 | 0.0034 | 0.0167 | 2.5865 | map00590 Arachidonic acid metabolism |
|  | M-H[-] | C14771 | 14,15-Epoxy-5,8,11-eicosatrienoic acid | 0.000576467 | 0.0034 | 0.0167 | 2.5865 | map00590 Arachidonic acid metabolism |
|  | M-H[-] | C14776 | 8-HETE | 0.000576467 | 0.0034 | 0.0167 | 2.5865 | map00590 Arachidonic acid metabolism; map03320 PPAR signaling pathway |
|  | M-H[-] | C14778 | 16(R)-HETE | 0.000576467 | 0.0034 | 0.0167 | 2.5865 | map00590 Arachidonic acid metabolism |
|  | M-H[-] | C14779 | 9(S)-HETE | 0.000576467 | 0.0034 | 0.0167 | 2.5865 | map00590 Arachidonic acid metabolism |
| **7.58_303.2327m/z** | M-H[-] | C00219 | Arachidonic acid | 0.000023533 | 0.0039 | 0.0194 | 2.7011 | map00590 Arachidonic acid metabolism; map00591 Linoleic acid metabolism |
| **11.91_1160.3458m/z** | M+HCOOK[1+] | HMDB62369 | 3-Oxoeicosanoyl-CoA | 0.009476467 | 0.0042 | 0.0205 | 3.1574 |  |
| **10.43_495.5046n** | M+H[1+] | G00035 | Sialyl-Tn antigen | 0.002676467 | 0.0085 | 0.0390 | 2.0541 | map00512 Mucin type O-glycan biosynthesis |

**Table S2.** List of Discriminating Muscle Metabolites between MAG and MGG *C. idellus*.

| **RT-Peaks** | **Matched.Form** | **ID** | **Description** | **Mass.Diff** | **p.value** | **FDR** | **FC** | **Pathway** |
| --- | --- | --- | --- | --- | --- | --- | --- | --- |
| **10.45_710.5450n** | M+K-2H[-] | C04886 | (N-acetylneuraminosyl(a2-6)lactosamine) | 0.004052934 | 0.0000 | 0.0000 | 6.8262 |  |
| **9.28_319.1888m/z** | M-H+O[-] | C05139 | 16a-Hydroxydehydroisoandrosterone | 0.002633533 | 0.0000 | 0.0001 | 5.4296 | map00140 Steroid hormone biosynthesis |
|  | M-H+O[-] | C05294 | 19-Hydroxytestosterone | 0.002633533 | 0.0000 | 0.0001 | 5.4296 | map00140 Steroid hormone biosynthesis |
|  | M-H+O[-] | C18045 | 7a-Hydroxydehydroepiandrosterone | 0.002633533 | 0.0000 | 0.0001 | 5.4296 | map00140 Steroid hormone biosynthesis |
|  | M-H+O[-] | C18075 | 11beta,17beta-Dihydroxy-4-androsten-3-one | 0.002633533 | 0.0000 | 0.0001 | 5.4296 | map00140 Steroid hormone biosynthesis |
| **9.92_633.4865m/z** | M(S34)-H[-] | G00157 | (Gal)2 (GlcA)1 (Xyl)1 (Ser)1 | 0.002023533 | 0.0000 | 0.0000 | 4.8402 | map00534 Glycosaminoglycan biosynthesis |
| **8.83_345.2429m/z** | M+CH3COO[-] | C00473 | Vitamin A | 0.000095000 | 0.0017 | 0.0080 | 4.7645 | map00830 Retinol metabolism |
| **9.23_522.5857m/z** | M+H+Na[2+] | C14119 | Naphthyl-2-oxomethyl-succinyl-CoA | 0.000873533 | 0.0000 | 0.0000 | 4.3115 | map00626 Naphthalene degradation; map01220 Degradation of aromatic compounds |
| **8.96_347.2595m/z** | M+CH3COO[-] | C15492 | All-trans-13,14-dihydroretinol | 0.000905000 | 0.0019 | 0.0087 | 4.1931 | map00830 Retinol metabolism |
| **8.59_387.2151m/z** | M-H[-] | C01177 | Inositol phosphate | 0.003376467 | 0.0002 | 0.0011 | 3.7549 | map00562 Inositol phosphate metabolism; map04070 Phosphatidylinositol signaling system |
|  | M-H[-] | C01194 | 1-Phosphatidyl-D-myo-inositol | 0.003376467 | 0.0002 | 0.0011 | 3.7549 | map00562 Inositol phosphate metabolism; map00563 Glycosylphosphatidylinositol (GPI)-anchor biosynthesis; map00564 Glycerophospholipid metabolism; map04070 Phosphatidylinositol signaling system |
| **12.17_437.7842m/z** | M-HCOONa+H[1+] | HMDB06455 | Arachidonyl carnitine | 0.000476467 | 0.0000 | 0.0000 | 3.7082 |  |
| **10.46_1070.9030m/z** | M+HCOONa[1+] | HMDB12473 | (3S)-3-Hydroxy-cis,cis-palmito-7,10-dienoyl-CoA | 0.002676467 | 0.0012 | 0.0060 | 3.7041 |  |
| **8.59_319.2274m/z** | M+Na-2H[-] | C04230 | LysoPC(14:0) | 0.000047066 | 0.0001 | 0.0004 | 3.4579 | map00564 Glycerophospholipid metabolism |
| **9.29_252.2095n** | M+NaCl[1+] | HMDB03892 | (S)-N-Methylsalsolinol | 0.001323533 | 0.0000 | 0.0000 | 3.1798 |  |
| **9.35_275.2359m/z** | M-CO2+H[1+] | C00909 | Leukotriene A4 | 0.001076467 | 0.0005 | 0.0029 | 2.9768 | map00590 Arachidonic acid metabolism |
| **10.45_377.2668m/z** | M+HCOO[-] | C16527 | Adrenic acid | 0.002345000 | 0.0000 | 0.0000 | 2.9507 | map01040 Biosynthesis of unsaturated fatty acids |
| **10.69_323.2954m/z** | M-H[-] | G00115 | GD1b | 0.002676467 | 0.0000 | 0.0000 | 2.7411 | map00604 Glycosphingolipid biosynthesis |
| **9.14_533.2639m/z** | M-H4O2+H[1+] | C06462 | Leukotriene F4 | 0.003976467 | 0.0000 | 0.0000 | 2.6614 | map00590 Arachidonic acid metabolism |
| **9.23_478.5050m/z** | M+Cl37[-] | HMDB12607 | 18-Carboxy-dinor-LTE4 | 0.001100000 | 0.0000 | 0.0002 | 2.6097 |  |
|  | M+Br81[-] | HMDB06317 | trans-Hexadec-2-enoyl carnitine | 0.003100000 | 0.0000 | 0.0002 | 2.6097 |  |
| **8.85_321.2435m/z** | M-H+O[-] | C03242 | 8,11,14-Eicosatrienoic acid | 0.000033533 | 0.0073 | 0.0294 | 2.5842 | map00591 Linoleic acid metabolism; map01040 Biosynthesis of unsaturated fatty acids |
| **10.45_649.2190m/z** | M+Br81[-] | C01079 | Protoporphyrinogen IX | 0.002300000 | 0.0000 | 0.0001 | 2.5169 | map00860 Porphyrin and chlorophyll metabolism |
| **9.39_269.6435m/z** | M+H+Na[2+] | C15516 | Taurohyocholate | 0.000626467 | 0.0000 | 0.0001 | 2.4845 |  |
| **9.67_726.3020m/z** | M(S34)+H[1+] | C02090 | Trypanothione | 0.005476467 | 0.0000 | 0.0000 | 2.4809 | map00480 Glutathione metabolism |
| **0.59_124.0062m/z** | M-H[-] | C00245 | Taurine | 0.001223533 | 0.0027 | 0.0123 | 2.3985 | map00120 Primary bile acid biosynthesis; map00430 Taurine and hypotaurine metabolism |
| **11.39_637.5638m/z** | M(C13)+2H[2+] | G00114 | GD2 | 0.004823533 | 0.0066 | 0.0269 | 2.3706 | map00604 Glycosphingolipid biosynthesis |
|  | M(C13)+2H[2+] | G00116 | GT1b | 0.004823533 | 0.0066 | 0.0269 | 2.3706 | map00604 Glycosphingolipid biosynthesis |
| **9.63_613.4801m/z** | M-H2O-H[-] | G00157 | (Gal)2 (GlcA)1 (Xyl)1 (Ser)1 | 0.002023533 | 0.0000 | 0.0001 | 2.3648 | map00534 Glycosaminoglycan biosynthesis |
| **0.59_126.0226m/z** | M-CO2+H[1+] | C00506 | Cysteic acid | 0.000623533 | 0.0016 | 0.0078 | 2.3518 | map00270 Cysteine and methionine metabolism; map00430 Taurine and hypotaurine metabolism |
|  | M-CO+H[1+] | C00606 | 3-Sulfinoalanine | 0.000723533 | 0.0016 | 0.0078 | 2.3518 | map00270 Cysteine and methionine metabolism; map00430 Taurine and hypotaurine metabolism |
| **9.94_580.3987m/z** | M+Br[-] | C06023 | D-Glucosaminide | 0.002400000 | 0.0021 | 0.0098 | 2.3227 | map00520 Amino sugar and nucleotide sugar metabolism |
| **9.92_373.2351m/z** | M+HCOO[-] | C06429 | Docosahexaenoic acid | 0.002745000 | 0.0000 | 0.0000 | 2.3056 | map01040 Biosynthesis of unsaturated fatty acids |
| **9.16_517.0267m/z** | M-H2O-H[-] | C00190 | UDP-D-Xylose | 0.000076467 | 0.0000 | 0.0000 | 2.2752 | map00520 Amino sugar and nucleotide sugar metabolism |
| **10.46_496.2127m/z** | M+Na-2H[-] | C11061 | Retinoyl b-glucuronide | 0.004252934 | 0.0000 | 0.0000 | 2.2711 | map00830 Retinol metabolism |
|  | M+Na-2H[-] | C11061 | Retinoyl b-glucuronide | 0.004252934 | 0.0000 | 0.0000 | 2.2711 | map00830 Retinol metabolism |
| **10.45_407.2097m/z** | M+HCOO[-] | C00735 | Hydrocortisone | 0.002755000 | 0.0000 | 0.0000 | 2.2513 | map00140 Steroid hormone biosynthesis; map04960 Aldosterone-regulated sodium reabsorption; map04976 Bile secretion |
| **9.38_526.5734m/z** | M+Br[-] | C00219 | Arachidonic acid | 0.004900000 | 0.0016 | 0.0079 | 2.2238 | map00590 Arachidonic acid metabolism; map00591 Linoleic acid metabolism; map01040 Biosynthesis of unsaturated fatty acids |
| **10.45_507.2261m/z** | M+CH3COO[-] | C05503 | 17-beta-Estradiol-3-glucuronide | 0.003105000 | 0.0000 | 0.0000 | 2.2067 | map00140 Steroid hormone biosynthesis; map04976 Bile secretion |
| **10.14_637.5188m/z** | M(C13)-H[-] | C00925 | Heparan sulfate | 0.000676467 | 0.0013 | 0.0065 | 2.1803 | map00531 Glycosaminoglycan degradation |
| **10.98_588.8992m/z** | M+Br81[-] | C00081 | Inosine triphosphate | 0.003100000 | 0.0000 | 0.0000 | 2.1422 | map00230 Purine metabolism |
| **9.49_508.5955m/z** | M+HCOOK[1+] | HMDB06461 | Linoelaidyl carnitine | 0.002176467 | 0.0003 | 0.0018 | 2.0908 |  |
|  | M+HCOOK[1+] | HMDB06469 | Linoleyl carnitine | 0.002176467 | 0.0003 | 0.0018 | 2.0908 |  |
| **9.93_402.1905m/z** | M-CO2+H[1+] | C00101 | Tetrahydrofolic acid | 0.002023533 | 0.0000 | 0.0000 | 2.0720 | map00260 Glycine, serine and threonine metabolism; map00790 Folate biosynthesis |
| **9.86_285.2580m/z** | M+HCOO[-] | C00012 | Peptide | 0.001355000 | 0.0000 | 0.0001 | 2.0703 | map00330 Arginine and proline metabolism |
| **10.45_439.2356m/z** | M(C13)-H[-] | C05952 | Leukotriene E4 | 0.000276467 | 0.0000 | 0.0000 | 2.0568 | map00590 Arachidonic acid metabolism |
| **9.16_330.2518m/z** | M(C13)-H[-] | C16513 | Docosapentaenoic acid | 0.000223533 | 0.0000 | 0.0000 | 2.0545 | map01040 Biosynthesis of unsaturated fatty acids |
| **9.51_546.9469m/z** | M+K[1+] | C00081 | Inosine triphosphate | 0.004323533 | 0.0084 | 0.0332 | 2.0496 | map00230 Purine metabolism |
| **9.16_526.2939m/z** | M(S34)-H[-] | C02737 | PS(16:0/16:0) | 0.001623533 | 0.0000 | 0.0000 | 2.0264 | map00260 Glycine, serine and threonine metabolism; map00564 Glycerophospholipid metabolism |
| **9.17_523.1746m/z** | M(C13)+2H[2+] | C16327 | OPC8-CoA | 0.003573533 | 0.0000 | 0.0000 | 2.0004 | map00592 alpha-Linolenic acid metabolism |
|  | M+NaCl[1+] | C05504 | 16-Glucuronide-estriol | 0.004123533 | 0.0000 | 0.0000 | 2.0004 | map00140 Steroid hormone biosynthesis |
| **11.40_738.5099m/z** | M+Br81[-] | G00111 | GD1a | 0.002000000 | 0.0000 | 0.0000 | 0.4985 | map00604 Glycosphingolipid biosynthesis |
| **8.65_261.2216m/z** | M-CO2+H[1+] | C05139 | 16a-Hydroxydehydroisoandrosterone | 0.000323533 | 0.0000 | 0.0001 | 0.4971 | map00140 Steroid hormone biosynthesis |
|  | M-CO2+H[1+] | C05294 | 19-Hydroxytestosterone | 0.000323533 | 0.0000 | 0.0001 | 0.4971 | map00140 Steroid hormone biosynthesis |
|  | M-CO2+H[1+] | C18045 | 7a-Hydroxydehydroepiandrosterone | 0.000323533 | 0.0000 | 0.0001 | 0.4971 | map00140 Steroid hormone biosynthesis |
|  | M-CO2+H[1+] | C18075 | 11beta,17beta-Dihydroxy-4-androsten-3-one | 0.000323533 | 0.0000 | 0.0001 | 0.4971 | map00140 Steroid hormone biosynthesis |
| **9.35_283.2077m/z** | M-H2O-H[-] | C06428 | Eicosapentaenoic acid | 0.000976467 | 0.0000 | 0.0000 | 0.4963 | map01040 Biosynthesis of unsaturated fatty acids |
|  | M-H[-] | C00376 | Retinal | 0.000976467 | 0.0000 | 0.0000 | 0.4963 | map00830 Retinol metabolism |
| **9.04_121.1015m/z** | M+H+Na[2+] | C03740 | 5-L-Glutamyl-L-alanine | 0.000376467 | 0.0002 | 0.0010 | 0.4914 | map00480 Glutathione metabolism |
| **10.88_848.5268m/z** | M+K[1+] | C00040 | Acyl-CoA | 0.006976467 | 0.0000 | 0.0001 | 0.4832 | map00062 Fatty acid elongation; map00561 Glycerolipid metabolism; map00564 Glycerophospholipid metabolism; map04975 Fat digestion and absorption |
|  | M+K[1+] | C00165 | DG(16:0/16:0/0:0) | 0.006976467 | 0.0000 | 0.0001 | 0.4832 | map04066 HIF-1 signaling pathway; map04071 Sphingolipid signaling pathway; map04072 Phospholipase D signaling pathway; map04915 Estrogen signaling pathway; map04918 Thyroid hormone synthesis; map04925 Aldosterone synthesis and secretion; map04975 Fat digestion and absorption |
| **8.48_766.2810m/z** | M+HCOO[-] | C03170 | Trypanothione disulfide | 0.005345000 | 0.0000 | 0.0002 | 0.4824 | map00480 Glutathione metabolism |
| **12.26_429.3735m/z** | M+H2O+H[1+] | C15783 | 5-Dehydroavenasterol | 0.000723533 | 0.0000 | 0.0000 | 0.4784 | map00100 Steroid biosynthesis |
| **8.52_301.2168m/z** | M-H2O-H[-] | C04742 | 15(S)-HETE | 0.000323533 | 0.0000 | 0.0003 | 0.4781 | map00590 Arachidonic acid metabolism |
| **9.90_361.2365m/z** | M+HCOO[-] | C01953 | Pregnenolone | 0.001345000 | 0.0000 | 0.0001 | 0.4772 | map00140 Steroid hormone biosynthesis; map04925 Aldosterone synthesis and secretion |
| **11.15_388.7314m/z** | M-HCOONa+H[1+] | HMDB06460 | Arachidyl carnitine | 0.002476467 | 0.0018 | 0.0087 | 0.4749 |  |
| **0.71_146.1184m/z** | M[1+] | C01996 | Acetylcholine | 0.000300000 | 0.0036 | 0.0158 | 0.4736 | map00564 Glycerophospholipid metabolism; map04810 Regulation of actin cytoskeleton; map04976 Bile secretion |
| **11.61_810.5685m/z** | M(Cl37)-H[-] | C00040 | Acyl-CoA | 0.007576467 | 0.0000 | 0.0000 | 0.4624 | map00062 Fatty acid elongation; map00561 Glycerolipid metabolism; map00564 Glycerophospholipid metabolism; map04922 Glucagon signaling pathway; map04975 Fat digestion and absorption |
|  | M(Cl37)-H[-] | C00165 | DG(16:0/16:0/0:0) | 0.007576467 | 0.0000 | 0.0000 | 0.4624 | map04066 HIF-1 signaling pathway; map04071 Sphingolipid signaling pathway; map04072 Phospholipase D signaling pathway; map04915 Estrogen signaling pathway; map04918 Thyroid hormone synthesis; map04925 Aldosterone synthesis and secretion; map04975 Fat digestion and absorption |
| **9.38_123.1175m/z** | M-H4O2+H[1+] | C01601 | Pelargonic acid | 0.000723533 | 0.0000 | 0.0000 | 0.4614 | Lipid-Straight Fatty acid |
| **8.54_647.2027n** | M-H2O-H[-] | C02052 | Maltotetraose | 0.001323533 | 0.0000 | 0.0001 | 0.4551 |  |
| **9.04_422.3250m/z** | M+Na[1+] | C02990 | L-Palmitoylcarnitine | 0.000823533 | 0.0000 | 0.0000 | 0.4540 | map00071 Fatty acid degradation; map01212 Fatty acid metabolism |
| **8.28_414.3579m/z** | M-NH3+H[1+] | C02477 | Alpha-Tocopherol | 0.003976467 | 0.0003 | 0.0015 | 0.4374 | map00130 Ubiquinone and other terpenoid-quinone biosynthesis; map04977 Vitamin digestion and absorption |
| **9.39_281.0853m/z** | M-HCOOK+H[1+] | C00655 | Xanthylic acid | 0.002676467 | 0.0000 | 0.0000 | 0.4370 | map00230 Purine metabolism; map01065 Biosynthesis of alkaloids derived from histidine and purine |
| **8.48_760.2627m/z** | M+Cl37[-] | C02090 | Trypanothione | 0.007600000 | 0.0000 | 0.0002 | 0.4310 | map00480 Glutathione metabolism |
| **8.50_184.0745m/z** | M-H4O2+H[1+] | HMDB62199 | 3,5,12-trihydroxy-eicosa-8-trans-6,14-cis-trienoyl-CoA | 0.001576467 | 0.0000 | 0.0002 | 0.4267 |  |
| **8.62_140.5438m/z** | M+3H[3+] | HMDB12515 | 11'-Carboxy-alpha-chromanol | 0.000090200 | 0.0013 | 0.0064 | 0.4255 |  |
| **9.36_131.0861m/z** | M(S34)+H[1+] | C11950 | 3-Isopropylbut-3-enoic acid | 0.000676467 | 0.0000 | 0.0000 | 0.4213 | map00903 Limonene and pinene degradation |
| **10.16_407.2099m/z** | M+HCOONa[1+] | C04748 | Protein C-terminal S-farnesyl-L-cysteine methyl ester | 0.000176467 | 0.0001 | 0.0008 | 0.4203 | map00900 Terpenoid backbone biosynthesis |
| **7.78_423.3331n** | M-CO2+H[1+] | C18043 | Cholesterol sulfate | 0.003923533 | 0.0000 | 0.0001 | 0.4195 | map00140 Steroid hormone biosynthesis |
| **7.78_301.2166m/z** | M-H2O-H[-] | C04805 | 5-HETE | 0.000623533 | 0.0000 | 0.0001 | 0.4155 | map00590 Arachidonic acid metabolism |
|  | M-H2O-H[-] | C14749 | 19(S)-HETE | 0.000623533 | 0.0000 | 0.0001 | 0.4155 | map00590 Arachidonic acid metabolism |
|  | M-H2O-H[-] | C14768 | 5,6-Epoxy-8,11,14-eicosatrienoic acid | 0.000623533 | 0.0000 | 0.0001 | 0.4155 | map00590 Arachidonic acid metabolism |
|  | M-H2O-H[-] | C14769 | 8,9-Epoxyeicosatrienoic acid | 0.000623533 | 0.0000 | 0.0001 | 0.4155 | map00590 Arachidonic acid metabolism |
|  | M-H2O-H[-] | C14770 | 11,12-Epoxyeicosatrienoic acid | 0.000623533 | 0.0000 | 0.0001 | 0.4155 | map00590 Arachidonic acid metabolism |
|  | M-H2O-H[-] | C14771 | 14,15-Epoxy-5,8,11-eicosatrienoic acid | 0.000623533 | 0.0000 | 0.0001 | 0.4155 | map00590 Arachidonic acid metabolism |
|  | M-H2O-H[-] | C14776 | 8-HETE | 0.000623533 | 0.0000 | 0.0001 | 0.4155 | map00590 Arachidonic acid metabolism; map03320 PPAR signaling pathway |
|  | M-H2O-H[-] | C14778 | 16(R)-HETE | 0.000623533 | 0.0000 | 0.0001 | 0.4155 | map00590 Arachidonic acid metabolism |
|  | M-H2O-H[-] | C14779 | 9(S)-HETE | 0.000623533 | 0.0000 | 0.0001 | 0.4155 | map00590 Arachidonic acid metabolism |
| **11.23_768.5523m/z** | M+HCOONa[1+] | C02686 | Galactosylceramide | 0.007376467 | 0.0000 | 0.0000 | 0.4122 | map00600 Sphingolipid metabolism |
| **9.70_277.2171m/z** | M-H2O-H[-] | C14762 | 13S-hydroxyoctadecadienoic acid | 0.000123533 | 0.0000 | 0.0000 | 0.4119 | map00591 Linoleic acid metabolism; map03320 PPAR signaling pathway |
| **8.52_643.1956m/z** | M-H[-] | C05981 | Phosphatidylinositol-3,4,5-trisphosphate | 0.002076467 | 0.0003 | 0.0017 | 0.4086 | map04150 mTOR signaling pathway; map04151 PI3K-Akt signaling pathway; map00562 Inositol phosphate metabolism; map04918 Thyroid hormone synthesis; map04070 Phosphatidylinositol signaling system; map04071 Sphingolipid signaling pathway; map04960 Aldosterone-regulated sodium reabsorption |
| **10.16_350.2827n** | M(S34)+H[1+] | C11695 | Anandamide | 0.002776467 | 0.0001 | 0.0005 | 0.4057 | map04080 Neuroactive ligand-receptor interaction |
| **11.32_833.0414n** | M+HCOOK[1+] | C04230 | LysoPC(14:0) | 0.007423533 | 0.0018 | 0.0085 | 0.3898 | map00564 Glycerophospholipid metabolism |
| **8.48_606.3420m/z** | M-H[-] | G10610 | UDP-N-acetyl-D-glucosamine | 0.004423533 | 0.0000 | 0.0001 | 0.3873 | map00563 Glycosylphosphatidylinositol (GPI)-anchor biosynthesis |
| **8.48_692.2733m/z** | M(C13)-H[-] | C00369 | Starch | 0.003176467 | 0.0000 | 0.0001 | 0.3846 | map00500 Starch and sucrose metabolism; map04910 Insulin signaling pathway |
| **10.94_482.2966n** | M-H2O+H[1+] | C05465 | Taurochenodesoxycholic acid | 0.003123533 | 0.0000 | 0.0000 | 0.3820 | map00120 Primary bile acid biosynthesis; map04979 Cholesterol metabolism |
| **10.16_489.1628m/z** | M(C13)+2H[2+] | C05273 | (2E)-Tetradecenoyl-CoA | 0.004873533 | 0.0000 | 0.0001 | 0.3630 | map00062 Fatty acid elongation; map00071 Fatty acid degradation; map01212 Fatty acid metabolism |
| **11.73_429.3726m/z** | M+H2O+H[1+] | C15783 | 5-Dehydroavenasterol | 0.000176467 | 0.0000 | 0.0000 | 0.3616 | map00100 Steroid biosynthesis |
| **11.34_277.2173m/z** | M-H[-] | C06427 | Alpha-Linolenic acid | 0.000023533 | 0.0000 | 0.0000 | 0.3471 | map00592 alpha-Linolenic acid metabolism; map01040 Biosynthesis of unsaturated fatty acids |
|  | M-H[-] | C06426 | Gamma-Linolenic acid | 0.000023533 | 0.0000 | 0.0000 | 0.3471 | map00591 Linoleic acid metabolism; map01040 Biosynthesis of unsaturated fatty acids |
| **8.51_125.0006m/z** | M-CO2+H[1+] | C00074 | Phosphoenolpyruvic acid | 0.000723533 | 0.0000 | 0.0001 | 0.3470 | map00010 Glycolysis / Gluconeogenesis; map00400 Phenylalanine, tyrosine and tryptophan biosynthesis; map01062 Biosynthesis of terpenoids and steroids; map01063 Biosynthesis of alkaloids derived from shikimate pathway; map01230 Biosynthesis of amino acids; map02060 Phosphotransferase system (PTS) |
|  | M-HCOOH+H[1+] | C00111 | Dihydroxyacetone phosphate | 0.000723533 | 0.0000 | 0.0001 | 0.3470 | map00010 Glycolysis / Gluconeogenesis; map00040 Pentose and glucuronate interconversions; map00051 Fructose and mannose metabolism; map00052 Galactose metabolism; map00561 Glycerolipid metabolism; map00562 Inositol phosphate metabolism; map00564 Glycerophospholipid metabolism; map00760 Nicotinate and nicotinamide metabolism; map01230 Biosynthesis of amino acids |
|  | M-HCOOH+H[1+] | C00118 | D-Glyceraldehyde 3-phosphate | 0.000723533 | 0.0000 | 0.0001 | 0.3470 | map00010 Glycolysis / Gluconeogenesis; map00562 Inositol phosphate metabolism; map00750 Vitamin B6 metabolism; map00900 Terpenoid backbone biosynthesis; map01230 Biosynthesis of amino acids; Biosynthesis of alkaloids |
| **9.34_810.5690m/z** | M(Cl37)-H[-] | C00040 | Acyl-CoA | 0.008076467 | 0.0000 | 0.0000 | 0.3459 | map00062 Fatty acid elongation; map00561 Glycerolipid metabolism; map00564 Glycerophospholipid metabolism; map04922 Glucagon signaling pathway; map04975 Fat digestion and absorption |
|  | M(Cl37)-H[-] | C00165 | DG(16:0/16:0/0:0) | 0.008076467 | 0.0000 | 0.0000 | 0.3459 | map04066 HIF-1 signaling pathway; map04071 Sphingolipid signaling pathway; map04072 Phospholipase D signaling pathway; map04915 Estrogen signaling pathway; map04918 Thyroid hormone synthesis; map04925 Aldosterone synthesis and secretion; map04975 Fat digestion and absorption |
| **8.54_499.2707n** | M+NaCl[1+] | C00110 | dolichyl phosphate | 0.001176467 | 0.0000 | 0.0000 | 0.3412 | map00510 N-Glycan biosynthesis |
| **10.01_261.2218m/z** | M-C3H4O2+H[1+] | C05138 | 17a-Hydroxypregnenolone | 0.000523533 | 0.0000 | 0.0000 | 0.3357 | map00140 Steroid hormone biosynthesis |
|  | M-CO2+H[1+] | C05139 | 16a-Hydroxydehydroisoandrosterone | 0.000523533 | 0.0000 | 0.0000 | 0.3357 | map00140 Steroid hormone biosynthesis |
|  | M-CO2+H[1+] | C05294 | 19-Hydroxytestosterone | 0.000523533 | 0.0000 | 0.0000 | 0.3357 | map00140 Steroid hormone biosynthesis |
|  | M-CO2+H[1+] | C18045 | 7a-Hydroxydehydroepiandrosterone | 0.000523533 | 0.0000 | 0.0000 | 0.3357 | map00140 Steroid hormone biosynthesis |
|  | M-CO2+H[1+] | C18075 | 11beta,17beta-Dihydroxy-4-androsten-3-one | 0.000523533 | 0.0000 | 0.0000 | 0.3357 | map00140 Steroid hormone biosynthesis |
| **10.92_154.9735m/z** | M+Br81[-] | C00151 | L-Amino acid | 0.001500000 | 0.0000 | 0.0000 | 0.3305 | map00480 Glutathione metabolism |
| **12.24_636.5559m/z** | M+HCOONa[1+] | C12126 | Dihydroceramide | 0.002223533 | 0.0000 | 0.0001 | 0.3247 | map00600 Sphingolipid metabolism |
| **8.51_542.2511m/z** | M+Br81[-] | C01747 | Galactosylsphingosine | 0.000500000 | 0.0000 | 0.0000 | 0.3239 | map00600 Sphingolipid metabolism |
| **11.65_741.5593n** | M+Na[1+] | C00157 | Phosphatidylcholine | 0.004976467 | 0.0000 | 0.0000 | 0.3154 | map00564 Glycerophospholipid metabolism; map00590 Arachidonic acid metabolism; map00591 Linoleic acid metabolism; map00592 alpha-Linolenic acid metabolism |
| **10.89_359.1533m/z** | M+HCOONa[1+] | C02839 | L-Tyrosyl-tRNA(Tyr) | 0.002376467 | 0.0000 | 0.0000 | 0.3097 | map00970 Aminoacyl-tRNA biosynthesis |
| **8.51_335.2585m/z** | M+H2O+H[1+] | C01953 | Pregnenolone | 0.000423533 | 0.0000 | 0.0000 | 0.3079 | map00140 Steroid hormone biosynthesis |
| **8.83_520.2658m/z** | M(C13)-H[-] | C00621 | Dolichyl diphosphate | 0.002323533 | 0.0001 | 0.0004 | 0.2954 | map00510 N-Glycan biosynthesis; map00900 Terpenoid backbone biosynthesis |
| **8.65_335.2584m/z** | M+H2O+H[1+] | C01953 | Pregnenolone | 0.000323533 | 0.0000 | 0.0000 | 0.2926 | map00140 Steroid hormone biosynthesis; map04925 Aldosterone synthesis and secretion |
| **11.11_952.6029m/z** | M+NaCl[1+] | C14144 | 5-Carboxy-2-pentenoyl-CoA | 0.006976467 | 0.0000 | 0.0001 | 0.2855 | map00360 Phenylalanine metabolism; map00362 Benzoate degradation; map00930 Caprolactam degradation |
| **11.03_1132.4581m/z** | M-H+O[-] | C16529 | Lignoceroyl-CoA | 0.000366467 | 0.0001 | 0.0007 | 0.2841 | map01040 Biosynthesis of unsaturated fatty acids; map01212 Fatty acid metabolism |
| **7.75_345.2048m/z** | M+HCOO[-] | C00777 | Retinoic acid | 0.001745000 | 0.0000 | 0.0002 | 0.2801 | map00830 Retinol metabolism; map04672 Intestinal immune network for IgA production |
| **10.92_1162.4750m/z** | M+HCOO[-] | C16529 | Lignoceroyl-CoA | 0.007255000 | 0.0032 | 0.0144 | 0.2594 | map01040 Biosynthesis of unsaturated fatty acids; map01212 Fatty acid metabolism |
| **8.62_1055.1110m/z** | M-HCOOK+H[1+] | C05461 | Chenodeoxyglycocholoyl-CoA | 0.005023533 | 0.0037 | 0.0159 | 0.2455 |  |
| **9.67_311.2937m/z** | M-H[-] | C06425 | Arachidic acid | 0.001823533 | 0.0002 | 0.0013 | 0.2436 | map01040 Biosynthesis of unsaturated fatty acids |
| **9.37_391.1250m/z** | M-H+O[-] | C00255 | Riboflavin | 0.000933533 | 0.0000 | 0.0000 | 0.2359 | map00740 Riboflavin metabolism |
| **10.85_365.3053m/z** | M-CO+H[1+] | C02528 | Chenodeoxycholic acid | 0.000323533 | 0.0000 | 0.0000 | 0.2334 | map00120 Primary bile acid biosynthesis |
| **8.50_277.2166m/z** | M-H2O-H[-] | C14762 | 13S-hydroxyoctadecadienoic acid | 0.000623533 | 0.0000 | 0.0000 | 0.2284 | map00591 Linoleic acid metabolism; map03320 PPAR signaling pathway |
| **8.20_291.1963m/z** | M-H2O-H[-] | C04785 | 13(S)-HPOT | 0.000223533 | 0.0000 | 0.0000 | 0.2283 | map00592 alpha-Linolenic acid metabolism |
|  | M-H2O-H[-] | C16321 | 9(S)-HPOT | 0.000223533 | 0.0000 | 0.0000 | 0.2283 | map00592 alpha-Linolenic acid metabolism |
| **11.06_1162.4652m/z** | M+HCOO[-] | C16529 | Lignoceroyl-CoA | 0.002545000 | 0.0000 | 0.0001 | 0.2261 | map01040 Biosynthesis of unsaturated fatty acids; map01212 Fatty acid metabolism |
| **10.01_293.2459m/z** | M-CO2+H[1+] | C02165 | Leukotriene B4 | 0.001676467 | 0.0000 | 0.0000 | 0.2224 | map00590 Arachidonic acid metabolism; map03320 PPAR signaling pathway |
|  | M-CO2+H[1+] | C14808 | Hepoxilin A3 | 0.001676467 | 0.0000 | 0.0000 | 0.2224 | map00590 Arachidonic acid metabolism |
|  | M-CO2+H[1+] | C05356 | 5(S)-Hydroperoxyeicosatetraenoic acid | 0.001676467 | 0.0000 | 0.0000 | 0.2224 | map00590 Arachidonic acid metabolism |
|  | M-CO2+H[1+] | C05966 | 15(S)-HPETE | 0.001676467 | 0.0000 | 0.0000 | 0.2224 | map00590 Arachidonic acid metabolism |
|  | M-CO2+H[1+] | C14781 | 15H-11,12-EETA | 0.001676467 | 0.0000 | 0.0000 | 0.2224 | map00590 Arachidonic acid metabolism |
|  | M-CO2+H[1+] | C14813 | 11H-14,15-EETA | 0.001676467 | 0.0000 | 0.0000 | 0.2224 | map00590 Arachidonic acid metabolism |
| **10.92_184.0741m/z** | M[1+] | C00588 | Phosphorylcholine | 0.000200000 | 0.0000 | 0.0000 | 0.2197 | map00564 Glycerophospholipid metabolism |
| **11.06_433.7534m/z** | M-C3H4O2+H[1+] | HMDB06455 | Arachidonyl carnitine | 0.002423533 | 0.0000 | 0.0000 | 0.2168 |  |
| **8.51_475.2708n** | M-HCOOH+H[1+] | C00621 | Dolichyl diphosphate | 0.003076467 | 0.0000 | 0.0000 | 0.2024 | map00510 N-Glycan biosynthesis; map00900 Terpenoid backbone biosynthesis |
| **7.75_277.2167m/z** | M-C3H4O2+H[1+] | C05478 | 3,21-Dihydroxy-5-pregnane-11,20-dione | 0.000423533 | 0.0000 | 0.0000 | 0.1982 | map00140 Steroid hormone biosynthesis |
|  | M-C3H4O2+H[1+] | C05487 | 17,21-Dihydroxypreg-nenolone | 0.000423533 | 0.0000 | 0.0000 | 0.1982 | map00140 Steroid hormone biosynthesis |
| **11.08_412.7299m/z** | M-CO2+H[1+] | HMDB06460 | Arachidyl carnitine | 0.001576467 | 0.0000 | 0.0000 | 0.1914 |  |
| **9.04_353.2679m/z** | M+HCOONa[1+] | C01530 | Stearic acid | 0.001723533 | 0.0000 | 0.0000 | 0.1697 | map01040 Biosynthesis of unsaturated fatty acids |
| **7.75_466.3175m/z** | M+K-2H[-] | C02477 | Alpha-Tocopherol | 0.004547066 | 0.0000 | 0.0000 | 0.1608 | map00130 Ubiquinone and other terpenoid-quinone biosynthesis; map04977 Vitamin digestion and absorption |
| **10.89_846.5329m/z** | M+Cl37[-] | C00040 | Acyl-CoA | 0.004000000 | 0.0000 | 0.0000 | 0.1432 | map00062 Fatty acid elongation; map00561 Glycerolipid metabolism; map00564 Glycerophospholipid metabolism; map04922 Glucagon signaling pathway; map04975 Fat digestion and absorption |
|  | M+Cl37[-] | C00165 | DG(16:0/16:0/0:0) | 0.004000000 | 0.0000 | 0.0000 | 0.1432 | map04066 HIF-1 signaling pathway; map04071 Sphingolipid signaling pathway; map04072 Phospholipase D signaling pathway; map04915 Estrogen signaling pathway; map04918 Thyroid hormone synthesis; map04925 Aldosterone synthesis and secretion; map04975 Fat digestion and absorption |
| **9.04_451.1796m/z** | M(S34)-H[-] | C00353 | Geranylgeranyl pyrophosphate | 0.002523533 | 0.0002 | 0.0011 | 0.0971 | map00130 Ubiquinone and other terpenoid-quinone biosynthesis; map01062 Biosynthesis of terpenoids and steroids |
|  | M(S34)-H[-] | C11356 | Geranylneryl diphosphate | 0.002523533 | 0.0002 | 0.0011 | 0.0971 | map01062 Biosynthesis of terpenoids and steroids |
| **11.03_738.5100m/z** | M+Br81[-] | G00111 | GD1a | 0.001900000 | 0.0000 | 0.0000 | 0.0911 | map00604 Glycosphingolipid biosynthesis |
| **12.65_688.5904m/z** | M-H[-] | G00094 | Globoside | 0.002323533 | 0.0000 | 0.0000 | 0.0456 | map00603 Glycosphingolipid biosynthesis |
|  | M-H[-] | G00093 | Globotriaosylceramide | 0.002323533 | 0.0000 | 0.0000 | 0.0456 | map00603 Glycosphingolipid biosynthesis |

**Note:** The intensity of the most abundant metabolites in females, and the intensity of the metabolites were “normalized”. Putatively identified using KEGG and HMDB.

**
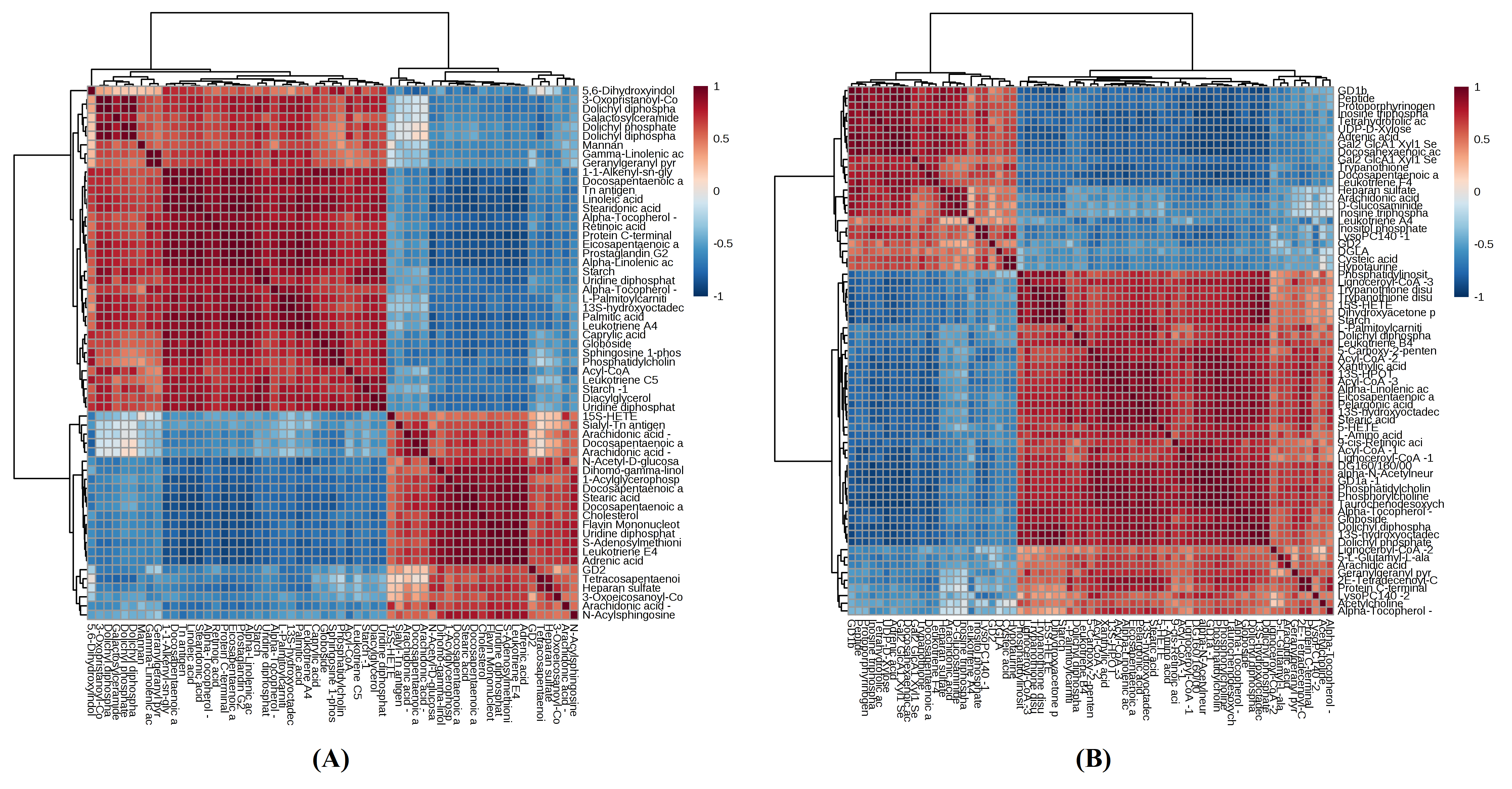
**

**Figure S2. The Pearson’s correlation analyses for the discriminating metabolites of lipids and carbohydrates metabolisms in muscle tissues of female (A) and male (B) *C. idellus*, respectively.**

**Note:** The differential signatures were annotated with their potential metabolite names after mapping with compound databases. The diversity of color referred to the pair-wise correlation coefficient ranging from 1 (red) to -1 (blue).


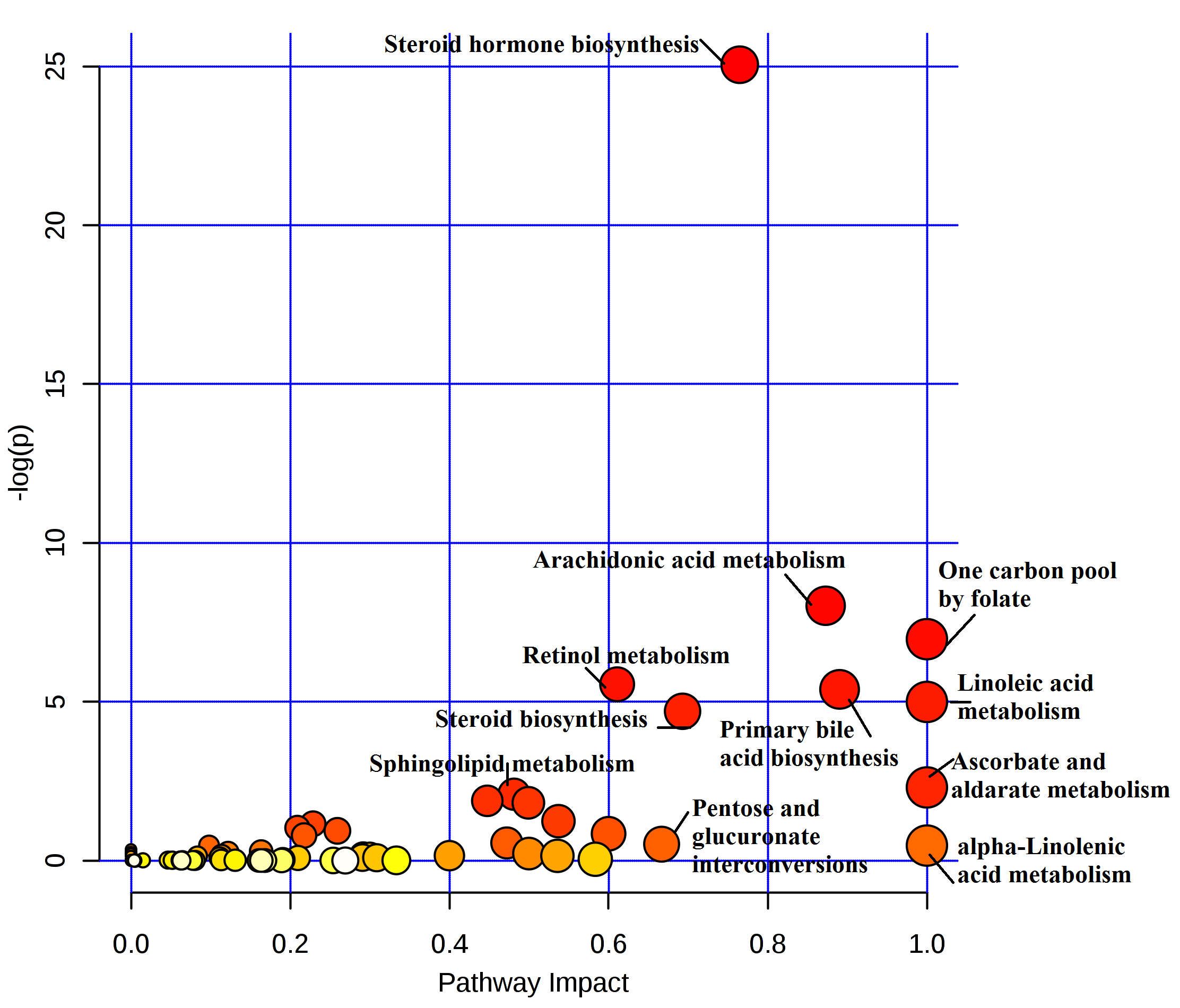


**(A)**

**
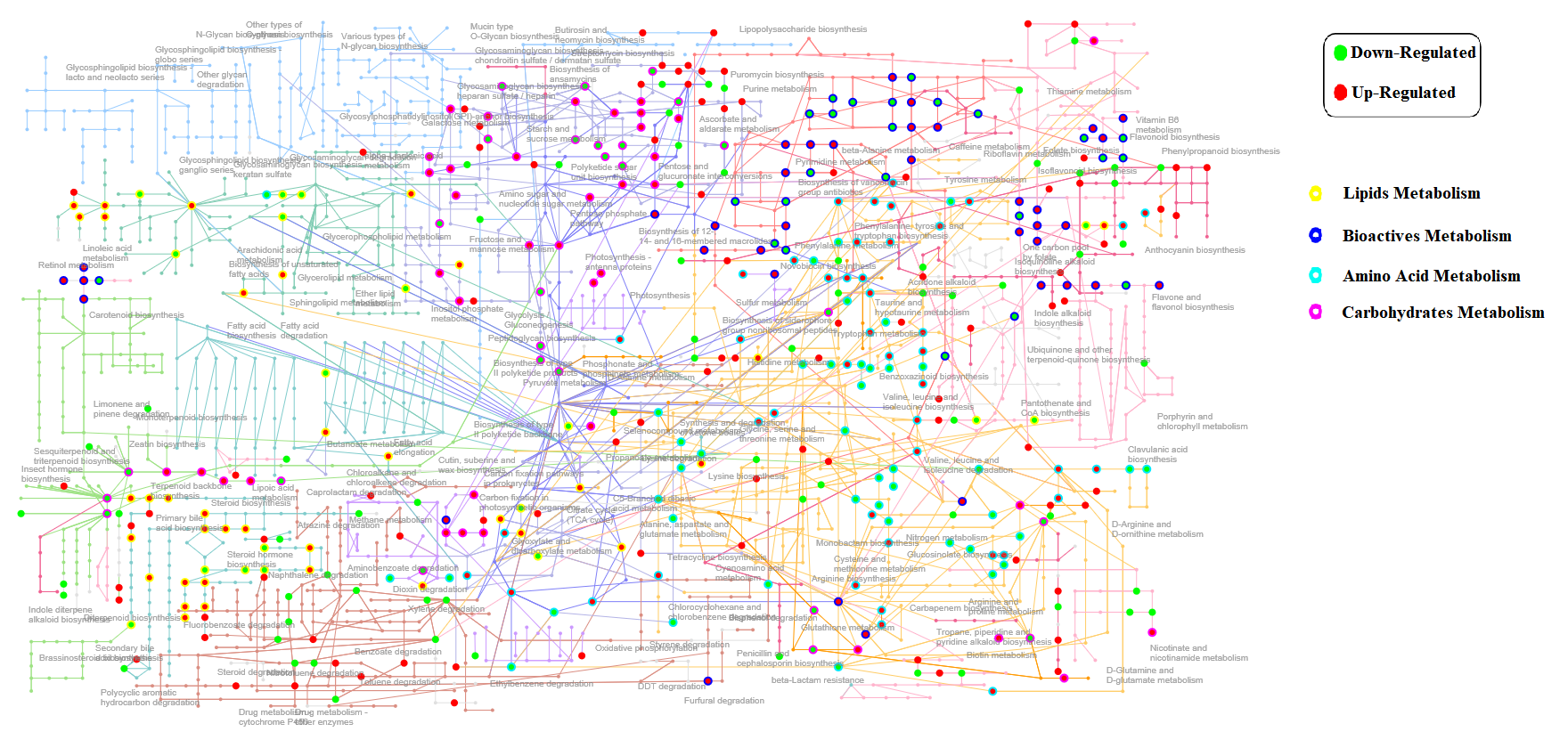
**

**(B)**

**Figure S3. The pathway enrichment and network analyses for the significant metabolites in male *C. idellus*. (A) The scatter plot was used to visualize the pathway impact and enrichment results for all matching significant metabolites in male *C. idellus*; (B) The KEGG global metabolic network visualization of all significant metabolites (*P* < 0.05) in the male *C. idellus* metabolic profile.**

**Note:** The colored points represent different metabolic pathways. The various color levels indicate different levels of significance of metabolic pathways from low (white) to high (red). The different sizes of each point were used to represent the number of metabolites participated in the metabolic pathway. The greater rich factor, the greater the degree of pathway enrichment. Moreover the corresponding pathway’s name of each point is labeled. In the metabolic network, all up-regulated metabolites (FC AF/GF > 2) in AF groups were colored with red, whereas the down regulated metabolites (FC < 0.5) were colored in green. In addition, the different color circles represent the various physiological functions that the discriminating metabolites belong to. Moreover, each enriched pathways is annotated with the corresponding name.

**
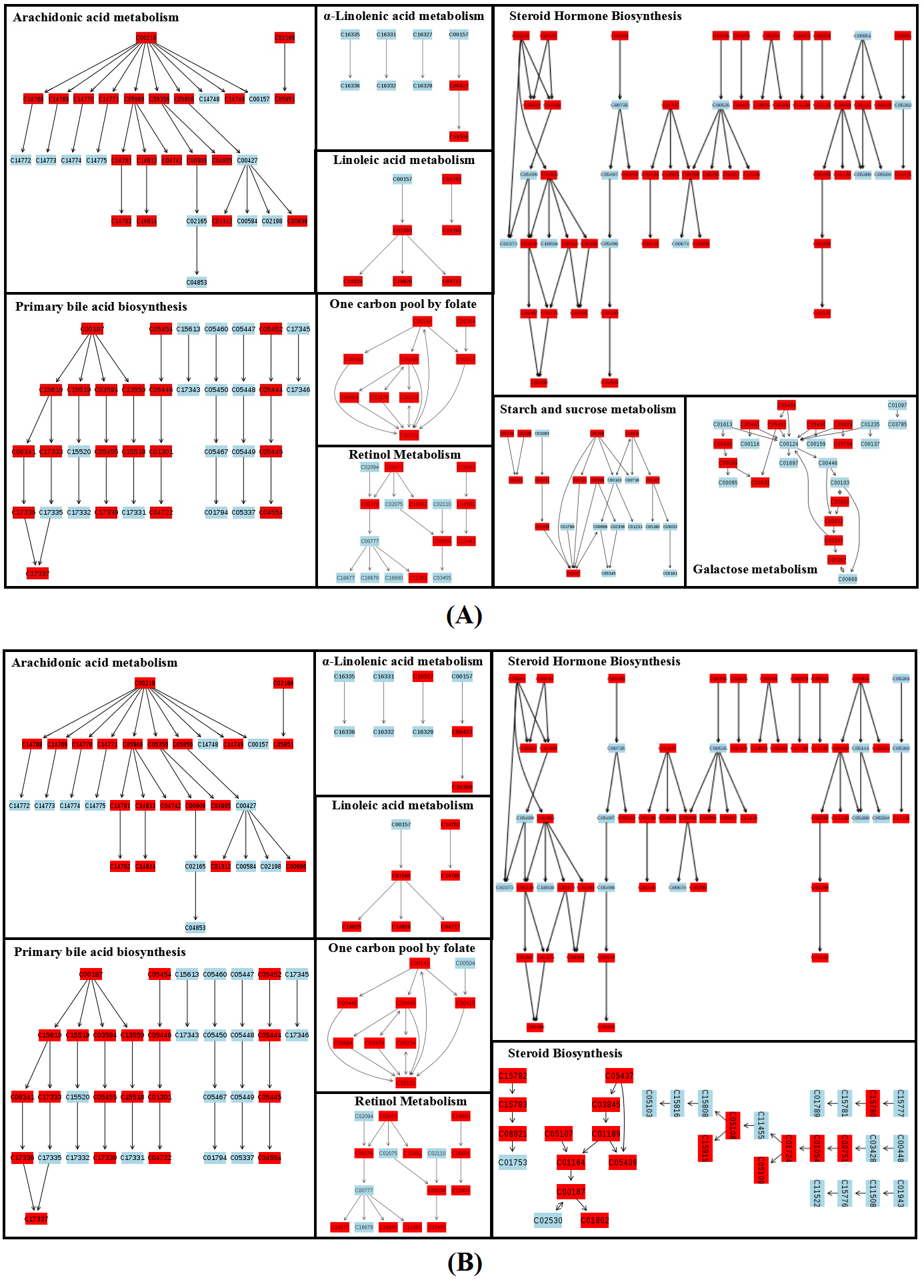
**

**Figure S4.** Visualization of overlapped significant metabolites onto corresponding pathways.

**Note:** **(A)** The overlapped metabolites in female *C. idellus* highlighted in significantly enriched pathways; **(B)** The pathway view for the altered metabolites between MAF and MGF. Light blue compounds in the figures mean that these metabolites were undetected in our data, but used as background for pathway enrichment analysis. Red colored compounds mean the metabolites were detected in our metabolomic data and involved in the specific metabolism pathway.
