# Supplementary material for: Metabolomics investigation of dietary effects on flesh quality in grass carp (Ctenopharyngodon idellus)
Source: Gigascience. 2018 Sep 6;7(10):giy111. doi: 10.1093/gigascience/giy111 (PMC6176498; doi:10.1093/gigascience/giy111)
Supplement: Supplemental Files [file giy111_supplemental_files.zip › Revised Supplementary Data.pdf]

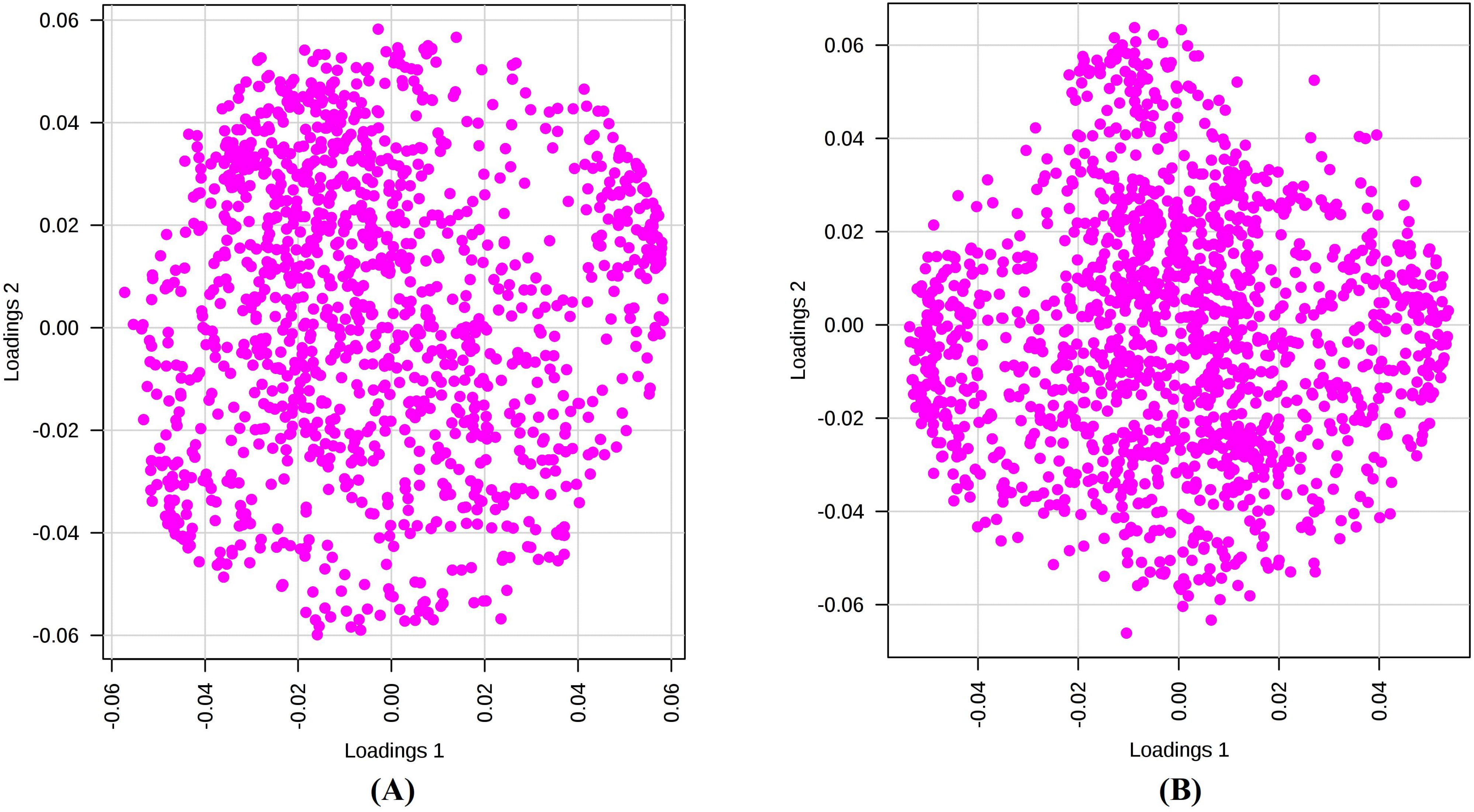

Figure S1. The PCA loading plots for the metabolomic data of muscle samples from female (A) and male (B) *C. idellus*.

Table S1. List of Discriminating Metabolites between Female-Artificial feed feeding group (FAF) and Female-Grass feeding group (FGF) *Ctenopharyngodon idellus*.

RT_Mass	Adducts	ID	Description	Mass.Diff	p.value	FDR	FC	Pathway
**9.37_280.2375m/z**	M[1+]	C01595	Linoleic acid	0.002700000	0.0000	0.0000	0.3137	map00591 Linoleic acid metabolism; map01040 Biosynthesis of unsaturated fatty acids
**9.37_278.2222n**	M(C13)+H[1+]	C16300	Stearidonic acid	0.002623533	0.0000	0.0000	0.4268	map00592 alpha-Linolenic acid metabolism
**8.93_369.2050m/z**	M(S34)-H[-]	C05956	Prostaglandin G2	0.003423533	0.0000	0.0000	0.4319	map00590 Arachidonic acid metabolism
	M(S34)-H[-]	C05962	6-Ketoprostaglandin E1	0.003423533	0.0000	0.0000	0.4319	map00590 Arachidonic acid metabolism
	M(S34)-H[-]	C05964	11-Dehydro-thromboxane B2	0.003423533	0.0000	0.0000	0.4319	map00590 Arachidonic acid metabolism
**9.37_243.2118m/z**	M-HCOOH+H[1+]	C00535	Testosterone	0.001023533	0.0000	0.0000	0.4794	map00140 Steroid hormone biosynthesis; map00984 Steroid degradation
	M-HCOOH+H[1+]	C01227	Dehydroepiandrosterone	0.001023533	0.0000	0.0000	0.4794	map00140 Steroid hormone biosynthesis; map00984 Steroid degradation
	M-HCOOH+H[1+]	C03772	Etiocholanedione	0.001023533	0.0000	0.0000	0.4794	map00140 Steroid hormone biosynthesis
**9.37_204.1522n**	M-H4O2+H[1+]	C04635	1-(1-Alkenyl)-sn-glycero-3-phosphoethanolamine	0.001123533	0.0000	0.0000	0.4157	map00565 Ether lipid metabolism
**8.94_417.1350m/z**	M+Br[-]	C04748	Protein C-terminal S-farnesyl-L-cysteine methyl ester	0.001300000	0.0000	0.0000	0.4069	map00900 Terpenoid backbone biosynthesis
**8.93_505.1806m/z**	M+Cl[-]	C04549	1-Phosphatidyl-1D-myo-inositol 3-phosphate	0.002700000	0.0000	0.0000	0.4827	map00562 Inositol phosphate metabolism; map04070 Phosphatidylinositol signaling system
**8.97_345.2049m/z**	M+CH3COO[-]	C00280	Androstenedione	0.001695000	0.0000	0.0000	0.3918	map00140 Steroid hormone biosynthesis; map00984 Steroid degradation
**9.37_227.0385m/z**	M+HCOONa[1+]	C02350	(S)(+)-Allantoin	0.000176467	0.0000	0.0000	0.4969	map00230 Purine metabolism
**9.37_123.1180m/z**	M-CO+H[1+]	C02576	Perillyl aldehyde	0.001223533	0.0000	0.0000	0.3947	map00903 Limonene and pinene degradation
**9.37_333.1511m/z**	M-H4O2+H[1+]	C04555	Dehydroepiandrosterone sulfate	0.000676467	0.0000	0.0000	0.4355	map00140 Steroid hormone biosynthesis
**8.93_347.2213m/z**	M+HCOO[-]	C06428	Eicosapentaenoic acid	0.000945000	0.0000	0.0000	0.4217	map01040 Biosynthesis of unsaturated fatty acids
**8.97_277.2173m/z**	M-H[-]	C06427	Alpha-Linolenic acid	0.000023533	0.0000	0.0000	0.3403	map00592 alpha-Linolenic acid metabolism; map01040 Biosynthesis of unsaturated fatty acids
**10.16_350.2826n**	M(S34)+H[1+]	C11695	Anandamide	0.002876467	0.0000	0.0000	0.2813	map04080 Neuroactive ligand-receptor interaction
**9.37_281.0868m/z**	M-HCOOK+H[1+]	C00655	Xanthylic acid	0.001176467	0.0000	0.0000	0.4893	map00230 Purine metabolism; map01065 Biosynthesis of alkaloids derived from histidine and purine
**8.94_301.2175m/z**	M-H+O[-]	C00473	Vitamin A	0.000166467	0.0000	0.0000	0.4077	map00830 Retinol metabolism
**9.54_349.2740m/z**	M+H2O+H[1+]	C16513	Docosapentaenoic acid	0.000223533	0.0000	0.0000	0.4267	map01040 Biosynthesis of unsaturated fatty acids
**9.69_323.2571m/z**	M-CO+H[1+]	C05476	Tetrahydrocorticosterone	0.000876467	0.0000	0.0000	0.3846	map00140 Steroid hormone biosynthesis
**9.36_443.2060m/z**	M-H4O2+H[1+]	C11131	2-Methoxy-estradiol-17b	0.000376467	0.0000	0.0000	0.4545	map00140 Steroid hormone biosynthesis

			3-glucuronide					
**8.93_257.2272m/z**	M(S34)-H[-]	C00249	Palmitic acid	0.001523533	0.0000	0.0000	0.4297	map00062 Fatty acid elongation; map00071 Fatty acid degradation; map01040 Biosynthesis of unsaturated fatty acids; map01212 Fatty acid metabolism
**9.97_445.2516m/z**	M+Cl37[-]	C00695	Cholic acid	0.001900000	0.0000	0.0000	2.0955	map00120 Primary bile acid biosynthesis
**9.69_449.1999m/z**	M(Cl37)-H[-]	C05503	17-beta-Estradiol-3-glucuronide	0.000276467	0.0000	0.0000	0.4155	map00140 Steroid hormone biosynthesis
**8.85_683.3352m/z**	M+ACN-H[-]	C11304	S-(PGA1)-glutathione	0.002031467	0.0000	0.0000	2.0319	
**9.97_439.2356m/z**	M(C13)-H[-]	C05952	Leukotriene E4	0.000276467	0.0000	0.0000	2.5133	map00590 Arachidonic acid metabolism
**9.55_400.1492m/z**	M+HCOO[-]	C01137	S-Adenosylmethioninamine	0.003645000	0.0000	0.0000	2.4926	map00270 Cysteine and methionine metabolism; map00330 Arginine and proline metabolism
**8.99_549.1676m/z**	M(S34)-H[-]	C04637	Phosphatidylinositol-4,5-bisphosphate	0.000676467	0.0000	0.0000	0.4606	map00562 Inositol phosphate metabolism; map04070 Phosphatidylinositol signaling system; map04072 Phospholipase D signaling pathway; map04151 PI3K-Akt signaling pathway; map04725 Cholinergic synapse; map04810 Regulation of actin cytoskeleton
**9.39_361.2719m/z**	M-H2O+H[1+]	C13856	2-Arachidonylglycerol	0.001776467	0.0000	0.0000	0.4186	map04080 Neuroactive ligand-receptor interaction
**8.34_714.2561m/z**	M+ACN-H[-]	C04886	(N-acetylneuraminosyl(a2-6)lactosamine)	0.001368533	0.0000	0.0000	0.3358	
**9.55_496.1222m/z**	M+ACN-H[-]	C00061	Flavin Mononucleotide	0.001668533	0.0000	0.0000	2.1552	map00190 Oxidative phosphorylation; map00740 Riboflavin metabolism
**9.36_533.4644n**	M+K[1+]	G00035	Sialyl-Tn antigen	0.001623533	0.0000	0.0000	0.4453	map00512 Mucin type O-glycan biosynthesis
**9.97_507.2250m/z**	M+CH3COO[-]	C05503	17-beta-Estradiol-3-glucuronide	0.002005000	0.0000	0.0000	2.7953	map00140 Steroid hormone biosynthesis
**9.55_396.1967m/z**	M(C13)-H[-]	C18044	3beta-Hydroxypregn-5-en-20-one sulfate	0.003576467	0.0000	0.0000	2.0027	map00140 Steroid hormone biosynthesis
**9.41_285.2587m/z**	M(S34)-H[-]	C01530	Stearic acid	0.001323533	0.0000	0.0000	2.2325	map01040 Biosynthesis of unsaturated fatty acids
**9.96_377.2680m/z**	M+HCOO[-]	C16527	Adrenic acid	0.001145000	0.0000	0.0000	2.7913	map01040 Biosynthesis of unsaturated fatty acids
**9.41_311.2379m/z**	M-H2O-H[-]	C16513	Docosapentaenoic acid	0.000123533	0.0000	0.0000	2.5819	map01040 Biosynthesis of unsaturated fatty acids
**11.69_688.5872m/z**	M-H[-]	G00094	Globoside	0.005523533	0.0000	0.0000	0.0637	map00603 Glycosphingolipid biosynthesis
**9.99_365.3030m/z**	M-CO+H[1+]	C02528	Chenodeoxycholic acid	0.001976467	0.0000	0.0000	2.8076	map00120 Primary bile acid biosynthesis
**9.36_147.1180m/z**	M(S34)+H[1+]	C06423	Caprylic acid	0.000076467	0.0000	0.0000	0.4958	map00061 Fatty acid biosynthesis; map01066 Biosynthesis of alkaloids derived from terpenoid and polyketide
**8.59_588.5224m/z**	M(S34)-H[-]	C04500	N-Acetyl-D-glucosaminyldiphosphodolichol	0.000623533	0.0000	0.0000	5.6192	Lipids
**8.71_299.2015m/z**	M-H2O-H[-]	C00909	Leukotriene A4	0.000123533	0.0000	0.0000	0.4558	map00590 Arachidonic acid metabolism
	M-H2O-H[-]	C14732	5-KETE	0.000123533	0.0000	0.0000	0.4558	map00590 Arachidonic acid metabolism

**11.72_430.3817m/z**	M[1+]	C02477	Alpha-Tocopherol	0.000600000	0.0000	0.0000	0.2414	map00130 Ubiquinone and other terpenoid-quinone biosynthesis; map04977 Vitamin digestion and absorption
**9.96_496.2127m/z**	M+Na-2H[-]	C11061	Retinoyl b-glucuronide	0.004252934	0.0000	0.0000	2.7578	map00830 Retinol metabolism
**10.37_467.2677m/z**	M+Br81[-]	C00187	Cholesterol	0.003500000	0.0000	0.0000	5.1926	map00120 Primary bile acid biosynthesis; map00140 Steroid hormone biosynthesis; map01066 Biosynthesis of alkaloids derived from terpenoid and polyketide; map04975 Fat digestion and absorption; map04977 Vitamin digestion and absorption; map04979 Cholesterol metabolism
**9.97_247.2428m/z**	M-CO2+H[1+]	C00167	Uridine diphosphate glucuronic acid	0.000723533	0.0000	0.0000	2.1033	map00040 Pentose and glucuronate interconversions; map00520 Amino sugar and nucleotide sugar metabolism
	M-CO2+H[1+]	C00523	Androsterone	0.000723533	0.0000	0.0000	2.1033	map00140 Steroid hormone biosynthesis
	M-CO2+H[1+]	C03917	Dihydrotestosterone	0.000723533	0.0000	0.0000	2.1033	map00140 Steroid hormone biosynthesis
	M-CO2+H[1+]	C04295	5-Androstenediol	0.000723533	0.0000	0.0000	2.1033	map00140 Steroid hormone biosynthesis
	M-CO2+H[1+]	C04373	Etiocholanolone	0.000723533	0.0000	0.0000	2.1033	map00140 Steroid hormone biosynthesis
**9.86_295.2427m/z**	M-H4O2+H[1+]	C16513	Docosapentaenoic acid	0.000723533	0.0000	0.0000	2.1815	map01040 Biosynthesis of unsaturated fatty acids
**9.81_375.1817m/z**	M-C3H4O2+H[1+]	C11133	Estrone glucuronide	0.001423533	0.0000	0.0000	0.4882	map00140 Steroid hormone biosynthesis
**7.80_422.3272m/z**	M+Na[1+]	C02990	L-Palmitoylcarnitine	0.003023533	0.0000	0.0000	0.2499	map00071 Fatty acid degradation; map01212 Fatty acid metabolism
**8.35_692.2700m/z**	M(C13)-H[-]	C00369	Starch	0.000123533	0.0000	0.0000	0.4039	map00500 Starch and sucrose metabolism
**8.93_943.6062m/z**	M+Br81[-]	C11378	Coenzyme Q10	0.006000000	0.0000	0.0000	0.0433	map04216 Ferroptosis
**8.94_283.2061m/z**	M-H[-]	C00376	Retinal	0.000623533	0.0000	0.0000	0.4549	map00830 Retinol metabolism
**8.99_335.1760m/z**	M+Cl[-]	C00777	Retinoic acid	0.001800000	0.0000	0.0000	0.3513	map00830 Retinol metabolism
**9.04_352.2613n**	M-CO+H[1+]	C06124	Sphingosine 1-phosphate	0.000223533	0.0000	0.0000	0.4019	map00600 Sphingolipid metabolism
**9.55_440.1357m/z**	M+Br[-]	C03819	1-Acylglycerophosphoinositol	0.000400000	0.0000	0.0000	2.5012	map00564 Glycerophospholipid metabolism
**7.36_277.2171m/z**	M-H2O-H[-]	C14762	13S-hydroxyoctadecadienoic acid	0.000123533	0.0000	0.0000	0.2522	map00591 Linoleic acid metabolism; map03320 PPAR signaling pathway
**8.48_240.1001m/z**	M-HCOOK+H[1+]	C00055	Cytidine monophosphate	0.002223533	0.0000	0.0001	0.4610	map00240 Pyrimidine metabolism
	M-HCOOK+H[1+]	C00165	Diacylglycerol	0.002223533	0.0000	0.0001	0.4610	map04066 HIF-1 signaling pathway; map04918 Thyroid hormone synthesis; map04923 Regulation of lipolysis in adipocytes; map04975 Fat digestion and absorption
	M-HCOOK+H[1+]	C01290	Lactosylceramide	0.002223533	0.0000	0.0001	0.4610	map00600 Sphingolipid metabolism
	M-HCOOK+H[1+]	C04776	T antigen	0.002223533	0.0000	0.0001	0.4610	map00512 Mucin type O-glycan biosynthesis
**8.62_549.2955m/z**	M-H2O+H[1+]	C00052	Uridine diphosphategalactose	0.002976467	0.0000	0.0001	0.4709	map00052 Galactose metabolism; map00520 Amino sugar and nucleotide sugar metabolism

**8.34_414.3582m/z**	M-NH3+H[1+]	C02477	Alpha-Tocopherol	0.003676467	0.0000	0.0001	0.3857	map00130 Ubiquinone and other terpenoid-quinone biosynthesis; map04977 Vitamin digestion and absorption
**9.04_313.0948m/z**	M+H[1+]	C00157	Phosphatidylcholine	0.002523533	0.0000	0.0001	0.2284	map00564 Glycerophospholipid metabolism; map00590 Arachidonic acid metabolism; map00591 Linoleic acid metabolism; map00592 alpha-Linolenic acid metabolism
**8.50_335.2587m/z**	M+H2O+H[1+]	C01953	Pregnenolone	0.000623533	0.0000	0.0001	0.3647	map00140 Steroid hormone biosynthesis; map04925 Aldosterone synthesis and secretion
**8.69_496.1751m/z**	M+Na-2H[-]	C11132	2-Methoxyestrone 3-glucuronide	0.003052934	0.0000	0.0002	0.4404	map00140 Steroid hormone biosynthesis
**9.92_271.2429m/z**	M-H4O2+H[1+]	C03242	8,11,14-Eicosatrienoic acid	0.000923533	0.0000	0.0002	2.1821	map00591 Linoleic acid metabolism; map01040 Biosynthesis of unsaturated fatty acids
**8.22_319.2279m/z**	M-H+O[-]	C00219	Arachidonic acid	0.000066467	0.0000	0.0002	3.5723	map00590 Arachidonic acid metabolism; map00591 Linoleic acid metabolism; map01040 Biosynthesis of unsaturated fatty acids
**8.35_647.2144m/z**	M+Br81[-]	C00029	Uridine diphosphate glucose	0.003700000	0.0000	0.0002	0.4508	map00040 Pentose and glucuronate interconversions; map00052 Galactose metabolism; map00500 Starch and sucrose metabolism; map00520 Amino sugar and nucleotide sugar metabolism; map00561 Glycerolipid metabolism
**8.35_712.2450m/z**	M+Na-2H[-]	C00369	Starch	0.003552934	0.0000	0.0002	0.4416	map00500 Starch and sucrose metabolism
**8.63_477.2161n**	M+H[1+]	C11132	2-Methoxyestrone 3-glucuronide	0.004223533	0.0000	0.0002	0.3273	map00140 Steroid hormone biosynthesis
**11.06_810.5628m/z**	M(Cl37)-H[-]	C00040	Acyl-CoA	0.001876467	0.0000	0.0002	0.4411	map00062 Fatty acid elongation; map00561 Glycerolipid metabolism; map00564 Glycerophospholipid metabolism; map04922 Glucagon signaling pathway; map04975 Fat digestion and absorption
**8.44_345.2439m/z**	M+CH3COO[-]	C00899	11-cis-Retinol	0.000905000	0.0000	0.0002	3.2567	map00830 Retinol metabolism
	M+CH3COO[-]	C16682	9-cis-Retinol	0.000905000	0.0000	0.0002	3.2567	map00830 Retinol metabolism
**9.37_269.6435m/z**	M+H+Na[2+]	C15516	Taurohyocholate	0.000626467	0.0000	0.0003	3.4145	
**8.50_532.1983m/z**	M(C13)+2H[2+]	C07297	3-Oxopristanoyl-CoA	0.003773533	0.0000	0.0004	0.2475	
**8.50_475.2707n**	M-HCOOH+H[1+]	C00621	Dolichyl diphosphate	0.003176467	0.0000	0.0004	0.2804	map00510 N-Glycan biosynthesis; map00900 Terpenoid backbone biosynthesis
**8.25_426.3586m/z**	M+H+Na[2+]	G00343	Maltopentaose	0.001173533	0.0001	0.0005	2.2398	
**8.62_556.7798m/z**	M-HCOONa+H[1+]	HMDB12993	Leukotriene C5	0.001923533	0.0001	0.0005	0.2669	
**9.26_393.2645m/z**	M-H+O[-]	C13856	2-Arachidonylglycerol	0.000133533	0.0001	0.0005	3.3809	map04080 Neuroactive ligand-receptor interaction
**8.63_335.2584m/z**	M+H2O+H[1+]	C01953	Pregnenolone	0.000323533	0.0001	0.0006	0.3822	map00140 Steroid hormone biosynthesis

**10.42_434.3409m/z**	M[1+]	C01301	3a,7a,12a-Trihydroxy-5b-cholestan-26-al	0.001300000	0.0001	0.0009	3.2825	map00120 Primary bile acid biosynthesis
**8.56_347.2596m/z**	M+CH3COO[-]	C15492	All-trans-13,14-dihydroretinol	0.001005000	0.0001	0.0011	3.1542	map00830 Retinol metabolism
**9.99_261.2216m/z**	M-C3H4O2+H[1+]	C05138	17a-Hydroxypregnenolone	0.000323533	0.0002	0.0011	0.1884	map00140 Steroid hormone biosynthesis
**9.20_403.2098m/z**	M+CH3COO[-]	C05490	11-Dehydrocorticosterone	0.002295000	0.0002	0.0015	2.0519	map00140 Steroid hormone biosynthesis
**9.53_461.2211m/z**	M-H2O+H[1+]	C11131	2-Methoxy-estradiol-17b 3-glucuronide	0.004123533	0.0003	0.0020	2.3475	map00140 Steroid hormone biosynthesis
**9.64_660.5101m/z**	M+Na[1+]	C00925	Heparan sulfate	0.001176467	0.0003	0.0022	2.1154	map00531 Glycosaminoglycan degradation
**10.90_780.4798m/z**	M+Br81[-]	C02686	Galactosylceramide	0.001500000	0.0005	0.0034	0.2193	map00600 Sphingolipid metabolism
**11.06_303.2326m/z**	M-H[-]	C00219	Arachidonic acid	0.000123533	0.0006	0.0039	2.4364	map00590 Arachidonic acid metabolism; map00591 Linoleic acid metabolism; map01040 Biosynthesis of unsaturated fatty acids
**11.37_637.5644m/z**	M(C13)+2H[2+]	C06140	GT1b	0.005423533	0.0009	0.0052	2.7712	map00604 Glycosphingolipid biosynthesis
**9.57_635.5003m/z**	M(Cl37)-H[-]	C00195	N-Acylsphingosine	0.005376467	0.0009	0.0055	2.7227	map00600 Sphingolipid metabolism
	M(Cl37)-H[-]	C02960	Ceramide 1-phosphate	0.005376467	0.0009	0.0055	2.7227	map00600 Sphingolipid metabolism
**8.97_323.2226m/z**	M+HCOO[-]	C06426	Gamma-Linolenic acid	0.000355000	0.0010	0.0060	0.2608	map00591 Linoleic acid metabolism; map01040 Biosynthesis of unsaturated fatty acids
**9.04_208.1113m/z**	M+NaCl[1+]	C05578	5,6-Dihydroxyindole	0.001276467	0.0012	0.0066	0.4928	map00350 Tyrosine metabolism
**8.63_499.2714n**	M+NaCl[1+]	C00110	dolichyl phosphate	0.000476467	0.0015	0.0085	0.4522	map00510 N-Glycan biosynthesis
**8.63_475.2712n**	M-HCOOH+H[1+]	C00621	Dolichyl diphosphate	0.002676467	0.0016	0.0089	0.3068	map00510 N-Glycan biosynthesis; map00900 Terpenoid backbone biosynthesis
**8.86_329.2486m/z**	M-H[-]	C16513	Docosapentaenoic acid	0.000023533	0.0017	0.0095	2.1767	map01040 Biosynthesis of unsaturated fatty acids
**9.54_427.2832m/z**	M+HCOONa[1+]	HMDB06322	Tetracosapentaenoic acid	0.001523533	0.0018	0.0096	2.5131	
**8.69_451.1790m/z**	M(S34)-H[-]	C11356	Geranylneryl diphosphate	0.003123533	0.0022	0.0115	0.2499	map01062 Biosynthesis of terpenoids and steroids
**6.37_334.2955m/z**	M+2H[2+]	C00464	Mannan	0.001276467	0.0028	0.0142	0.4904	map00051 Fructose and mannose metabolism
**8.37_319.2284m/z**	M-H[-]	C04742	15(S)-HETE	0.000576467	0.0034	0.0167	2.5865	map00590 Arachidonic acid metabolism
	M-H[-]	C04805	5-HETE	0.000576467	0.0034	0.0167	2.5865	map00590 Arachidonic acid metabolism
	M-H[-]	C14749	19(S)-HETE	0.000576467	0.0034	0.0167	2.5865	map00590 Arachidonic acid metabolism
	M-H[-]	C14768	5,6-Epoxy-8,11,14-eicosatrienoic acid	0.000576467	0.0034	0.0167	2.5865	map00590 Arachidonic acid metabolism
	M-H[-]	C14769	8,9-Epoxyeicosatrienoic acid	0.000576467	0.0034	0.0167	2.5865	map00590 Arachidonic acid metabolism
	M-H[-]	C14770	11,12-Epoxyeicosatrienoic acid	0.000576467	0.0034	0.0167	2.5865	map00590 Arachidonic acid metabolism
	M-H[-]	C14771	14,15-Epoxy-5,8,11-eicosatrien	0.000576467	0.0034	0.0167	2.5865	map00590 Arachidonic acid metabolism

			oic acid					
	M-H[-]	C14776	8-HETE	0.000576467	0.0034	0.0167	2.5865	map00590 Arachidonic acid metabolism; map03320 PPAR signaling pathway
	M-H[-]	C14778	16(R)-HETE	0.000576467	0.0034	0.0167	2.5865	map00590 Arachidonic acid metabolism
	M-H[-]	C14779	9(S)-HETE	0.000576467	0.0034	0.0167	2.5865	map00590 Arachidonic acid metabolism
**7.58_303.2327m/z**	M-H[-]	C00219	Arachidonic acid	0.000023533	0.0039	0.0194	2.7011	map00590 Arachidonic acid metabolism; map00591 Linoleic acid metabolism
**11.91_1160.3458m/z**	M+HCOOK[1+]	HMDB62369	3-Oxoeicosanoyl-CoA	0.009476467	0.0042	0.0205	3.1574	
**10.43_495.5046n**	M+H[1+]	G00035	Sialyl-Tn antigen	0.002676467	0.0085	0.0390	2.0541	map00512 Mucin type O-glycan biosynthesis

Table S2. List of Discriminating Muscle Metabolites between MAG and MGG *C. idellus*.

RT-Peaks	Matched.Form	ID	Description	Mass.Diff	p.value	FDR	FC	Pathway
**10.45_710.5450n**	M+K-2H[-]	C04886	(N-acetylneuraminosyl(a2-6)lactosamine)	0.004052934	0.0000	0.0000	6.8262	
**9.28_319.1888m/z**	M-H+O[-]	C05139	16a-Hydroxydehydroisoandrosterone	0.002633533	0.0000	0.0001	5.4296	map00140 Steroid hormone biosynthesis
	M-H+O[-]	C05294	19-Hydroxytestosterone	0.002633533	0.0000	0.0001	5.4296	map00140 Steroid hormone biosynthesis
	M-H+O[-]	C18045	7a-Hydroxydehydroepiandrosterone	0.002633533	0.0000	0.0001	5.4296	map00140 Steroid hormone biosynthesis
	M-H+O[-]	C18075	11beta,17beta-Dihydroxy-4-androsten-3-one	0.002633533	0.0000	0.0001	5.4296	map00140 Steroid hormone biosynthesis
**9.92_633.4865m/z**	M(S34)-H[-]	G00157	(Gal)2 (GlcA)1 (Xyl)1 (Ser)1	0.002023533	0.0000	0.0000	4.8402	map00534 Glycosaminoglycan biosynthesis
**8.83_345.2429m/z**	M+CH3COO[-]	C00473	Vitamin A	0.000095000	0.0017	0.0080	4.7645	map00830 Retinol metabolism
**9.23_522.5857m/z**	M+H+Na[2+]	C14119	Naphthyl-2-oxomethyl-succinyl-CoA	0.000873533	0.0000	0.0000	4.3115	map00626 Naphthalene degradation; map01220 Degradation of aromatic compounds
**8.96_347.2595m/z**	M+CH3COO[-]	C15492	All-trans-13,14-dihydroretinol	0.000905000	0.0019	0.0087	4.1931	map00830 Retinol metabolism
**8.59_387.2151m/z**	M-H[-]	C01177	Inositol phosphate	0.003376467	0.0002	0.0011	3.7549	map00562 Inositol phosphate metabolism; map04070 Phosphatidylinositol signaling system
	M-H[-]	C01194	1-Phosphatidyl-D-myo-inositol	0.003376467	0.0002	0.0011	3.7549	map00562 Inositol phosphate metabolism; map00563 Glycosylphosphatidylinositol (GPI)-anchor biosynthesis; map00564 Glycerophospholipid metabolism; map04070 Phosphatidylinositol signaling system
**12.17_437.7842m/z**	M-HCOONa+H[1+]	HMDB06455	Arachidonyl carnitine	0.000476467	0.0000	0.0000	3.7082	

**10.46_1070.9030m/z**	M+HCOONa[1+]	HMDB12473	(3S)-3-Hydroxy-cis,cis-palmito -7,10-dienoyl-CoA	0.002676467	0.0012	0.0060	3.7041	
**8.59_319.2274m/z**	M+Na-2H[-]	C04230	LysoPC(14:0)	0.000047066	0.0001	0.0004	3.4579	map00564 Glycerophospholipid metabolism
**9.29_252.2095n**	M+NaCl[1+]	HMDB03892	(S)-N-Methylsalsolinol	0.001323533	0.0000	0.0000	3.1798	
**9.35_275.2359m/z**	M-CO2+H[1+]	C00909	Leukotriene A4	0.001076467	0.0005	0.0029	2.9768	map00590 Arachidonic acid metabolism
**10.45_377.2668m/z**	M+HCOO[-]	C16527	Adrenic acid	0.002345000	0.0000	0.0000	2.9507	map01040 Biosynthesis of unsaturated fatty acids
**10.69_323.2954m/z**	M-H[-]	G00115	GD1b	0.002676467	0.0000	0.0000	2.7411	map00604 Glycosphingolipid biosynthesis
**9.14_533.2639m/z**	M-H4O2+H[1+]	C06462	Leukotriene F4	0.003976467	0.0000	0.0000	2.6614	map00590 Arachidonic acid metabolism
**9.23_478.5050m/z**	M+Cl37[-]	HMDB12607	18-Carboxy-dinor-LTE4	0.001100000	0.0000	0.0002	2.6097	
	M+Br81[-]	HMDB06317	trans-Hexadec-2-enoyl carnitine	0.003100000	0.0000	0.0002	2.6097	
**8.85_321.2435m/z**	M-H+O[-]	C03242	8,11,14-Eicosatrienoic acid	0.000033533	0.0073	0.0294	2.5842	map00591 Linoleic acid metabolism; map01040 Biosynthesis of unsaturated fatty acids
**10.45_649.2190m/z**	M+Br81[-]	C01079	Protoporphyrinogen IX	0.002300000	0.0000	0.0001	2.5169	map00860 Porphyrin and chlorophyll metabolism
**9.39_269.6435m/z**	M+H+Na[2+]	C15516	Taurohyocholate	0.000626467	0.0000	0.0001	2.4845	
**9.67_726.3020m/z**	M(S34)+H[1+]	C02090	Trypanothione	0.005476467	0.0000	0.0000	2.4809	map00480 Glutathione metabolism
**0.59_124.0062m/z**	M-H[-]	C00245	Taurine	0.001223533	0.0027	0.0123	2.3985	map00120 Primary bile acid biosynthesis; map00430 Taurine and hypotaurine metabolism
**11.39_637.5638m/z**	M(C13)+2H[2+]	G00114	GD2	0.004823533	0.0066	0.0269	2.3706	map00604 Glycosphingolipid biosynthesis
	M(C13)+2H[2+]	G00116	GT1b	0.004823533	0.0066	0.0269	2.3706	map00604 Glycosphingolipid biosynthesis
**9.63_613.4801m/z**	M-H2O-H[-]	G00157	(Gal)2 (GlcA)1 (Xyl)1 (Ser)1	0.002023533	0.0000	0.0001	2.3648	map00534 Glycosaminoglycan biosynthesis
**0.59_126.0226m/z**	M-CO2+H[1+]	C00506	Cysteic acid	0.000623533	0.0016	0.0078	2.3518	map00270 Cysteine and methionine metabolism; map00430 Taurine and hypotaurine metabolism
	M-CO+H[1+]	C00606	3-Sulfinoalanine	0.000723533	0.0016	0.0078	2.3518	map00270 Cysteine and methionine metabolism; map00430 Taurine and hypotaurine metabolism
**9.94_580.3987m/z**	M+Br[-]	C06023	D-Glucosaminide	0.002400000	0.0021	0.0098	2.3227	map00520 Amino sugar and nucleotide sugar metabolism
**9.92_373.2351m/z**	M+HCOO[-]	C06429	Docosahexaenoic acid	0.002745000	0.0000	0.0000	2.3056	map01040 Biosynthesis of unsaturated fatty acids
**9.16_517.0267m/z**	M-H2O-H[-]	C00190	UDP-D-Xylose	0.000076467	0.0000	0.0000	2.2752	map00520 Amino sugar and nucleotide sugar metabolism
**10.46_496.2127m/z**	M+Na-2H[-]	C11061	Retinoyl b-glucuronide	0.004252934	0.0000	0.0000	2.2711	map00830 Retinol metabolism
	M+Na-2H[-]	C11061	Retinoyl b-glucuronide	0.004252934	0.0000	0.0000	2.2711	map00830 Retinol metabolism
**10.45_407.2097m/z**	M+HCOO[-]	C00735	Hydrocortisone	0.002755000	0.0000	0.0000	2.2513	map00140 Steroid hormone biosynthesis; map04960 Aldosterone-regulated sodium reabsorption; map04976 Bile secretion
**9.38_526.5734m/z**	M+Br[-]	C00219	Arachidonic acid	0.004900000	0.0016	0.0079	2.2238	map00590 Arachidonic acid metabolism; map00591 Linoleic acid metabolism;

								map01040 Biosynthesis of unsaturated fatty acids
**10.45_507.2261m/z**	M+CH3COO[-]	C05503	17-beta-Estradiol-3-glucuronide	0.003105000	0.0000	0.0000	2.2067	map00140 Steroid hormone biosynthesis; map04976 Bile secretion
**10.14_637.5188m/z**	M(C13)-H[-]	C00925	Heparan sulfate	0.000676467	0.0013	0.0065	2.1803	map00531 Glycosaminoglycan degradation
**10.98_588.8992m/z**	M+Br81[-]	C00081	Inosine triphosphate	0.003100000	0.0000	0.0000	2.1422	map00230 Purine metabolism
**9.49_508.5955m/z**	M+HCOOK[1+]	HMDB06461	Linoelaidyl carnitine	0.002176467	0.0003	0.0018	2.0908	
	M+HCOOK[1+]	HMDB06469	Linoleyl carnitine	0.002176467	0.0003	0.0018	2.0908	
**9.93_402.1905m/z**	M-CO2+H[1+]	C00101	Tetrahydrofolic acid	0.002023533	0.0000	0.0000	2.0720	map00260 Glycine, serine and threonine metabolism; map00790 Folate biosynthesis
**9.86_285.2580m/z**	M+HCOO[-]	C00012	Peptide	0.001355000	0.0000	0.0001	2.0703	map00330 Arginine and proline metabolism
**10.45_439.2356m/z**	M(C13)-H[-]	C05952	Leukotriene E4	0.000276467	0.0000	0.0000	2.0568	map00590 Arachidonic acid metabolism
**9.16_330.2518m/z**	M(C13)-H[-]	C16513	Docosapentaenoic acid	0.000223533	0.0000	0.0000	2.0545	map01040 Biosynthesis of unsaturated fatty acids
**9.51_546.9469m/z**	M+K[1+]	C00081	Inosine triphosphate	0.004323533	0.0084	0.0332	2.0496	map00230 Purine metabolism
**9.16_526.2939m/z**	M(S34)-H[-]	C02737	PS(16:0/16:0)	0.001623533	0.0000	0.0000	2.0264	map00260 Glycine, serine and threonine metabolism; map00564 Glycerophospholipid metabolism
**9.17_523.1746m/z**	M(C13)+2H[2+]	C16327	OPC8-CoA	0.003573533	0.0000	0.0000	2.0004	map00592 alpha-Linolenic acid metabolism
	M+NaCl[1+]	C05504	16-Glucuronide-estriol	0.004123533	0.0000	0.0000	2.0004	map00140 Steroid hormone biosynthesis
**11.40_738.5099m/z**	M+Br81[-]	G00111	GD1a	0.002000000	0.0000	0.0000	0.4985	map00604 Glycosphingolipid biosynthesis
**8.65_261.2216m/z**	M-CO2+H[1+]	C05139	16a-Hydroxydehydroisoandrosterone	0.000323533	0.0000	0.0001	0.4971	map00140 Steroid hormone biosynthesis
	M-CO2+H[1+]	C05294	19-Hydroxytestosterone	0.000323533	0.0000	0.0001	0.4971	map00140 Steroid hormone biosynthesis
	M-CO2+H[1+]	C18045	7a-Hydroxydehydroepiandrosterone	0.000323533	0.0000	0.0001	0.4971	map00140 Steroid hormone biosynthesis
	M-CO2+H[1+]	C18075	11beta,17beta-Dihydroxy-4-androsten-3-one	0.000323533	0.0000	0.0001	0.4971	map00140 Steroid hormone biosynthesis
**9.35_283.2077m/z**	M-H2O-H[-]	C06428	Eicosapentaenoic acid	0.000976467	0.0000	0.0000	0.4963	map01040 Biosynthesis of unsaturated fatty acids
	M-H[-]	C00376	Retinal	0.000976467	0.0000	0.0000	0.4963	map00830 Retinol metabolism
**9.04_121.1015m/z**	M+H+Na[2+]	C03740	5-L-Glutamyl-L-alanine	0.000376467	0.0002	0.0010	0.4914	map00480 Glutathione metabolism
**10.88_848.5268m/z**	M+K[1+]	C00040	Acyl-CoA	0.006976467	0.0000	0.0001	0.4832	map00062 Fatty acid elongation; map00561 Glycerolipid metabolism; map00564 Glycerophospholipid metabolism; map04975 Fat digestion and absorption
	M+K[1+]	C00165	DG(16:0/16:0/0:0)	0.006976467	0.0000	0.0001	0.4832	map04066 HIF-1 signaling pathway; map04071 Sphingolipid signaling pathway; map04072 Phospholipase D signaling pathway; map04915 Estrogen signaling

								pathway; map04918 Thyroid hormone synthesis; map04925 Aldosterone synthesis and secretion; map04975 Fat digestion and absorption
**8.48_766.2810m/z**	M+HCOO[-]	C03170	Trypanothione disulfide	0.005345000	0.0000	0.0002	0.4824	map00480 Glutathione metabolism
**12.26_429.3735m/z**	M+H2O+H[1+]	C15783	5-Dehydroavenasterol	0.000723533	0.0000	0.0000	0.4784	map00100 Steroid biosynthesis
**8.52_301.2168m/z**	M-H2O-H[-]	C04742	15(S)-HETE	0.000323533	0.0000	0.0003	0.4781	map00590 Arachidonic acid metabolism
**9.90_361.2365m/z**	M+HCOO[-]	C01953	Pregnenolone	0.001345000	0.0000	0.0001	0.4772	map00140 Steroid hormone biosynthesis; map04925 Aldosterone synthesis and secretion
**11.15_388.7314m/z**	M-HCOONa+H[1+]	HMDB06460	Arachidyl carnitine	0.002476467	0.0018	0.0087	0.4749	
**0.71_146.1184m/z**	M[1+]	C01996	Acetylcholine	0.000300000	0.0036	0.0158	0.4736	map00564 Glycerophospholipid metabolism; map04810 Regulation of actin cytoskeleton; map04976 Bile secretion
**11.61_810.5685m/z**	M(Cl37)-H[-]	C00040	Acyl-CoA	0.007576467	0.0000	0.0000	0.4624	map00062 Fatty acid elongation; map00561 Glycerolipid metabolism; map00564 Glycerophospholipid metabolism; map04922 Glucagon signaling pathway; map04975 Fat digestion and absorption
	M(Cl37)-H[-]	C00165	DG(16:0/16:0/0:0)	0.007576467	0.0000	0.0000	0.4624	map04066 HIF-1 signaling pathway; map04071 Sphingolipid signaling pathway; map04072 Phospholipase D signaling pathway; map04915 Estrogen signaling pathway; map04918 Thyroid hormone synthesis; map04925 Aldosterone synthesis and secretion; map04975 Fat digestion and absorption
**9.38_123.1175m/z**	M-H4O2+H[1+]	C01601	Pelargonic acid	0.000723533	0.0000	0.0000	0.4614	Lipid-Straight Fatty acid
**8.54_647.2027n**	M-H2O-H[-]	C02052	Maltotetraose	0.001323533	0.0000	0.0001	0.4551	
**9.04_422.3250m/z**	M+Na[1+]	C02990	L-Palmitoylcarnitine	0.000823533	0.0000	0.0000	0.4540	map00071 Fatty acid degradation; map01212 Fatty acid metabolism
**8.28_414.3579m/z**	M-NH3+H[1+]	C02477	Alpha-Tocopherol	0.003976467	0.0003	0.0015	0.4374	map00130 Ubiquinone and other terpenoid-quinone biosynthesis; map04977 Vitamin digestion and absorption
**9.39_281.0853m/z**	M-HCOOK+H[1+]	C00655	Xanthylic acid	0.002676467	0.0000	0.0000	0.4370	map00230 Purine metabolism; map01065 Biosynthesis of alkaloids derived from histidine and purine
**8.48_760.2627m/z**	M+Cl37[-]	C02090	Trypanothione	0.007600000	0.0000	0.0002	0.4310	map00480 Glutathione metabolism
**8.50_184.0745m/z**	M-H4O2+H[1+]	HMDB62199	3,5,12-trihydroxy-eicosa-8-trans-6,14-cis-trienoyl-CoA	0.001576467	0.0000	0.0002	0.4267	
**8.62_140.5438m/z**	M+3H[3+]	HMDB12515	11'-Carboxy-alpha-chromanol	0.000090200	0.0013	0.0064	0.4255	
**9.36_131.0861m/z**	M(S34)+H[1+]	C11950	3-Isopropylbut-3-enoic acid	0.000676467	0.0000	0.0000	0.4213	map00903 Limonene and pinene degradation
**10.16_407.2099m/z**	M+HCOONa[1+]	C04748	Protein C-terminal S-farnesyl-L-cysteine methyl	0.000176467	0.0001	0.0008	0.4203	map00900 Terpenoid backbone biosynthesis

			ester					
**7.78_423.3331n**	M-CO2+H[1+]	C18043	Cholesterol sulfate	0.003923533	0.0000	0.0001	0.4195	map00140 Steroid hormone biosynthesis
**7.78_301.2166m/z**	M-H2O-H[-]	C04805	5-HETE	0.000623533	0.0000	0.0001	0.4155	map00590 Arachidonic acid metabolism
	M-H2O-H[-]	C14749	19(S)-HETE	0.000623533	0.0000	0.0001	0.4155	map00590 Arachidonic acid metabolism
	M-H2O-H[-]	C14768	5,6-Epoxy-8,11,14-eicosatrienoic acid	0.000623533	0.0000	0.0001	0.4155	map00590 Arachidonic acid metabolism
	M-H2O-H[-]	C14769	8,9-Epoxyeicosatrienoic acid	0.000623533	0.0000	0.0001	0.4155	map00590 Arachidonic acid metabolism
	M-H2O-H[-]	C14770	11,12-Epoxyeicosatrienoic acid	0.000623533	0.0000	0.0001	0.4155	map00590 Arachidonic acid metabolism
	M-H2O-H[-]	C14771	14,15-Epoxy-5,8,11-eicosatrienoic acid	0.000623533	0.0000	0.0001	0.4155	map00590 Arachidonic acid metabolism
	M-H2O-H[-]	C14776	8-HETE	0.000623533	0.0000	0.0001	0.4155	map00590 Arachidonic acid metabolism; map03320 PPAR signaling pathway
	M-H2O-H[-]	C14778	16(R)-HETE	0.000623533	0.0000	0.0001	0.4155	map00590 Arachidonic acid metabolism
	M-H2O-H[-]	C14779	9(S)-HETE	0.000623533	0.0000	0.0001	0.4155	map00590 Arachidonic acid metabolism
**11.23_768.5523m/z**	M+HCOONa[1+]	C02686	Galactosylceramide	0.007376467	0.0000	0.0000	0.4122	map00600 Sphingolipid metabolism
**9.70_277.2171m/z**	M-H2O-H[-]	C14762	13S-hydroxyoctadecadienoic acid	0.000123533	0.0000	0.0000	0.4119	map00591 Linoleic acid metabolism; map03320 PPAR signaling pathway
**8.52_643.1956m/z**	M-H[-]	C05981	Phosphatidylinositol-3,4,5-trisphosphate	0.002076467	0.0003	0.0017	0.4086	map04150 mTOR signaling pathway; map04151 PI3K-Akt signaling pathway; map00562 Inositol phosphate metabolism; map04918 Thyroid hormone synthesis; map04070 Phosphatidylinositol signaling system; map04071 Sphingolipid signaling pathway; map04960 Aldosterone-regulated sodium reabsorption
**10.16_350.2827n**	M(S34)+H[1+]	C11695	Anandamide	0.002776467	0.0001	0.0005	0.4057	map04080 Neuroactive ligand-receptor interaction
**11.32_833.0414n**	M+HCOOK[1+]	C04230	LysoPC(14:0)	0.007423533	0.0018	0.0085	0.3898	map00564 Glycerophospholipid metabolism
**8.48_606.3420m/z**	M-H[-]	G10610	UDP-N-acetyl-D-glucosamine	0.004423533	0.0000	0.0001	0.3873	map00563 Glycosylphosphatidylinositol (GPI)-anchor biosynthesis
**8.48_692.2733m/z**	M(C13)-H[-]	C00369	Starch	0.003176467	0.0000	0.0001	0.3846	map00500 Starch and sucrose metabolism; map04910 Insulin signaling pathway
**10.94_482.2966n**	M-H2O+H[1+]	C05465	Taurochenodesoxycholic acid	0.003123533	0.0000	0.0000	0.3820	map00120 Primary bile acid biosynthesis; map04979 Cholesterol metabolism
**10.16_489.1628m/z**	M(C13)+2H[2+]	C05273	(2E)-Tetradecenoyl-CoA	0.004873533	0.0000	0.0001	0.3630	map00062 Fatty acid elongation; map00071 Fatty acid degradation; map01212 Fatty acid metabolism
**11.73_429.3726m/z**	M+H2O+H[1+]	C15783	5-Dehydroavenasterol	0.000176467	0.0000	0.0000	0.3616	map00100 Steroid biosynthesis
**11.34_277.2173m/z**	M-H[-]	C06427	Alpha-Linolenic acid	0.000023533	0.0000	0.0000	0.3471	map00592 alpha-Linolenic acid metabolism; map01040 Biosynthesis of unsaturated fatty acids
	M-H[-]	C06426	Gamma-Linolenic acid	0.000023533	0.0000	0.0000	0.3471	map00591 Linoleic acid metabolism; map01040 Biosynthesis of unsaturated

								fatty acids
**8.51_125.0006m/z**	M-CO2+H[1+]	C00074	Phosphoenolpyruvic acid	0.000723533	0.0000	0.0001	0.3470	map00010 Glycolysis / Gluconeogenesis; map00400 Phenylalanine, tyrosine and tryptophan biosynthesis; map01062 Biosynthesis of terpenoids and steroids; map01063 Biosynthesis of alkaloids derived from shikimate pathway; map01230 Biosynthesis of amino acids; map02060 Phosphotransferase system (PTS)
	M-HCOOH+H[1+]	C00111	Dihydroxyacetone phosphate	0.000723533	0.0000	0.0001	0.3470	map00010 Glycolysis / Gluconeogenesis; map00040 Pentose and glucuronate interconversions; map00051 Fructose and mannose metabolism; map00052 Galactose metabolism; map00561 Glycerolipid metabolism; map00562 Inositol phosphate metabolism; map00564 Glycerophospholipid metabolism; map00760 Nicotinate and nicotinamide metabolism; map01230 Biosynthesis of amino acids
	M-HCOOH+H[1+]	C00118	D-Glyceraldehyde 3-phosphate	0.000723533	0.0000	0.0001	0.3470	map00010 Glycolysis / Gluconeogenesis; map00562 Inositol phosphate metabolism; map00750 Vitamin B6 metabolism; map00900 Terpenoid backbone biosynthesis; map01230 Biosynthesis of amino acids; Biosynthesis of alkaloids
**9.34_810.5690m/z**	M(Cl37)-H[-]	C00040	Acyl-CoA	0.008076467	0.0000	0.0000	0.3459	map00062 Fatty acid elongation; map00561 Glycerolipid metabolism; map00564 Glycerophospholipid metabolism; map04922 Glucagon signaling pathway; map04975 Fat digestion and absorption
	M(Cl37)-H[-]	C00165	DG(16:0/16:0/0:0)	0.008076467	0.0000	0.0000	0.3459	map04066 HIF-1 signaling pathway; map04071 Sphingolipid signaling pathway; map04072 Phospholipase D signaling pathway; map04915 Estrogen signaling pathway; map04918 Thyroid hormone synthesis; map04925 Aldosterone synthesis and secretion; map04975 Fat digestion and absorption
**8.54_499.2707n**	M+NaCl[1+]	C00110	dolichyl phosphate	0.001176467	0.0000	0.0000	0.3412	map00510 N-Glycan biosynthesis
**10.01_261.2218m/z**	M-C3H4O2+H[1+]	C05138	17a-Hydroxypregnenolone	0.000523533	0.0000	0.0000	0.3357	map00140 Steroid hormone biosynthesis
	M-CO2+H[1+]	C05139	16a-Hydroxydehydroisoandrosterone	0.000523533	0.0000	0.0000	0.3357	map00140 Steroid hormone biosynthesis
	M-CO2+H[1+]	C05294	19-Hydroxytestosterone	0.000523533	0.0000	0.0000	0.3357	map00140 Steroid hormone biosynthesis
	M-CO2+H[1+]	C18045	7a-Hydroxydehydroepiandrosterone	0.000523533	0.0000	0.0000	0.3357	map00140 Steroid hormone biosynthesis
	M-CO2+H[1+]	C18075	11beta,17beta-Dihydroxy-4-androsten-3-one	0.000523533	0.0000	0.0000	0.3357	map00140 Steroid hormone biosynthesis
**10.92_154.9735m/z**	M+Br81[-]	C00151	L-Amino acid	0.001500000	0.0000	0.0000	0.3305	map00480 Glutathione metabolism
**12.24_636.5559m/z**	M+HCOONa[1+]	C12126	Dihydroceramide	0.002223533	0.0000	0.0001	0.3247	map00600 Sphingolipid metabolism
**8.51_542.2511m/z**	M+Br81[-]	C01747	Galactosylsphingosine	0.000500000	0.0000	0.0000	0.3239	map00600 Sphingolipid metabolism
**11.65_741.5593n**	M+Na[1+]	C00157	Phosphatidylcholine	0.004976467	0.0000	0.0000	0.3154	map00564 Glycerophospholipid metabolism; map00590 Arachidonic acid

								metabolism; map00591 Linoleic acid metabolism; map00592 alpha-Linolenic acid metabolism
**10.89_359.1533m/z**	M+HCOONa[1+]	C02839	L-Tyrosyl-tRNA(Tyr)	0.002376467	0.0000	0.0000	0.3097	map00970 Aminoacyl-tRNA biosynthesis
**8.51_335.2585m/z**	M+H2O+H[1+]	C01953	Pregnenolone	0.000423533	0.0000	0.0000	0.3079	map00140 Steroid hormone biosynthesis
**8.83_520.2658m/z**	M(C13)-H[-]	C00621	Dolichyl diphosphate	0.002323533	0.0001	0.0004	0.2954	map00510 N-Glycan biosynthesis; map00900 Terpenoid backbone biosynthesis
**8.65_335.2584m/z**	M+H2O+H[1+]	C01953	Pregnenolone	0.000323533	0.0000	0.0000	0.2926	map00140 Steroid hormone biosynthesis; map04925 Aldosterone synthesis and secretion
**11.11_952.6029m/z**	M+NaCl[1+]	C14144	5-Carboxy-2-pentenoyl-CoA	0.006976467	0.0000	0.0001	0.2855	map00360 Phenylalanine metabolism; map00362 Benzoate degradation; map00930 Caprolactam degradation
**11.03_1132.4581m/z**	M-H+O[-]	C16529	Lignoceroyl-CoA	0.000366467	0.0001	0.0007	0.2841	map01040 Biosynthesis of unsaturated fatty acids; map01212 Fatty acid metabolism
**7.75_345.2048m/z**	M+HCOO[-]	C00777	Retinoic acid	0.001745000	0.0000	0.0002	0.2801	map00830 Retinol metabolism; map04672 Intestinal immune network for IgA production
**10.92_1162.4750m/z**	M+HCOO[-]	C16529	Lignoceroyl-CoA	0.007255000	0.0032	0.0144	0.2594	map01040 Biosynthesis of unsaturated fatty acids; map01212 Fatty acid metabolism
**8.62_1055.1110m/z**	M-HCOOK+H[1+]	C05461	Chenodeoxyglycocholoyl-CoA	0.005023533	0.0037	0.0159	0.2455	
**9.67_311.2937m/z**	M-H[-]	C06425	Arachidic acid	0.001823533	0.0002	0.0013	0.2436	map01040 Biosynthesis of unsaturated fatty acids
**9.37_391.1250m/z**	M-H+O[-]	C00255	Riboflavin	0.000933533	0.0000	0.0000	0.2359	map00740 Riboflavin metabolism
**10.85_365.3053m/z**	M-CO+H[1+]	C02528	Chenodeoxycholic acid	0.000323533	0.0000	0.0000	0.2334	map00120 Primary bile acid biosynthesis
**8.50_277.2166m/z**	M-H2O-H[-]	C14762	13S-hydroxyoctadecadienoic acid	0.000623533	0.0000	0.0000	0.2284	map00591 Linoleic acid metabolism; map03320 PPAR signaling pathway
**8.20_291.1963m/z**	M-H2O-H[-]	C04785	13(S)-HPOT	0.000223533	0.0000	0.0000	0.2283	map00592 alpha-Linolenic acid metabolism
	M-H2O-H[-]	C16321	9(S)-HPOT	0.000223533	0.0000	0.0000	0.2283	map00592 alpha-Linolenic acid metabolism
**11.06_1162.4652m/z**	M+HCOO[-]	C16529	Lignoceroyl-CoA	0.002545000	0.0000	0.0001	0.2261	map01040 Biosynthesis of unsaturated fatty acids; map01212 Fatty acid metabolism
**10.01_293.2459m/z**	M-CO2+H[1+]	C02165	Leukotriene B4	0.001676467	0.0000	0.0000	0.2224	map00590 Arachidonic acid metabolism; map03320 PPAR signaling pathway
	M-CO2+H[1+]	C14808	Hepoxilin A3	0.001676467	0.0000	0.0000	0.2224	map00590 Arachidonic acid metabolism
	M-CO2+H[1+]	C05356	5(S)-Hydroperoxyeicosatetraenoic acid	0.001676467	0.0000	0.0000	0.2224	map00590 Arachidonic acid metabolism
	M-CO2+H[1+]	C05966	15(S)-HPETE	0.001676467	0.0000	0.0000	0.2224	map00590 Arachidonic acid metabolism
	M-CO2+H[1+]	C14781	15H-11,12-EETA	0.001676467	0.0000	0.0000	0.2224	map00590 Arachidonic acid metabolism
	M-CO2+H[1+]	C14813	11H-14,15-EETA	0.001676467	0.0000	0.0000	0.2224	map00590 Arachidonic acid metabolism

**10.92_184.0741m/z**	M[1+]	C00588	Phosphorylcholine	0.000200000	0.0000	0.0000	0.2197	map00564 Glycerophospholipid metabolism
**11.06_433.7534m/z**	M-C3H4O2+H[1+]	HMDB06455	Arachidonyl carnitine	0.002423533	0.0000	0.0000	0.2168	
**8.51_475.2708n**	M-HCOOH+H[1+]	C00621	Dolichyl diphosphate	0.003076467	0.0000	0.0000	0.2024	map00510 N-Glycan biosynthesis; map00900 Terpenoid backbone biosynthesis
**7.75_277.2167m/z**	M-C3H4O2+H[1+]	C05478	3,21-Dihydroxy-5-pregnane-11, 20-dione	0.000423533	0.0000	0.0000	0.1982	map00140 Steroid hormone biosynthesis
	M-C3H4O2+H[1+]	C05487	17,21-Dihydroxypreg-nenolone	0.000423533	0.0000	0.0000	0.1982	map00140 Steroid hormone biosynthesis
**11.08_412.7299m/z**	M-CO2+H[1+]	HMDB06460	Arachidyl carnitine	0.001576467	0.0000	0.0000	0.1914	
**9.04_353.2679m/z**	M+HCOONa[1+]	C01530	Stearic acid	0.001723533	0.0000	0.0000	0.1697	map01040 Biosynthesis of unsaturated fatty acids
**7.75_466.3175m/z**	M+K-2H[-]	C02477	Alpha-Tocopherol	0.004547066	0.0000	0.0000	0.1608	map00130 Ubiquinone and other terpenoid-quinone biosynthesis; map04977 Vitamin digestion and absorption
**10.89_846.5329m/z**	M+Cl37[-]	C00040	Acyl-CoA	0.004000000	0.0000	0.0000	0.1432	map00062 Fatty acid elongation; map00561 Glycerolipid metabolism; map00564 Glycerophospholipid metabolism; map04922 Glucagon signaling pathway; map04975 Fat digestion and absorption
	M+Cl37[-]	C00165	DG(16:0/16:0/0:0)	0.004000000	0.0000	0.0000	0.1432	map04066 HIF-1 signaling pathway; map04071 Sphingolipid signaling pathway; map04072 Phospholipase D signaling pathway; map04915 Estrogen signaling pathway; map04918 Thyroid hormone synthesis; map04925 Aldosterone synthesis and secretion; map04975 Fat digestion and absorption
**9.04_451.1796m/z**	M(S34)-H[-]	C00353	Geranylgeranyl pyrophosphate	0.002523533	0.0002	0.0011	0.0971	map00130 Ubiquinone and other terpenoid-quinone biosynthesis; map01062 Biosynthesis of terpenoids and steroids
	M(S34)-H[-]	C11356	Geranylneryl diphosphate	0.002523533	0.0002	0.0011	0.0971	map01062 Biosynthesis of terpenoids and steroids
**11.03_738.5100m/z**	M+Br81[-]	G00111	GD1a	0.001900000	0.0000	0.0000	0.0911	map00604 Glycosphingolipid biosynthesis
**12.65_688.5904m/z**	M-H[-]	G00094	Globoside	0.002323533	0.0000	0.0000	0.0456	map00603 Glycosphingolipid biosynthesis
	M-H[-]	G00093	Globotriaosylceramide	0.002323533	0.0000	0.0000	0.0456	map00603 Glycosphingolipid biosynthesis

Note: The intensity of the most abundant metabolites in females, and the intensity of the metabolites were “normalized”. Putatively identified using KEGG and HMDB.

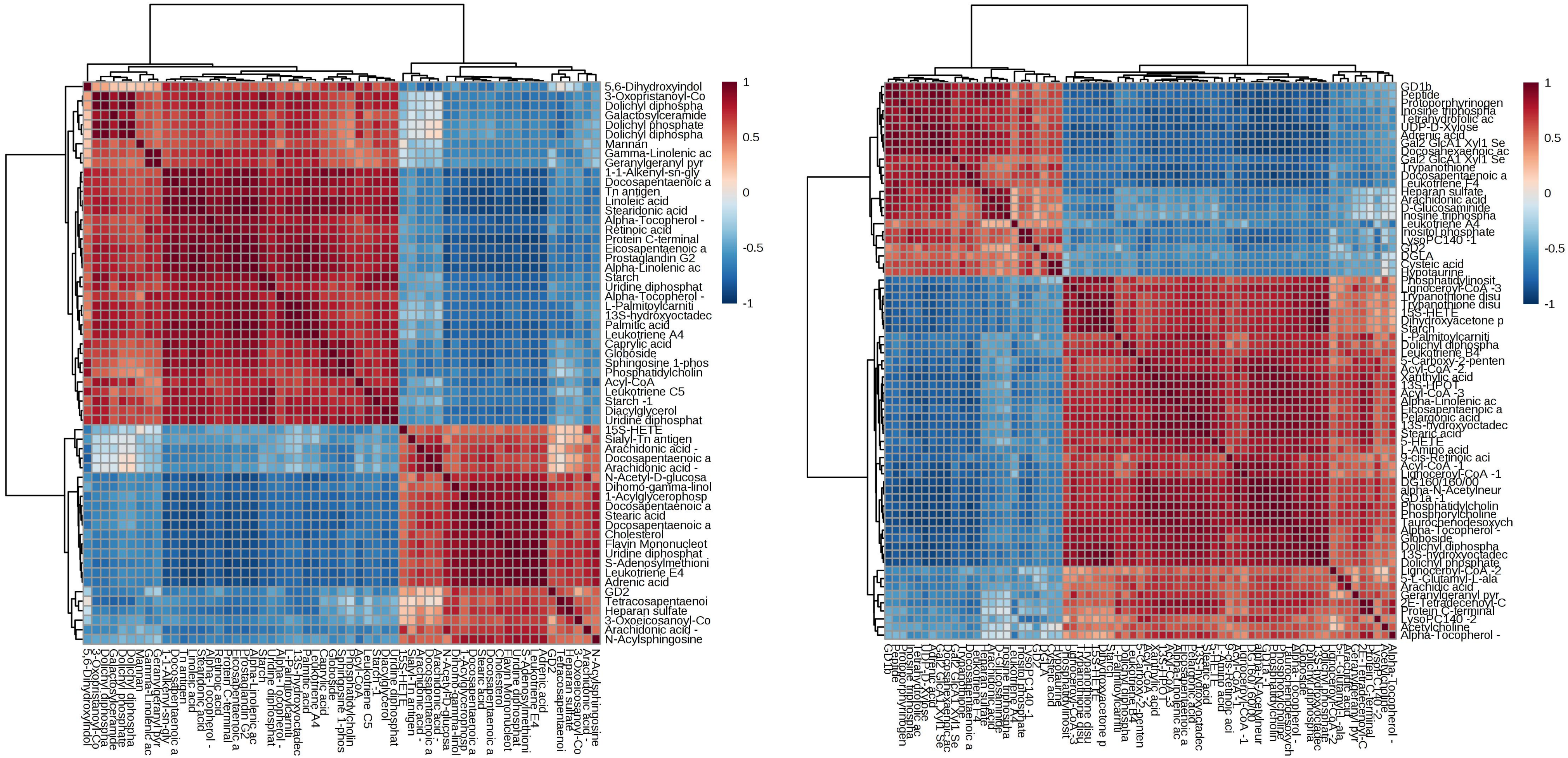

(A) **(B)**

Figure S2. The Pearson's correlation analyses for the discriminating metabolites of lipids and carbohydrates metabolisms in muscle tissues of female (A) and male (B) *C. idellus*, respectively.

Note: The differential signatures were annotated with their potential metabolite names after mapping with compound databases. The diversity of color referred to the pair-wise correlation coefficient ranging from 1 (red) to -1 (blue).

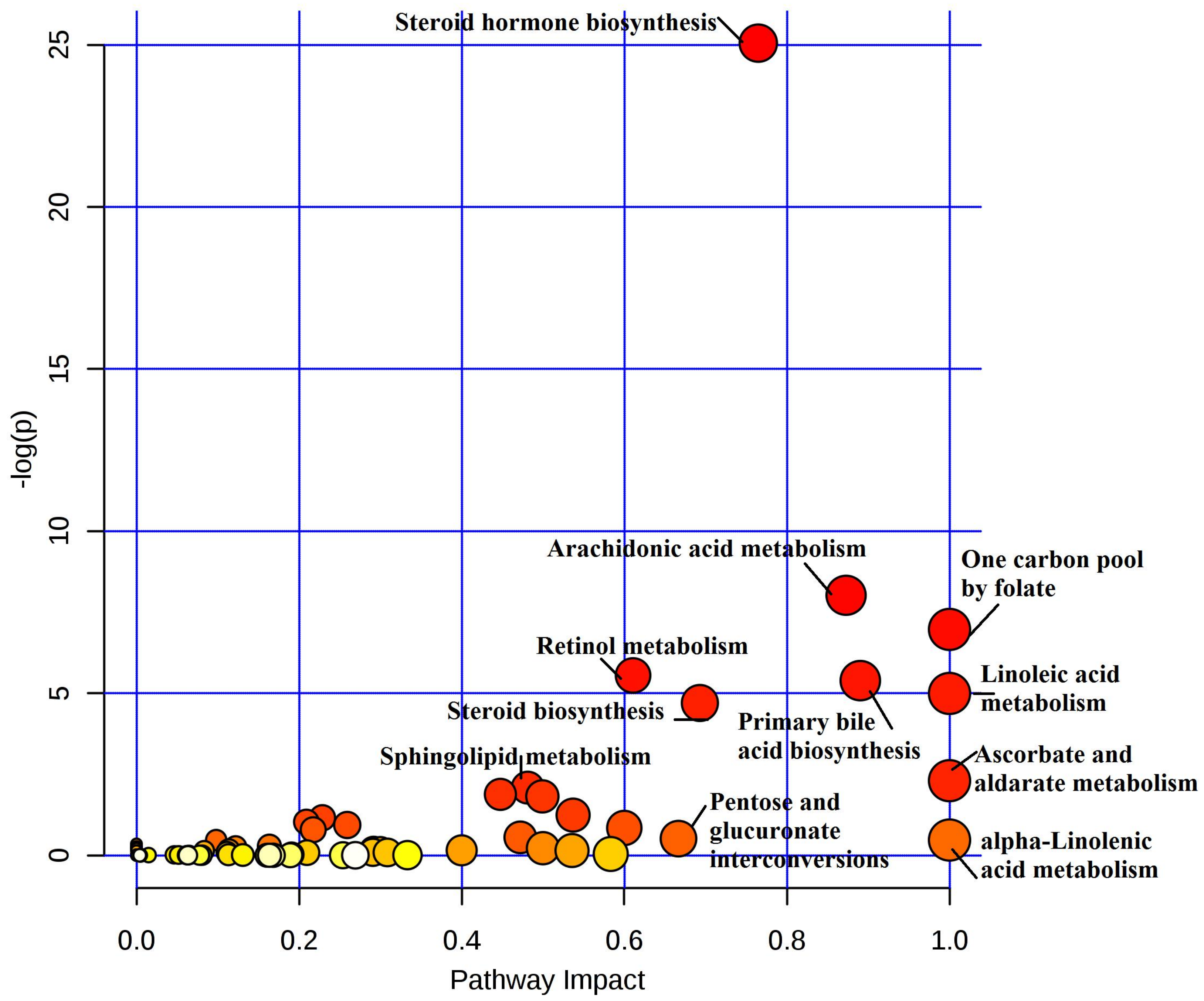

Steroid hormone biosynthesis
Arachidonic acid metabolism
One carbon pool by folate
Retinol metabolism
Linoleic acid metabolism
Steroid biosynthesis
Primary bile acid biosynthesis
Sphingolipid metabolism
Ascorbate and aldarate metabolism
Pentose and glucuronate interconversions
alpha-Linolenic acid metabolism
-log(p)
Pathway Impact
0
5
10
15
20
25
0.0
0.2
0.4
0.6
0.8
1.0

(A)

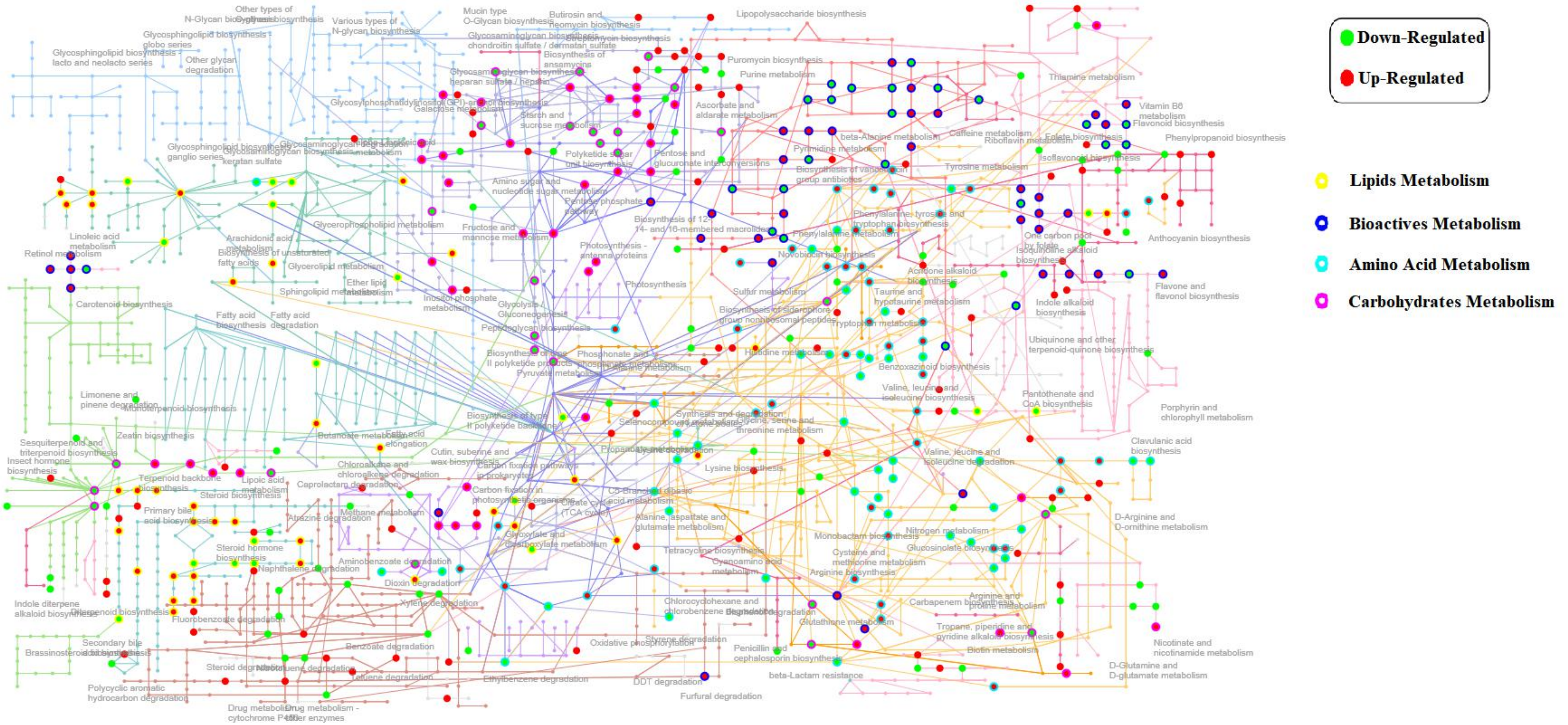

(B)

Figure S3. The pathway enrichment and network analyses for the significant metabolites in male *C. idellus*. (A) The scatter plot was used to visualize the pathway impact and enrichment results for all matching significant metabolites in male *C. idellus*; (B) The KEGG global metabolic network visualization of all significant metabolites (P < 0.05) in the male *C. idellus* metabolic profile.

Note: The colored points represent different metabolic pathways. The various color levels indicate different levels of significance of metabolic pathways from low (white) to high (red). The different sizes of each point were used to represent the number of metabolites participated in the metabolic pathway. The greater rich factor, the greater the degree of pathway enrichment.

Moreover the corresponding pathway's name of each point is labeled. In the metabolic network, all up-regulated metabolites (FC AF/GF > 2) in AF groups were colored with red, whereas the down regulated metabolites (FC < 0.5) were colored in green. In addition, the different color circles represent the various physiological functions that the discriminating metabolites belong to. Moreover, each enriched pathways is annotated with the corresponding name.

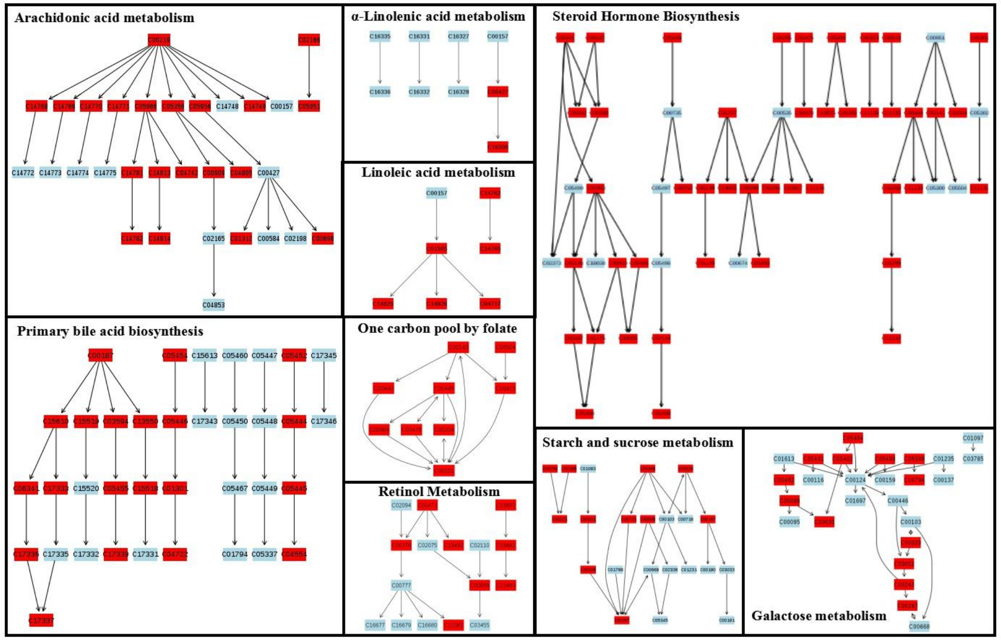
Arachidonic acid metabolism
α-Linolenic acid metabolism
Steroid Hormone Biosynthesis
Linoleic acid metabolism
Primary bile acid biosynthesis
One carbon pool by folate
Retinol Metabolism
Starch and sucrose metabolism
Galactose metabolism

(A)

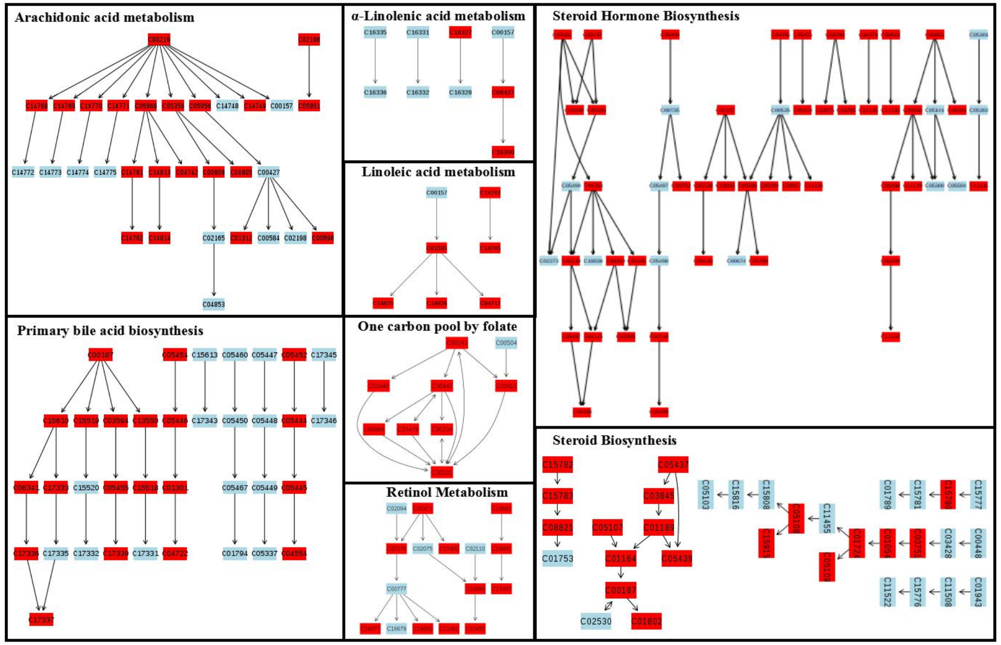
Arachidonic acid metabolism
α-Linolenic acid metabolism
Steroid Hormone Biosynthesis
Linoleic acid metabolism
Primary bile acid biosynthesis
One carbon pool by folate
Retinol Metabolism
Steroid Biosynthesis

(B)

Figure S4. Visualization of overlapped significant metabolites onto corresponding pathways.

Note: **(A)** The overlapped metabolites in female *C. idellus* highlighted in significantly enriched pathways; **(B)** The pathway view for the altered metabolites between MAF and MGF. Light blue compounds in the figures mean that these metabolites were undetected in our data, but used as background for pathway enrichment analysis. Red colored compounds mean the metabolites were detected in our metabolomic data and involved in the specific metabolism pathway.
