# Supplementary material for: Metabolomics investigation of dietary effects on flesh quality in grass carp (Ctenopharyngodon idellus)
Source: Gigascience. 2018 Sep 6;7(10):giy111. doi: 10.1093/gigascience/giy111 (PMC6176498; doi:10.1093/gigascience/giy111)
Supplement: Response_to_Reviewer_Comments_Report_(Original_Submission).pdf [file giy111_response_to_reviewer_comments_report_(original_submission).pdf]

Author's Response To Reviewer Comments

Close

Dear Dr. Nogoy and reviewers,

We gratefully appreciate the helpful comments and suggestions from the editor and reviewers. We have carefully revised our manuscript accordingly, changes in text are highlighted in yellow, and our item-wise replies are provided below.

Tim Young, Ph.D (Reviewer 1)

Major questions.:
1)Please clarify sample replication, as they are not currently sufficiently detailed to allow study reproduction. Two diets were tested, and three replicate pond systems per treatment group were used. However, it is not clear how the ten replicate fish muscle samples for metabolomics were obtained from this design; precisely how many fish per pond were sampled for metabolomics, and how exactly were they selected? Please provide similar information for the serum samples.
Response: Thank you for your valuable advice. The detailed descriptions of sample replications have been modified accordingly in text, which were also highlighted.
About 85 tails of C. idellus were caught from each pond. Meanwhile, the total number of fish from each experimental group was 250 (Line 454 to 455).
White muscle (including those used for both metabolic detections and histological sections) and gonadal tissues were taken from 250 tails per experimental group. (Line 467 to 468).
Blood samples were collected from 5 fish per pond, however, the final presented results were measured and calculated from 10 tails of fish (n = 10) per experimental group (Line 482).
In addition, the muscle samples used for LC-MS detections were randomly selected based on sex determinations. (Line 477 to 478). Based on sexes, metabolomic analysis of muscle samples were divided into 4 test groups (n = 10, each repetition mixed by 5 individuals), each repetition was taken from 5 individuals (approximately 25 mg per individual) (Line 5024 to 503).
Additionally, please note that the samples were selected based on the same cohort for metabolome detection, the corresponding sample numbers were same with samples used for metabolic tests (n = 50) (Line 491 to 493).

2)Please provide replication information (n = ?) in legends for all relevant figures and tables (i.e., Figs 2B & 4; Table 1 & 2).
Response: We agree that providing replication information to all relevant figures/tables is necessary and have modified the related legends and notes according to the reviewer's suggestions. In detail, we have provided the related replication information for Figures 1, 2, and 4, as well as Tables 1 and 2.

3)Figure 2B: Please state what data represent e.g., mean +/- SD or SE.
Response: We have clarified in the manuscript what the data in Figure 2B represents, providing the corresponding explanation in the figure legend: "Vertical bars represent the mean±S.E." (Line 774).

4) Were metabolite profile data normalized at all to biomass?

Response: Thank you for your question. We have described the related details concerning metabolomics sample collection in the manuscript. Each sample for metabolomic detection of muscle samples (n = 10) consisted of a mixture of 5 individuals (25 mg per individual) (Line 477-478). All replicate samples used for metabolome detection were therefore the same weight (125 mg).

5) Why were two different normalization approaches used for the multivariate analyses (i.e., auto-scaled for PCA and log transformed for heatmap and cluster analysis)?
Response: Thank you so much for pointing out this issue. In this study, we consistently used the log-transformation method during the analysis of metabolome profiles. We updated the corresponding text (Line 541 to 546):
" The log transformation and auto-scaling were also used in data normalization procedures. Univariate data analysis was applied to the metabolomics data using the Student's t-test. Statistical significance was set at $P < 0.05$ and $0.05 < P < 0.10$ as trends. Multiple testing corrections were performed based on false discovery rate (FDR)-adjusted p-values (q-values) with a significance threshold set at q-value < 0.05 [28]. For multivariate analysis, the data were subject to principle component analysis (PCA) for pattern discovery. For clustering analysis, a heatmap was created based on log-transformed relative intensities of detected features."

6) Please provide information on the exposure duration of MS-222, and to what level of anaesthesia the fish were dosed to.
Response: Thanks for the advice. We have provided further details of the anesthetic treatment as follows (Line 461-462): "Prior to sample collection, C. idellus were anesthetized by 100 $mg \cdot L^{-1}$ MS-222 (Sigma, St. Louis, Missouri, USA) for 2-4 minutes, then the growth performances were measured for each fish."

7)Line 271: It seems like a very large dataset of putative metabolite identifications was generated during this study, features of which may be of interest to some readers. This data is mentioned but not shown or provided - perhaps it might be useful to make it accessible. This is not essential for the manuscript, but it would make a nice addition.
Response: Thank you for your valuable suggestion. The metabolomics data have been submitted the data to MetaboLights and we intend to make it available to public. However, we may not be able to disclose this part of data for the time being. This is due to the fact that part of data being carried out comparison and cross-analysis with our other detected multiple-omics data. It may be used and presented in the follow-up research manuscript.

8) The title is misleading, and I would recommend re-wording it. I expected to see an analysis of correlations between the metabolite profiles and flesh quality. This was not the case; apart from fat content and muscle fiber thickness, other measures of fish flesh quality were not evaluated (e.g., appearance, taste, texture, gaping), and the experimental design does not appear to permit such correlations to be tested. I suggest authors make clear the difference between 'potential associations' and true 'correlations' throughout the manuscript. Indeed, a simple suggestion to make the title more reflective of the work being presented would be to just change the word 'correlations' to 'associations'.
Response: We appreciate the reviewer's reasonable proposal and recommendation for a more accurate title. In order to better summarize the results presented in this manuscript, we changed the title to "Metabolomics Investigation of Dietary Effects on Flesh Quality in Grass Carp (Ctenopharyngodon idellus)".
Furthermore, the basic experimental measurements of fish quality (including appearances,

textures, shear force detection, nutritional components in muscle tissues and so on) of the two feeding group Ctenopharyngodon idellus have been measured and analyzed in our previous work and have been summarized in two previously published articles. They both are used as references in this manuscript, Ref [21]: Zhao HH, Xia JG, Zhang X, He XG, Li L, Tang R, Chi W, Li DP. Diet Affects Muscle Quality and Growth Traits of Grass Carp (Ctenopharyngodon idellus): A Comparison Between Grass and Artificial Feed. Front. Physiol 2018; 9: 283-295; the other Ref [30] is published in Chinese, and compared the test results of nutritional components (e.g. 17 kinds of amino acids, pH, crude contents and 17 kinds of fatty acids) in muscle tissues of GF and AG fish.
Although these specific test results were not presented in this manuscript, we still had a correlation analysis between the current metabolomics detection results and the previous basic experimental results in the discussion section. We hope to fully analyze the differences in growth, physiology and metabolome between the GF and AF groups, as well as to reveal the potential regulatory mechanisms causing these differences, from epigenetic to molecular, and then to mechanism.
We agree with the reviewer’s comments that the “correlations” mentioned and revealed in our manuscript are only “potential links or associations”. Accordingly, in view of the major questions the reviewer raised, we further strengthened the descriptions of previously published research findings in the revised version, so that readers and researchers can easily and clearly understand and support the possible associations or related regulatory mechanisms revealed in our research. Accordingly, we have recited the data of flesh quality of fish under the same conditions we published recently, and further strengthened the discussion on the potential relations between flesh quality and muscle metabolites.

9)Related to the previous comment, components of the discussion are speculative and would benefit from revision. For example, authors state in Lines 317-319 that the higher levels of SFA's (i.e., free [?] C20:0, C18:0 and C16:0) in GF fish would result in a crisper taste and firmer flesh (thus altering taste, texture and quality). Although a citation is provided (albeit from cow meat which may not translate to fish), these indices were not actually tested and is therefore highly speculative in my opinion for a manuscript that centres itself largely on flesh quality. The language therefore should to be toned appropriately to reflect this. The manuscript could be suggestive and build upon the novelty of hypothesis generation, rather than projected to provide conclusive evidence.
Response: We thank the reviewer for their insightful comment. As mentioned in our answer to the previous question, The results of muscle characteristics detections (such as texture, amino acids and fatty acids in muscle tissues, as well as pH) of C. idellus fed with two different diets were summarized and represented in our two previously published articles, Ref [21] and Ref [30]. In these papers, a series of physiological indicators (e.g. growth rate, texture, shear force, pH, crude contents, and profiles of fatty acids and amino acids, as well as the expressions of the growth-related genes) were compared in muscle tissues of GF and AG fish. We therefore used these data as references to discuss in the manuscript. Although these specific measurements were not carried out in this paper, we conducted a correlation analysis between the current metabolomics detection results and the previous basic experimental results in the discussion section. We hope to fully analyze and reveal the potential regulatory mechanisms causing metabolic alterations and muscle characteristic differences, from epigenetic to molecular, then to metabolic pathway analyses.
Accordingly, we further strengthened the detailed descriptions of references and our previously published research findings in the revised version, so that readers and researchers can be easier, clearer, and support the possible associations or related regulatory mechanisms in our research.

Based on the reviewer's comment, the rewritten sentence is as follows: "As reported in other research, SFAs (e.g. C16:0 and C18:0) play important roles in influencing flesh texture, with higher levels resulting in a "crisper" flesh taste [30,44]. In line with the previous findings, the increased arachidic acid (20:0), stearic acid (18:0) and palmitic acid (16:0) were detected in our GF groups, therefore the flesh of GF C. idellus would be harder than that of AF fish. This conclusion is consistent and proved by our previously published test results of textures, shear force detection and fatty acid contents measurement of the two feeding groups C. idellus flesh [21, 30]." (Line 315 to 320)

10)Lines 308-309: This sentence should be re-phrased as it suggests that correlations between physiological changes and metabolic profiles were systematically studied by the authors. Unless I have missed some core information/dataset, I cannot see such correlation analyses. Also, please define 'physiological changes' if the phrase is to be used.
Response: We thank the reviewer for their recommendations. Accordingly, we have modified the text in manuscript. The corresponding sentence was changed to "Furthermore, the potential associations between physiological changes (the muscle characteristics, together with nutrients and textures measured in our previously published studies) [21,30] and metabolic profiles in different feeding conditions will be systematically further examined below." (Line 303 to 305).

11) Lines 419-421: Authors state that they correlated physiological, biochemical and metabolic parameters with specific parameters of flesh quality. Please re-state. Perhaps use the term 'associated' or 'linked' rather than correlated.
Response: Thanks for your advice. We modified the text in the manuscript according to your comment (Line 420 to 421), and enumerated the specific test parameters in order to give readers a better understanding of our narration.

12)Lines 422-423: The results indicate that flavour may have been affected, or perhaps even likely. However, a flavour analysis was not performed in this study. Please re-phrase accordingly.
Response: In view of the major questions you raised, we corrected and unified the word 'correlations' to 'associations' in the whole manuscript.
However, you pointed out the major problem, Although flavor or texture analysis was not performed in this work, a flavors analysis of the same fish in the two feeding group were conducted in our previous publish works (references [21] and [30] in this manuscript). Both studies had demonstrated that the flavors were affected by the two different feeding diets. Although these specific test results were not reproduced in this manuscript, we still correlated metabolomics alterations with the previously basic physiological and biochemical results in the discussion section. We hope to fully understand the differences in growth, physiology and metabolome of fish caused by feeding with grass and artificial feed, as well as reveal the potential regulatory mechanisms resulting in these differences, from epigenetic to molecular, then to mechanism.

13) Please highlight somewhere that the results of this particular study does not necessarily mean that all AF diets will perform worse than GF diets.
Response: We appreciate your reasonable proposal and agree with your point. Therefore, the related contents were added and the last conclusion section was rewritten as follows: "However, from commercial point of view, we still need to maintain the faster growths and higher yields of C. idellus contributed by artificial feeds. Based on the above considerations, we propose that feeding fish with both artificial feed and natural grass in a suitable

proportion will produce healthy, fast growing and high yield aquatic products. Further experiments are required to verify and to refine the feed in order to achieve optimal growth and health.” (Line 433 to 437).

14) Lines 438: What ratios by mass were these grasses fed to the fish?
Response: During the whole feeding experiment, we fully fed grass carp to satiation status. We weighed the fresh weight of the forage, not dehydrated. A total of 100 kg of L. perenne, E. pectinata and S. Sudanense were fed to GF group C. idellus each pond per day (Line 452).
On the other hand, the use of the three species of natural grasses is not determined by some specific collocation ratios. It depends on the time/ month course of the whole rearing experiment. Specifically, the best growth period of Lolium perenne is from May to July, that of Sorghum sudanense is from July to September. Compared with the other two grasses, Euphrasia pectinata has the longest suitable growth periods. However, its interlude of circular harvesting is very long, needs 3-4 weeks. Accordingly, our feeding experiment spanned from July 8th, 2016 to October 28th, 2016. During the experimental period, we fed fish with L. perenne at the beginning, it was planted in May, and S. Sudanense was planted at the same time. After July, S. Sudanense could be fed to our GF C. idellus. In addition, natural grasses feeding for all experimental fish should be fully fed. As a result, E. pectinata was repeatedly cut and intermittently fed to the fish during the 113 days.

15) Table S1: Correct the abbreviation for the 'Female-Grass feeding group' (i.e., FAF to FGF)
Response: I’m so sorry for this wrong abbreviation. It has been corrected.

Serap Saglik Aslan (Reviewer 2)

Comments to Author:
1.In the Abstract, the sentence that "DHA was only found in the male samples" is not impossible. Because DHA (Docosahexaenoic acid) is found in the fish tissues certainly. So If you can evidence this matter with a literatüre It may be acceptable. Otherwise There is a big mistake in the experimental of the study.
Response: We appreciate the reviewer’s comment. According to the question, we reconfirmed our metabolomics test results. This is caused by our inaccurate expression. The docosahexaenoic acid (DHA) was really detected in all of the four groups muscle samples (including FAF, FGF, MAF and MGF). Hence, we reworded this sentence as “Artificial feeding also resulted in significant increased docosahexaenoic acid (DHA) in MAF muscle than MGF fish, whereas no significance in the comparison of female samples.” (Line 43 to 45).
We mean that although DHA has been detected in the four groups samples, the comparisons of DHA between the two feeding patterns (FAF and FGF; MAF and MGF), it only showed significant difference in the comparison between MAF and MGF (q-value < 0.05). In other words, there is no significance in the relative intensities of DHA in FAF and FGF muscle samples (q-value > 0.05).

2.In the Materials and Methods, the title of "Experimental Design" must be changed as "Experimental part". Because experimental design has a different meaning. You can check in the literatüre.
Response: Sorry for this mistake. It has been corrected to “Experimental Procedures” (Line

447)

3.In Table 5, The total percentages are 125.9 for natural grass and 73.6 for artificial feed. 125.9 is impossible. Because; % moisture+%crude protein+%crude fat+ %crude fiber+%ash+%carbohydrate=100 in the proximate analysis. So it must be corrected.
Response: We thank the reviewer for pointing out this problem. In this experiment, the artificial feed is most frequently used in the aquaculture of farmed grass carp with known feed ingredient formula. Meanwhile, the natural grass is the traditional feed in the culture history of grass carp. Because the influence of exact nutrients in both diets on muscle characteristics and metabolites is not the final aim of our current study, we detected the crude nutrients in the two diets. In order to compare the specific and detected components between the two diets, we only listed the percentages of nutrients in artificial feeds corresponding to the detected contents in forage. Therefore, the percentages of many other detailed components in the AF (showed in the feed ingredient formula) were not listed. Consequently, the total percentage of artificial feeds used in our experiment is less than 100%.
For the natural grass, we weighed the fresh weight of the grasses under uncleaned and untreated status. As the grass were undehydrated, the soil composition could result in serious errors in the initial weighing, which will directly influence the accuracy of moisture detected data. Because we must present the original, available and real measured results, we removed the percentages of moisture in the two diets, making the overall feed nutrient compositions more reasonable, available, without affecting their authenticities.

Close
