# Supplementary material for: Metabolomics investigation of dietary effects on flesh quality in grass carp (Ctenopharyngodon idellus)
Source: Gigascience. 2018 Sep 6;7(10):giy111. doi: 10.1093/gigascience/giy111 (PMC6176498; doi:10.1093/gigascience/giy111)
Supplement: Reviewer_1_Report_(Original_Submission) -- Tim Young, Ph.D — 06/04/2018 Reviewed [file giy111_reviewer_1_report_(original_submission).pdf]

Reviewer Report

Title: **Metabolomics Investigation of Dietary Effects on Flesh Quality in Grass Carp (Ctenopharyngodon idellus)**

Version: **Original Submission** **Date:** 6/4/2018

Reviewer name: **Tim Young, Ph.D**

Reviewer Comments to Author:

In their manuscript, Honghao Zhao and colleagues report on various effects of feeding grass carp with a grass-based feed (GF) versus an artificially formulated high protein-based dried diet (AF). Measured effects include six indices to assess growth performance, intramuscular lipid content and fibre structure of abdominal muscle tissue, a targeted analysis of ten serum biomarkers typically used for veterinary screening/diagnostics, and an untargeted analysis of serum metabolites via an LC/MS-based metabolomics approach. Primary findings indicate that although an AF diet resulted in improved growth measures, it also lowered condition factor, led to an accumulation of fat via proliferation of adipocyte cells, decreased muscular fibre thickness and thus altered texture, increased serum triglyceride levels, and produced metabolite profiles that indicated key shifts in lipid and carbohydrate metabolism. Interpreting these findings together, authors suggest that the specific AF diet provided in this study negatively impacted fish fillet quality, nutritional content, and general fish wellbeing. Thus, authors further propose that a GF diet is better than an AF diet because it benefits the fish, and provides a higher quality and healthier product for the consumer. The manuscript is generally well written and organised, and the authors provide a clear impetus for the study. A paradigm shift away from highly intensive aquaculture farming practices to provide maximum yield, to one that focuses more on high quality / high value products with improved sustainability is occurring in many regions. Optimising diet and nutrition under farmed conditions will no doubt prove critical to achieving this aim. Long- term application of AF diets in carp aquaculture is suspected to have led to a decline in fillet quality, but the mechanisms and associations between dietary source, muscle metabolism, and flesh quality is poorly understood. Authors address this problem in part using a metabolomics approach that generated largescale metabolic data, and they link their findings well with previous work. The collection and curation of data was satisfactory, and I commend the authors for their transparency and provision for all supporting information. Complete data (including raw) was reportedly deposited in two online repositories (Metabolights and Github), but only one of these (Github) was accessible and verifiable to me at the time of review. Supporting data is available via GigaScience. Accessible links to data analysis tools (MetaboAnalyst 4.0) was provided, as was the history of R code during the analyses. The authors have reported their data to high standards. The experimental design and the data generally appear sound, pending on some details regarding replication being confirmed and made clearer (see specific comments below). This study took an exploratory metabolomics approach for the first time in this model, and as such in my opinion, provides novel data and associated interpretations to assist hypothesis generation rather than being conclusive in nature. Future interrogation of these interpretations and hypotheses will be a necessary next step. To this end, the manuscript would benefit from providing clear strategies for future research based on testable hypotheses. In addition, the interpretations of the data support and promote farmers to move from AF to GF diets. However, this study only tested one high protein and dried pellet-type AF diet compared to traditional feed-type, and it is certainly possible that other AF diets could be formulated in the future (if they do not already exist) to provide unique advantages. Authors should make this clear. There are some problems in how the authors have linked muscle tissue metabolite profiles to flesh quality, and there is no evidence to support that such a 'correlation' exists; indeed the experimental design was not appropriate to test specifically this (see specific comments below).The statistical methods for data analyses appear sound. PCA was used as an exploratory multivariate method, followed by t-test analysis with multiple hypothesis testing which is recommended for metabolomics data. Furthermore, authors placed constraints on their findings by only reporting upon metabolites which were had simultaneous p-values < 0.05 and foldchanges > 2. Pathway Analysis was performed with only those

features which passed this previous criterion, although they could have included all data. Authors perform pairwise correlation analyses between metabolites themselves, but not between metabolite abundances and measures of flesh quality as the title suggests. This needs addressing. Additionally, discussion on the differential metabolite correlations that exist between the experimental groups is sparse.Overall, this is an interesting and relevant study, and with some minor revisions should be a valuable resource for future research in fish aquaculture, nutrition and health. Some specific comments for consideration, in no particular order of importance:1) Please clarify sample replication, as they are not currently sufficiently detailed to allow study reproduction. Two diets were tested, and three replicate pond systems per treatment group were used. However, it is not clear how the ten replicate fish muscle samples for metabolomics were obtained from this design; precisely how many fish per pond were sampled for metabolomics, and how exactly were they selected? Please provide similar information for the serum samples. 2) Please provide replication information (n = ?) in legends for all relevant figures and tables (i.e., Figs 2B & 4; Table 1 & 2). 3) Figure 2B: Please state what data represent e.g., mean +/- SD or SE.4) Were metabolite profile data normalised at all to biomass?5) Why were two different normalisation approaches used for the multivariate analyses (i.e., autoscaled for PCA and log transformed for heatmap and cluster analysis)?6) Please provide information on the exposure duration of MS-222, and to what level of anaesthesia the fish were dosed to.7)Line 271: It seems like a very large dataset of putative metabolite identifications was generated during this study, features of which may be of interest to some readers. This data is mentioned but not shown or provided - perhaps it might be useful to make it accessible. This is not essential for the manuscript, but it would make a nice addition.8) The title is misleading, and I would recommend re-wording it. I expected to see an analysis of correlations between the metabolite profiles and flesh quality. This was not the case; apart from fat content and muscle fibre thickness, other measures of fish flesh quality were not evaluated (e.g., appearance, taste, texture, gaping), and the experimental design does not appear to permit such correlations to be tested. I suggest authors make clear the difference between 'potential associations' and true 'correlations' throughout the manuscript. Indeed, a simple suggestion to make the title more reflective of the work being presented would be to just change the word 'correlations' to 'associations'. 9)Related to the previous comment, components of the discussion are speculative and would benefit from revision. For example, authors state in Lines 317-319 that the higher levels of SFA's (i.e., free [?] C20:0, C18:0 and C16:0) in GF fish would result in a crisper taste and firmer flesh (thus altering taste, texture and quality). Although a citation is provided (albeit from cow meat which may not translate to fish), these indices were not actually tested and is therefore highly speculative in my opinion for a manuscript that centres itself largely on flesh quality. The language therefore should to be toned appropriately to reflect this. The manuscript could be suggestive and build upon the novelty of hypothesis generation, rather than projected to provide conclusive evidence.10) Lines 308-309: This sentence should be re-phrased as it suggests that correlations between physiological changes and metabolic profiles were systematically studied by the authors. Unless I have missed some core information/dataset, I cannot see such correlation analyses. Also, please define 'physiological changes' if the phrase is to be used. 11) Lines 419-421: Authors state that they correlated physiological, biochemical and metabolic parameters with specific parameters of flesh quality. Please re-state. Perhaps use the term 'associated' or 'linked' rather than correlated.12) Lines 422-423: The results indicate that flavour may have been affected, or perhaps even likely. However, a flavour analysis was not performed in this study. Please re-phrase accordingly.13) Please highlight somewhere that the results of this particular study does not necessarily mean that all AF diets will perform worse than GF diets.14) Lines 438: What ratios by mass were these grasses fed to the fish? 15) Table S1: Correct the abbreviation for the 'Female-Grass feeding group' (i.e., FAF to FGF)

Level of Interest

Please indicate how interesting you found the manuscript: Choose an item.

Quality of Written English

Please indicate the quality of language in the manuscript: Choose an item.

Declaration of Competing Interests

Please complete a declaration of competing interests, considering the following questions:

- Have you in the past five years received reimbursements, fees, funding, or salary from an organisation that may in any way gain or lose financially from the publication of this manuscript, either now or in the future?
- Do you hold any stocks or shares in an organisation that may in any way gain or lose financially from the publication of this manuscript, either now or in the future?
- Do you hold or are you currently applying for any patents relating to the content of the manuscript?
- Have you received reimbursements, fees, funding, or salary from an organization that holds or has applied for patents relating to the content of the manuscript?
- Do you have any other financial competing interests?
- Do you have any non-financial competing interests in relation to this paper?

If you can answer no to all of the above, write 'I declare that I have no competing interests' below. If your reply is yes to any, please give details below.

I declare that I have no competing interests

I agree to the open peer review policy of the journal. I understand that my name will be included on my report to the authors and, if the manuscript is accepted for publication, my named report including any attachments I upload will be posted on the website along with the authors' responses. I agree for my report to be made available under an Open Access Creative Commons CC-BY license (http://creativecommons.org/licenses/by/4.0/). I understand that any comments which I do not wish to be included in my named report can be included as confidential comments to the editors, which will not be published.

I agree to the open peer review policy of the journal

To further support our reviewers, we have joined with Publons, where you can gain additional credit to further highlight your hard work (see: https://publons.com/journal/530/gigascience). On publication of this paper, your review will be automatically added to Publons, you can then choose whether or not to claim your Publons credit. I understand this statement. Yes
